# Human papillomavirus (HPV) vaccination for the prevention of cervical cancer and other HPV‐related diseases: a network meta‐analysis

**DOI:** 10.1002/14651858.CD015364.pub2

**Published:** 2025-11-24

**Authors:** Hanna Bergman, Nicholas Henschke, Ingrid Arevalo-Rodriguez, Brian S Buckley, Emma J Crosbie, Jennifer C Davies, Kerry Dwan, Su P Golder, Yoon Kong Loke, Katrin Probyn, Jennifer Petkovic, Gemma Villanueva, Jo Morrison

**Affiliations:** Cochrane ResponseThe Cochrane CollaborationLondonUK; Department of SurgeryUniversity of the PhilippinesManilaPhilippines; Division of Cancer Sciences, Faculty of Biology, Medicine and HealthUniversity of ManchesterManchesterUK; Centre for Reviews and DisseminationUniversity of YorkYorkUK; Department of Health SciencesUniversity of YorkYorkUK; Norwich Medical SchoolUniversity of East AngliaNorwichUK; Bruyère Research InstituteUniversity of OttawaOttawaCanada; Department of Gynaecological OncologyMusgrove Park HospitalTauntonUK; Faculty of Health and Life SciencesUniversity of ExeterExeter, DevonUK

## Abstract

**Background:**

Cervical cancer is the fourth most common cause of cancer‐related death amongst females worldwide. Persistent infection with high‐risk human papillomavirus (HPV) is the key factor in cervical cancer development. HPV vaccines aim to prevent cancer by generating antibodies against HPV infection.

**Objectives:**

To evaluate the safety and efficacy of HPV vaccines, in females and males, to prevent cervical cancer and other HPV‐related diseases, in standard (pairwise) and network meta‐analysis (NMA) of randomised controlled trials.

**Search methods:**

On 10 January 2022, we searched the Cochrane Central Register of Controlled Trials (CENTRAL), MEDLINE and Embase. We searched Epistemonikos, ClinicalTrials.gov, WHO International Clinical Trials Registry Platform, the Health Technology Assessment database and vaccine manufacturer websites, and we checked reference lists from other relevant systematic reviews. We applied for Clinical Study Reports (CSRs) from the European Medicines Agency. An update search of electronic databases was done on 18 September 2024.

**Selection criteria:**

We included randomised controlled trials (RCTs) regardless of language or publication status, assessing HPV vaccines pre‐qualified by the World Health Organization (WHO) (Cervarix, Gardasil, Gardasil‐9 and Cecolin).

**Data collection and analysis:**

We used methods recommended by Cochrane. We primarily used CSRs to collect data, and we included outcome data irrespective of participants' baseline HPV infection or serostatus. We assessed risk of bias using the Cochrane tool (RoB 2). All outcomes were dichotomous, and we estimated risk ratios (RR) with 95% confidence intervals (CI). We used pairwise analysis for all outcomes. Where data were available, we carried out NMA for critical outcomes for networks in females and males in three age groups, ranking the vaccines using surface under the cumulative ranking curve (SUCRA) and mean ranks. We assessed the certainty of evidence using the GRADE approach.

**Main results:**

We included 60 individual studies with 157,414 participants ranging in follow‐up from seven months to 11 years. Few participants were under 15. There were no studies for males under 15 years and males over 25 years. We obtained CSRs for 33 of the included studies. We assessed the risk of bias as low to 'some concerns' for the critical outcomes.

**Cancer and pre‐cancer outcomes**

The studies were not of sufficient duration for cancers to develop. Four studies reported on cancer. No cancers were detected.

Critical pre‐cancer outcomes were reported in 15‐ to 25‐year‐old populations by 11 studies and in > 25‐year‐old females by three studies with up to seven years follow‐up. None were reported in the under 15 years age group.

In 15‐ to 25‐year‐old females, there was a reduction in CIN2+ irrespective of HPV type after six years (RR 0.70, 95% CI 0.56 to 0.88) (moderate‐certainty) and a larger reduction in CIN2+ from vaccine‐matched HPV types after six years (RR 0.40, 95% CI 0.30 to 0.54) (moderate‐certainty). In females over 25 years old, there was little to no difference between Cervarix and Gardasil compared with control (moderate‐certainty). There was no evidence on CIN2+ irrespective of HPV type from studies assessing Cecolin, or from studies assessing different dose schedules.

In 15‐ to 25‐year‐old females, there was a slight reduction in vaccine‐matched HPV‐type high‐grade vulval (VIN) or vaginal (VaIN) intraepithelial neoplasia following vaccination with Gardasil or Gardasil‐9 (moderate‐certainty). The NMA found a slight reduction of 1 case per 1000 following Gardasil (RR 0.21, 95% CI 0.1 to 0.45) and 0 cases per 1000 following Gardasil‐9 (RR 0.16, 95% CI 0.05 to 0.51). Little to no difference was found in the NMA for Cervarix compared with control (RR 0.28, 95% CI 0.06 to 1.37), or for Cervarix, Gardasil and Gardasil‐9 compared to each other.

There was a reduction in high‐grade anal intraepithelial neoplasia (AIN) irrespective of HPV type in the Gardasil group in one study in men who have sex with men (RR 0.75, 95% CI 0.53 to 1.07) (low‐certainty).

For both high‐grade penile intraepithelial neoplasia (PeIN) irrespective of HPV type and vaccine‐matched HPV‐type high‐grade PeIN, little to no difference per 1000 participants was reported in the Gardasil group in one study with 3880 participants at 36 months follow‐up (RR 1.00, 95% CI 0.20 to 4.93) (low‐certainty).

**Serious adverse events**

In a pairwise analysis of serious adverse events in 39 studies across all vaccine types with 97,272 participants, there was little to no difference in the HPV vaccine groups compared with the control group at up to 72 months follow‐up (RR 0.99, 95% CI 0.94 to 1.04) (high‐certainty).

**Treatment rates for HPV‐related pre‐invasive disease**

In pairwise analysis of five studies with 38,606 participants, there were 12 fewer people that needed to seek treatment per 1000 participants (95% CI 5 to 17 fewer per 1000) in the HPV vaccine groups compared with the control group rate at up to 84 months follow‐up (RR 0.76, 95% CI 0.65 to 0.89) (moderate‐certainty).

**Anogenital warts**

In pairwise analysis of three studies with 21,271 participants, there were 25 fewer cases of anogenital warts irrespective of HPV type per 1000 participants (95% CI 22 to 28 fewer per 1000) in the HPV vaccine groups compared with the control group rate at up to 48 months follow‐up (RR 0.38, 95% CI 0.32 to 0.46) (high‐certainty). In the NMA for females 15 to 25 years old, Gardasil‐9 was most likely to reduce the risk of developing anogenital warts.

**Authors' conclusions:**

The evidence in this network meta‐analysis of HPV vaccines is based on extensive searches and analyses. There is evidence from randomised controlled trials that HPV vaccination reduces the risk of pre‐cancerous outcomes such as CIN2+ and anogenital warts. No data were available for cervical cancer or other cancer outcomes, and no data on pre‐cancer outcomes were available for vaccination under age 15 years. There were no safety concerns noted in the studies.

## Plain language summary

**What are the benefits and risks of different human papillomavirus (HPV) vaccines for preventing cervical cancer and other HPV‐related diseases?**

**Key messages**

‐ In women and girls aged 15 to 25 years receiving human papillomavirus (HPV) vaccination with Cervarix, Gardasil or Gardasil‐9, compared with no HPV vaccination, there was a slight reduction in high‐grade **cervical** pre‐cancer (abnormal‐looking cells that may become cancer if left untreated) after four to six years. In those vaccinated with Gardasil or Gardasil‐9 compared with no vaccination, there was a small reduction in pre‐cancer of the **vulva and vagina** after four years. HPV vaccination reduced the risk of anogenital warts and the need for treatment for HPV‐related disease. No study lasted long enough to report on the development of cancers.

‐ Some pain and swelling at the injection site were common after HPV vaccination, but we found no serious unwanted effects.

‐ Most studies included people aged 15 years and over, who are more likely to have been exposed to the infection and therefore less likely to benefit from vaccination. The trials were too short to measure long‐term outcomes like the development of cancer.

**What is human papillomavirus (HPV)?**

HPV is a common infection. It is transmitted between people through close contact, including vaginal, anal or oral sex. There are many types of HPV. Some types can cause cancer. Cervical cancer is the most common type, but it can also cause vaginal, vulval, penile, anal, and head and neck cancer, and anogenital warts, so HPV can affect both men and women. Cervical cancer usually takes more than 10 years to develop after initial infection. Other HPV‐related cancers develop more slowly.

**How can HPV vaccines help?**

HPV vaccines aim to prevent the types of HPV infections that sometimes cause cancers and anogenital warts. The HPV vaccines do not work as well in people that have already been exposed to HPV, so most vaccination programmes are for young people before they become sexually active.

**What did we want to find out?**

We wanted to find out if HPV vaccines:

‐ prevent cancers and pre‐cancers (abnormal‐looking cells that may become cancer if left untreated);

‐ reduce the need for treatment of HPV‐related disease;

‐ prevent anogenital warts; and

‐ cause any unwanted effects.

**What did we do?**

We searched for studies that compared:

‐ an HPV vaccine with either a placebo (a dummy vaccine), a non‐HPV vaccine or no vaccine; or

‐ different HPV vaccines or number of doses of HPV vaccines.

We compared and summarised the results, and rated our confidence in the evidence, based on factors such as study methods and sizes. We were supported by an Independent Advisory Group, including consumers.

**What did we find?**

We found 60 studies with 157,414 people. The biggest study involved 34,412 people and the smallest 11 people. They were followed from 4 days to 11 years. Studies took place around the world. Most lasted for 12 months. Pharmaceutical companies funded 44 of the studies.

**Main results**

‐ The studies were not long enough to tell us directly about prevention of cancers and focussed on shorter‐term outcomes.

‐ In women and girls aged 15 to 25 years, Cervarix and Gardasil reduced all high‐grade cervical pre‐cancer (CIN2+) in the short term. In women over 25 years, there was little to no difference.

‐ In people aged 15 to 25 years, there was little to no difference in high‐grade anal or penile pre‐cancer in the short term. Gardasil and Gardasil‐9 vaccines reduced high‐grade vaginal or vulval pre‐cancer in this group of people.

‐ HPV vaccination reduced the risk of anogenital warts and the number of people aged 15 to 25 years needing treatment for possible early‐stage HPV‐related cancer.

‐ Some pain and swelling were common after all HPV vaccines, but there were no serious unwanted effects. We do not know whether there are differences in risks between different vaccines.

**What are the limitations of the evidence?**

We had some concerns about the way some studies were done, which might affect some results. We are moderately confident in the evidence for serious unwanted effects, treatment of HPV‐related disease, cervical, vaginal and vulval pre‐cancer, and anogenital warts. We are less confident in the evidence on cancer and penile and anal pre‐cancer because there were few cases, studies were too short to measure cancer and people in the studies were older than people who receive the vaccines.

Many studies were industry‐funded, but we found no differences compared with independently funded studies.

**How up‐to‐date is this evidence?**

The evidence is current to 18 September 2024.

## Summary of findings

**Summary of findings 1:** Summary of findings: Efficacy and safety of any HPV vaccine compared with control (standard meta‐analysis)

**Population:** males and females of all ages **Interventions:** Cervarix, Gardasil, Gardasil‐9, Cecolin **Comparators:** injection control (saline placebo, adjuvant placebo, non‐HPV control vaccine), no intervention						
**Outcome**		**N participants (trials)**	Anticipated absolute effect*		**Relative effect** **RR (95% CI)**	**Certainty of the evidence** **(GRADE)**
			**With control**	**With HPV vaccine**		
Invasive cervical cancer Follow‐up: up to 6 years		Two trials with 17,662 participants reported on this outcome and detected zero events.				
Invasive vulval or vaginal cancer positive for vaccine‐type HPV Follow‐up: up to 6 years		One trial with 5455 participants reported on this outcome and detected zero events.**				
Invasive anal cancer Follow‐up: 36 months		One trial with 551 participants reported on this outcome and detected zero events.				
Invasive penile cancer Follow‐up: 36 months		One trial with 3880 participants reported on this outcome and detected zero events.				
Invasive head and neck cancer		No trials reported on this outcome.				
High‐grade CIN Follow‐up: up to 84 months	CIN3+ irrespective of HPV type Follow‐up: up to 72 months	43,901 (6)	23 per 1000	18 per 1000 (13 to 26)	0.78 (0.55 to 1.12)	LOW^a,b^
			5 fewer cases per 1000 participants (11 fewer to 3 more)			
	CIN3+ positive for vaccine‐type HPV Follow‐up: up to 48 months	35,655 (4)	16 per 1000	9 per 1000 (7 to 11)	0.54 (0.44 to 0.65)	MODERATE^c^
			8 fewer cases per 1000 participants (6 to 9 fewer)			
	CIN2+ irrespective of HPV type Follow‐up: up to 84 months	55,911 (7)	46 per 1000	36 per 1000 (13 to 26)	0.79 (0.65 to 0.96)	MODERATE^a,d^
			10 fewer cases per 1000 participants (2 to 16 fewer)			
	CIN2+ positive for vaccine‐type HPV Follow‐up: up to 72 months	59,717 (10)	25 per 1000	13 per 1000 (10 to 16)	0.50 (0.39 to 0.65)	MODERATE^e,f^
			13 fewer cases per 1000 participants (9 to 15 fewer)			
High‐grade VIN or VaIN positive for vaccine‐type HPV Follow‐up: up to 48 months		36,873 (5)	3 per 1000	1 per 1000 (0 to 3)	0.35 (0.10 to 1.24)	MODERATE^g,h^
			2 fewer cases per 1000 participants (2 fewer to 1 more)			
High‐grade AIN irrespective of HPV type Follow‐up: 36 months		551*** (1)	214 per 1000	160 per 1000 (113 to 229)	0.75 (0.53 to 1.07)	LOW^i,j^
			54 fewer cases per 1000 participants (101 fewer to 15 more)			
High‐grade AIN positive for vaccine‐type HPV Follow‐up: 36 months		551*** (1)	141 per 1000	65 per 1000 (38 to 112)	0.46 (0.27 to 0.79)	LOW^j,k^
			76 fewer cases per 1000 participants (30 to 103 fewer)			
High‐grade PeIN irrespective of HPV type Follow‐up: 36 months		3880 (1)	2 per 1000	2 per 1000 (0 to 8)	1.00 (0.20 to 4.93)	LOW^l^
			No difference per 1000 participants (1 fewer to 6 more)			
High‐grade PeIN positive for vaccine‐type HPV Follow‐up: 36 months		3880 (1)	2 per 1000	2 per 1000 (0 to 8)	1.00 (0.20 to 4.93)	LOW^l^
			No difference per 1000 participants (1 fewer to 6 more)			
Need to seek treatment for HPV‐related pre‐invasive disease Follow‐up: up to 84 months		38,604 (5)	49 per 1000	37 per 1000 (32 to 43)	0.76 (0.65 to 0.89)	MODERATE^a,m^
			12 fewer cases per 1000 participants (5 to 17 fewer)			
Anogenital warts Follow‐up: up to 48 months		21,271 (3)	41 per 1000	16 per 1000 (13 to 19)	0.38 (0.32 to 0.46)	HIGH
			25 fewer cases per 1000 participants (22 to 28 fewer)			
Serious adverse events Follow‐up: up to 72 months		97,272 (39)	57 per 1000	56 per 1000 (54 to 59)	0.99 (0.94 to 1.04)	HIGH
			1 less case per 1000 participants (3 fewer to 2 more)			
**GRADE Working Group grades of evidence** High certainty: We are very confident that the true effect lies close to that of the estimate of the effect. Moderate certainty: We are moderately confident in the effect estimate: The true effect is likely to be close to the estimate of the effect, but there is a possibility that it is substantially different. Low certainty: Our confidence in the effect estimate is limited: The true effect may be substantially different from the estimate of the effect. Very low certainty: We have very little confidence in the effect estimate: The true effect is likely to be substantially different from the estimate of effect.						

**Abbreviations**: AIN: anal intraepithelial neoplasia; CI: confidence interval; CIN3+: cervical intraepithelial neoplasia, grade 3 or worse (including CIN grade 3, adenocarcinoma *in situ*, and invasive cervical cancer); HPV: human papillomavirus; PeIN: penile intraepithelial neoplasia; RR: risk ratio; VaIN: vaginal intraepithelial neoplasia; VIN: vulval intraepithelial neoplasia High‐grade intraepithelial neoplasia refers to grade 2 or 3. *Anticipated absolute effect compares two risks by calculating the difference between the risks of the intervention groups with the risk of the control group. **One case of non‐HPV perineal cancer was reported in the Gardasil group at 24 months, negative for HPV vaccine types and 10 other oncogenic HPV types. ***Measured and reported in a pre‐planned subgroup of men who have sex with men. ^a^Downgraded one level for risk of bias due to some concerns regarding deviations from intended intervention, missing data and selective reporting. ^b^Downgraded one level for inconsistency: one study had an effect estimate in the opposite direction compared to the other studies, leading to substantial heterogeneity (I^2^ = 74%). ^c^Downgraded one level for risk of bias due to some concerns regarding missing data and selective reporting. ^d^Although there was substantial heterogeneity (I^2^ = 76%), this may be explained by subgroup differences (I^2^ = 83.0%) between 15‐ to 25‐year‐old (RR 0.70, 95% CI 0.56 to 0.88) and over 25‐year‐old (RR 1.04, 95% CI 0.83 to 1.30) subgroups (not downgraded for inconsistency). ^e^Downgraded one level for risk of bias due to some concerns regarding mainly missing data and selective reporting. ^f^Although there was substantial heterogeneity (I^2^ = 54%), this may be explained by subgroup differences (I^2^ = 87.5%) between 15‐ to 25‐year‐old (RR 0.40, 95% CI 0.30 to 0.54) and over 25‐year‐old (RR 0.73, 95% CI 0.54 to 0.98) subgroups (not downgraded for inconsistency). ^g^Downgraded one level for risk of bias due to some concerns regarding randomisation process, deviations from intended intervention, missing data and selective reporting. ^h^Although there was substantial heterogeneity (I^2^ = 50%), this may be explained by subgroup differences (I^2^ = 74.1%) between 15‐ to 25‐year‐old (RR 0.22, 95% CI 0.11 to 0.42) and over 25‐year‐old (RR 4.98, 95% CI 0.24 to 103.58) subgroups (not downgraded for inconsistency). ^i^Downgraded one level for imprecision due to few events and participants, leading to wide 95% CIs around the absolute effect. ^j^Downgraded one level for indirectness: measured and reported in a high‐risk subgroup (men who have sex with men). ^k^Downgraded one level for imprecision due to few events and participants. ^l^Downgraded two levels for imprecision due to very few events and few participants. ^m^Although there was substantial heterogeneity (I^2^ = 62%), all effect estimates were in the same direction (favouring HPV vaccine), so we did not downgrade for inconsistency.

**Summary of findings 2:** Summary of findings: High‐grade CIN by population, sub‐outcome and vaccine

**Population:** females of all ages **Interventions:** Cervarix, Gardasil, Gardasil‐9, Cecolin **Comparator:** injection control (saline placebo, adjuvant placebo, non‐HPV control vaccine), Cervarix, Gardasil **Outcome:** high‐grade CIN (composite outcome, includes: CIN grade 2, CIN grade 3, adenocarcinoma in situ, invasive cervical cancer)										
		**Direct evidence**					**Network meta‐analysis**			
**Sub‐outcome**	**Comparison**	**N participants (studies)**	Anticipated absolute effect*		**Relative effect RR (95% CI)**	**Certainty of the evidence** **(GRADE)**	Anticipated absolute effect*		**Relative effect** **RR (95% CI)**	**Certainty of the evidence** **(GRADE)**
			**With control**	**With vaccine**			**With control**	**With vaccine**		
***Females 14 years or younger:***no studies reported on this outcome for this population group.										
***Females 15 to 25 years***										
CIN3+ irrespective of HPV type	Cervarix vs injection control Follow‐up: 4 to 6 years	26,741 (4)	16 per 1000	12 per 1000 (7 to 23)	0.76 (0.41 to 1.41)	VERY LOW^a,b,c^	23 per 1000	18 per 1000 (10 to 31)	0.76 (0.44 to 1.34)	VERY LOW^a,c,d^
			4 fewer per 1000 (9 fewer to 7 more)				6 fewer per 1000 (13 fewer to 8 more)			
CIN3+ positive for vaccine‐type HPV		18,495 (2)	10 per 1000	5 per 1000 (4 to 8)	0.54 (0.39 to 0.76)	HIGH	16 per 1000	9 per 1000 (6 to 13)	0.55 (0.39 to 0.77)	HIGH
			5 fewer per 1000 (from 2 to 6 fewer)				7 fewer per 1000 (from 4 to 10 fewer)			
CIN2+ irrespective of HPV type		29,464 (4 studies)	41 per 1000	26 per 1000 (18 to 37)	0.63 (0.44 to 0.90)	LOW^a,c^	41 per 1000	25 per 1000 (16 to 39)	0.61 (0.39 to 0.95)	LOW^a,c^
			15 fewer per 1000 (from 4 to 23 fewer)				16 fewer per 1000 (from 2 to 25 fewer)			
CIN2+ positive for vaccine‐type HPV		27,302 (5)	23 per 1000	8 per 1000 (5 to 11)	0.33 (0.21 to 0.50)	MODERATE^e^	31 per 1000	11 per 1000 (4 to 26)	0.34 (0.14 to 0.81)	MODERATE^e^
			15 fewer per 1000 (from 11 to 18 fewer)				21 fewer per 1000 (from 6 to 27 fewer)			
CIN3+ irrespective of HPV type	Gardasil vs injection control Follow‐up: 4 years	17,160 (2)	35 per 1000	28 per 1000 (24 to 33)	0.81 (0.69 to 0.96)	HIGH	23 per 1000	19 per 1000 (8 to 47)	0.81 (0.33 to 2.00)	LOW^f^
			7 fewer per 1000 (1 to 11 fewer)				4 fewer per 1000 (16 fewer to 23 more)			
CIN3+ positive for vaccine‐type HPV		17,160 (2)	23 per 1000	12 per 1000 (10 to 16)	0.54 (0.43 to 0.68)	HIGH	16 per 1000	9 per 1000 (7 to 11)	0.54 (0.43 to 0.68)	HIGH
			11 fewer per 1000 (7 to 13 fewer)				8 fewer per 1000 (5 to 9 fewer)			
CIN2+ irrespective of HPV type		17,160 (2)	60 per 1000	49 per 1000 (44 to 56)	0.81 (0.72 to 0.92)	HIGH	60 per 1000	49 per 1000 (23 to 105)	0.81 (0.38 to 1.73)	LOW^f^
			11 fewer per 1000 (from 5 to 17 fewer)				11 fewer per 1000 (from 37 fewer to 44 more)			
CIN2+ positive for vaccine‐type HPV		17,160 (2)	44 per 1000	22 per 1000 (18 to 26)	0.50 (0.42 to 0.59)	HIGH	31 per 1000	16 per 1000 (10 to 24)	0.50 (0.33 to 0.76)	HIGH
			22 fewer per 1000 (from 18 to 25 fewer)				16 fewer per 1000 (from 8 to 21 fewer)			
CIN3+ irrespective of HPV type	Gardasil‐9 vs injection control Follow‐up: 4 to 6 years	No studies with direct or indirect comparison								
CIN3+ positive for vaccine‐type HPV		No studies with direct comparison					16 per 1000	0 per 1000 (0 to 9)	0.03 (0.00 to 0.55)	MODERATE^a^
							16 fewer per 1000 (7 to 16 fewer)			
CIN2+ irrespective of HPV type		No studies with direct comparison					48 per 1000	39 per 1000 (13 to 115)	0.82 (0.28 to 2.39)	VERY LOW^a,f^
							9 fewer per 1000 (from 35 fewer to 67 more)			
CIN2+ positive for vaccine‐type HPV		No studies with direct comparison					31 per 1000	15 per 1000 (9 to 28)	0.49 (0.27 to 0.89)	MODERATE^a^
							16 fewer per 1000 (3 to 23 fewer)			
CIN3+ irrespective of HPV type	Cecolin vs injection control	No studies with direct or indirect comparison								
CIN3+ positive for vaccine‐type HPV		No studies with direct or indirect comparison								
CIN2+ irrespective of HPV type		No studies with direct or indirect comparison								
CIN2+ positive for vaccine‐type HPV		No studies with direct or indirect comparison								
CIN3+ irrespective of HPV type	Gardasil vs Cervarix Follow‐up: 4 to 6 years	No studies with direct comparison					11 per 1000	12 per 1000 (4 to 35)	1.06 (0.37 to 3.07)	LOW^a,g^
							1 more per 1000 (7 fewer to 23 more)			
CIN3+ positive for vaccine‐type HPV		No studies with direct comparison					10 per 1000	10 per 1000 (7 to 15)	0.98 (0.65 to 1.49)	MODERATE^h^
							0 more per 1000 (from 4 fewer to 5 more)			
CIN2+ irrespective of HPV type		No studies with direct comparison					28 per 1000	37 per 1000 (15 to 90)	1.32 (0.55 to 3.18)	VERY LOW^a,f^
							9 more per 1000 (from 13 fewer to 61 more)			
CIN2+ positive for vaccine‐type HPV							7 per 1000	10 per 1000 (4 to 26)	1.47 (0.56 to 3.90)	LOW^a,g^
							3 more per 1000 (from 3 fewer to 19 more)			
CIN3+ irrespective of HPV type	Gardasil‐9 vs Cervarix Follow‐up: 4 to 6 years	No studies with direct or indirect comparison								
CIN3+ positive for vaccine‐type HPV		No studies with direct comparison					10 per 1000	1 per 1000 (0 to 10)	0.06 (0.00 to 1.03)	MODERATE^a^
							10 fewer per 1000 (from 10 fewer to 0 more)			
CIN2+ irrespective of HPV type		No studies with direct comparison					28 per 1000	38 per 1000 (12 to 120)	1.34 (0.42 to 4.26)	VERY LOW^a,f^
							10 more per 1000 (from 16 fewer to 92 more)			
CIN2+ positive for vaccine‐type HPV		No studies with direct comparison					7 per 1000	10 per 1000 (3 to 28)	1.46 (0.51 to 4.21)	LOW^a,g^
							3 more per 1000 (from 3 fewer to 21 more)			
CIN3+ irrespective of HPV type	Gardasil‐9 vs Gardasil Follow‐up: 6 years	No studies with direct or indirect comparison								
CIN3+ positive for vaccine‐type HPV		11,892 (1)	1 per 1000	0 per 1000 (0 to 1)	0.06 (0.00 to 1.02)	MODERATE^a^	8 per 1000	0 per 1000 (0 to 8)	0.06 (0.00 to 1.02)	MODERATE^a^
			1 fewer per 1000 (from none to 1 fewer)				7 fewer per 1000 (from none to 8 fewer)			
CIN2+ irrespective of HPV type		13,754 (1)	51 per 1000	51 per 1000 (44 to 60)	1.01 (0.87 to 1.17)	HIGH	51 per 1000	51 per 1000 (24 to 110)	1.01 (0.47 to 2.16)	LOW^f^
			0 fewer per 1000 (from 7 fewer to 9 more)				0 fewer per 1000 (from 27 fewer to 59 more)			
CIN2+ positive for vaccine‐type HPV		13,754 (1)	41 per 1000	41 per 1000 (35 to 48)	0.99 (0.84 to 1.16)	HIGH	31 per 1000	30 per 1000 (20 to 46)	0.99 (0.65 to 1.52)	LOW^f^
			0 fewer per 1000 (from 7 fewer to 7 more)				0 fewer per 1000 (from 11 fewer to 16 more)			
***Females 25 years or older***										
CIN3+ irrespective of HPV type	Cervarix vs injection control Follow‐up: 7 years	No studies with direct or indirect comparison								
CIN3+ positive for vaccine‐type HPV		No studies with direct or indirect comparison								
CIN2+ irrespective of HPV type		5468 (1)	39 per 1000	38 per 1000 (29 to 49)	0.95 (0.73 to 1.24)	MODERATE^d^	No NMA			
			2 fewer per 1000 (from 11 fewer to 9 more)							
CIN2+ positive for vaccine‐type HPV		5477 (1)	16 per 1000	12 per 1000 (7 to 18)	0.71 (0.45 to 1.11)	HIGH	17 per 1000	12 per 1000 (8 to 19)	0.71 (0.45 to 1.11)	HIGH
			5 fewer per 1000 (from 9 fewer to 2 more)				5 fewer per 1000 (from 9 fewer to 2 more)			
CIN3+ irrespective of HPV type	Gardasil vs injection control Follow‐up: 4 years	No studies with direct or indirect comparison								
CIN3+ positive for vaccine‐type HPV		No studies with direct or indirect comparison								
CIN2+ irrespective of HPV type		3819 (1)	27 per 1000	32 per 1000 (22 to 47)	1.21 (0.84 to 1.75)	MODERATE^g^	No NMA			
			6 more per 1000 (from 4 fewer to 20 more)							
CIN2+ positive for vaccine‐type HPV		6778 (2)	17 per 1000	13 per 1000 (9 to 19)	0.74 (0.50 to 1.09)	MODERATE^i^	17 per 1000	12 per 1000 (8 to 18)	0.74 (0.50 to 1.09)	MODERATE^i^
			4 fewer per 1000 (from 9 fewer to 2 more)				4 fewer per 1000 (from 8 fewer to 2 more)			
CIN3+ irrespective of HPV type	Gardasil vs Cervarix Follow‐up: 4 to 7 years	No studies with direct or indirect comparison								
CIN3+ positive for vaccine‐type HPV		No studies with direct or indirect comparison								
CIN2+ irrespective of HPV type		No studies with direct or indirect comparison								
CIN2+ positive for vaccine‐type HPV		No studies with direct comparison					12 per 1000	12 per 1000 (7 to 22)	1.04 (0.57 to 1.89)	MODERATE^i^
							0 fewer per 1000 (from 5 fewer to 10 more)			
**GRADE Working Group grades of evidence** High certainty: We are very confident that the true effect lies close to that of the estimate of the effect. Moderate certainty: We are moderately confident in the effect estimate: The true effect is likely to be close to the estimate of the effect, but there is a possibility that it is substantially different. Low certainty: Our confidence in the effect estimate is limited: The true effect may be substantially different from the estimate of the effect. Very low certainty: We have very little confidence in the effect estimate: The true effect is likely to be substantially different from the estimate of effect.										

**Abbreviations**: CI: confidence interval; CIN2+: cervical intraepithelial neoplasia, grade 2 or worse; CIN2+: cervical intraepithelial neoplasia, grade 3 or worse; HPV: human papillomavirus; RR: risk ratio *Anticipated absolute effect compares two risks by calculating the difference between the risks of the intervention groups with the risk of the control group. ^a^Downgraded one level for risk of bias due to some concerns regarding deviations from intended intervention, missing data and selective reporting. ^b^Downgraded one level for inconsistency: one study had an effect estimate in the opposite direction compared to the other studies, leading to substantial heterogeneity (I^2^ = 79%). ^c^Downgraded one level for indirectness: three of the four studies reported on any oncogenic HPV types (i.e. excluding outcome testing positive for non‐oncogenic HPV or testing negative for HPV). ^d^Downgraded one level for imprecision due to wide confidence interval consistent with the possibility of benefit and the possibility of no effect/trivial effect. ^e^Downgraded one level for risk of bias due to some concerns regarding deviations from intended intervention and missing data. ^f^Downgraded two levels for imprecision due to wide confidence interval consistent with the possibility of benefit and the possibility of harm. ^g^Downgraded one level for imprecision due to wide confidence interval consistent with the possibility of harm and the possibility of no effect/trivial effect. ^h^Downgraded one level for risk of bias due to some concerns regarding missing data and selective reporting. ^i^Downgraded one level for risk of bias due to some concerns regarding randomisation and selective reporting.

**Summary of findings 3:** Summary of findings: Vaccine‐matched HPV type‐associated high‐grade VIN or VaIN* by population and by vaccine

**Population:** females of all ages **Interventions:** Cervarix, Gardasil, Gardasil‐9, Cecolin **Comparator:** injection control (saline placebo, adjuvant placebo, non‐HPV control vaccine), Cervarix, Gardasil **Outcome:** HPV‐associated high‐grade VIN or VaIN										
**Comparison**	**Direct evidence**						**Network meta‐analysis**			
	**N participants (studies)**	Anticipated absolute effect**		**Relative effect** **RR (95% CI)**	**Certainty of the evidence** **(GRADE)**		Anticipated absolute effect*		**Relative effect** **RR (95% CI)**	**Certainty of the evidence** **(GRADE)**
		**With control**	**With vaccine**				**With control**	**With vaccine**		
***Females 14 years or younger:*****no studies reported on this outcome for this population group**										
*****Females 15 to 25 years*****										
Cervarix vs injection control Follow‐up: 4 years	15,566 (1)	1 per 1000	0 per 1000 (0 to 1)	0.28 (0.06 to 1.37)	MODERATE^a^		3 per 1000	1 per 1000 (0 to 4)	0.28 (0.06 to 1.37)	MODERATE^a^
		1 fewer per 1000 (1 to 0 fewer)					2 fewer per 1000 (3 fewer to 1 more)			
Gardasil vs injection control Follow‐up: 4 years	17,925 (3)	5 per 1000	1 per 1000 (0 to 2)	0.21 (0.10 to 0.43)	MODERATE^b^		3 per 1000	1 per 1000 (0 to 1)	0.21 (0.10 to 0.45)	MODERATE^b^
		4 fewer per 1000 (3 to 4 fewer)					2 fewer per 1000 (1 to 2 fewer)			
Gardasil‐9 vs injection control Follow‐up: up to 6 years	No studies with direct comparison						3 per 1000	0 per 1000 (0 to1)	0.16 (0.05 to 0.51)	MODERATE^c^
							2 fewer per 1000 (1 to 3 fewer)			
Cecolin vs injection control	No studies with direct or indirect comparison									
Gardasil vs Cervarix Follow‐up: up to 6 years	No studies with direct comparison						0 per 1000	0 per 1000 (0 to1)	0.74 (0.13 to 4.17)	MODERATE^d^
							0 more per 1000 (0 to 1 more)			
Gardasil‐9 vs Cervarix Follow‐up: up to 6 years	No studies with direct comparison						0 per 1000	0 per 1000 (0 to 1)	0.58 (0.09 to 3.94)	MODERATE^a^
							0 more per 1000 (0 to 1 more)			
Gardasil‐9 vs Gardasil Follow‐up: 6 years	14,042 (1)	2 per 1000	2 per 1000 (1 to 3)	0.79 (0.36 to 1.73)	MODERATE^c^		1 per 1000	1 per 1000 (0 to 2)	0.79 (0.35 to 1.75)	MODERATE^c^
		0 fewer per 1000 (1 fewer to 1 more)					0 fewer per 1000 (1 fewer to 1 more)			
*****Females over 25 years*****										
Cervarix vs injection control	No studies with direct or indirect comparison									
Gardasil vs injection control Follow‐up: 4 years	3382 (1)	Not estimable, no events in control group		4.98 (0.24 to 103.58)	LOW^e^	No network meta‐analysis				
Gardasil‐9 vs injection control	No studies with direct or indirect comparison									
Cecolin vs injection control	No studies with direct or indirect comparison									
**GRADE Working Group grades of evidence** High certainty: We are very confident that the true effect lies close to that of the estimate of the effect. Moderate certainty: We are moderately confident in the effect estimate: The true effect is likely to be close to the estimate of the effect, but there is a possibility that it is substantially different. Low certainty: Our confidence in the effect estimate is limited: The true effect may be substantially different from the estimate of the effect. Very low certainty: We have very little confidence in the effect estimate: The true effect is likely to be substantially different from the estimate of effect.										

**Abbreviations**: CI: confidence interval; HPV: human papillomavirus; RR: risk ratio; VaIN: vaginal intraepithelial neoplasia; VIN: vulval intraepithelial neoplasia *Vaccine‐matched HPV‐type indicates positive for HPV 16 and/or 18 for Cervarix and Cecolin; HPV 6, 11, 16 and/or 18 for Gardasil; HPV 6, 11, 16, 18, 31, 33, 45, 52 and/or 58 for Gardasil‐9. High‐grade intraepithelial neoplasia refers to grade 2 or 3. **Anticipated absolute effect compares two risks by calculating the difference between the risks of the intervention groups with the risk of the control group. ^a^Downgraded one level for risk of bias due to some concerns regarding deviations from intended interventions and missing data. ^b^Downgraded one level for risk of bias due to some concerns regarding bias arising from the randomisation process and selection of the reported result. ^c^Downgraded one level for imprecision due to low number of events. ^d^Downgraded one level for risk of bias due to some concerns regarding bias arising from the randomisation process, deviations from intended interventions, missing data and selection of the reported result. ^e^Downgraded two levels for imprecision due to wide confidence interval consistent with the possibility of benefit and the possibility of harm, and a very low number of events.

**Summary of findings 4:** Summary of findings: High‐grade penile or anal intraepithelial neoplasia by population and by vaccine

**Population:** males of all ages **Interventions:** Cervarix, Gardasil, Gardasil‐9, Cecolin **Comparator:** injection control (saline placebo, adjuvant placebo, non‐HPV control vaccine), Cervarix, Gardasil **Outcomes:** histologically confirmed high‐grade PeIN or AIN, irrespective of HPV vaccine and associated with vaccine HPV‐types					
**Comparison**	**N participants (studies)**	Anticipated absolute effect*		**Relative effect** **RR (95% CI)**	**Certainty of the evidence** **(GRADE)**
		**With control**	**With vaccine**		
*****Males 14 years or younger:***no studies reported on this outcome for this population group**					
Males 15 to 25 years*					
Cervarix vs injection control	No studies with direct or indirect comparison				
***High‐grade AIN, irrespective of HPV type*** Gardasil vs injection control Follow‐up: 3 years	551**(1)	214 per 1000	160 per 1000 (113 to 229)	0.75 (0.53 to 1.07)	LOW^a,b^
		54 fewer per 1000 (101 fewer to 15 more)			
***High‐grade AIN, associated with vaccine HPV‐types*** Gardasil vs injection control Follow‐up: 3 years	551**(1)	141 per 1000	65 per 1000 (38 to 112)	0.46 (0.27 to 0.79)	LOW^b,c^
		76 fewer per 1000 (30 to 103 fewer)			
***High‐grade PeIN, irrespective of HPV type*** Gardasil vs injection control Follow‐up: 3 years	3880 (1)	2 per 1000	2 per 1000 (0 to 8)	1.00 (0.20 to 4.93)	LOW^d^
		0 fewer per 1000 (1 fewer to 6 more)			
***High‐grade PeIN, associated with vaccine HPV‐types*** Gardasil vs injection control Follow‐up: 3 years	3880 (1)	2 per 1000	2 per 1000 (0 to 8)	1.00 (0.20 to 4.93)	LOW^d^
		0 fewer per 1000 (1 fewer to 6 more)			
Gardasil‐9 vs injection control	No studies with direct or indirect comparison				
Cecolin vs injection control	No studies with direct or indirect comparison				
*****Males 25 years or older:***no studies reported on this outcome for this population group**					
**GRADE Working Group grades of evidence** High certainty: We are very confident that the true effect lies close to that of the estimate of the effect. Moderate certainty: We are moderately confident in the effect estimate: The true effect is likely to be close to the estimate of the effect, but there is a possibility that it is substantially different. Low certainty: Our confidence in the effect estimate is limited: The true effect may be substantially different from the estimate of the effect. Very low certainty: We have very little confidence in the effect estimate: The true effect is likely to be substantially different from the estimate of effect.					

**Abbreviations**: AIN: anal intraepithelial neoplasia; CI: confidence interval; HPV: human papillomavirus; PeIN: penile intraepithelial neoplasia; RR: risk ratio High‐grade intraepithelial neoplasia refers to grade 2 or 3. *Anticipated absolute effect compares two risks by calculating the difference between the risks of the intervention groups with the risk of the control group. **Measured and reported in a subgroup of men who have sex with men. ^a^Downgraded one level for imprecision due to few events and participants, leading to wide 95% CIs around the absolute effect. ^b^Downgraded one level for indirectness: measured and reported in a high‐risk subgroup (men who have sex with men). ^c^Downgraded one level for imprecision due to few events and participants. ^d^Downgraded two levels for imprecision due to very few events and few participants.

**Summary of findings 5:** Summary of findings: Treatment rates for HPV‐related pre‐invasive disease by population and by vaccine

**Population:** females and males of all ages **Interventions:** Cervarix, Gardasil, Gardasil‐9, Cecolin **Comparator:** injection control (saline placebo, adjuvant placebo, non‐HPV control vaccine), Cervarix, Gardasil **Outcome:** treatment rates for HPV‐related pre‐invasive disease					
**Comparison**	**N participants (studies)**	Anticipated absolute effect*		**Relative effect** **RR (95% CI)**	**Certainty of the evidence** **(GRADE)**
		**With control**	**With vaccine**		
*****Females 14 years or younger:***no studies reported on this outcome for this population group**					
Females 15 to 25 years**					
Cervarix vs injection control Follow‐up: 4 years	25,488 (2)	42 per 1000	32 per 1000 (25 to 41)	0.76 (0.59 to 0.98)	MODERATE^a,b^
		10 fewer per 1000 (1 to 17 fewer)			
Gardasil vs injection control	No direct or indirect evidence				
Gardasil‐9 vs injection control	No direct or indirect evidence				
Cecolin vs injection control	No direct or indirect evidence				
Gardasil‐9 vs Gardasil Follow‐up: 6 years	13,754 (1)	16 per 1000	17 per 1000 (13 to 22)	1.05 (0.81 to 1.37)	HIGH
		1 more per 1000 (3 fewer to 6 more)			
Females > 25 years***					
Cervarix vs injection control Follow‐up: 7 years	5468 (1)	40 per 1000	32 per 1000 (25 to 42)	0.80 (0.61 to 1.05)	MODERATE^c^
		8 fewer per 1000 (16 fewer to 2 more)			
Gardasil vs injection control Follow‐up: 4 years	3768 (1)	87 per 1000	79 per 1000 (64 to 98)	0.91 (0.74 to 1.13)	MODERATE^c^
		8 fewer per 1000 (23 fewer to 11 more)			
Gardasil‐9 vs injection control	No studies with direct or indirect comparison				
Cecolin vs injection control	No studies with direct or indirect comparison				
*****Males 14 years or younger:***no studies reported on this outcome for this population group**					
*****Males 15 to 25 years*****					
Cervarix vs injection control	No studies with direct or indirect comparison				
Gardasil vs injection control Follow‐up: 3 years	3880 (1)	66 per 1000	39 per 1000 (30 to 51)	0.59 (0.45 to 0.78)	HIGH
		27 fewer per 1000 (14 to 36 fewer)			
Gardasil‐9 vs injection control	No studies with direct or indirect comparison				
Cecolin vs injection control	No studies with direct or indirect comparison				
*****Males 25 years or older:***no studies reported on this outcome for this population group**					
**GRADE Working Group grades of evidence** High certainty: We are very confident that the true effect lies close to that of the estimate of the effect. Moderate certainty: We are moderately confident in the effect estimate: The true effect is likely to be close to the estimate of the effect, but there is a possibility that it is substantially different. Low certainty: Our confidence in the effect estimate is limited: The true effect may be substantially different from the estimate of the effect. Very low certainty: We have very little confidence in the effect estimate: The true effect is likely to be substantially different from the estimate of effect.					

**Abbreviations**: CI: confidence interval; HPV: human papillomavirus; NMA: network meta‐analysis; RR: risk ratio *Anticipated absolute effect compares two risks by calculating the difference between the risks of the intervention groups with the risk of the control group. **Direct evidence only; no NMA because there were only three disjointed studies. ***Direct evidence only; no NMA because there were only two studies. ^a^Downgraded one level for risk of bias due to some concerns regarding deviations from intended interventions, missing outcome data and selection of the reported result. ^b^There was unexplained statistical heterogeneity in this analysis (I^2^ = 75%); however, we did not downgrade for inconsistency since all estimates were in the same direction.  ^c^Downgraded one level for imprecision due to low number of participants/events.

**Summary of findings 6:** Summary of findings: Anogenital warts irrespective of HPV type by population and by vaccine

**Population:** females and males of all ages **Interventions:** Cervarix, Gardasil, Gardasil‐9, Cecolin **Comparator:** injection control (saline placebo, adjuvant placebo, non‐HPV control vaccine), Cervarix, Gardasil **Outcome:** anogenital warts									
**Comparison**	**N participants (studies)**	**Direct evidence**				**Network meta‐analysis**			
		Anticipated absolute effect*		**Relative effect** **RR (95% CI)**	**Certainty of the evidence** **(GRADE)**	Anticipated absolute effect*		**Relative effect** **RR (95% CI)**	**Certainty of the evidence** **(GRADE)**
		**With control**	**With vaccine**			**With control**	**With vaccine**		
*****Females 14 years or younger:***no studies reported on this outcome for this population group**									
*****Females 15 to 25 years*****									
Cervarix vs injection control	No studies with direct or indirect comparison								
Gardasil vs injection control Follow‐up: 4 years	17,391 (2)	40 per 1000	15 per 1000 (13 to 19)	0.38 (0.31 to 0.47)	HIGH	41 per 1000	16 per 1000 (13 to 19)	0.38 (0.31 to 0.47)	HIGH
		25 per 1000 (21 to 28 fewer)				25 fewer per 1000 (22 to 28 fewer)			
Gardasil‐9 vs injection control	No studies with direct comparison					41 per 1000	14 per 1000 (9 to 21)	0.34 (0.23 to 0.51)	HIGH
						27 fewer per 1000 (20 to 32 fewer)			
Cecolin vs injection control	No studies with direct or indirect comparison								
Gardasil‐9 vs Gardasil Follow‐up: 6 years	14,050 (1)	12 per 1000	11 per 1000 (8 to 15)	0.90 (0.66 to 1.22)	HIGH	15 per 1000	14 per 1000 (10 to 19)	0.90 (0.65 to 1.23)	HIGH
		1 fewer per 1000 (4 fewer to 3 more)				2 fewer per 1000 (5 fewer to 4 more)			
*****Females 25 years or older:***no studies reported on this outcome for this population group**									
*****Males 14 years or younger:***no studies reported on this outcome for this population group**									
*****Males 15 to 25 years*****									
Cervarix vs injection control	No studies with direct or indirect comparison								
Gardasil vs injection control Follow‐up: 36 months	3880 (1)	44 per 1000	17 per 1000 (11 to 25)	0.39 (0.26 to 0.58)	HIGH	No studies with indirect comparison			
		27 fewer per 1000 (18 to 32 fewer)							
Gardasil‐9 vs injection control	No studies with direct or indirect comparison								
Cecolin vs injection control	No studies with direct or indirect comparison								
*****Males 25 years or older:***no studies reported on this outcome for this population group**									
**GRADE Working Group grades of evidence** High certainty: We are very confident that the true effect lies close to that of the estimate of the effect. Moderate certainty: We are moderately confident in the effect estimate: The true effect is likely to be close to the estimate of the effect, but there is a possibility that it is substantially different. Low certainty: Our confidence in the effect estimate is limited: The true effect may be substantially different from the estimate of the effect. Very low certainty: We have very little confidence in the effect estimate: The true effect is likely to be substantially different from the estimate of effect.									

**Abbreviations**: CI: confidence interval; HPV: human papillomavirus; RR: risk ratio *Anticipated absolute effect compares two risks by calculating the difference between the risks of the intervention groups with the risk of the control group.

**Summary of findings 7:** Summary of findings: Serious adverse events by population and by vaccine

**Population:** females and males of all ages **Interventions:** Cervarix, Gardasil, Gardasil‐9, Cecolin **Comparator:** injection control (saline placebo, adjuvant placebo, non‐HPV control vaccine), Cervarix, Gardasil **Outcome:** serious adverse events					
**Comparison**	**N participants (studies)**	**Direct evidence**			
		Anticipated absolute effect*		**Relative effect** **RR (95% CI)**	**Certainty of the evidence** **(GRADE)**
		**With control**	**With vaccine**		
*****Females < 15 years*****					
Cervarix vs injection control Follow‐up: 7 to 12 months	4838 (6)	15 per 1000	15 per 1000 (9 to 24)	1.00 (0.62 to 1.60)	HIGH
		0 fewer per 1000 (6 fewer to 9 more)			
Gardasil vs injection control Follow‐up: 6 to 7 months	205 (2)	Not estimable, no events in control group		1.57 (0.08 to 31.59)	LOW^b^
Gardasil‐9 vs injection control	No direct evidence				
Cecolin vs injection control	No direct evidence				
Gardasil vs Cervarix Follow‐up: 15 months to 3 years	1273 (2)	51 per 1000	30 per 1000 (17 to 52)	0.59 (0.34 to 1.02)	MODERATE^c^
		21 fewer per 1000 (33 fewer to 1 more)			
Gardasil‐9 vs Cervarix Follow‐up: 2 years	930 (1)	39 per 1000	51 per 1000 (28 to 94)	1.33 (0.73 to 2.42)	MODERATE^d^
		13 more per 1000 (10 fewer to 55 more)			
Gardasil‐9 vs Gardasil Follow‐up: 7 months	599 (1)	7 per 1000	3 per 1000 (0 to 37)	0.50 (0.05 to 5.50)	LOW^e^
		3 fewer per 1000 (6 fewer to 30 more)			
*****Females 15 to 25 years*****					
Cervarix vs injection control Follow‐up: up to 6 years	37,802 (12)	102 per 1000	102 per 1000 (96 to 108)	1.00 (0.94 to 1.06)	HIGH
		0 fewer per 1000 (6 fewer to 6 more)			
Gardasil vs injection control Follow‐up: up to 4 years	19,467 (6)	17 per 1000	14 per 1000 (10 to 18)	0.80 (0.61 to 1.04)	HIGH
		3 fewer per 1000 (7 fewer to 1 more)			
Gardasil‐9 vs injection control Follow‐up: 18 months	1515 (1)	52 per 1000	45 per 1000 (29 to 70)	0.87 (0.56 to 1.36)	LOW^a^
		7 fewer per 1000 (23 fewer to 19 more)			
Cecolin vs injection control Follow‐up: 7 months	1594 (1)	8 per 1000	9 per 1000 (3 to 33)	1.22 (0.34 to 4.34)	LOW^g^
		2 more per 1000 (5 fewer to 25 more)			
Gardasil vs Cervarix Follow‐up: 7 months	62 (1)	Not estimable, no events		Not estimable, no events	VERY LOW^h,i^
Gardasil‐9 vs Cervarix Follow‐up: 18 months	1518 (1)	51 per 1000	45 per 1000 (29 to 70)	0.87 (0.56 to 1.37)	LOW^j^
		7 fewer per 1000 (23 fewer to 19 more)			
Gardasil‐9 vs Gardasil Follow‐up: 6 years	14,149 (1)	26 per 1000	33 per 1000 (27 to 40)	1.27 (1.05 to 1.53)	HIGH
		7 more per 1000 (1 to 14 more)			
Cecolin vs Cervarix	No direct evidence				
Cecolin vs Gardasil	No direct evidence				
Cecolin vs Gardasil‐9	No direct evidence				
*****Females > 25 years*****					
Cervarix vs injection control Follow‐up: up to 4 years	6959 (2)	77 per 1000	83 per 1000 (71 to 98)	1.07 (0.92 to 1.26)	MODERATE^k^
		5 more per 1000 (6 fewer to 20 more)			
Gardasil vs injection control Follow‐up: 7.5 years	6775 (2)	17 per 1000	15 per 1000 (11 to 22)	0.88 (0.61 to 1.27)	HIGH
		2 fewer per 1000 (7 fewer to 5 more)			
Gardasil‐9 vs injection control	No direct evidence				
Cecolin vs injection control Follow‐up: 3.5 years	7072 (1)	61 per 1000	60 per 1000 (51 to 72)	0.99 (0.83 to 1.19)	MODERATE^k^
		1 fewer per 1000 (10 fewer to 12 more)			
Gardasil vs Cervarix Follow‐up: 5 years	1106 (1)	80 per 1000	67 per 1000 (44 to 102)	0.84 (0.55 to 1.28)	LOW^j^
		13 fewer per 1000 (36 fewer to 22 more)			
Cecolin vs Cervarix	No direct evidence				
Cecolin vs Gardasil	No direct evidence				
***Males < 15 years*****: no studies reported on this outcome for this population group.**					
***Males 15 to 25 years***					
Cervarix vs injection control Follow‐up: 12 months	270 (1)	11 per 1000	17 per 1000 (2 to 157)	1.48 (0.16 to 13.98)	LOW^g^
		5 more per 1000 (9 fewer to 146 more)			
Gardasil vs injection control Follow‐up: up to 57 months	5008 (2)	5 per 1000	3 per 1000 (1 to 8)	0.69 (0.29 to 1.65)	MODERATE^l^
		1 fewer per 1000 (3 fewer to 3 more)			
Gardasil‐9 vs injection control	No direct evidence				
Cecolin vs injection control	No direct evidence				
Gardasil vs Cervarix	No direct evidence				
Gardasil‐9 vs Cervarix	No direct evidence				
Gardasil‐9 vs Gardasil Follow‐up: 7 months	496 (1)	24 per 1000	2 per 1000 (0 to 33	0.08 (0.00 to 1.36)	LOW^n^
		22 fewer per 1000 (24 fewer to 9 more)			
***Males > 25 years*****: no studies reported on this outcome for this population group**					
**GRADE Working Group grades of evidence** High certainty: We are very confident that the true effect lies close to that of the estimate of the effect. Moderate certainty: We are moderately confident in the effect estimate: The true effect is likely to be close to the estimate of the effect, but there is a possibility that it is substantially different. Low certainty: Our confidence in the effect estimate is limited: The true effect may be substantially different from the estimate of the effect. Very low certainty: We have very little confidence in the effect estimate: The true effect is likely to be substantially different from the estimate of effect.					

**Abbreviations**: CI: confidence interval; HPV: human papillomavirus; RR: risk ratio *Anticipated absolute effect compares two risks by calculating the difference between the risks of the intervention groups with the risk of the control group. ^a^Downgraded two levels for imprecision due to wide confidence interval consistent with the possibility of benefit and the possibility of harm. ^b^Downgraded two levels for imprecision due to wide confidence interval consistent with the possibility of benefit and the possibility of harm, and very low number of participants/events. ^c^Downgraded one level for imprecision due to wide confidence interval consistent with the possibility of benefit for Gardasil and the possibility of no difference/trivial effect. ^d^Downgraded one level for imprecision due to wide confidence interval consistent with the possibility of no difference/trivial effect and the possibility of benefit with Cervarix. ^e^Downgraded two levels for imprecision due to wide confidence interval consistent with the possibility of no effect/trivial effect and the possibility of benefit with Gardasil and low number of participants/events. ^f^Downgraded one level for imprecision due to wide confidence interval consistent with the possibility of benefit and the possibility of no effect/trivial effect. ^g^Downgraded two levels for imprecision due to wide confidence interval consistent with the possibility of no effect/trivial effect and the possibility of harm, and low number of participants/events. ^h^Downgraded one level for risk of bias due to some concerns with bias arising from the randomisation process and missing outcome data. ^i^Downgraded two levels for imprecision due to no events in either group. ^j^Downgraded two levels for imprecision due to wide confidence interval consistent with the possibility of benefit with either vaccine. ^k^Downgraded one level for imprecision due to wide confidence interval consistent with the possibility of no effect/trivial effect and the possibility of harm. ^l^Downgraded one level for imprecision due to low number of events.  ^m^Downgraded one level for imprecision due to wide confidence interval consistent with the possibility of benefit with Gardasil‐9 and the possibility of no difference/trivial effect. ^n^Downgraded two levels for imprecision due to wide confidence interval consistent with the possibility of benefit with Gardasil‐9 and the possibility of no effect/trivial effect and low number of participants/events. ^o^Downgraded one level for imprecision due to low number of participants/events.

**Summary of findings 8:** Summary of findings: Serious adverse events by population, vaccine and dose

**Population:** females and males of all ages **Interventions:** Cervarix, Gardasil, Gardasil‐9, Cecolin **Comparator:** injection control (saline placebo, adjuvant placebo, non‐HPV control vaccine), Cervarix, Gardasil **Outcome:** serious adverse events					
**Comparison**	**N participants (studies)**	Anticipated absolute effect*		**Relative effect** **RR (95% CI)**	**Certainty of the evidence** **(GRADE)**
		**With more doses**	**With fewer doses**		
**Females < 15 years**					
2 vs 3 doses Cervarix Follow‐up: 2 years	310 (1)	39 per 1000	26 per 1000 (7 to 90)	0.67 (0.19 to 2.32)	LOW^a^
		13 fewer per 1000 (31 fewer to 51 more)			
1 vs 2 doses Cervarix Follow‐up: 2 years	310 (1)	26 per 1000	52 per 1000 (16 to 168)	2.00 (0.61 to 6.50)	LOW^b^
		26 more per 1000 (10 fewer to 142 more)			
1 vs 3 doses Cervarix Follow‐up: 2 years	310 (1)	39 per 1000	51 per 1000 (18 to 145)	1.33 (0.47 to 3.75)	LOW^a^
		13 more per 1000 (21 fewer to 106 more)			
2 vs 3 doses Gardasil Follow‐up: 3 years	869 (2)	23 per 1000	18 per 1000 (8 to 39)	0.79 (0.36 to 1.171)	LOW^a^
		5 fewer per 1000 (15 fewer to 16 more)			
2 vs 3 doses Gardasil‐9 Follow‐up: 3 years	904 (2)	31 per 1000	31 per 1000 (15 to 64)	1.01 (0.49 to 2.09)	LOW^a^
		0 more per 1000 (16 fewer to 34 more)			
1 vs 2 doses Gardasil‐9 Follow‐up: 2 years	310 (1)	52 per 1000	52 per 1000 (20 to 134)	1.00 (0.39 to 2.60)	LOW^a^
		0 more per 1000 (31 fewer to 83 more)			
1 vs 3 doses Gardasil‐9 Follow‐up: 2 years	310 (1)	52 per 1000	52 per 1000 (20 to 134)	1.00 (0.39 to 2.60)	LOW^a^
		0 more per 1000 (31 fewer to 83 more)			
2 vs 3 doses Cecolin Follow‐up: 7 months	310 (1)	7 per 1000	1 per 1000 (0 to 28)	0.20 (0.01 to 4.19)	VERY LOW^b,c^
		5 fewer per 1000 (7 fewer to 21 more)			
**Females 15 to 25 years**					
2 vs 3 doses Cervarix Follow‐up: 5 years	960 (1)	63 per 1000	68 per 1000 (39 to 119)	1.08 (0.62 to 1.89)	LOW^a^
		5 more per 1000 (24 fewer to 56 more)			
**Females > 25 years: no studies reported on different dose schedules for this population group**					
**Males: no studies reported on serious adverse events for different dose schedules for this population group**					
**GRADE Working Group grades of evidence** High certainty: We are very confident that the true effect lies close to that of the estimate of the effect. Moderate certainty: We are moderately confident in the effect estimate: The true effect is likely to be close to the estimate of the effect, but there is a possibility that it is substantially different. Low certainty: Our confidence in the effect estimate is limited: The true effect may be substantially different from the estimate of the effect. Very low certainty: We have very little confidence in the effect estimate: The true effect is likely to be substantially different from the estimate of effect.					

**Abbreviations**: CI: confidence interval; HPV: human papillomavirus; RR: risk ratio *Anticipated absolute effect compares two risks by calculating the difference between the risks of the intervention groups with the risk of the control group. ^a^Downgraded two levels for imprecision due to wide confidence interval consistent with the possibility of benefit with fewer doses and the possibility of benefit with more doses, and low number of participants/events. ^b^Downgraded two levels for imprecision due to wide confidence interval consistent with the possibility of no or trivial effect and the possibility of benefit with more doses, and low number of participants/events. ^c^Downgraded one level for risk of bias due to potential bias arising from the randomisation process and measurement of the outcome.

## Background

### Description of the condition

Cervical cancer is the fourth most common cancer and the fourth leading cause of death from cancer amongst females worldwide, with an estimated 570,000 new cases and 311,000 deaths in 2018 (Bray 2018). Cervical cancer is a common cancer in young women and people with a uterine cervix, particularly in the 25‐ to 45‐year age group (Bray 2018). The risk of developing cervical cancer by the age of 65 ranges from 0.8% in developed countries to 1.5% in developing countries and more than 85% of all cervical cancer deaths occur in low‐ and middle‐income countries (LMIC) (Bray 2018). The large geographical variation in cervical cancer rates and survival correlates with the availability of primary and secondary prevention strategies, as well as the prevalence of high‐risk human papillomavirus (hrHPV) infection. However, even in countries such as the UK, with highly organised, regulated and effective cervical screening programmes, cervical cancer in females aged 25 to 49 is the fourth‐highest cause of cancer death (Cancer Research UK 2021a). In England, 4.63 million women are invited for cervical screening in a year (2019 to 2020), in order to identify and treat those at higher risk of cervical cancer (NHS Digital 2020a). Of these, nearly 100,000 require further investigation with colposcopy (direct visualisation of the cervix with a microscope) to determine whether treatment is needed for cervical intra‐epithelial neoplasia (CIN), a precursor lesion, to prevent cervical cancer (NHS Digital 2020b). This can cause anxiety and distress for many people. Furthermore, treatment for CIN, although relatively minor and straightforward in most cases, may put some people at higher risk of premature birth, thereby having long‐term knock‐on effects of preventative treatment (Kyrgiou 2017).

Human papillomavirus (HPV) is the most common viral infection of the reproductive tract (WHO 2017). Persistent infection with hrHPV is necessary, but not sufficient, to develop cervical cancer. The majority of people are exposed to hrHPV and, although most HPV infections resolve spontaneously (Insinga 2011), persistent infections can lead to pre‐cancerous lesions (e.g. cervical and vulval intraepithelial neoplasia (CIN and VIN)), and cancer of the cervix, vagina, vulva, anus, penis, and head and neck. In 2012, HPV‐related cancers accounted for an estimated 4.5% of all cancers worldwide (De Martel 2017). Of these estimated 636,000 HPV‐related cancers, 530,000 were cervical cancer, 35,000 anal cancer, 8500 vulval cancer, 13,000 penile cancer and 37,000 head and neck cancers (De Martel 2017). A recent review article highlights the role of hrHPV in the development of a variety of cancers in the USA (Markowitz 2023).

Vulval cancer is a rare disease, with 45,240 new vulval cancers recorded globally in 2020, and an age‐standardised incidence rate of 0.85/100,000 females (Ferlay 2020d). Incidence rates are highest in Western Europe (2.4 per 100,000), although age‐standardised mortality is lower (0.49/100,000) than in Eastern (0.89/100,000) and Middle Africa (0.85/100,000). The risk of vulval cancer increases with age: in the UK the rates are highest in those aged over 90 years (Morrison 2024). Around 70% of vulval cancers in the USA are thought to be HPV‐mediated (Markowitz 2023), with the rest occurring on a background of vulval dermatoses, such as lichen sclerosus and lichen planus (Morrison 2024). In the UK, the incidence of vulval cancer has increased by around a sixth (17%) since the early 1990s (Lai 2014), with an additional projected 5% increase over the next 15 to 20 years (Cancer Research UK 2025e). This increased incidence in younger cohorts (< 60 years), due to an increase in HPV‐related VIN (Lai 2014; Kang 2017), is seen across many countries, where rates have nearly doubled in the past decade (Buttmann‐Schweiger 2015; Kang 2017; Pils 2017). Vaginal cancers are even rarer, with a global incidence of 18,800 in 2020 (Ferlay 2020a), and 75% to 80% are associated with hrHPV infection (Daling 2002; Markowitz 2023). Vaginal cancer is a disease of elderly females, with peak incidence in the 85‐ to 89‐year age cohort (Cancer Research UK 2025d).

Penile cancer is also rare, with 36,068 new cases and 13,211 deaths in 2020 (Ferlay 2020b; Fu 2022). It predominately affects older males (Mannweiler 2013), the risk increasing with age, and with the highest incidences in the ≥ 90‐year age group in the UK (Cancer Research UK 2025a). Similar to vulval cancer, the majority are squamous cell cancers and arise on a background of persistent infections with hrHPV, or independent of HPV, secondary to dermatoses, e.g. lichen sclerosus (LS) and lichen planus (LP). In a case series, 51 of 123 penile squamous cell cancers were negative for hrHPV DNA, whereas the rest (59%) were hrHPV DNA positive (mostly HPV 16) (Mannweiler 2013). Incidence rates of penile cancers vary worldwide, with 10% of cancers in men due to penile cancer in South America, South Africa and South Asia (Bleeker 2009).

Anal cancer, although rare, is more common in females than males, although in males it is more common in men who have sex with men (Mignozzi 2024). In 2020, there were 54,196 cases reported globally, with just under 22,000 deaths (Ferlay 2020c). The median age of diagnosis is 63 years in the USA (SEER 2024), and the 60‐ to 64‐year age group in females and 65‐ to 70‐year age group in males in the UK, although the peak incidence rates are in the 75 to 79 age group for females and 80 to 84 age group for males (Cancer Research UK 2025b). Almost 90% are associated with hrHPV infection (Daling 2004; Goodman 2010). In one series, HPV 16 was the most frequent hrHPV type (73% of all tumours), followed by HPV 18 (6.9%) (Daling 2004); smoking and receptive anal intercourse were major risk factors. Incidence rates are increasing, with an almost 3% per year increase seen in the USA between 2001–2015, due to HPV‐related disease in younger age groups (Cancer Research UK 2025b; Deshmukh 2020).

Globally, head and neck squamous cell cancers account for 650,000 new cases of cancer annually (Sabatini 2020; Sung 2021). Smoking and alcohol were traditionally thought to be the major risk factors, although increasing evidence points to hrHPV as a cause, with the relative percentage of hrHPV‐positive cancer varying between studies and location of tumour (Sabatini 2020), from 3.9% of oral cavity squamous cell cancers, to over 60% of oropharyngeal squamous cell cancers (Leemans 2018). The prevalence of HPV‐associated oropharyngeal cancer has increased over time; in the USA between 1995 and 2012, hrPHV oropharyngeal cancers increased in males (from 36% to 72%; P < 0.001) and in females (29% to 77%; P = 0.005) (D’Souza 2017). In the UK, incidence rates rise from around age 35 to 39, with the highest rates in the 85 to 89 age group for females and the 65 to 69 age group for males (Cancer Research UK 2025c).

Anogenital warts are caused by non‐oncogenic HPV subtypes, with HPV 6 and 11 responsible for 90% (Hawkins 2013). Anogenital warts are highly transmissible and difficult to eradicate, with high recurrence rates. The cost of treatment of anogenital warts in England in 2008 was estimated to be GBP 16.8 million, contributing to 6.6 days of healthy life lost per episode (Desai 2011; Woodhall 2011), and USD 220 million in the USA in 2004 (Insinga 2005). A systematic review found that annual incidence rates of new and recurrent anogenital warts, from clinical studies, vary from 160 to 289 per 100,000 (Patel 2013). Incidence is higher in people who are immunocompromised, such as following organ transplantation and HIV infection, and in men who have sex with men (MSM), with 11.6% of MSM reporting anogenital warts in a UK‐based study (Sonnenberg 2019). Many studies included in the systematic review came from high‐income countries (Patel 2013). However, in one study from Nigeria, the incidence of anogenital warts was 1% in HIV‐negative women and 5% in HIV‐positive women, demonstrating a significant health burden, especially in LMICs, which can have a profound effect upon quality of life (Dareng 2019).

With the advent of immunisation and screening programmes in developed countries, the majority of invasive cervical cancers could be prevented (Cancer Research UK 2021b). In 2018, The World Health Organization (WHO) Director‐General made a global call for the elimination of cervical cancer (Adhanom‐Ghebreyesus 2018). However, in the absence of organised screening, many people present with symptoms and locally advanced cervical cancer at diagnosis (WHO 2018). Sadly, even in countries with well‐organised, freely available screening programmes, screening cannot prevent all cervical cancers, and is not widely accessible globally. Cervical cancer, therefore, remains a significant global disease. Furthermore, ~20% of HPV‐related cancers do not have effective screening methods (WHO 2018). The three pillars of the WHO cervical cancer elimination strategy are:

- vaccination: 90% of girls are fully vaccinated with the HPV vaccine by the age of 15;
- screening: 70% of women screened using a high‐performance test by the age of 35, and again by the age of 45; and
- treatment: 90% of women with pre‐cancer are treated and 90% of women with invasive cancer managed.

The introduction of primary testing for hrHPV, compared to cervical cytology, improves the sensitivity of screening, albeit at the cost of increased referrals to colposcopy (Koliopoulos 2017). This leads to an increased rate of detection of CIN and is likely to reduce the rate of cervical cancer within a population over time.

### Description of the intervention

As per the WHO cervical cancer elimination strategy above, HPV vaccination is a primary prevention strategy, and sits alongside effective secondary prevention methods, such as screening to identify and then treatment of high‐grade CIN.

HPV vaccines were first licenced in 2006, and by 2016, 55% of high‐income (HIC) and upper‐middle‐income (UMIC) countries had introduced vaccination programmes, compared to just 14% of lower‐middle‐income (LMIC) and lower income (LIC) countries, where disease burden of cervical cancer is higher, according to World Bank figures (Gallagher 2018; LaMontagne 2017).

Uptake of HPV vaccination varies widely between countries, ranging from 8% to 98% (83.9% in the UK) (Gallagher 2018). Reasons for this variation include organisation of immunisation programmes, resistance from healthcare providers, adverse media coverage and concerns about safety (Gallagher 2018).

Four prophylactic HPV vaccines are available and have been pre‐qualified by WHO (see Table). Each vaccine is directed against two or more hrHPV genotypes. All four vaccines contain L1 proteins of HPV genotypes 16 and 18 (Qiao 2020; WHO 2017), because these cause about 70% of cervical cancers globally. In addition to the pre‐qualified vaccines, as of December 2021, there are two vaccines in stage 2 to 3 development, one bivalent vaccine manufactured by Walvax in China, and a quadrivalent vaccine manufactured by the Serum Institute of India (LaMontagne 2017). Two vaccines also contain L1 proteins for HPV 6 and 11 (see Table), which are responsible for 90% of anogenital warts (Hawkins 2013).

**1:** Characteristics of WHO pre‐qualified prophylactic HPV vaccines

	**Cervarix**	**Gardasil**	**Gardasil 9**	**Cecolin**
**Manufacturer**	GlaxoSmithKline (GSK, Rixensart, Belgium)	Merck, Sharp & Dome (Merck & Co, Whitehouse Station, NJ, USA)	Merck, Sharp & Dome (Merck & Co, Whitehouse Station, NJ, USA)	Xiamen Innovax Biotech Co. Ltd. (Xiamen, Fujian province, China)
**Antigens**	Bivalent: L1 VLPs of HPV16 (20 μg) and HPV18 (20 μg)	Quadrivalent: L1 VLPs of HPV 6 (20 μg), HPV 11 (40 μg), HPV 16 (40 μg) and HPV 18 (20 μg)	Nonavalent: L1 VLPs of HPV 6 (30 μg), HPV 11 (40 μg), HPV 16 (60 μg), HPV 18 (40 μg), HPV 31 (20 μg), HPV 33 (20 μg), HPV 45 (20 μg), HPV 52 (20 μg) and HPV 58 (20 μg)	Bivalent: L1 VLPs of HPV 16 (40 μg) and HPV 18 (20 μg)
**Vaccination schedule**	3 doses: at day 1, month 1 and month 6	3 doses: at day 1, month 2 and month 6	3 doses: at day 1, month 2 and month 6	2 doses: at day 1 and month 6
**Adjuvant**	AS04: 500 μg aluminium hydroxide, 50 μg 3‐deacylated monophosphoryl lipid A (MPL)	225 μg amorphous aluminium hydroxyl‐phosphate sulphate	500 μg amorphous aluminium hydroxyl‐phosphate sulphate	208 μg aluminium adjuvant
**Trade name**	Cervarix	Gardasil, Silgard	Gardasil 9	Cecolin
**Produced by recombinant technology using**	Baculovirus in *Trichoplusia* in insect cells	*Saccharomyces cerevisae* (Baker’s yeast)	*Saccharomyces cerevisae* (Baker’s yeast)	*Escherichia coli*

**Abbreviations**: HPV: human papillomavirus; MPL: monophosphoryl lipid; VLP: virus‐like particle.

### How the intervention might work

HPV L1 coat proteins self‐assemble into virus‐like particles (VLP), empty virus particles (capsids), containing no virus DNA (Kirnbauer 1992), which cannot cause an active infection. They work as prophylactic vaccines, which means they prevent an initial infection by HPV, in turn preventing the development of intraepithelial lesions caused by HPV genotypes that are present in the vaccine (Stanley 2006). HPV vaccines are therefore less effective in those already exposed to HPV (Arbyn 2018), hence why they are offered to adolescents, aiming for immunity prior to onset of sexual activity.

The virus‐like particles in the vaccines produce very high levels of antibodies in blood samples. The International Agency for Research on Cancer regards persistent HPV infection with HPV types 16 and 18 as an accurate surrogate marker for the development of pre‐cancerous lesions of the cervix and anus (IARC 2014). Persistent infection with hrHPV is the main cause of cervical cancer (Bosch 2002; Jaisamrarn 2013; Munoz 1996), with a well‐recognised progression from persistent HPV infection to the development of cervical intraepithelial neoplasia (CIN), although the majority of infections are cleared spontaneously and do not cause persistent infection (Insinga 2011). However, left untreated, almost one in three of those with high‐grade CIN (CIN3) will go on to develop cancer over 8 to 15 years (Campbell 1989; McIndoe 1984). It was therefore assumed that prevention of pre‐cancerous lesions would also be shown to prevent cancer when sufficient follow‐up time has accrued in post‐licensure studies. Less is known about the prognostic value of persistent HPV infection in the development of vaginal, vulval and oropharyngeal cancers (IARC 2014).

Initial vaccine schedules used a three‐dose regimen. However, data from randomised controlled trials (RCTs) and post‐licensure studies demonstrated good effectiveness for those who had not received all three doses (D'Addario 2017; Kreimer 2011; Kreimer 2015; Markowitz 2018; Sankaranarayanan 2016). Subsequent studies have used a two‐dose and, more recently, a single‐dose strategy. Simplified HPV vaccination schedules, with fewer doses, should allow more people to receive the vaccine, especially in resource‐poor settings (Nasser 2023). Pre‐adolescents and adolescents (age 9 to 15 years) produce stronger antibody responses to VLP HPV vaccines than older adolescents and adults (Block 2006; Dobson 2013), even after a single dose (Sankaranarayanan 2016). The likely mechanism for this is more fully explained in a previous Cochrane review (Bergman 2019).

Unlike cervical cancer, which is common globally and affects young women, other HPV‐related cancers affect much older age cohorts, suggesting a much longer natural history from initial infection with hrHPV to development of HPV‐dependent cancers. Effects of primary prevention with HPV vaccination are therefore likely to take many decades to become apparent in other HPV‐dependent cancers, especially as these cancers, again unlike cervical cancer, currently lack effective screening strategies to detect and treat pre‐cancer.

### Why it is important to do this review

Prevention or early detection of cancer is a major priority for health care, especially within the UK, where survival rates for many cancers lag behind European counterparts, largely due to late detection (De Angelis 2014).

In cervical cancer we are fortunate, as the main focus is on prevention, since, unlike many cancers, it can be prevented or detected at a pre‐invasive stage. A major priority for healthcare providers, including the WHO and Cochrane, is to update and combine data from two separate Cochrane reviews on HPV vaccination (Arbyn 2018; Bergman 2019), including non‐published data, and draw these together as a network meta‐analysis (NMA) to compare different vaccines and vaccination schedules.

The recommended dosing schedules for HPV vaccines changed from originally being three doses (as assessed in the placebo‐controlled trials) to two doses (WHO 2017). More recent results of RCTs on single‐dose vaccination led the WHO and the UK‐based Joint Committee on Vaccination and Immunisation (JCVI) to recommend a one‐dose schedule for the routine adolescent programme (FDA 2022; Henschke 2022; JCVI 2022; WHO 2022). The aim of this NMA is to compare these dose schedules, as well as the different types of HPV vaccine in terms of benefits and harms. This will aid the WHO and other decision‐makers in recommending vaccine schedules with fewer doses, as this will have implications for screening intervals and change the cost‐effectiveness of immunisation and screening programmes. HPV vaccination, especially in countries where screening programmes are currently unaffordable, has the potential to be transformative.

Evaluating the longer‐term harms and benefits of HPV vaccination is extremely important, especially in the face of community concerns about these issues, which can fuel vaccine hesitancy (Karafillakis 2019; Wong 2020). Health scares about adverse events can be catastrophic to a vaccination programme. For example, in Denmark and Ireland, community scares saw vaccination rates temporarily drop from over 80% to around 50% (Corcoran 2018; Suppli 2018). In Japan, a scare also resulted in a pause in government recommendation of vaccination (Ujiie 2022).

With the global reach of social media, dissemination of information regarding adverse effects of vaccination can be extremely pervasive. It is therefore extremely important to evaluate these outcomes, some of which take more time to become apparent, more fully, to provide reliable data to young people, parents, clinicians, policymakers and others when they are making choices about vaccination.

The previous Cochrane intervention reviews only compared head‐to‐head studies, whereas a NMA approach explores the benefits and harms of different vaccines and dosing schedules, even where these have not been directly compared. This NMA also includes clinical study report data, allaying this criticism of the previous review (Jørgensen 2018c). Furthermore, an update of the evidence at this stage is timely, as there is potential for inclusion of longer‐term follow‐up data from RCTs. Those vaccinated as adolescents have had 10 to 20 years since vaccination, allowing evaluation of the impact on cervical cancer outcomes, as seen in population‐level studies (Falcaro 2021; Lei 2020), which would allow evaluation of the use of surrogate outcomes in clinical trials (IARC 2014). The full impact of HPV vaccination on cancer incidence will not be known for many years, since the natural history of anal, vulval, penile, and head and neck cancers, caused by hrHPV, is much longer.

A comprehensive examination of rare risks and a better understanding of the longer‐term benefits of HPV vaccination, such as effects on cancer rates, preterm birth rates and reduced complications due to falling need for treatment of CIN, require large datasets from population‐level studies. We have evaluated these in a parallel Cochrane review based on non‐RCT data (Henschke 2025). It is hoped that these reviews will better inform the public debate about the benefits and harms of HPV vaccination and allow better decision‐making at an individual level.

## Objectives

To evaluate the safety and efficacy of HPV vaccines, in females and males, to prevent cervical cancer and other HPV‐related diseases, in standard (pairwise) and network meta‐analysis (NMA) of randomised controlled trials.

## Methods

### Criteria for considering studies for this review

#### Types of studies

Randomised controlled trials (RCTs) and quasi‐RCTs were included. Quasi‐RCTs are trials that intend to produce similar groups but are not truly random due to using randomisation sequences where allocation can be predicted based on, for example, order of participant recruitment or date of birth. If studies maintained randomised groups (i.e. participants were not allocated or exposed to other interventions) at longer‐term follow‐up, we included those time points in this review. If they did not, they were included in the partner review on population effects (Henschke 2025).

#### Types of participants

We considered studies on females and males of all ages for inclusion in the review. Results were stratified by age groups as follows: ≤ 14 years, 15 to 25 years, > 25 years. This division is in line with most studies (in over 15s it is more ethical to do cervical testing than in younger girls) and in line with catch‐up programmes.

We aimed to evaluate the effect of HPV vaccination on the general population. People who are immunocompromised, such as people living with HIV, are a particularly important risk group for HPV‐related disease. Nevertheless, we decided to exclude studies assessing only this group because the development of immunity following vaccination, waning of immunity after vaccination and the development of HPV‐related disease may differ in immunocompromised compared with immunocompetent people (Bergman 2019; Lacey 2019; Zhan 2019). We included studies that enroled pregnant women, although pregnant women may also be considered immunosuppressed.

#### Types of interventions

Primary prophylactic administration of HPV vaccines pre‐qualified by the WHO (WHO 2021), including Cervarix (bivalent, GlaxoSmithKline), Gardasil (quadrivalent, Merck), Gardasil‐9 (nonavalent, Merck) or Cecolin (bivalent, Innovax) HPV vaccines (see Table). We excluded studies assessing non‐prophylactic and secondary prevention (i.e. used to prevent recurrence in those treated for HPV‐related disease) use of HPV vaccines.

Specifically, we investigated the safety and efficacy of:

- vaccination with one of the pre‐qualified HPV vaccines compared with saline placebo, adjuvant placebo (aluminium hydroxide or another aluminium compound), no intervention or a non‐HPV control vaccine;
- head‐to‐head comparisons of vaccination with one of the pre‐qualified HPV vaccines compared with one of the other pre‐qualified HPV vaccines;
- different number of doses of the pre‐qualified HPV vaccines.

Please see Table detailing the nodes in the networks.

**2:** Nodes in the network*

**Grouped intervention**	**Subgrouped by dose**
Bi‐valent Cervarix	1 dose
	2 doses
	3 doses
Quadri‐valent Gardasil	1 dose
	2 doses
	3 doses
Nona‐valent Gardasil‐9	1 dose
	2 doses
	3 doses
Bi‐valent Cecolin	1 dose
	2 doses
	3 doses
**Control groups**	
Injection control**: adjuvant placebo; saline placebo; non‐HPV control vaccine (active control, e.g. hepatitis A virus vaccine)	
No intervention control	

*The nodes presented in Table are for the following 6 networks: Network 1: Safety and efficacy of HPV vaccines in females ≤ 14 years Network 2: Safety and efficacy of HPV vaccines in females 15 to 25 years Network 3: Safety and efficacy of HPV vaccines in females > 25 years Network 4: Safety and efficacy of HPV vaccines in males ≤ 14 years Network 5: Safety and efficacy of HPV vaccines in males 15 to 25 years Network 6: Safety and efficacy of HPV vaccines in males > 25 years **The components of injection control (saline, adjuvant, other non‐HPV vaccine) were analysed in subgroup analyses, see Subgroup analysis and investigation of heterogeneity.

We did not assess schedules including more than three doses of HPV vaccine.

We assumed that all included interventions are legitimate alternatives and can therefore be considered jointly randomisable. That is, any patient that meets the inclusion criteria will, in principle, be equally likely to be randomised to any of the eligible interventions.

#### Types of outcome measures

We used data from the longest follow‐up time reported, unless otherwise stated below.

We classified outcomes as critical or important to patients and policymakers. We included critical outcomes in the summary of findings tables (see Summary of findings and assessment of the certainty of the evidence).

Whilst we recognise the importance of serious adverse events (those causing death, disability or hospitalisation), we also realise the importance of those adverse events perceived by patients as most prevalent and those adverse events that may prevent uptake. We have therefore conducted surveillance of the social media platforms WebMD and X (formerly Twitter) (Appendix 1). We identified reports of 276 adverse events on WebMD, which we analysed by frequency and added pertinent adverse events to our strategy. We also identified 9781 tweets on HPV and found that injury was the top mentioned adverse event (51%), followed by death (23%) as well as similar adverse events to those in WebMD and concern about the HPV vaccine promoting sexual promiscuity.

##### Primary outcomes

- Invasive cervical, vaginal, vulval, anal, penile, or head and neck cancer rates (critical outcome). The different types of cancer were analysed separately. In the summary of findings tables, the different cancer rates were presented separately, except vaginal and vulval cancer, which was presented as a composite outcome. We recognise that most RCTs are unlikely to report on these outcomes since they require very long‐term follow‐up due to the natural history of HPV‐related cancers. Nevertheless, we include them since they are the ultimate outcomes HPV vaccination is aiming to prevent.
- In females, histologically confirmed high‐grade cervical (CIN2, CIN3 and adenocarcinoma in situ (AIS)), vaginal (VAIN), vulval (VIN) or anal intraepithelial neoplasia (AIN), irrespective of HPV genotype, or any lesions associated with the HPV genotypes included in the vaccine (critical outcome). The cervical lesions were analysed as composite outcomes of grade 2 or worse and grade 3 or worse (i.e. CIN2+ and CIN3+), with separate analyses showing the components of these.
- In males, histologically confirmed penile (PeIN) or anal (AIN) intraepithelial neoplasia of any grade irrespective of HPV genotype, or any lesions associated with the HPV genotypes included in the vaccine (critical outcome). These lesions were analysed as a composite outcome, with separate analyses showing the components of these.
- Serious adverse events (that are fatal, life‐threatening, result in hospitalisation, persistent or significant disability/incapacity, congenital anomaly/birth defect, or require intervention to prevent permanent impairment or damage (FDA 2022)) (critical outcome) were analysed as a composite outcome. We performed a separate analysis for serious adverse events related to the vaccines (as assessed and reported in the studies).

##### Secondary outcomes

- Reduction in treatment rates for CIN, AIN and other HPV‐related pre‐invasive disease (critical outcome).
- Anogenital warts (critical outcome).
- Participation rates in cervical screening.
- In females, adverse pregnancy outcomes, including any adverse pregnancy outcome; fetal or infant abnormality; cervical cerclage and incompetence; miscarriage (or spontaneous abortion); pre‐term birth; stillbirth (or late fetal loss).
- Any local adverse events (overall local/injection site adverse events, redness, swelling, pain at the injection site) (important outcome) up to seven days after vaccination.
- Any overall systemic events and general symptoms (important outcome) up to seven days after vaccination.
- Total adverse events (solicited, unsolicited or both) (important outcome) up to 28 days after vaccination.
- Adverse events that led to discontinuation of the intervention (important outcome).
- Specific adverse events (important outcome): incidence of postural tachycardia syndrome (POTS); chronic fatigue syndrome/myalgic encephalomyelitis (CFS/ME); paralysis; complex regional pain syndrome (CRPS); premature ovarian failure; Guillain‐Barré syndrome; infertility; change in sexual activity. We only included events that were ascertained in the studies; we did not attempt to retrospectively classify events into these outcome categories.
- All‐cause mortality (important outcome). We tabulated causes of death where this information was available (see Table).
- Incident infection with vaccine HPV genotypes (HPV 16 and HPV 18 jointly; HPV 6, HPV 11, HPV 16 and HPV 18 jointly; and HPV 31, HPV 33, HPV 45, HPV 52 and HPV 58 jointly) (important outcome).
- Persistent infection (persisting during at least six months or at least 12 months) with vaccine HPV genotypes (important outcome).

**3:** Causes of death

**Study**	**Intervention arm**	**Control arm**	**Relatedness to intervention**
2v CVT 2011‐CRI	Cervarix: 8/3727 Traffic accident (1); physical assault (2); ovarian cancer (1); Crohn’s disease (1); systemic lupus erythematosus (1); HIV‐associated conditions (1); acute myocardial infarction (1)	Non‐HPV control vaccine: 7/3739 Suicide (4); traffic accident (2); colon neoplasm (1)	Crohn’s disease (1) fatality was assessed by the investigator as possibly related to the study vaccination
2v Konno 2010‐JPN	Cervarix: 1/519 Completed suicide	Non‐HPV control vaccine: 0/521	Assessed by the investigator as not related to the study vaccination
2v PATRICIA 2012‐INT	Cervarix: 10/9319	Non‐HPV control vaccine: 13/9325	No deaths were considered possibly related to vaccination in either the vaccine group or control group
	All 23 fatalities (causes not reported per arm): traffic accidents, homicide or suicide (11); systemic lupus erythematosus (1); myocarditis (1); deep vein thrombosis (1); sepsis (1); subarachnoid haemorrhage (1); pyoderma gangrenosum (1); gestational trophoblastic tumour and metastases to lung (1); pneumococcal sepsis and cardiopulmonary failure (1); bone sarcoma (1); diabetic ketoacidosis (1); cardio‐respiratory arrest (1); tuberculosis meningitis with obstructive hydrocephalus (1)		
2v VIVIANE 2014‐INT	Cervarix: 13/2877 Acute myocardial infarction (1); breast cancer metastatic (1); bronchopneumonia (1); cervix cancer metastatic (1); drug hypersensitivity and acute renal failure (1); glioblastoma multiforme (1); homicide (1); interstitial lung disease (1); lung neoplasm malignant (1); multiple myeloma and pulmonary embolism (1); suicide (3)	Adjuvant placebo: 5/2870 Anaplastic astrocytoma (1); cardiac valve disease and liver disorder (1); cardiorespiratory arrest (1); lower respiratory tract infection and sepsis (1); nasopharyngeal cancer (1)	No deaths were considered by the investigator to be related to study vaccination
2v Zhu 2014‐CHNa	Cervarix: 0/3026	Adjuvant placebo: 3/3025 Gastric neoplasm (1); suicide (2)	Assessed by the investigator as not related to vaccination
2v Zhu 2014‐CHNc	Cervarix: 1/606 Intracranial haemorrhage resulting from a car accident	Non‐HPV control vaccine: 0/606	The investigator considered this SAE not to be related to vaccination
4v FUTURE 2007‐INT	Gardasil: 2/2673 Traffic accident; suicide	Adjuvant placebo: 2/2672 Traffic accident; deep vein thrombosis, renal insufficiency and shock to the lung	Neither of these deaths were considered by the investigator to be related to the vaccine/placebo
4v FUTURE II 2007‐INT	Gardasil: 7/6019 Pneumonia and sepsis (1); overdose of an illicit drug (1); traffic accident (3); pulmonary embolism (1); infective thrombosis (1)	Adjuvant placebo: 5/6031 Suicide (2); asphyxia (1); traffic accident (2)	None of the deaths were judged by the research investigator to be related to vaccine/placebo
4v FUTURE III 2009‐INT	Gardasil: 7/1890 Cardiac arrest secondary to breast cancer metastasis; cardiac arrest secondary to cerebrovascular accident; acute liver disease secondary to nasopharyngeal cancer; breast cancer; tuberculosis; pulmonary embolism; pericarditis	Adjuvant placebo: 1/1888 Pulmonary embolism	No study deaths were deemed by investigators as related to vaccination
4v Giuliano 2011‐INT	Gardasil: 3/2020 Traffic accident (2); gunshot wound (1)	Adjuvant placebo: 10/2029 Gunshot wound (3); drug overdose (2); suicide (2); traffic accident (1); chemical poisoning (1); myocardial ischaemia (1)	No serious adverse events (deaths included) related to vaccination were reported
4v Mikamo 2019‐JPN	Gardasil: 0/554	Adjuvant placebo: 1/559 Suicide	This death was not considered vaccine‐related.
4v Wei 2019‐CHN	Gardasil: 2/1499 Traffic accident; stage 3 ovarian cancer	Adjuvant placebo: 0/1498	Neither death was considered related to vaccination
Cecolin 2v Qiao 2020‐CHN	Cecolin: 3/3391 Traffic accident; suicide; diabetic ketoacidosis (participant with a history of type 2 diabetes for > 10 years)	Non‐HPV control vaccine: 3/3681 Traffic accident (3)	The data and safety monitoring board did not consider any of the SAEs, death or not, to be related to vaccination
2v4v Einstein 2009‐USA	Cervarix: 1/553 Metastatic renal cell carcinoma	Gardasil: 0/553	The event was considered by the investigator to be unrelated to vaccination
2v4v Leung 2015‐INT	Cervarix: 0/359	Gardasil, 2 doses: 0/358 Gardasil, 3 doses: 1/ 358 Suicide	The investigator considered that there was no causal relationship between the suicide and the vaccine administered
2v9v DoRIS 2022‐TZN	Cervarix: 0/456	Gardasil‐9: 1/456 Severe malaria	No SAE was considered to be related to the vaccine
4v9v Joura 2015‐INT	Gardasil‐9: 6/7071 Acute B cell lymphoblastic leukaemia (1); suicide (1); traffic accident (1); hypovolemic shock and septic shock following a salpingo‐oophorectomy for an ovarian tumour, appendectomy and tubal sterilisation (1); sudden unexpected death syndrome (1); acute promyelocytic leukaemia (1)	Gardasil: 5/7078 Airplane crash (1); gunshot trauma (1); suicide (1); adenocarcinoma of the stomach (1); intraparenchymal cerebral haemorrhage (1); traffic accident (1)	None of the deaths were considered vaccine‐related by the reporting investigator
9v Iversen 2016‐INT	Gardasil‐9, 2 doses: 0/294	Gardasil‐9, 3 doses: 1/300 Autoimmune encephalitis and status epilepticus followed by cardiac arrest	None of these SAEs were considered vaccine related

**Abbreviations**: HIV: human immunodeficiency virus; HPV: human papillomavirus; SAE: serious adverse event Causes of death from studies reporting on all‐cause mortality or fatal adverse events. Twenty‐one studies reporting no deaths are not included in the table.

We also collected information from each trial on methods of adverse events data monitoring and collection (Table) based on the CONSORT statement (Ioannidis 2004; Lineberry 2016), including:

**4:** Adverse events methods

**Study**	**Mode of data collection**	**Time frame**	**Attribution methods**	**Vaccine‐related AEs**	**Intensity of ascertainment**	**Harms‐related monitoring and stopping rules**	**Frequency‐based filter**
2v4v Draper 2013‐UK	Proactively collected	Specified	Blinded investigators Prespecified events	Not reported	30 min after vaccination, then during 7 days until the end of follow‐up (month 15) (local/systemic AEs, SAEs)	No information	No information
2v4v Einstein 2009‐USA	Proactively collected	Specified	Blinded investigators Prespecified events	Yes, determined by blinded investigators	7 days after each dose (local/systemic AEs); 30 days after each vaccine dose (solicited/unsolicited/all AEs); then to month 60 (adverse pregnancy outcome, SAE)	No information	No information
2v4v Gilca 2015‐CAN	Proactively collected	Specified	Blinded participants and investigators Prespecified events	Not reported	5 days using standardised diary cards (local/systemic AEs); 12 months (SAEs, passive)	No information	No information
2v4v Leung 2015‐INT	Proactively collected	Specified	Participants and investigators attributed events as adverse, but blinding not reported Prespecified events	Yes, determined by investigators (blinding not reported)	7 days after each vaccination (local/general symptoms); 30 days after each vaccine dose (unsolicited AEs); throughout the study (adverse pregnancy outcome, SAEs)	No information	No limit
2v4v Nelson 2013‐HKG	Proactively collected	Specified	Blinded participants Prespecified events	Not reported	Each evening for 7 days (oral temperature); 14 days following each vaccination (injection site reactions and adverse effects)	No information	No limit
2v4v Sangar 2015‐IND	Proactively collected (not reported for SAE)	Specified	No information about who attributed events as adverse, but attribution was reported as blinded (local/systemic/any/total AEs) Not reported (SAE)	Yes. No information about who reported on vaccine‐related AEs, but relatedness was reported as blinded	Up to 7 days after each dose using a diary card (local AEs); up to 7 days using a diary card (any/total AEs, unwanted events were followed for 14 days after each dose); up to 7 months (vaccine‐related SAEs)	No information	No limit (not reported for any/total AE)
2v9v DoRIS 2022‐TZN	Proactively collected	Specified	Unblinded investigators	Yes. No information about who reported on vaccine‐related AEs, but relatedness was reported as unblinded	From enrolment to month 24 (SAEs, adverse pregnancy outcome)	No information	No limit
2v9v Gilca 2018‐CAN	Proactively collected	Specified	Blinded participants Prespecified events	Not reported	4 days after each dose (local/systemic AEs) using standardised diaries	No information	No information
2v9v KENSHE 2021‐KEN	Both active and passive	Specified	Blinded participants	Not reported	3 months, 6 months, 12 months, 18 months (SAEs, systemic AEs, adverse pregnancy outcome)	No information	No information
2v Bhatla 2010‐IND	Proactively collected	Specified	Blinded investigators Prespecified events	Yes, determined by blinded investigators	During 0 to 7 days post‐vaccination, then until 30 days and to the end of the study (SAEs, AEs that led to discontinuation, local/systemic/any/total/unsolicited/solicited AEs, adverse pregnancy outcome, specific AEs)	No information	No information
2v Carozzi 2016‐ITA	This study did not report on adverse events.						
2v CVT 2011‐CRI	Proactively collected	Specified	Blinded participants and investigators	Yes, determined by blinded investigators	Within 60 min after vaccination and from days 3 to 6 post‐vaccination for a 10% subsample of participants (local/systemic AEs); to 30 days after each dose (unsolicited AEs) and throughout the duration of the study (SAEs)	No information	Yes, specify %:
2v Garcia‐Sicilia 2010‐EU	Proactively collected	Specified	Unblinded investigators Prespecified events	Yes, determined by unblinded investigators	Until 7 days post‐vaccination (local/systemic AEs), then until 30 days (AEs that led to discontinuation, any/total AEs, adverse pregnancy outcome)	No information	No information
2v Harper 2004‐BRA	Both active and passive	Specified	Blinded participants and investigators	Yes, determined by investigators (blinding not reported)	Diary for the first 7 days, then interview at 30 days (SAEs, AEs that led to discontinuation, local/systemic/any/total/unsolicited/solicited AEs, adverse pregnancy outcome)	No information	No information
2v Khatun 2012‐BGD	Not reported	Specified	Not reported	Yes (no further details)	No information	No information	No information
2v Kim 2010‐KOR	Proactively collected (local/systemic AEs) Both active and passive (SAEs, AEs that led to discontinuation, any/total/unsolicited AEs) Not reported (adverse pregnancy outcome)	Specified	Blinded investigators Prespecified events	Yes, determined by blinded investigators (not reported for adverse pregnancy outcome)	At vaccination and follow‐up visits or participants could report at any time (SAEs, AEs that led to discontinuation, any/total/unsolicited AEs); at follow‐up visits or participants could report at any time (local/systemic AEs); observed for 30 minutes, then predefined adverse events recorded each of 7 days after each vaccination using a diary card (along with participant's assessment of severity) for adverse pregnancy outcome	No information	No information
2v Kim 2011‐KOR	Proactively collected	Specified	Blinded investigators (SAEs, AEs that led to discontinuation, any/total/unsolicited AEs) Blinded participants (local/systemic AEs) Not reported (adverse pregnancy outcome)	Yes, determined by blinded investigators. For adverse pregnancy outcome, no information about who reported on vaccine‐related AEs, but relatedness was reported as blinded	Days 0 to 6 follow‐up period after each vaccination using diary cards (local/systemic AEs); to day 30 after each dose using diary cards (any/unsolicited AEs); for the entire 7‐month follow‐up duration (SAEs)	No information	No limit
2v Konno 2010‐JPN	Proactively collected (local/systemic AEs) Both active and passive (SAEs, AEs that led to discontinuation, adverse pregnancy outcome, unsolicited AEs)	Specified	Blinded investigators (SAEs, unsolicited AEs, AEs that led to discontinuation, adverse pregnancy outcome) No information about who attributed events as adverse, but attribution was reported as blinded (local/systemic AEs)	Yes, determined by blinded investigators	Day 0 to 6 after each dose (local/systemic AEs); at each study visit and spontaneously (SAEs, AEs that led to discontinuation, adverse pregnancy outcome); during day 0 to 30 after each dose (unsolicited AEs)	No information	No limit
2v Lehtinen 2018‐FIN	Proactively collected (local/systemic/unsolicited AEs) Both active and passive (SAEs, AE that led to discontinuation, specific AEs) Passively collected /spontaneously reported (adverse pregnancy outcome)	Specified	Unblinded participants and investigators, Prespecified events (SAEs, AEs that led to discontinuation, specific/local/systemic/unsolicited AEs) No information about who attributed events as adverse, but attribution was reported as blinded (adverse pregnancy outcome)	Yes, determined by unblinded investigators	Up to age 18.5 passive/spontaneous for adverse pregnancy outcome; up to months 7 and 12 (active subset); up to age 18.5 passive/spontaneous (SAEs, AE that led to discontinuation, specific AEs); at vaccination visits (0, 1, 6 months), and at 7‐month visit using a diary card (local/systemic/unsolicited AEs)	No information	No limit
2v Leroux‐Roels 2011‐BEL	Proactively collected	Specified	Unblinded investigators	Yes, determined by unblinded investigators	Up to 6 days (local/systemic AEs); within 30 days after each dose (AEs); throughout the study (SAEs, adverse pregnancy outcome)	No information	No information
2v Lim 2014‐MYS	Proactively collected	Specified	Blinded investigators Prespecified events	Yes, determined by blinded investigators	To 7 days after each/any dose (local/systemic); to 30 days after each/any dose (any/total/solicited/unsolicited AEs); during entire study period to 7 months (SAEs, AEs leading to discontinuation, pregnancy events, specific AEs)	No information	No information
2v Lin 2018‐LA	Proactively collected	Specified	Unblinded investigators	Yes, determined by unblinded investigators	Daily during the 7‐day period (days 0 to 6) following each vaccination (SAEs, AEs that led to discontinuation, local/systemic/any/total/specific AEs)	Yes. "The study will be put on hold and subject to GSK Vaccine Safety Monitoring Board (VSMB) and IDMC review in case of:– any fatal or life‐threatening SAE considered as related to vaccination with HPV vaccine; – any fatal or life‐threatening SAE unrelated to vaccination for which no alternative cause can be provided (i.e., an event related to an accident does not constitute an event that applies to this holding rule)"	No information
2v Medina 2010‐INT	Proactively collected	Specified	Blinded participants (local/systemic AEs) Blinded participants and investigators (unsolicited AEs) Blinded investigators (SAEs, AEs that led to discontinuation, adverse pregnancy outcome)	Yes (only unsolicited AEs), determined by a blinded investigator	During 7 days after each dose using a symptom diary (unsolicited AEs); at months 1, 2, 6 months (local/systemic AEs, SAEs, AEs that led to discontinuation, adverse pregnancy outcome)	Yes. "Your daughter/ward’s participation in the study may be stopped [if] the study doctor decides it is in the best interest of her health and welfare to discontinue participation in the study"	No limit
2v Ngan 2010‐HKG	Proactively collected (local/systemic/any/total/unsolicited/solicited AEs) Passively collected /spontaneously reported (SAEs, adverse pregnancy outcome)	Specified	Blinded investigators Prespecified events	Yes, determined by blinded investigators	Daily for 7 days after each dose using a diary card (local/systemic AEs); at months 1, 2, 7 (any/total/unsolicited/solicited AEs, SAEs, adverse pregnancy outcome)	Yes. In the patient information document: "participation in the study may be stopped [if] The study doctor decides it is in the best interest of your health and welfare to discontinue participation in the study."	No limit
2v PATRICIA 2012‐INT	Proactively collected	Specified	Blinded participants (unsolicited AEs) Blinded participants and investigators (SAEs, AEs that led to discontinuation, adverse pregnancy outcome, specific AEs) Prespecified events (local/systemic/any/total/unsolicited AEs)	Yes, determined by blinded investigators	Up to 30 days after each dose using diary cards (safety‐subset, including pre‐specified local/systemic AEs to 7 days after each dose); at every visit (SAEs, AEs that led to discontinuation, adverse pregnancy outcome, specific AEs)	No information	No limit
2v Pedersen 2012‐NA/EU	Proactively collected	Specified	Unblinded investigators Prespecified events	Yes, determined by unblinded investigators	To 7 days after each dose (local/systemic AEs); to 30 days after each dose (any/total/unsolicited AEs); to 12 months (SAEs, fatal SEAs, SAEs related to vaccine, AEs leading to discontinuation)	No information	No information
2v Petaja 2009‐FIN	Proactively collected	Specified	Blinded participants (local/systemic AEs) Blinded investigators (unsolicited/any/total AEs, AEs that led to discontinuation, SAEs)	Yes, determined by blinded investigators. Not reported for SAEs and AEs that led to discontinuation	At day 0, month 1 (30 days post‐dose 1), month 2 (30 days post‐dose 2, month 6 (dose 3), month 7 (30 days post‐dose 3) and month 12 by telephone contact (SAE, unsolicited AEs); 7 days after each dose using a daily symptom diary (local/systemic AEs)	Yes. In the informed consent form: "Termination of Subjects’ Study Participation. Your participation in the study may be stopped for any of the following reasons: The study doctor decides it is in the best interest of your health and welfare to discontinue participation in the study"	No limit
2v Romanowski 2011‐CAN/GER	Both active and passive	Specified	Blinded participants and investigators	Yes, determined by investigators (blinding not reported)	7 days using a diary card during the 24‐month study period (SAE, local/systemic/unsolicited/solicited AEs, adverse pregnancy outcome)	30 min, 7 days and using a diary card during the 24‐month study period	No limit
2v Schmeink 2011‐NLD/SWE	Both active and passive	Specified	Unblinded investigators Prespecified events	Yes, determined by unblinded investigators	Up to 7 days after each/any dose (local/systemic AEs); up to 30 days after each/any dose (total/any/unsolicited AEs); to end of study (12 months) (SAEs, fatal SAEs)	No information	No limit
2v Sow 2013‐SEN/TZN	Proactively collected	Specified	Blinded investigators (SAEs, AEs that led to discontinuation, adverse pregnancy outcome, any/total/unsolicited/solicited AEs) Blinded participants (local/systemic AEs)	Yes, determined by blinded investigators	At months 0, 1, 2, 4, 6, 7, 10, 12 (SAEs, AEs that led to discontinuation, adverse pregnancy outcome); at visits 30 days after each dose (any/total/unsolicited/solicited AEs); up to 7 days after each dose using diary cards (local/systemic AEs)	No information	No limit
2v VIVIANE 2014‐INT	Proactively collected (local/systemic/solicited AEs) Both active and passive (any/total/unsolicited AEs, SAEs, adverse pregnancy outcome, AEs that led to discontinuation)	Specified	Blinded participants and prespecified events (local/systemic/solicited AEs) Blinded participants and investigators (any/total/unsolicited AEs, SAEs, adverse pregnancy outcome, AEs that led to discontinuation)	Yes, determined by blinded investigators	At 30 days after each dose (any/total/unsolicited AEs); at 30 days after each dose (months 1, 6, 7, 12, 18, 24, 30, 36, 42, 48) (SAEs, adverse pregnancy outcome); for 7 days after each dose (solicited AEs, local/systemic AEs); at months 1, 6, 7, 12, 18, 24, 30, 36, 42, 48 (AEs that led to discontinuation)	Yes. Patient information leaflet: "Your participation in the study may be stopped ...[if] The study doctor decides it is in the best interest of your health and welfare to discontinue participation in the study."	No limit
2v Zhu 2014‐CHNa	Proactively collected	Specified	Blinded investigators	Yes, determined by unblinded investigators	7 days after each dose, then until 30 days, and at the end of the study (month 72) (SAEs, AEs that led to discontinuation, local/systemic/any/total/unsolicited/solicited AEs, adverse pregnancy outcome)	No information	No information
2v Zhu 2014‐CHNb	Proactively collected	Specified	Blinded investigators Prespecified events	Yes, determined by blinded investigators	To 7 days after each dose (local/systemic AEs); to 30 days after each dose (any/total/unsolicited AEs); to months 7 and 12 (extended safety follow‐up) (SAEs, AEs leading to discontinuation, SAEs related to vaccine)	No information	No information
2v Zhu 2014‐CHNc	Proactively collected	Specified	Blinded investigators Prespecified events	Yes, determined by blinded investigators	7 days after each dose (local/systemic AEs), then until 30 days post‐vaccination (any/unsolicited AEs) and at the end of the study (months 7 and 12) (SAE, adverse pregnancy outcome)	No information	No information
4v9v Garland 2015‐INT	Proactively collected	Specified	Blinded investigators and participants Prespecified events	Yes, determined by blinded investigators	5 days after each dose (local AEs); 15 days (systemic/any AEs); 7 months study duration (SAE, adverse pregnancy outcome)	No information	No limit
4v9v Joura 2015‐INT	Proactively collected (local/solicited AEs) Both active and passive (SAEs, adverse pregnancy outcome)	Specified	Blinded investigators (SAEs) No information about who attributed events as adverse, but attribution was reported as blinded (local/solicited AEs)	Yes (only for SAEs), determined by blinded investigators and the Monitor Board	30 minutes after each dose (day 0 and months 2, 6) and reported pre‐specified local reactions on days 1 to 5 after each dose using a vaccine report card (local adverse events); 30 minutes after each dose (day 0 and months 2, 6) and reported pre‐specified local reactions on days 1 to 15 after each dose on a vaccine report card (solicited AEs); from day 1 to 6 months following the last vaccination (other SAEs). Deaths and serious vaccine­‐related adverse events were reported throughout the study; events of fetal loss were reported as serious adverse events for any pregnancy with a last menstrual period before 6 months following the last vaccination.	Yes. Protocol: "a subject/patient may be withdrawn by the investigator or the SPONSOR if he/she violates the study plan or for administrative and/or other safety reasons."	No limit
4v9v van Damme 2016‐EU	Proactively collected	Specified	Blinded investigators (SAEs, AEs that led to discontinuation) Prespecified events (local/systemic/any/total AEs)	Yes, determined by blinded investigators	30 min after each dose, then up to 15 days post‐dose (any/total/local/systemic AEs); 30 min after each dose, then up to 15 days post‐dose, then up to month 7 (SAEs, AEs that led to discontinuation)	No information	No limit (SAEs, AEs that led to discontinuation); ≥ 2% (local/systemic AEs)
4v9v Vesikari 2015‐EU	Both active and passive	Specified	Blinded investigators	Yes, determined by blinded investigators	First 30 min, at the day 1, month 2 and month 6 reported from day 1 to day 5 after any vaccination (oral temperature); from day 1 to day 15 after any vaccination (injection‐site reactions, systemic AEs) using a report card	No information	No information
4v Chang 2020‐USA	Proactively collected	Specified	Blinded participants	Yes (no further details)	For 30 min after vaccination (unsolicited/systemic AEs/ solicited injection site/body temperature) and up to 7 days (unsolicited AEs) using diary cards, digital thermometers; up to day 30 (unsolicited AEs); up to 6 (Groups 1 and 2) or 7 months (Groups 3 and 4) after the first dose (SAEs)	No information	No information
4v Dobson 2013‐CAN	Both active and passive	Specified	Not reported	Not reported	At 2, 4, 12 months or if the participant called with concerns (SAEs)	No information	No information
4v EVRI 2016‐ZAF	Not reported	Not reported	Not reported	Not reported	No information	No information	No information
4v Foresta 2015‐ITA	This study did not report on adverse events.						
4v FUTURE 2007‐INT	Proactively collected	Specified	No information about who attributed events as adverse, but attribution was reported as blinded	Yes (no further details)	To day 5 after vaccination (local/systemic AEs); through the study duration (SAE, pregnancy adverse outcome)	No information	1% for non‐serious and non‐pregnancy events
4v FUTURE II 2007‐INT	Both active and passive	Not reported	Not reported	Not reported	First 30 min, then until 15 days after each injection (local/systemic AEs). Deaths and pregnancy‐related adverse events were reported throughout the trial	No information	1% for other AEs
4v FUTURE III 2009‐INT	Proactively collected	Not reported	Not reported	Yes, determined by investigators (blinding not reported)	Days 1 to 15 after each vaccination visit using a vaccine report card (body temperatures, local/systemic AEs); during the entire study period by general questioning at study (other AEs, SAEs, adverse pregnancy outcome, mortality)	No information	No limit (local/systemic AEs); 5% (other AEs)
4v Giuliano 2011‐INT	Proactively collected	Specified	Blinded investigators	Yes, determined by blinded investigators	On days 1 through 5 using vaccination report cards (local AEs); on days 1 through 15 after receiving each dose (systemic AEs); during the entire study period (SAEs)	No information	≥ 1%
4v Kang 2008‐KOR	Proactively collected	Specified	Blinded investigators	Yes, determined by blinded investigators	For 14 days after vaccination using a vaccination report card (SAEs, AEs that led to discontinuation, local/systemic/any/total AEs)	No information	5% (NCT record)
4v Li 2012‐CHN	Proactively collected	Specified	Blinded investigators (SAEs) No information about who attributed events as adverse, but attribution was reported as blinded (local/systemic/any/total AEs)	Yes, determined by blinded investigators (systemic AEs, SAEs) Not reported (any/total AEs)	Daily for days 1 to 15 after each vaccination using a vaccination report card (all AEs); 4 h after vaccination and for the next 4 days following vaccination (body temperature; immediately following vaccination and for the next 4 days (injection site AEs); for 14 days following each dose (systemic AEs); at months 2, 6, 7 and throughout the study (SAEs, any new medical condition or health concerns)	Yes. "A subject may be withdrawn by the investigator or the SPONSOR if he/she violates the study plan or for administrative and/or other safety reasons".	No limit (SAEs); information (any/local/total AEs); systemic AEs reported in ≥ 4 participants
4v Mikamo 2019‐JPN	Proactively collected Passively collected/spontaneously reported (SAEs)	Specified	Blinded participants	Yes, determined by blinded investigators	For 5 days for local and 15 days (systemic AEs); for 15 days after each dose (AEs and SAEs). Also, SAEs were collected for the entire study duration (57 months).	No information	≥ 1% (local/systemic AEs)
4v Mugo 2015‐AF	Proactively collected	Specified	No information about who attributed events as adverse, but attribution was reported as blinded	Yes. No information about who reported on vaccine‐related AEs, but relatedness was reported as blinded	To 15 days after any injection (AEs, local/systemic AEs); to end of study (SAEs)	No information	Incidence> 5% among any qHPV vaccination group
4v NCT00411749 2006‐JPN	Proactively collected	Specified	Not reported	Not reported	On days 0 to 15 following each vaccine dose (local/systemic AEs); to month 7 (SAE)	No information	No information
4v NCT01461993 2011‐USA	Proactively collected	Specified	No information about who attributed events as adverse, but attribution was reported as blinded	Not reported	1 month after 3rd dose (local/systemic/any AEs); 6 months after the last dose (SAE)	No information	No information
4v Reisinger 2007‐INT	Proactively collected Both active and passive (SAEs)	Specified	Blinded investigators (only SAEs) Prespecified events	Yes, determined by blinded investigators (SAE) Not reported (local/systemic/any/total AEs)	For 14 days following each dose using a vaccination report card (systemic/any/ total AEs) and at months 2, 6, 7, 12, 18 included an interview to assess general safety (SAEs); for 5 days following each injection (body temperature); days 1 to 5 following any vaccination visit (local AEs)	No information	No limit
4v S+A54enders 2016‐USA	Proactively collected	Specified	Not reported	Yes. No information about who reported on vaccine‐related AEs, but relatedness was reported as blinded	Up to 7 days after each dose (local/systemic AEs, AEs that led to discontinuation)	No information	No information
4v Villa 2005‐INT	Proactively collected	Specified	Blinded investigators	Yes, determined by blinded investigators	To 14 days after each dose using a diary card reporting (local/systemic AEs); to day 5 (body temperature). Registry: "At Months 2, 3, 6, and 7, subjects were solicited for any gynecological health concerns and any SAEs that had occurred".	No information	No information
4v Wei 2019‐CHN	Proactively collected	Specified	Blinded investigators (SAEs, AEs that led to discontinuation, any/total AEs, adverse pregnancy outcome) Prespecified events (local/systemic AEs)	Yes, determined by blinded investigators	For 15 days using vaccine report cards (local/systemic AEs). SAEs were actively solicited at each study visit by the investigators, and spontaneously reported by the participants during the intervals between two visits (each dose, month 30 and 78 and telephone call at month 90)	No information	No information
4v Yoshikawa 2013‐JPN	Proactively collected (local/systemic/any/total AEs) Passively collected /spontaneously reported (SAEs, adverse pregnancy outcome)	Specified	No information about who attributed events as adverse, but attribution was reported as blinded	Yes, determined by blinded investigators (local/systemic/any/total AEs)	Daily for 4 days after vaccination (body temperature); for 15 days after vaccination using a standard diary card (any AEs). Registry: "events were collected by non‐systematic assessment."	No information	No limit
9v Iversen 2016‐INT	Not reported	Specified	Unblinded investigators	Yes, determined by unblinded investigators	From day 1 (month 0) through 6 months after the last dose/37 months (NCT) (SAEs)	No information	No information
Cecolin 2v Hu 2020‐CHN	Proactively collected (local/systemic AEs) Both active and passive (SAEs) Passively collected /spontaneously reported (unsolicited AEs)	Specified	Unblinded participants (unsolicited AEs, SAEs) No information about who attributed events as adverse, but unblinded (local/systemic AEs)	Not reported	For 30 min after each vaccination and actively followed up by the investigators on days 7 and 30 after each vaccination using a diary card. SAEs that occurred from the first vaccination through one month after the final dose were carefully investigated and recorded.	No information	No limit
Cecolin 2v Qiao 2020‐CHN	Proactively collected (local/systemic AEs) Passively collected /spontaneously reported (unsolicited AEs, SAEs, adverse pregnancy outcome)	Specified (local/systemic/unsolicited AEs) Not reported (SAEs, adverse pregnancy outcome)	Blinded participants (unsolicited AEs, SAEs, adverse pregnancy outcome) No information about who attributed events as adverse, but attribution was reported as blinded (local/systemic AEs)	Not reported	Within 1 month after each dose using diary cards (all AEs); within 72 h after each dose (local/systemic AEs). Unsolicited AEs included any AEs that occurred during the period from day 8 to day 30 after each vaccination and any AEs that occurred within 7 days after each vaccination but that had not been listed on the diary card for registering solicited AEs. Throughout the trial, all serious adverse events (SAEs) and pregnancy outcome were requested to be reported.	No information	1%
Cecolin 2v Wu 2015‐CHN	Proactively collected	Specified	Investigators attributed events as adverse, but blinding not reported Prespecified events	Yes, determined by investigators (blinding not reported)	6 h, 24 h, 48 h, 72 h, 7 days (local/systemic AEs), 14 to 28 days after each dose (unsolicited AEs); to month 7 (SAEs)	No information	No information

**Abbreviations**: AE: adverse event; SAE: serious adverse event

- Mode of data collection: proactive monitoring, spontaneous reporting, or both.
- Timing: whether the timeframe of adverse events collection was reported and, if so, what it was.
- Attribution methods: who attributed events as adverse and whether they were blinded to the intervention, definitions used.
- Intensity of ascertainment.
- Harms‐related monitoring and stopping rules.
- Frequency‐based filter: limiting reporting to adverse events experienced by a minimum percentage of study participants.

Finally, we collected information from each trial about whether the adverse events were considered to be vaccine‐related and how this was determined within each trial (e.g. by trialists or by an independent monitoring board).

It should be noted that POTS, CFS/ME and CRPS are diagnoses of exclusion, and global population background rates are not well‐established. We therefore sought to ascertain rates of these and other specific diagnoses, rather than rely on a constellation of symptoms that might or might not be indicative of these rare syndromes.

### Search methods for identification of studies

We attempted to identify all relevant studies regardless of language or publication status (published, unpublished, in press and in progress).

#### Electronic searches

The Information Specialist at the Cochrane Gynaecological, Neuro‐oncology and Orphan Cancers group designed search strategies and ran the searches in the core databases on 10 January 2022. An update search was run on 18 September 2024 in:

- the Cochrane Central Register of Controlled Trials (CENTRAL; 2024, Issue 9), in the *Cochrane Library*;
- MEDLINE Ovid (2000 to 18 September 2024);
- Embase Ovid (2000 to 18 September 2024).

Due to the timeline of HPV vaccine development, searches earlier than 2000 were not required.

Search strategies are presented in Appendix 2.

We did not apply language restrictions to the electronic searches.

#### Searching other resources

We searched the following databases for related systematic reviews and ongoing studies, and checked the reference lists of those that were relevant, for additional studies:

- Epistemonikos: https://www.epistemonikos.org;
- ClinicalTrials.gov:http://clinicaltrials.gov;
- WHO International Clinical Trials Registry Platform (ICTRP): www.who.int/clinical-trials-registry-platform;
- HTA Database (Health Technology Assessments Database): www.york.ac.uk/crd/#HTA.

We used all studies that we identified as relevant as seeds in PubMed to search for additional studies using the related articles feature. We also used the relevant studies as seeds in the Science Citation Index ISI Web of Knowledge ResearchGate and Google Scholar to determine whether articles citing these studies were also relevant.

We handsearched abstract books of meetings of the International Gynecologic Cancer Society, the European Society of Gynaecological Oncology, International Papillomavirus Meetings, European Research Organisation on Genital Infection and Neoplasia (EUROGIN) and the Society of Gynecologic Oncology from 2010 to the latest edition, to identify ongoing and unpublished studies.

In addition, we searched vaccine manufacturer websites for relevant clinical study reports (CSR) (GlaxoSmithKline; Merck). We also screened a list of HPV vaccine studies (Jørgensen 2018c), which was constructed through enquiries to HPV vaccine manufacturers and regulators, as well as searches of trial registers and journal publication databases. For each included study, where available, we identified and screened study governance documents (protocols, trial registration listings and results, manufacturers' clinical study reports) for relevant data and outcomes. We also applied for access to any missing study governance documents through the European Medicines Agency (EMA).

### Data collection and analysis

We uploaded the results of all searches to DistillerSR to aid sifting and remote teamwork (DistillerSR 2021). We used RevMan Web for review production, using standard Cochrane methods (RevMan 2025).

#### Selection of studies

Search results were put through the RCT classifier (Thomas 2021), which uses machine learning to sift out irrelevant studies and automate some aspects of review production. Citations and abstracts were screened independently, in duplicate by two review authors. A third review author resolved any disagreements. We obtained full‐text reports for all potentially eligible studies. Two independent review authors determined the eligibility of studies for inclusion in the review from the full reports according to predefined criteria. A third systematic review author resolved any disagreements.

#### Data extraction and management

Two review authors carried out data extraction independently using pre‐tested data extraction forms. Study characteristics and outcome data were independently extracted, and any differences were resolved by discussion between the two review authors and referral to the study reports.

We named studies based on the vaccine, first‐named study author, year of publication and country. Many studies have more than one document associated with them: journal publications (main study reports, reports of long‐term follow‐up, secondary outcomes and post hoc analyses), conference abstracts and study governance documents (protocols, trial registration listings and results, manufacturers' clinical study reports). For each study, we grouped these documents together and designated one report as the primary reference for the study; the study name is derived from this particular report. Where available, the CSR was considered the primary reference. When CSRs were not available for a study, we considered the main peer‐reviewed publication reporting on primary outcome/s the primary reference. For unpublished studies, we considered results reported in trial registries as the primary reference.

If data between the different study documents differed, we extracted data from the primary reference.

#### Outcome data

We collected outcome definitions and time points for each outcome.

All outcomes were dichotomous. We collected the number of participants experiencing an outcome event and the number analysed in each intervention group. Where only rates were reported, we collected the number of events and the person‐years in each intervention group. Where data per group were not available, we extracted any relative effect estimates reported.

We used data with the longest follow‐up time for the primary analysis.

#### Study characteristics

From each included study we extracted data on the following study methods, interventions and population characteristics:

- Methods: randomisation (individual or cluster), duration of follow‐up, number of study centres, location, inclusion and exclusion criteria, and date of study.
- Participants: number, setting* high‐ (HIC), upper‐middle‐ (UMIC), lower‐middle‐ (LMIC) or low‐income country (LIC) using World Bank classifications (World Bank 2025), age at first dose*, sex*, sexual orientation, sexual history*, HPV DNA positive or negative at first dose*, morbidities (including HIV, previous HPV disease history and other genital infections), smoking status*, drug misuse, indicators of socioeconomic status/poverty.
- Interventions: type of vaccine, number of doses and schedule*, comparison group*, co‐interventions (e.g. presence of a screening programme in the country*, co‐administration of other vaccines).
- Outcomes: primary and secondary outcomes specified and collected, and time points reported.
- Notes: sponsorship/funding for trial* (public/non‐profit or industry/private), notable conflicts of interest of trial authors*, trial registry ID numbers.

We considered variables marked with an asterisk (*) as effect modifiers. These were evaluated in the context of the transitivity assumption by comparing the distribution of the potential effect modifiers across the available direct comparisons in the network (see Subgroup analysis and investigation of heterogeneity). Further, we carried out subgroup analyses on variables described in the Subgroup analysis and investigation of heterogeneity section. Analyses are stratified by type of vaccine, sex and age at first dose (see Data synthesis).

#### Assessment of risk of bias in included studies

We employed standard Cochrane methodology for assessment of the risk of bias of the included studies. This involved the use of the updated Cochrane risk of bias tool (RoB 2) for assessing risk of bias in randomised trials (Sterne 2019). To manage RoB 2 assessments, we used a form in DistillerSR (DistillerSR 2021), based on the RoB 2 Excel tool to implement RoB 2 (available on the riskofbias.info website). Two review authors independently assessed the risk of bias for each critical outcome per study. We resolved any disagreements through discussion. We assessed the risk of bias for those outcome measures and time points selected for the summary of findings tables. We assessed the effect of assignment to intervention at baseline (the 'intention‐to‐treat effect'), regardless of whether the interventions were received as intended.

We assessed the risk of bias in the following domains: 1) risk of bias arising from the randomisation process; 2) risk of bias due to deviations from intended interventions; 3) risk of bias due to missing outcome data; 4) risk of bias in measurement of the outcome; 5) risk of bias in selection of the reported result; 6) overall risk of bias based on the assessments in the five domains. For cluster‐RCTs, we also assessed and reported the risk of bias associated with an additional domain: timing of identification or recruitment of participants in a cluster.

In the RoB 2 tool, there are a series of signalling questions within each domain that elicit information relevant to the assessment. The response options to the signalling questions are 'yes,' 'probably yes,' 'probably no,' 'no' and 'no information'. A risk of bias judgement arising from each domain is generated by an algorithm, based on answers to the signalling questions. Judgements can be 'low risk of bias,' 'some concerns' or 'high risk of bias'. We considered the overall risk of bias to be low if all domains were at low risk; some concerns if at least one domain was of some concern and no domain was at high risk; and high risk of bias if there was at least one domain considered to be at high risk, or several domains with some concerns (Higgins 2021).

When we assessed the risk of bias due to deviations from intended interventions, we considered a national cervical cancer screening programme as a co‐intervention that may have an impact on study outcomes if it differed between intervention and control groups.

#### Relative treatment effects

We estimated the pairwise relative treatment effects of the competing interventions using risk ratios (RRs) with their respective 95% confidence intervals (CIs) for dichotomous outcomes. Efficacy outcomes were presented as % vaccine efficacy (VE = (1‐RR)*100).

We carried out a complete‐case analysis (the number analysed) and an intention‐to‐treat analysis (see Sensitivity analysis).

#### Relative treatment ranking

We obtained a hierarchy of the competing interventions using the surface under the cumulative ranking curve (SUCRA) and mean ranks. We obtained a hierarchy of the different interventions according to the critical outcomes.

#### Cluster‐randomised trials

We included one cluster‐RCT, but the results were not analysed with other studies because the cluster‐RCT was considered too heterogenous to compare with the other studies (see description of Included studies ‐ Outcomes). Had we included cluster‐RCTs in meta‐analysis, we would have extracted the intra‐cluster correlation coefficient (ICC) when available, and recorded the number of clusters per group, the total size of clusters per group and the unit of randomisation (e.g. household or geographical area). The statistical methods used to analyse the trial results would have been documented, along with details describing whether these methods were adjusted for clustering or for other co‐variables.

We would have pooled cluster‐RCT data that had been adjusted for clustering with data from trials that randomly assigned individuals (individual‐RCTs) using the generic inverse variance random‐effects method. If the results of a cluster‐RCT had not been adjusted for clustering, we would have adjusted the data using the clustering effect (ICC) imputed from another study (see Chapter 23.1.4 of the *Cochrane Handbook for Systematic Reviews of Interventions*) (Higgins 2022). Finally, we would have performed sensitivity analyses excluding cluster‐RCTs.

#### Cross‐over trials

Had cross‐over trials been identified, or should cross‐over trials be identified for any future update of this review, then we would only include the first phase, before crossing over, in the review results.

#### Trials with multiple intervention arms

We analysed trials with multiple intervention arms as follows:

- Arms of different concentrations, formulations or lots of the same HPV vaccine were combined.
- Arms of different doses were combined in all pairwise analyses except in analyses of dose comparisons. They were not combined in network meta‐analyses.
- Arms of different intervals between doses were combined, but evaluated separately in subgroup analyses.
- Arms co‐administering a non‐HPV vaccine with an HPV vaccine and arms of HPV vaccine alone were combined, but evaluated separately in subgroup analyses.
- Arms of different non‐HPV vaccines as control were combined.

#### Dealing with missing data

If data on specific outcomes or population groups were missing, we attempted to contact study authors or data owners to request these data. We did not impute missing outcome data. Where missing data were substantial (> 5%), we assessed the risk of bias using the RoB 2 tool as some concerns, or high risk (Sterne 2019).

#### Assessment of heterogeneity

We assessed the presence of clinical heterogeneity within each pairwise comparison by comparing the trial and study population characteristics across all eligible trials.

#### Measures and tests for heterogeneity

For pairwise analyses, we inspected forest plots visually to detect heterogeneity. We reported the I^2^ values with 95% CIs to indicate statistical heterogeneity.

We assessed statistically the presence of heterogeneity in the entire network by calculating I^2^. For I^2^, we considered that values over 50% suggest the presence of substantial heterogeneity in the entire network.

#### Assessment of statistical incoherence

##### Local approaches for evaluating incoherence

To evaluate the presence of local incoherence, we used the *network* macro for Stata (Stata 2017). We considered loops/comparisons as potential sources of incoherence in the network based on the SIDE (Separating Indirect from Direct Evidence) approach.

##### Global approaches for evaluating incoherence

To evaluate coherence in the entire network simultaneously, we used the *network* macro for Stata to develop incoherence models.

#### Assessment of reporting biases

To assess the risk of bias in a synthesis when entire studies or particular results within studies were missing selectively, we used the following methods when possible:

- Retrieved protocols and trial registry entries as part of our search and contacted study authors of planned studies where results have not been made available for more information.
- Used the ORBIT tool on each included study by recording whether trials planned or measured outcomes but failed to report on them (Kirkham 2018).
- Inspected funnel plots for asymmetry (Page 2021; Sterne 2011).
- Undertook network meta‐regression to account for small‐study effects (Chaimani 2012).

We carried out assessments of reporting biases for the critical outcomes.

#### Methods for direct treatment comparisons

For pairwise comparisons, we performed standard meta‐analyses using a random‐effects model in RevMan Web (RevMan 2025). We stratified analyses by type of vaccine, number of doses, sex and age at first dose. If age groups were mixed or unknown within a study and could not be disaggregated, we placed studies in an age group if ≥ 75% of participants qualified for that age group. If the proportions were more equal or unknown, we analysed the study in a mixed age stratum.

#### Methods for indirect and mixed comparisons

We included RCTs in the NMA providing that populations of the included studies were sufficiently similar to satisfy the assumption of joint randomisation and that the interventions connect, creating a network. We planned for six networks of sufficiently similar populations, for females and males and for age groups (younger adolescents ≤ 14 years, older adolescents 15 to 25 years, and adults > 25 years) (see Table). For the critical outcomes and latest time points, we estimated the effects (risk ratios (RRs)) of the interventions and their 95% CIs using the random‐effects model in Stata fitting a multivariate network.

#### Subgroup analysis and investigation of heterogeneity

We assessed the assumption of transitivity by comparing the distribution of the potential effect modifiers (listed in the Data extraction and management section) across the different pairwise comparisons.

If there was a sufficient number of included studies, we performed meta‐regression analyses on the critical efficacy outcomes by using the following effect modifiers to explore their influence as possible sources of incoherence.

- Setting: LIC, MIC, HIC.
- HPV DNA positivity status at baseline: HPV seropositive/HPV seronegative.
- History of sexual activity: yes/no.
- Presence of screening programme: yes/no/mixed (for multinational trials). Data on screening programmes were obtained from the WHO (WHO 2019).
- Schedule: longer (> 2 months) or shorter (≤ 2 months) duration between doses.
- Type of comparison group: saline placebo, adjuvant placebo, other non‐HPV vaccine, no intervention.
- Funding source for trials: public/non‐profit or industry/private.
- Study authors' conflicts of interest: yes/no.

#### Sensitivity analysis

To test the robustness of the data, we planned to carry out several sensitivity analyses for the critical outcomes. We carried out sensitivity analyses for intention‐to‐treat (ITT) analysis: we prioritised available case analyses to avoid making assumptions about missing data. We carried out sensitivity analyses using the ITT denominators. For the remaining planned sensitivity analyses there were no data (rare events, high risk of bias, trials with abstracts only, cluster trials).

#### Summary of findings and assessment of the certainty of the evidence

We prepared summary of findings tables according to a format developed by Yepes‐Nuñez (Yepes‐Nuñez 2019) for the following outcomes that were assessed as critical according to GRADE guidelines (Guyatt 2011):

- For females: invasive cervical cancer; HPV‐associated vulval, vaginal, head and neck, or anal cancer; high‐grade CIN+; HPV‐associated VIN, VAIN or AIN; treatment rates for CIN and other HPV‐related pre‐invasive disease; anogenital warts; and serious adverse events.
- For males: invasive anal cancer; HPV‐associated penile or head and neck cancer; histologically confirmed PeIN or AIN; HPV‐associated PeIN or AIN; treatment rates for AIN and other HPV‐related pre‐invasive disease; anogenital warts; and serious adverse events.

We assessed the certainty of evidence in the review through discussion between review authors using the GRADE approach (Guyatt 2011). We considered the following factors for downgrading the certainty of the evidence: limitations in the study design (overall risk of bias); inconsistency of results (heterogeneity); indirectness of evidence (applicability); imprecision (few events and wide confidence intervals); and publication bias (Guyatt 2011; Puhan 2014).

When the certainty of evidence was downgraded, we detailed the reasons in footnotes of the summary of findings tables and summarise these in the Quality of the evidence section. Depending on whether the evidence was downgraded or not, we rated the certainty of the evidence for each outcome as follows.

- High‐certainty evidence indicates that we are very confident that the true effect lies close to that of the estimate of the effect (evidence was not downgraded).
- Moderate‐certainty evidence indicates that we are moderately confident in the effect estimate: the true effect is likely to be close to the estimate of the effect, but there is a possibility that it is substantially different (evidence was downgraded one step for any of the factors described above).
- Low‐certainty evidence indicates that our confidence in the effect estimate is limited: the true effect may be substantially different from the estimate of the effect (evidence was downgraded two steps for any of the factors described above).
- Very low‐certainty evidence indicates that we have very little confidence in the effect estimate: the true effect is likely to be substantially different from the estimate of effect (evidence was downgraded three steps for any of the factors described above).

##### Stakeholder engagement

HPV vaccination is a major target for misinformation, especially targeting parents and carers via social media. We aimed to provide robust and unbiased evidence for patients, clinicians and policymakers, to enable fully informed decision‐making. This Cochrane HPV vaccine NMA was conducted in parallel with a Cochrane review on the long‐term population impact of HPV vaccination, mainly from observational studies. These reviews are both high priority for Cochrane and will inform the WHO and national government screening and immunisation strategies at a global level. We are aware that this will subject the review authors to significant scrutiny from communities with concerns about vaccination in general, and HPV vaccination specifically, but we are committed to promoting evidence‐based health care and improving outcomes for HPV‐related disease globally.

An Independent Advisory Group (IAG), including consumers, has advised on review production and content.

## Results

### Description of studies

#### Results of the search

Electronic database searches resulted in 16,683 records. We retrieved 449 records from additional sources: 407 from Epistemonikos and HTA databases, 25 CSRs from the GlaxoSmithKline (GSK) trial registry, six CSRs from the EMA, two CSRs from Health Canada and nine records from handsearching.

After de‐duplication of the retrieved records, 14,598 records were screened. We excluded 14,132 records after screening the titles and abstracts. We retrieved full texts for the remaining 466 records. We excluded 223 full texts (we only report on the 48 most relevant excluded studies in the Excluded studies); 60 studies (reported in 208 records) were included, including three pooled analyses reporting on more than one included study. In addition, 15 relevant studies are ongoing (reported in 16 references) and 16 studies are awaiting classification (reported in 19 references). See Figure for a flow diagram of the search and screening process.

**1:**
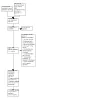


#### Included studies

We identified 60 included studies reported in 208 records. Three published analyses of more than one of the included studies were also used (4v FUTURE I/II 2010‐INT; 4v Villa/FUTURE I/II 2009‐INT; 4v Yoshikawa/NCT00411749 2013‐JPN). See Characteristics of included studies for full details on study characteristics, summarised below.

#### Methods

All studies were randomised. One study was cluster‐randomised (2v Lehtinen 2018‐FIN).

#### Setting

Most studies were multi‐centre (two to 135 centres); 10 studies were conducted at a single centre.

Thirty‐seven studies were carried out in one country. Of these, eight studies were in China; four in Japan; three in the USA; three in Canada; three in South Korea; two studies each in Finland, Hong Kong, India and Italy; and one study each in Bangladesh, Belgium, Costa Rica, Kenya, Malaysia, South Africa, Tanzania and the UK.

Twenty‐three studies were multi‐country trials. Of these, 12 were carried out in countries on more than two continents, four studies were in European countries, two in African countries, two in European and North American countries, one in European and Asian countries, one in Latin American countries, and one in North and South American countries.

Seven studies were carried out in lower‐middle income countries, 13 in upper‐middle income countries, 27 in high‐income countries and 13 in multiple countries of more than one income category.

Fifty studies were carried out in countries with existing national cervical screening programmes. Screening coverage ranged from less than 10% in three studies, 10% to 50% in 12 studies, 50% to 70% in three studies, and 70% or over in 13 studies. Twelve studies had unknown coverage and seven studies were carried out in countries with different coverage ranging from less than 10% to 70% or over.

#### Participants

A median number of 782 participants were included per trial, ranging from 11 (2v4v Nelson 2013‐HKG) to 34,412 (2v Lehtinen 2018‐FIN). Four trials included more than 10,000 participants (2v Lehtinen 2018‐FIN; 2v PATRICIA 2012‐INT; 4v9v Joura 2015‐INT; 4v FUTURE II 2007‐INT). In total, the trials included 157,414 participants. Of these, 110,005 were female (from 49 trials), 6031 were male (from five trials) and six trials with 41,378 participants included both females and males.

Eighteen studies randomised 45,718 participants that were 16 years or younger, and 12 of these included 7172 participants that were 14 years or younger, including 2v Lin 2018‐LA that included young children aged four to six years old. Twenty‐one studies included 79,009 participants within the range 15 to 27 years old, and 15 of these included 58,073 participants up to 25 years old. Three studies included 10,778 participants over 24 years old. Seventeen studies included participants with broader age ranges: 10 studies included 8828 participants within the range of 9 to 25 years old, six included 12,409 participants within the range of 18 to 45 years old, and one study included 600 participants aged 9 to 45 years (4v Li 2012‐CHN). One study did not report age at first dose (4v Foresta 2015‐ITA).

#### Interventions

Thirty‐four studies assessed Cervarix, 29 assessed Gardasil, eight assessed Gardasil‐9 and three assessed Cecolin. Thirteen of these studies assessed more than one of the eligible HPV vaccines.

Fifty studies had two arms. Twenty‐three of these compared three doses of one of the HPV vaccines with a non‐vaccine injection control. Twenty‐one of the control arms were administered adjuvant only (no viral components) and two saline placebo. Nine of these were Cervarix studies and 14 were Gardasil studies. Ten of the two‐arm studies compared an HPV vaccine with a non‐HPV vaccine (hepatitis A virus, hepatitis B virus, hepatitis E virus, measles, mumps and rubella (MMR) or diphtheria, tetanus and pertussis (DTP) vaccines). Nine of these were Cervarix studies and one Cecolin (Cecolin 2v Qiao 2020‐CHN). Three of the two‐arm studies compared an HPV vaccine with no intervention (2v Carozzi 2016‐ITA; 2v Khatun 2012‐BGD; 4v Foresta 2015‐ITA). Seven of the two‐arm trials compared one HPV vaccine with another (2v4v Draper 2013‐UK; 2v4v Einstein 2009‐USA; 2v4v Nelson 2013‐HKG; 2v4v Sangar 2015‐IND; 4v9v Joura 2015‐INT; 4v9v Van Damme 2016‐EU; 4v9v Vesikari 2015‐EU), and three compared a different number of doses (4v Dobson 2013‐CAN; 9v Iversen 2016‐INT; Cecolin 2v Hu 2020‐CHN). Three studies compared a homologous schedule with a schedule of two different HPV vaccines (2v4v Gilca 2015‐CAN; 2v9v Gilca 2018‐CAN; 4v9v Garland 2015‐INT).

Eleven studies had more than two arms. Three‐arm studies included 2v4v Leung 2015‐INT that compared different vaccines and doses, 2v9v KENSHE 2021‐KEN that compared one dose of different HPV vaccines and placebo, and three trials that compared an HPV vaccine co‐administered with other vaccines (2v Garcia‐Sicilia 2010‐EU; 2v Pedersen 2012‐NA/EU; 2v Schmeink 2011‐NLD/SWE). Four‐arm studies included 2v Lehtinen 2018‐FIN, 4v Chang 2020‐USA, 4v Senders 2016‐USA and Cecolin 2v Wu 2015‐CHN, which included arms with different dose strengths as well as a non‐vaccine control or a non‐HPV comparison vaccine. 2v Romanowski 2011‐CAN/GER also included arms with different dose strength and also different numbers of doses. Finally, 2v9v DoRIS 2022‐TZN had six arms and compared one, two and three doses of two different HPV vaccines.

Most studies administered standard dose concentrations with the recommended interval between doses (month 0, 1 and 6 for Cervarix and Cecolin, and month 0, 2 and 6 for Gardasil and Gardasil‐9). Two‐dose arms administered at month 0 and 6 (2v Lin 2018‐LA; 4v Dobson 2013‐CAN; 9v Iversen 2016‐INT; Cecolin 2v Hu 2020‐CHN), month 0 and 2 or 0 and 6 (2v Romanowski 2011‐CAN/GER) or one dose (2v9v DoRIS 2022‐TZN; 2v9v KENSHE 2021‐KEN). 2v4v Gilca 2015‐CAN administered the third dose at month 42.

#### Outcomes

Twenty‐one studies (35%) reported on efficacy outcomes.

All studies, except 2v Carozzi 2016‐ITA and 4v Foresta 2015‐ITA, reported on safety outcomes. Twenty‐nine studies (78% of studies including females > 15 years) reported on adverse pregnancy outcomes.

Follow‐up ranged from four days after each dose to 11 years, with a median of 12 months follow‐up.

Six studies did not provide quantitative data for any of the meta‐analyses:

- Three trials compared a mixed vaccine schedule with a homologous schedule and reported on immunogenicity and safety outcomes:
  - 2v4v Gilca 2015‐CAN compared a Gardasil versus Cervarix booster in 9‐ to 10‐year‐old girls who had received two doses of Gardasil 42 months earlier.
  - 2v9v Gilca 2018‐CAN compared one dose of Cervarix followed by one dose of Gardasil‐9 with two doses of Gardasil‐9 in 9‐ to 10‐year‐old girls and boys.
  - 4v9v Garland 2015‐INT compared three doses of Gardasil‐9 with saline placebo in 12‐ to 26‐year‐old females that had received three doses of Gardasil one year earlier.
- One study reported outcome data for the vaccine group only (2v Khatun 2012‐BGD).
- One cluster trial randomised different vaccination strategies and was considered too heterogenous to be pooled with the other trials. In addition, it did not report outcome data for randomised groups, but for participants that received the vaccine compared with those that did not (2v Lehtinen 2018‐FIN).
- One study did not report on outcomes relevant to this review: HPV DNA in semen following HPV vaccination in infertile men (4v Foresta 2015‐ITA).

#### Funding and study authors' conflicts of interest

##### Funding

Forty‐four studies were funded by the vaccine developers and/or manufacturers GlaxoSmithKline (25 studies), Merck (18 studies), Sanofi Pasteur (three studies), Innovax (two studies) or Pfizer (two studies). Seven of these studies also received funding from public or non‐profit institutions. Another two studies had other commercial funders: 2v4v Nelson 2013‐HKG received academic funding but also study materials from PharmaJet; 2v Khatun 2012‐BGD received funding from Grameenphone Ltd, Bangladesh.

Eight studies had only public or non‐profit funders. These included one Cervarix study (2v Carozzi 2016‐ITA), one Cecolin study (Cecolin 2v Wu 2015‐CHN), and six studies assessing more than one HPV vaccine (2v4v Draper 2013‐UK; 2v4v Gilca 2015‐CAN; 2v4v Sangar 2015‐IND; 2v9v DoRIS 2022‐TZN; 2v9v Gilca 2018‐CAN; 2v9v KENSHE 2021‐KEN).

One study did not report how the trial was funded (4v Foresta 2015‐ITA).

##### Conflicts of interest

Conflicts of interest could not be detected among study authors of eight trials (2v4v Draper 2013‐UK; 2v4v Gilca 2015‐CAN; 2v4v Sangar 2015‐IND; 2v9v DoRIS 2022‐TZN; 2v9v Gilca 2018‐CAN; 2v Carozzi 2016‐ITA; 2v Khatun 2012‐BGD; 2v Kim 2010‐KOR).

In 48 trials, authors had conflicts of interest due to being affiliated with or employed by and/or owning stock or holding patents in the vaccine development or manufacturing company.

Four trials did not report on conflicts of interest (2v Konno 2010‐JPN; 4v9v Vesikari 2015‐EU; 4v FUTURE 2007‐INT; 4v NCT00411749 2006‐JPN).

#### Studies awaiting classification

There are 16 studies reported in 19 references awaiting classification.

In the update search on 18 September 2024, we identified 11 relevant references:

- Three new RCTs (4v‐Cecolin Zaman 2024‐BGD/GHA; 9v Berenson 2024‐USA; 9v MacCosham 2022‐CAN).
- Eight companion papers to already included studies:
  - one companion to 2v CVT 2011‐CRI (Shing 2022);
  - one companion paper to 2v Zhu 2014‐CHNa (Zhao 2023);
  - one companion paper to 2v9v DoRIS 2022‐TZN (Watson‐Jones 2023);
  - one companion paper to 2v9v KENSHE 2021‐KEN (Barnabas 2023);
  - one companion paper to 4v Wei 2019‐CHN (Zhao 2022a);
  - one companion paper to Cecolin 2v Hu 2020‐CHN (Yao 2022);
  - two companion papers to Cecolin 2v Qiao 2020‐CHN (Zhao 2022b; Zhong 2023).

These papers are unlikely to have an impact on the critical outcomes in this review. Most of them (10 out of 11) presented secondary analyses that do not change the results from the primary publications already included in the review. One study reported only on outcomes that are not critical (9v MacCosham 2022‐CAN). See Studies awaiting classification for full details.

Another five studies are awaiting classification (see Studies awaiting classification for full details). We either cannot tell if these studies are eligible (HPV‐003; NCT00520598; V503‐018) or we have not been able to access any relevant data (NCT05149248; Rivera 2018). We have contacted the investigators to confirm study eligibility and availability of data. At the time of writing this review, no conclusive responses have been received.

#### Ongoing studies

We identified 15 relevant ongoing studies. Four were conducted in China (NCT03998254: Gardasil‐9 versus Gardasil in 20‐ to 45‐year‐old females; NCT05279248: Cecolin with different doses in 13‐ to 14‐year‐old females; NCT05415345: Cecolin versus control (hepatitis E vaccine) in 18‐ to 25‐year‐old females; NCT06345885: Cecolin versus Gardasil in 9‐ to 14‐year‐old females). Three were conducted in the USA (Giuliano 2022: Gardasil‐9 versus saline placebo in 20‐ to 45‐year‐old males; NCT03943875: Gardasil‐9 versus delayed Gardasil‐9 in 15‐ to 26‐year‐old females and males; NCT05672927: two versus three doses of Gardasil in 24‐ to 45‐year‐old females). Two each were conducted in Costa Rica (ESCUDDO: one versus two doses of Gardasil or Cervarix in 12‐ to 16‐year‐old girls; PRISMA ESCUDDO: one dose of Gardasil‐9 versus Cervarix in 18‐ to 30‐year‐old females) and Japan (NCT04635423: Gardasil‐9 versus saline placebo in 16‐ to 26‐year‐old males; NCT04772534: two versus three doses of Gardasil‐9 in 9‐ to 15‐year‐old boys and girls), and one each was conducted in Canada (ICI‐VPH: two versus three doses of Gardasil in 9‐ to 14‐year‐old females), the Gambia (HANDS: one versus two versus three doses of Gardasil‐9 in 4‐ to 26‐year‐old females), Mexico (Salmeron 2016: Gardasil versus Cervarix in 25‐ to 45‐year‐old females) and Tanzania (Add‐Vacc: single‐dose Gardasil versus no intervention in 14‐ to 18‐year‐old males). See Characteristics of ongoing studies for full details.

#### Excluded studies

Here we report on the 48 most relevant excluded studies:

- 13 were not randomised;
- 18 had other comparisons that were not relevant to this review;
- 14 reported only on immunogenicity outcomes for already included or excluded studies, or for pooled analyses of included and excluded studies;
- two assessed other HPV vaccines;
- one evaluated vaccination to prevent HPV recurrence.

See Characteristics of excluded studies for full details.

### Risk of bias in included studies

We assessed risk of bias for all critical outcomes using the Cochrane risk of bias tool (RoB 2) (Sterne 2019) and the ORBIT tool (Kirkham 2018). Full details can be found in Characteristics of included studies, at doi: 10.17605/OSF.IO/WM8PU and in Table.

**5:** Outcome reporting bias

**TRIALS INCLUDING FEMALES**											
**Study**	**Prospective protocol?**	**Cervical cancer**	**Vulval or vaginal cancer**	**High‐grade CIN**				**High‐grade VIN or VaIN**	**Treatment for HPV‐related disease**	**Anogenital warts**	**Serious adverse events**
				**CIN3+, any HPV**	**CIN3+, vaccine‐type**	**CIN2+, any HPV**	**CIN2+, vaccine‐type**				
2v4v Draper 2013‐UK	Yes**	D ‐ Not reported, not planned	D ‐ Not reported, not planned	D ‐ Not reported, not planned				D ‐ Not reported, not planned	D ‐ Not reported, not planned	D ‐ Not reported, not planned	A ‐ Reported as planned
2v4v Einstein 2009‐USA	Yes**	D ‐ Not reported, not planned	D ‐ Not reported, not planned	D ‐ Not reported, not planned				D ‐ Not reported, not planned	D ‐ Not reported, not planned	D ‐ Not reported, not planned	A ‐ Reported as planned
2v4v Gilca 2015‐CAN	Yes	D ‐ Not reported, not planned	D ‐ Not reported, not planned	D ‐ Not reported, not planned				D ‐ Not reported, not planned	D ‐ Not reported, not planned	D ‐ Not reported, not planned	A ‐ Reported as planned
2v4v Leung 2015‐INT	Yes	D ‐ Not reported, not planned	D ‐ Not reported, not planned	D ‐ Not reported, not planned				D ‐ Not reported, not planned	D ‐ Not reported, not planned	D ‐ Not reported, not planned	A ‐ Reported as planned
2v4v Nelson 2013‐HKG	Yes	D ‐ Not reported, not planned	D ‐ Not reported, not planned	D ‐ Not reported, not planned				D ‐ Not reported, not planned	D ‐ Not reported, not planned	D ‐ Not reported, not planned	G ‐ Reported but unable to use^a^
2v4v Sangar 2015‐IND	Yes	D ‐ Not reported, not planned	D ‐ Not reported, not planned	D ‐ Not reported, not planned				D ‐ Not reported, not planned	D ‐ Not reported, not planned	D ‐ Not reported, not planned	A ‐ Reported as planned
2v9v DoRIS 2022‐TZN	Yes	D ‐ Not reported, not planned	D ‐ Not reported, not planned	D ‐ Not reported, not planned				D ‐ Not reported, not planned	D ‐ Not reported, not planned	D ‐ Not reported, not planned	A ‐ Reported as planned
2v9v Gilca 2018‐CAN*	Yes**	D ‐ Not reported, not planned	D ‐ Not reported, not planned	D ‐ Not reported, not planned				D ‐ Not reported, not planned	D ‐ Not reported, not planned	D ‐ Not reported, not planned	D ‐ Not reported, not planned
2v9v KENSHE 2021‐KEN	Yes	D ‐ Not reported, not planned	D ‐ Not reported, not planned	D ‐ Not reported, not planned				D ‐ Not reported, not planned	D ‐ Not reported, not planned	D ‐ Not reported, not planned	A ‐ Reported as planned
2v Bhatla 2010‐IND	Yes	D ‐ Not reported, not planned	D ‐ Not reported, not planned	D ‐ Not reported, not planned				D ‐ Not reported, not planned	D ‐ Not reported, not planned	D ‐ Not reported, not planned	A ‐ Reported as planned
2v Carozzi 2016‐ITA	No	E ‐ Not reported, no plan available	E ‐ Not reported, no plan available	E ‐ Not reported, no plan available				E ‐ Not reported, no plan available	E ‐ Not reported, no plan available	E ‐ Not reported, no plan available	E ‐ Not reported, no plan available
2v CVT 2011‐CRI	Yes**	D ‐ Not reported, not planned	D ‐ Not reported, not planned	B ‐ Reported, not planned		A ‐ Reported as planned		D ‐ Not reported, not planned	B ‐ Reported, not planned	D ‐ Not reported, not planned	A ‐ Reported as planned
2v Garcia‐Sicilia 2010‐EU	Yes	D ‐ Not reported, not planned	D ‐ Not reported, not planned	D ‐ Not reported, not planned				D ‐ Not reported, not planned	D ‐ Not reported, not planned	D ‐ Not reported, not planned	G ‐ Reported but unable to use^b^
2v Harper 2004‐BRA	Yes	D ‐ Not reported, not planned	D ‐ Not reported, not planned	D ‐ Not reported, not planned	A ‐ Reported as planned	D ‐ Not reported, not planned	A ‐ Reported as planned	D ‐ Not reported, not planned	D ‐ Not reported, not planned	D ‐ Not reported, not planned	A ‐ Reported as planned
2v Khatun 2012‐BGD	No	E ‐ Not reported, no plan available	E ‐ Not reported, no plan available	E ‐ Not reported, no plan available				E ‐ Not reported, no plan available	E ‐ Not reported, no plan available	E ‐ Not reported, no plan available	G ‐ Reported but unable to use^c^
2v Kim 2010‐KOR	Yes**	D ‐ Not reported, not planned	D ‐ Not reported, not planned	D ‐ Not reported, not planned				D ‐ Not reported, not planned	D ‐ Not reported, not planned	D ‐ Not reported, not planned	A ‐ Reported as planned
2v Kim 2011‐KOR	Yes**	D ‐ Not reported, not planned	D ‐ Not reported, not planned	D ‐ Not reported, not planned				D ‐ Not reported, not planned	D ‐ Not reported, not planned	D ‐ Not reported, not planned	A ‐ Reported as planned
2v Konno 2010‐JPN	Yes	D ‐ Not reported, not planned	D ‐ Not reported, not planned	A ‐ Reported as planned	D ‐ Not reported, not planned	B ‐ Reported, not planned	A ‐ Reported as planned	D ‐ Not reported, not planned	D ‐ Not reported, not planned	D ‐ Not reported, not planned	A ‐ Reported as planned
2v Lehtinen 2018‐FIN*	Yes	D ‐ Not reported, not planned	D ‐ Not reported, not planned	D ‐ Not reported, not planned				D ‐ Not reported, not planned	D ‐ Not reported, not planned	D ‐ Not reported, not planned	G ‐ Reported but unable to use^d^
2v Leroux‐Roels 2011‐BEL	Yes	D ‐ Not reported, not planned	D ‐ Not reported, not planned	D ‐ Not reported, not planned				D ‐ Not reported, not planned	D ‐ Not reported, not planned	D ‐ Not reported, not planned	A ‐ Reported as planned
2v Lim 2014‐MYS	Yes**	D ‐ Not reported, not planned	D ‐ Not reported, not planned	D ‐ Not reported, not planned				D ‐ Not reported, not planned	D ‐ Not reported, not planned	D ‐ Not reported, not planned	A ‐ Reported as planned
2v Lin 2018‐LA	Yes	D ‐ Not reported, not planned	D ‐ Not reported, not planned	D ‐ Not reported, not planned				D ‐ Not reported, not planned	D ‐ Not reported, not planned	D ‐ Not reported, not planned	A ‐ Reported as planned
2v Medina 2010‐INT	Yes	D ‐ Not reported, not planned	D ‐ Not reported, not planned	D ‐ Not reported, not planned				D ‐ Not reported, not planned	D ‐ Not reported, not planned	D ‐ Not reported, not planned	A ‐ Reported as planned
2v Ngan 2010‐HKG	Yes	D ‐ Not reported, not planned	D ‐ Not reported, not planned	D ‐ Not reported, not planned				D ‐ Not reported, not planned	D ‐ Not reported, not planned	D ‐ Not reported, not planned	A ‐ Reported as planned
2v PATRICIA 2012‐INT	Yes**	D ‐ Not reported, not planned	D ‐ Not reported, not planned	A ‐ Reported as planned				D ‐ Not reported, not planned	A ‐ Reported as planned	D ‐ Not reported, not planned	A ‐ Reported as planned
2v Pedersen 2012‐NA/EU	Yes	D ‐ Not reported, not planned	D ‐ Not reported, not planned	D ‐ Not reported, not planned				D ‐ Not reported, not planned	D ‐ Not reported, not planned	D ‐ Not reported, not planned	A ‐ Reported as planned
2v Romanowski 2011‐CAN/GER	Yes	D ‐ Not reported, not planned	D ‐ Not reported, not planned	D ‐ Not reported, not planned				D ‐ Not reported, not planned	D ‐ Not reported, not planned	D ‐ Not reported, not planned	A ‐ Reported as planned
2v Schmeink 2011‐NLD/SWE	Yes	D ‐ Not reported, not planned	D ‐ Not reported, not planned	D ‐ Not reported, not planned				D ‐ Not reported, not planned	D ‐ Not reported, not planned	D ‐ Not reported, not planned	A ‐ Reported as planned
2v Sow 2013‐SEN/TZN	Yes	D ‐ Not reported, not planned	D ‐ Not reported, not planned	D ‐ Not reported, not planned				D ‐ Not reported, not planned	D ‐ Not reported, not planned	D ‐ Not reported, not planned	A ‐ Reported as planned
2v VIVIANE 2014‐INT	Yes	D ‐ Not reported, not planned	D ‐ Not reported, not planned	D ‐ Not reported, not planned		B ‐ Reported, not planned	A ‐ Reported as planned	A ‐ Reported as planned	D ‐ Not reported, not planned	D ‐ Not reported, not planned	A ‐ Reported as planned
2v Zhu 2014‐CHNa	Yes	D ‐ Not reported, not planned	D ‐ Not reported, not planned	B ‐ Reported, not planned	D ‐ Not reported, not planned	A ‐ Reported as planned		A ‐ Reported as planned	D ‐ Not reported, not planned	D ‐ Not reported, not planned	A ‐ Reported as planned
2v Zhu 2014‐CHNb	Yes	D ‐ Not reported, not planned	D ‐ Not reported, not planned	D ‐ Not reported, not planned				D ‐ Not reported, not planned	D ‐ Not reported, not planned	D ‐ Not reported, not planned	A ‐ Reported as planned
2v Zhu 2014‐CHNc	Yes	D ‐ Not reported, not planned	D ‐ Not reported, not planned	D ‐ Not reported, not planned				D ‐ Not reported, not planned	D ‐ Not reported, not planned	D ‐ Not reported, not planned	A ‐ Reported as planned
4v9v Garland 2015‐INT	Yes	D ‐ Not reported, not planned	D ‐ Not reported, not planned	D ‐ Not reported, not planned				D ‐ Not reported, not planned	D ‐ Not reported, not planned	D ‐ Not reported, not planned	A ‐ Reported as planned
4v9v Joura 2015‐INT	Yes**	A ‐ Reported as planned	A ‐ Reported as planned	A ‐ Reported as planned		B ‐ Reported, not planned	A ‐ Reported as planned	A ‐ Reported as planned	B ‐ Reported, not planned	A ‐ Reported as planned	A ‐ Reported as planned
4v9v Vesikari 2015‐EU	Yes	D ‐ Not reported, not planned	D ‐ Not reported, not planned	D ‐ Not reported, not planned				D ‐ Not reported, not planned	D ‐ Not reported, not planned	D ‐ Not reported, not planned	A ‐ Reported as planned
4v Chang 2020‐USA*	Yes	D ‐ Not reported, not planned	D ‐ Not reported, not planned	D ‐ Not reported, not planned				D ‐ Not reported, not planned	D ‐ Not reported, not planned	D ‐ Not reported, not planned	A ‐ Reported as planned
4v Dobson 2013‐CAN	Yes	D ‐ Not reported, not planned	D ‐ Not reported, not planned	D ‐ Not reported, not planned				D ‐ Not reported, not planned	D ‐ Not reported, not planned	D ‐ Not reported, not planned	B ‐ Reported, not planned
4v EVRI 2016‐ZAF	Yes	D ‐ Not reported, not planned	D ‐ Not reported, not planned	D ‐ Not reported, not planned				D ‐ Not reported, not planned	D ‐ Not reported, not planned	D ‐ Not reported, not planned	B ‐ Reported, not planned
4v FUTURE 2007‐INT	Yes**	A ‐ Reported as planned	A ‐ Reported as planned	B ‐ Reported, not planned				A ‐ Reported as planned	D ‐ Not reported, not planned	A ‐ Reported as planned	B ‐ Reported, not planned
4v FUTURE II 2007‐INT	Yes**	A ‐ Reported as planned	A ‐ Reported as planned	B ‐ Reported, not planned			A ‐ Reported as planned	B ‐ Reported, not planned	D ‐ Not reported, not planned	D ‐ Not reported, not planned	B ‐ Reported, not planned
4v FUTURE III 2009‐INT	Yes**	D ‐ Not reported, not planned	A ‐ Reported as planned	D ‐ Not reported, not planned		B ‐ Reported, not planned		A ‐ Reported as planned	B ‐ Reported, not planned	A ‐ Reported as planned	A ‐ Reported as planned
4v Kang 2008‐KOR	Yes	D ‐ Not reported, not planned	D ‐ Not reported, not planned	D ‐ Not reported, not planned				D ‐ Not reported, not planned	D ‐ Not reported, not planned	D ‐ Not reported, not planned	A ‐ Reported as planned
4v Li 2012‐CHN*	Yes	D ‐ Not reported, not planned	D ‐ Not reported, not planned	D ‐ Not reported, not planned				D ‐ Not reported, not planned	D ‐ Not reported, not planned	D ‐ Not reported, not planned	A ‐ Reported as planned
4v Mugo 2015‐AF	Yes	D ‐ Not reported, not planned	D ‐ Not reported, not planned	D ‐ Not reported, not planned				D ‐ Not reported, not planned	D ‐ Not reported, not planned	D ‐ Not reported, not planned	A ‐ Reported as planned
4v NCT00411749 2006‐JPN	Yes	D ‐ Not reported, not planned	D ‐ Not reported, not planned	D ‐ Not reported, not planned				D ‐ Not reported, not planned	D ‐ Not reported, not planned	D ‐ Not reported, not planned	B ‐ Reported, not planned
4v Reisinger 2007‐INT*	No	E ‐ Not reported, no plan available	E ‐ Not reported, no plan available	E ‐ Not reported, no plan available				E ‐ Not reported, no plan available	E ‐ Not reported, no plan available	E ‐ Not reported, no plan available	F ‐ Reported, no plan available
4v Senders 2016‐USA*	Yes	D ‐ Not reported, not planned	D ‐ Not reported, not planned	D ‐ Not reported, not planned				D ‐ Not reported, not planned	D ‐ Not reported, not planned	D ‐ Not reported, not planned	A ‐ Reported as planned
4v Villa 2005‐INT	Yes**	D ‐ Not reported, not planned	D ‐ Not reported, not planned	G ‐ Reported but not used^e^	D ‐ Not reported, not planned		B ‐ Reported, not planned	B ‐ Reported, not planned	D ‐ Not reported, not planned	G ‐ Reported but not extractable	A ‐ Reported as planned
4v Wei 2019‐CHN	Yes**	D ‐ Not reported, not planned	D ‐ Not reported, not planned	D ‐ Not reported, not planned			B ‐ Reported, not planned	D ‐ Not reported, not planned	D ‐ Not reported, not planned	D ‐ Not reported, not planned	A ‐ Reported as planned
4v Yoshikawa 2013‐JPN	Yes**	D ‐ Not reported, not planned	D ‐ Not reported, not planned	D ‐ Not reported, not planned				D ‐ Not reported, not planned	D ‐ Not reported, not planned	D ‐ Not reported, not planned	B ‐ Reported, not planned
9v Iversen 2016‐INT	Yes	D ‐ Not reported, not planned	D ‐ Not reported, not planned	D ‐ Not reported, not planned				D ‐ Not reported, not planned	D ‐ Not reported, not planned	D ‐ Not reported, not planned	A ‐ Reported as planned
Cecolin 2v Hu 2020‐CHN	Yes	D ‐ Not reported, not planned	D ‐ Not reported, not planned	D ‐ Not reported, not planned				D ‐ Not reported, not planned	D ‐ Not reported, not planned	D ‐ Not reported, not planned	A ‐ Reported as planned
Cecolin 2v Qiao 2020‐CHN	Yes	D ‐ Not reported, not planned	D ‐ Not reported, not planned	D ‐ Not reported, not planned		A ‐ Reported as planned		A ‐ Reported as planned	D ‐ Not reported, not planned	D ‐ Not reported, not planned	A ‐ Reported as planned
Cecolin 2v Wu 2015‐CHN	Yes	D ‐ Not reported, not planned	D ‐ Not reported, not planned	D ‐ Not reported, not planned				D ‐ Not reported, not planned	D ‐ Not reported, not planned	D ‐ Not reported, not planned	A ‐ Reported as planned
**TRIALS INCLUDING MALES**											
**Study**	**Prospective protocol?**	**Anal cancer**	**Penile cancer**	**High‐grade PeIN**				**High‐grade AIN**	**Treatment for HPV‐related disease**	**Anogenital warts**	**Serious adverse events**
2v9v Gilca 2018‐CAN*	Yes**	D ‐ Not reported, not planned	D ‐ Not reported, not planned	D ‐ Not reported, not planned				D ‐ Not reported, not planned	D ‐ Not reported, not planned	D ‐ Not reported, not planned	D ‐ Not reported, not planned
2v Lehtinen 2018‐FIN*	Yes	D ‐ Not reported, not planned	D ‐ Not reported, not planned	D ‐ Not reported, not planned				D ‐ Not reported, not planned	D ‐ Not reported, not planned	D ‐ Not reported, not planned	G ‐ Reported but unable to use^d^
2v Petaja 2009‐FIN	Yes	D ‐ Not reported, not planned	D ‐ Not reported, not planned	D ‐ Not reported, not planned				D ‐ Not reported, not planned	D ‐ Not reported, not planned	D ‐ Not reported, not planned	A ‐ Reported as planned
4v9v van Damme 2016‐EU	Yes	D ‐ Not reported, not planned	D ‐ Not reported, not planned	D ‐ Not reported, not planned				D ‐ Not reported, not planned	D ‐ Not reported, not planned	D ‐ Not reported, not planned	A ‐ Reported as planned
4v Chang 2020‐USA*	Yes	D ‐ Not reported, not planned	D ‐ Not reported, not planned	D ‐ Not reported, not planned				D ‐ Not reported, not planned	D ‐ Not reported, not planned	D ‐ Not reported, not planned	A ‐ Reported as planned
4v Foresta 2015‐ITA	No	E ‐ Not reported, no plan available	E ‐ Not reported, no plan available	E ‐ Not reported, no plan available				E ‐ Not reported, no plan available	E ‐ Not reported, no plan available	E ‐ Not reported, no plan available	E ‐ Not reported, no plan available
4v Giuliano 2011‐INT	Yes	A ‐ Reported as planned	A ‐ Reported as planned	A ‐ Reported as planned				A ‐ Reported as planned	D ‐ Not reported, not planned	A ‐ Reported as planned	A ‐ Reported as planned
4v Li 2012‐CHN*	Yes	D ‐ Not reported, not planned	D ‐ Not reported, not planned	D ‐ Not reported, not planned				D ‐ Not reported, not planned	D ‐ Not reported, not planned	D ‐ Not reported, not planned	A ‐ Reported as planned
4v Mikamo 2019‐JPN	Yes	D ‐ Not reported, not planned	D ‐ Not reported, not planned	D ‐ Not reported, not planned				D ‐ Not reported, not planned	D ‐ Not reported, not planned	D ‐ Not reported, not planned	A ‐ Reported as planned
4v Reisinger 2007‐INT*	No	E ‐ Not reported, no plan available	E ‐ Not reported, no plan available	E ‐ Not reported, no plan available				E ‐ Not reported, no plan available	E ‐ Not reported, no plan available	E ‐ Not reported, no plan available	F ‐ Reported, no plan available
4v Senders 2016‐USA*	Yes	D ‐ Not reported, not planned	D ‐ Not reported, not planned	D ‐ Not reported, not planned				D ‐ Not reported, not planned	D ‐ Not reported, not planned	D ‐ Not reported, not planned	A ‐ Reported as planned
A: A study result is available for inclusion in the synthesis, as reported in the clinical trial registry or trial protocol. B: A study result is available for inclusion in the synthesis, but not reported in the clinical trial registry or trial protocol. C: No study result is available for inclusion, (probably) because the P value, magnitude or direction of the results were considered unfavourable by the study investigators. The outcome was planned in the clinical trial registry or protocol. D: No study result is available for inclusion, (probably) because the outcome was not assessed, or for a reason unrelated to the P value, magnitude or direction of the results. The outcome is not reported in the clinical trial registry or trial protocol. E: No study result is available for inclusion, and it is unclear if the outcome was assessed in the study. There is no clinical trial registry or trial protocol available for assessment. F: A study result is available for inclusion in the synthesis. There is no clinical trial registry or trial protocol available for assessment. G: A study result is available, but data were not used (see explanations in footnotes).											

**Abbreviations**: AIN: anal intraepithelial neoplasia; CIN3+: cervical intraepithelial neoplasia grade 3 or higher, including CIN grade 3, adenocarcinoma in situ, and invasive cervical cancer; HPV: human papillomavirus; PeIN: penile intraepithelial neoplasia; VaIN: vaginal intraepithelial neoplasia; VIN: vulval intraepithelial neoplasia *Studies including males and females are presented both under females and under males in this table to cover all outcomes. **Protocol or trial registry dated after recruitment start but before analysis start. ^a^Two SAEs were reported but not by arm. ^b^Follow‐up included time period after which control group had received (delayed) intervention vaccine. ^c^No comparative data; SAEs were only reported for the intervention group. ^d^Outcomes were not reported by randomised arms. ^e^CIN3+ reported in pooled analysis but a different pooled analysis with longer follow‐up and more cases was used.

#### Cancer outcomes

For cervical cancer, comparing HPV vaccine with control, we assumed a low overall risk of bias as both trials reporting on this outcome were assessed as low risk of bias in all domains (4v FUTURE 2007‐INT; 4v FUTURE II 2007‐INT). One trial reporting on cervical cancer compared Gardasil‐9 with Gardasil and we assessed it as overall 'some concerns' due to 'some concerns' assessments in two domains (4v9v Joura 2015‐INT): per‐protocol analysis was used, which is not considered appropriate to assess assignment to intervention and over 5% of participant data were missing.

For HPV‐related vaginal or vulval cancer, comparing HPV vaccine with control, we assumed a low overall risk of bias as we assessed the trial reporting on this outcome as low risk of bias in all domains (4v FUTURE 2007‐INT). One trial reporting on HPV‐related vaginal or vulval cancer compared Gardasil‐9 with Gardasil and we assessed it as having some concerns in two domains (4v9v Joura 2015‐INT): per‐protocol analysis was used, which is not considered appropriate to assess assignment to intervention and over 5% of participant data were missing.

For anal cancer and penile cancer, comparing HPV vaccine with control, we assumed a low overall risk of bias as the trial reporting on these outcomes was assessed as low risk of bias in all domains for both outcomes (4v Giuliano 2011‐INT).

Outcome reporting bias was not detected for any of the cancer outcomes (see Table). Most trials had not planned to analyse cancer outcomes. Funnel plot inspection to detect publication bias was not carried out due to too few trials reporting on these outcomes.

#### Pre‐cancer outcomes

For CIN3+ and CIN2+, comparing HPV vaccine with control, we assumed overall 'some concerns' due to 'some concerns' assessments in more than one domain for the studies reporting on this outcome: 4v Wei 2019‐CHN did not supply information on allocation concealment, 2v CVT 2011‐CRI used per‐protocol analysis, which is not considered appropriate to assess assignment to intervention, in four studies over 5% of participant data were missing (2v CVT 2011‐CRI; 2v Harper 2004‐BRA/NA; 2v Konno 2010‐JPN; 2v PATRICIA 2012‐INT), and in seven studies we assessed there was a risk of bias in selection of the reported results since the outcome was not pre‐specified in a protocol or trial registry (2v CVT 2011‐CRI; 2v Konno 2010‐JPN; 2v VIVIANE 2014‐INT; 2v Zhu 2014‐CHNa; 4v FUTURE I/II 2010‐INT; 4v FUTURE III 2009‐INT; 4v Wei 2019‐CHN).

For HPV vaccine‐type high‐grade VIN or VaIN, comparing HPV vaccine with control, we assumed overall some concerns due to some concerns assessments in more than one domain for the studies reporting on this outcome: 2v PATRICIA 2012‐INT used per‐protocol analysis, which is not considered appropriate to assess assignment to intervention, and over 5% of participant data were missing, and 4v Villa/FUTURE I/II 2009‐INT was a published analysis of three studies where allocation concealment was not reported for one of the studies and only one of the three studies in the analysis had pre‐specified the outcome in a protocol or trial registry. One trial reporting on HPV vaccine‐type high‐grade VIN or VaIN compared Gardasil‐9 with Gardasil, and we assessed it as overall low risk of bias due to low risk of bias assessments in all domains (4v9v Joura 2015‐INT).

For high‐grade anal intraepithelial neoplasia and high‐grade penile intraepithelial neoplasia (irrespective of vaccine types and for HPV vaccine‐types), comparing HPV vaccine with control, we assumed a low overall risk of bias as the trial reporting on these outcomes was assessed as low risk of bias in all domains for all these outcomes (4v Giuliano 2011‐INT).

Outcome reporting bias was not detected for any of the critical pre‐cancer outcomes (see Table). Most trials had not planned to analyse critical pre‐cancer outcomes. One study, 4v Villa 2005‐INT, reported on critical outcomes that we did not use because the outcome was reported in a pooled analysis (4v Villa/FUTURE I/II 2009‐INT), where a different pooled analysis with longer follow‐up and more cases was used (4v FUTURE I/II 2010‐INT). We did not carry out funnel plot inspection to detect publication bias due to too few trials reporting on these outcomes.

#### Serious adverse events

Thirty‐nine studies that reported on serious adverse events compared HPV vaccine with control, and we assumed a low overall risk of bias despite 'some concerns' assessments in more than one domain for the studies reporting on this outcome. We assumed low risk of bias since the five largest studies contributing more than 85% to the outcome were all assessed as low risk of bias in all domains (2v CVT 2011‐CRI; 2v PATRICIA 2012‐INT; 2v VIVIANE 2014‐INT; 2v Zhu 2014‐CHNa; Cecolin 2v Qiao 2020‐CHN).

Four trials reporting on serious adverse events compared Gardasil with Cervarix and were assessed as overall low risk of bias due to low risk of bias assessments in all domains for trials contributing to the estimate (2v4v Draper 2013‐UK; 2v4v Einstein 2009‐USA; 2v4v Leung 2015‐INT). Two trials reporting on serious adverse events compared Gardasil‐9 with Cervarix, and we assessed them as overall low risk of bias due to low risk of bias assessments in all domains but one (2v9v DoRIS 2022‐TZN; 2v9v KENSHE 2021‐KEN). One trial reporting on serious adverse events compared Gardasil‐9 with Gardasil, and we assessed it as overall low risk of bias (4v9v Joura 2015‐INT).

For the studies reporting on serious adverse events that compared different doses (one versus two, one versus three, or two versus three), all were assumed as overall low risk of bias due to low risk of bias assessments in all domains (2v4v Leung 2015‐INT) or in all domains but one (2v9v DoRIS 2022‐TZN; 2v Romanowski 2011‐CAN/GER; 4v Dobson 2013‐CAN; 9v Iversen 2016‐INT), except for two versus three doses of Cecolin, which we assumed as overall 'some concerns' due to 'some concerns' in two domains in the study that reported on this outcome and comparison (Cecolin 2v Hu 2020‐CHN). This study did not report information about allocation concealment, giving rise to concerns about risk of bias in the randomisation process, and it was unblinded, with participant self‐report of adverse events giving rise to concerns about risk of bias in measurement of the outcome.

Outcome reporting bias was not detected (see Table). Four studies reported on serious adverse events that we did not use because cases were not reported by arm (2v4v Nelson 2013‐HKG), follow‐up included a time period after which the control group had received (delayed) intervention vaccine (2v Garcia‐Sicilia 2010‐EU), outcome was only reported for the intervention group (2v Khatun 2012‐BGD), and outcomes were not reported by randomised arms (2v Lehtinen 2018‐FIN). We did not detect publication bias upon inspection of a funnel plot (Figure).

**2:**
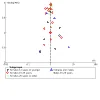
Funnel plot (1.16 Serious adverse events)

#### Treatment for HPV‐related pre‐invasive disease

For treatment for HPV‐related pre‐invasive disease, comparing HPV vaccine with control, we assumed overall 'some concerns' due to 'some concerns' assessments in more than one domain for the studies reporting on this outcome. 2v CVT 2011‐CRI used per‐protocol analysis, which is not considered appropriate to assess assignment to intervention and over 5% of participant data were missing, and in three studies we assessed there was a risk of bias in selection of the reported results since the outcome was not pre‐specified in a protocol or trial registry (2v CVT 2011‐CRI; 4v FUTURE III 2009‐INT; 4v Giuliano 2011‐INT). One trial reporting on treatment for HPV‐related pre‐invasive disease compared Gardasil‐9 with Gardasil and was assessed as overall low risk of bias (4v9v Joura 2015‐INT).

Outcome reporting bias was not detected (see Table). We did not carry out funnel plot inspection to detect publication bias due to too few trials reporting on this outcome.

#### Anogenital warts

For anogenital warts irrespective of HPV type, comparing HPV vaccine with control, we assumed a low overall risk of bias. This was because the trials reporting on these outcomes (4v FUTURE I/II 2010‐INT; 4v Giuliano 2011‐INT) were assessed as low risk of bias in all but one domain (4v FUTURE I/II 2010‐INT was assessed as some concerns in the selective reporting domain due to pooled post hoc analysis). One trial reporting on anogenital warts irrespective of HPV type compared Gardasil‐9 with Gardasil, and we assessed it as overall low risk of bias (4v9v Joura 2015‐INT).

Outcome reporting bias was not detected (see Table). We did not carry out funnel plot inspection to detect publication bias due to too few trials reporting on this outcome.

### Effects of interventions

See: **Summary of findings 1** Summary of findings: Efficacy and safety of any HPV vaccine compared with control (standard meta‐analysis); **Summary of findings 2** Summary of findings: High‐grade CIN by population, sub‐outcome and vaccine; **Summary of findings 3** Summary of findings: Vaccine‐matched HPV type‐associated high‐grade VIN or VaIN* by population and by vaccine; **Summary of findings 4** Summary of findings: High‐grade penile or anal intraepithelial neoplasia by population and by vaccine; **Summary of findings 5** Summary of findings: Treatment rates for HPV‐related pre‐invasive disease by population and by vaccine; **Summary of findings 6** Summary of findings: Anogenital warts irrespective of HPV type by population and by vaccine; **Summary of findings 7** Summary of findings: Serious adverse events by population and by vaccine; **Summary of findings 8** Summary of findings: Serious adverse events by population, vaccine and dose

First, we report results for outcomes assessed as critical, for which NMA and summary of findings tables were prepared. Second, we report standard meta‐analysis results for important outcomes for which NMA and summary of findings tables were not prepared (as planned).

#### 1. Critical outcomes

For critical outcomes where more than two studies were included within a network and the assumption of transitivity was satisfied, we carried out NMA and report the results below. We report on the following networks: females 14 years or younger, females 15 to 25 years, females > 25 years and males 15 to 25 years. There were no trials for males 14 years or younger, or for males > 25 years. We carried out standard meta‐analysis for all critical outcomes, but only report below when it was not possible to carry out NMA or when effects were in different directions for the NMA and standard meta‐analysis estimates. Due to the scarcity of trials for many of the outcomes, we also report standard meta‐analysis results of trials comparing any HPV vaccine with control in all populations.

##### 1.1 Invasive cervical cancer in females

###### 1.1.1 Females 14 years or younger: invasive cervical cancer (no trials)

There were no trials that reported on invasive cervical cancer in females receiving their first dose at 14 years or younger.

###### 1.1.2 Females 15 to 25 years: invasive cervical cancer (NMA not possible)

Three included trials reported no cases of invasive cervical cancer in females aged 15 to 25 years (4v9v Joura 2015‐INT; 4v FUTURE 2007‐INT; 4v FUTURE II 2007‐INT); see Table. Consequently, we did not carry out NMA. Two of the trials assessed Gardasil versus control (17,622 participants, 48 months follow‐up; Analysis 3.5) and one of the trials assessed Gardasil‐9 versus Gardasil (11,386 participants, 72 months follow‐up; Analysis 8.8).

**6:** Cervical disease component outcomes results

**Population**	**RR (95% CI)** **Participants (trials)**						
	**Invasive cervical cancer**	**AIS, irrespective of HPV type**	AIS, vaccine‐matched HPV‐type*	**CIN3, irrespective of HPV type**	CIN3, vaccine‐matched HPV‐type*	**CIN2, irrespective of HPV type**	CIN2, vaccine‐matched HPV‐type*
***Any HPV vaccine vs control*** (Analysis 1.1; Analysis 1.6; Analysis 1.7; Analysis 1.8; Analysis 1.9; Analysis 1.10; Analysis 1.11)							
All populations	No events 17,622 (2)	0.36 (0.16 to 0.78) 35,024 (3)	0.36 (0.17 to 0.78) 35,085 (4)	0.91 (0.66 to 1.27) 17,622 (2)	0.55 (0.43 to 0.70) 18,796 (4)	0.81 (0.70 to 0.94) 17,160 (2)	0.44 (0.34 to 0.57) 18,796 (4)
***Cervarix vs control*** (Analysis 2.5; Analysis 2.6; Analysis 2.7; Analysis 2.8)							
Female < 15 years	‐	‐	‐	‐	‐	‐	‐
Female 15 to 25 years	‐	0.23 (0.07 to 0.81) 17,402 (1)	0.30 (0.08 to 1.09) 17,402 (1)	‐	No events 1113 (1)	‐	0.14 (0.01 to 2.72) 1113 (1)
Female > 25 years	‐	‐	‐	‐	‐	‐	‐
***Gardasil vs control*** (Analysis 3.5; Analysis 3.6; Analysis 3.7; Analysis 3.8; Analysis 3.9; Analysis 3.10; Analysis 3.11)							
Female < 15 years	‐	‐	‐	‐	‐	‐	‐
Female 15 to 25 years	No events 17,622 (2)	0.44 (0.14 to 1.40) 17,622 (2)	0.40 (0.16 to 1.03) 17,683 (3)	0.91 (0.66 to 1.27) 17,622 (2)	0.55 (0.43 to 0.70) 17,683 (3)	0.81 (0.70 to 0.94) 17,160 (2)	0.44 (0.34 to 0.58) 17,683 (3)
Female > 25 years	‐	‐	‐	‐	‐	‐	‐
***Gardasil‐9 vs control:** no trials*							
***Cecolin vs control:** no trials*							
***Gardasil vs Cervarix:** no trials*							
***Gardasil‐9 vs Cervarix:** no trials*							
***Gardasil‐9 vs Gardasil*** (Analysis 8.8; Analysis 8.9; Analysis 8.10)							
Female < 15 years	‐	‐	‐	‐	‐	‐	‐
Female 15 to 25 years	No events 11,386 (1)	‐	‐	‐	‐	‐	***HPV 6,11,16,18:*** 3.00 (0.12 to 73.73) 11,656 (1) ***HPV 31,33,45,52,58:*** 0.03 (0.00 to 0.23) 11,892 (1)
Female > 25 years	‐	‐	‐	‐	‐	‐	‐
*Dose schedule comparisons: no trials*							

**Abbreviations**: AIS: adenocarcinoma in situ; CI: confidence interval; CIN: cervical intraepithelial neoplasm; HPV: human papillomavirus; RR: risk ratio *Vaccine‐matched HPV‐type indicates positive for HPV 16 and/or 18 for Cervarix and Cecolin; HPV 6, 11, 16 and/or 18 for Gardasil; HPV 6, 11, 16, 18, 31, 33, 45, 52 and/or 58 for Gardasil‐9.

Trials comparing Cervarix, Gardasil‐9 or Cecolin with control did not report on invasive cervical cancer. There were no trials comparing different HPV dose schedules that reported on invasive cervical cancer.

###### 1.1.3 Females > 25 years: invasive cervical cancer (no trials)

There were no trials that reported on invasive cervical cancer in females receiving their first dose at > 25 years.

###### 1.1.4 HPV vaccine compared with control in all populations: invasive cervical cancer (standard meta‐analysis)

Two trials that compared Gardasil with control reported no cases of invasive cervical cancer in either group at 48 months follow‐up (RR not estimable, 17,622 participants; Analysis 1.1; Table) (4v FUTURE 2007‐INT; 4v FUTURE II 2007‐INT). Trials comparing Cervarix, Gardasil‐9 or Cecolin with control did not report on invasive cervical cancer.

##### 1.2 HPV‐associated vulval, vaginal, anal or head and neck cancer in females

No trials in females reported on anal cancer or on head and neck cancer.

###### 1.2.1 Females 14 years or younger: HPV‐associated vulval or vaginal cancer (no trials)

There were no trials that reported on HPV‐associated vulval or vaginal cancer in females receiving their first dose at 14 years or younger.

###### 1.2.2 Females 15 to 25 years: HPV‐associated vulval or vaginal cancer (NMA not possible)

Two included trials reported no cases of HPV‐associated vulval or vaginal cancer in females aged 15 to 25 years (4v9v Joura 2015‐INT; 4v FUTURE 2007‐INT); see Table. Consequently, we did not carry out NMA. One of the trials assessed Gardasil versus control (5455 participants, 48 months follow‐up) and one of the trials assessed Gardasil‐9 versus Gardasil (11,509 participants, 72 months follow‐up; Analysis 8.11).

**7:** Vulval and vaginal disease outcomes results

**Population**	**RR (95% CI)** **Participants (trials)**				
	**Invasive vulval or vaginal cancer, irrespective of HPV type**	Invasive vulval or vaginal cancer, vaccine‐matched HPV‐type*	**High‐grade VIN or VaIN, irrespective of HPV type**	High‐grade VIN or VaIN, vaccine‐matched HPV‐type*	High‐grade VIN or VaIN, vaccine‐matched HPV‐type* NMA
***Any HPV vaccine vs control*** (Analysis 1.12; Analysis 1.13; Analysis 1.14; Analysis 1.15)					
All populations	3.01 (0.12 to 73.85) 5455 (1)	No events, 5455 (1)	0.48 (0.33 to 0.70) 33,092 (3)	0.35 (0.10 to 1.24) 36,873 (5)	‐
***Cervarix vs control** (*Analysis 2.9; Analysis 2.10*)*					
Female < 15 years	‐	‐	‐	‐	‐
Female 15 to 25 years	‐	‐	0.43 (0.20 to 0.93) 15,701 (1)	0.28 (0.06 to 1.37) 15,566 (1)	0.28 (0.06 to 1.37)
Female > 25 years	‐	‐	‐	‐	‐
***Gardasil vs control** (*Analysis 3.12; Analysis 3.13; Analysis 3.14*)*					
Female < 15 years	‐	‐	‐	‐	‐
Female 15 to 25 years	3.01 (0.12 to 73.85) 5455 (1)	No events, 5455 (1)	0.49 [0.32 to 0.76] 17,391 (2)	0.21 (0.10 to 0.43) 17,925 (3)	0.21 (0.10 to 0.45)
Female > 25 years	‐	‐	‐	4.98 (0.24 to 103.58) 3382 (1)	‐
***Gardasil‐9 vs control** (only indirect evidence)*					
Female < 15 years	‐	‐	‐	‐	‐
Female 15 to 25 years	‐	‐	‐	‐	0.16 (0.05 to 0.51)
Female > 25 years	‐	‐	‐	‐	‐
***Cecolin vs control:** no trials*					
***Gardasil vs Cervarix** (only indirect evidence)*					
Female < 15 years	‐	‐	‐	‐	‐
Female 15 to 25 years	‐	‐	‐	‐	0.74 (0.13 to 4.17)
Female > 25 years	‐	‐	‐	‐	‐
***Gardasil‐9 vs Cervarix** (only indirect evidence)*					
Female < 15 years	‐	‐	‐	‐	‐
Female 15 to 25 years	‐	‐	‐	‐	0.58 (0.09 to 3.94)
Female > 25 years	‐	‐	‐	‐	‐
***Gardasil‐9 vs Gardasil** (*Analysis 8.11; Analysis 8.12; Analysis 8.13; Analysis 8.14*)*					
Female < 15 years	‐	‐	‐	‐	‐
Female 15 to 25 years	‐	No events, 11,509 (1)	0.88 (0.50 to 1.56) 14,052 (1)	***HPV 6,11,16,18:*** 0.79 (0.36 to 1.73) 14,042 (1) ***HPV 31,33,45,52,58:*** 0.71 (0.23 to 2.25) 14,042 (1)	0.79 (0.35 to 1.75)
Female > 25 years	‐	‐	‐	‐	‐
***Dose schedule comparisons**: no trials*					

**Abbreviations**: CI: confidence interval; HPV: human papillomavirus; NMA: network meta‐analysis; RR: risk ratio; VaIN: vaginal intraepithelial neoplasm; VIN: vulval intraepithelial neoplasm *Vaccine‐matched HPV‐type indicates positive for HPV 16 and/or 18 for Cervarix and Cecolin; HPV 6, 11, 16 and/or 18 for Gardasil; HPV 6, 11, 16, 18, 31, 33, 45, 52 and/or 58 for Gardasil‐9.

Trials comparing Cervarix, Gardasil‐9 or Cecolin with control did not report on HPV‐associated vulval or vaginal cancer. There were no trials comparing different HPV dose schedules that reported on HPV‐associated vulval or vaginal cancer.

###### 1.2.3 Females > 25 years: HPV‐associated vulval or vaginal cancer (no trials)

There were no trials that reported on HPV‐associated vulval or vaginal cancer in females receiving their first dose at > 25 years.

###### 1.2.4 HPV vaccine compared with control in all populations: HPV‐associated vulval or vaginal cancer (single trial)

One trial that compared Gardasil with control reported no cases of vaccine‐type invasive vulval or vaginal cancer in either group at 48 months follow‐up (RR not estimable, 5455 participants; Analysis 1.13; Table) (4v FUTURE 2007‐INT). Trials comparing Cervarix, Gardasil‐9 or Cecolin with control did not report on invasive vulval or vaginal cancer.

##### 1.3 Invasive anal, penile or head and neck cancer in males

No trials in males reported on head and neck cancer.

###### 1.3.1 Males 14 years or younger: invasive anal or penile cancer (no trials)

There were no trials that reported on invasive anal or penile cancer in males receiving their first dose at 14 years or younger.

###### 1.3.2 Males 15 to 25 years: invasive anal or penile cancer (single trial)

One included trial reported no cases of invasive anal or penile cancer in males aged 15 to 25 years at 36 months follow‐up (4v Giuliano 2011‐INT); see Table and Table. Consequently, we did not carry out NMA.

**8:** Anal disease outcomes results

**Population**	**RR (95% CI)** **Participants (trials)**				
	**Invasive anal cancer irrespective of HPV type**	**Vaccine‐matched HPV‐type invasive anal cancer**	**Anal AIS irrespective of HPV type**	**High‐grade AIN irrespective of HPV type**	**Vaccine‐matched HPV‐type high‐grade AIN**
***Cervarix vs control:** no trials*					
***Gardasil vs control*** (Analysis 3.6; Analysis 3.15; Analysis 3.16; Analysis 3.17)					
Male < 15 years	‐	‐	‐	‐	‐
Male 15 to 25 years	No events, 551* (1)	No events, 551* (1)	No events, 551* (1)	0.75 (0.53 to 1.07) 551* (1)	0.46 (0.27 to 0.79) 551* (1)
Male > 25 years	‐	‐	‐	‐	‐
***Gardasil‐9 vs control:** no trials*					
***Cecolin vs control:** no trials*					
*HPV vaccine and dose schedule comparisons: no trials*					

**Abbreviations**: AIN: anal intraepithelial neoplasia; AIS: (anal) adenocarcinoma in situ; CI: confidence interval; RR: risk ratio *Measured in subgroup of men who have sex with men.

**9:** Penile disease outcomes results

**Population**	**RR (95% CI)** **participants (trials)**			
	**Invasive penile cancer irrespective of HPV type**	**Vaccine‐matched HPV‐type invasive penile cancer**	**High‐grade PeIN irrespective of HPV type**	**Vaccine‐matched HPV‐type high‐grade PeIN**
***Cervarix vs control:** no trials*				
***Gardasil vs control** (*Analysis 3.18; Analysis 3.19; Analysis 3.20*)*				
Male < 15 years	‐	‐	‐	‐
Male 15 to 25 years	‐	No events, 3880 (1)	1.00 (0.20 to 4.93) 3880 (1)	1.00 (0.20 to 4.93) 3880 (1)
Male > 25 years	‐	‐	‐	‐
***Gardasil‐9 vs control:** no trials*				
***Cecolin vs control:** no trials*				
*HPV vaccine and dose schedule comparisons: no trials*				

**Abbreviations**: CI: confidence interval; PeIN: penile intraepithelial neoplasia; RR: risk ratio

Trials comparing Cervarix, Gardasil‐9 or Cecolin with control did not report on invasive anal or penile cancer. There were no trials comparing different HPV vaccines or different HPV dose schedules that reported on invasive anal or penile cancer.

###### 1.3.3 Males > 25 years: invasive anal or penile cancer (no trials)

There were no trials that reported on invasive anal or penile cancer in males receiving their first dose at > 25 years.

###### 1.3.4 HPV vaccine compared with control in all populations: invasive anal or penile cancer (single trial)

One trial that compared Gardasil with control reported no cases of invasive anal cancer at 36 months follow‐up in a subgroup of 551 men who have sex with men (MSM) (4v Giuliano 2011‐INT); Table; Table. The same trial reported no cases of invasive penile, perineal or perianal cancer in 3880 participants at 36 months follow‐up; Table; Table. Trials comparing Cervarix, Gardasil‐9 or Cecolin with control did not report on invasive anal or penile cancer.

##### 1.4 High‐grade CIN+

High‐grade CIN+ is a composite outcome that includes the following component outcomes: invasive cervical cancer, cervical AIS, CIN grade 3 and CIN grade 2. Within this outcome we report on the sub‐outcomes CIN3+ irrespective of HPV type, vaccine‐type CIN3+, CIN2+ irrespective of HPV type and vaccine‐type CIN2+.

###### 1.4.1 Females 14 years or younger: high‐grade CIN+ (no trials)

There were no trials that reported on high‐grade CIN+ in females receiving their first dose at 14 years or younger.

###### 1.4.2 Females 15 to 25 years: high‐grade CIN+

####### *CIN3+ irrespective of HPV type (NMA)*

We carried out a NMA of six trials for CIN3+ irrespective of HPV type in females aged 15 to 25 years at the time of the first dose, with follow‐up ranging from 48 to 72 months (2v CVT 2011‐CRI; 2v Konno 2010‐JPN; 2v PATRICIA 2012‐INT; 2v Zhu 2014‐CHNa; 4v FUTURE I/II 2010‐INT (pooled analysis of two trials)); see Figure for the network map and interval plot, and Appendix 3 for the matrix of results (league table) and rank.

**3:**
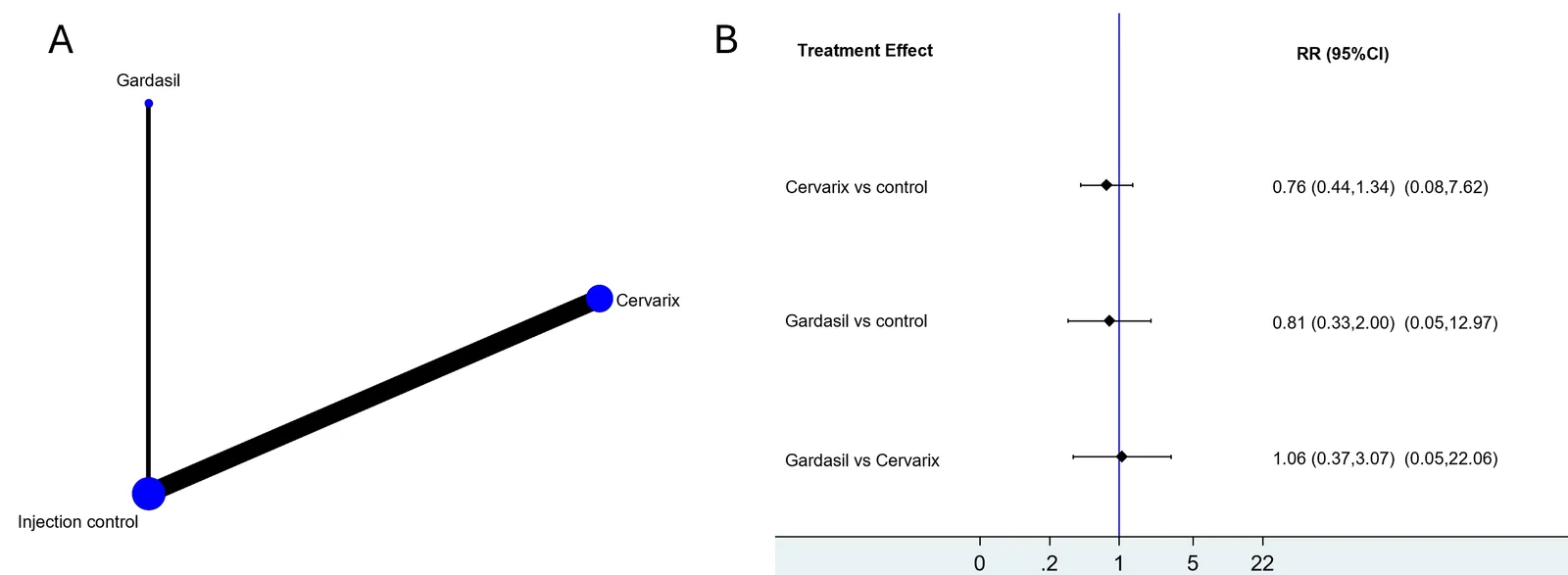
NMA of CIN3+ irrespective of HPV type in females 15 to 25 years old: A) network map, B) interval plot

In the NMA, there was very low‐ to low‐certainty evidence of a reduction in CIN3+ irrespective of HPV type for Cervarix compared with control (very low‐certainty evidence, 6 fewer per 1000, 95% CI 13 fewer to 8 more; RR 0.76, 95% CI 0.44 to 1.34) and Gardasil compared with control (low‐certainty evidence, 4 fewer per 1000, 95% CI 16 fewer to 23 more; RR 0.81, 95% CI 0.33 to 2.00), and little to no difference with Gardasil compared with Cervarix (low‐certainty evidence, 1 more per 1000, 95% CI 7 fewer to 23 more; RR 1.06, 95% CI 0.37 to 3.07). There was no evidence on CIN3+ irrespective of HPV type from trials assessing Gardasil‐9 or Cecolin, or from trials assessing different dose schedules. See Table for standard meta‐analysis and NMA results and Table.

**10:** Cervical disease composite outcomes results

**Population**	**RR (95% CI)** **Participants (trials)**							
	**CIN3+, irrespective of HPV type** ***NMA***	**CIN3+, irrespective of HPV type**	CIN3+, vaccine‐matched HPV‐type* ***NMA***	CIN3+, vaccine‐matched HPV‐type*	**CIN2+, irrespective of HPV type** ***NMA***	**CIN2+, irrespective of HPV type**	CIN2+, vaccine‐matched HPV‐type* ***NMA***	CIN2+, vaccine‐matched HPV‐type*
***Any HPV vaccine vs control*** (Analysis 1.2; Analysis 1.3; Analysis 1.4; Analysis 1.5)								
All populations	‐	0.78 (0.55 to 1.12) 43,901 (6)	‐	0.54 (0.44 to 0.65) 35,655 (4)	‐	0.79 (0.65 to 0.96) 55,911 (8)	‐	0.50 (0.39 to 0.65) 59,690 (10)
***Cervarix vs control*** (Analysis 2.1; Analysis 2.2; Analysis 2.3; Analysis 2.4)								
Female < 15 years	‐	‐	‐	‐	‐	‐	‐	‐
Female 15 to 25 years	0.76 (0.44 to 1.34)	0.76 (0.41 to 1.41) 26,741 (4)	0.55 (0.39 to 0.77)	0.54 (0.39 to 0.76) 18,495 (2)	0.61 (0.39 to 0.95)	0.63 (0.44 to 0.90) 29,464 (4)	0.34 (0.14 to 0.81)	0.33 (0.21 to 0.50) 30,302 (5)
Female > 25 years	‐	‐	‐	‐	‐	0.95 (0.73 to 1.24) 5468 (1)	0.71 (0.45 to 1.11)	0.71 (0.45 to 1.11) 5477 (1)
***Gardasil vs control*** (Analysis 3.1; Analysis 3.2; Analysis 3.4; Analysis 3.3)								
Female < 15 years	‐	‐	‐	‐	‐	‐	‐	‐
Female 15 to 25 years	0.81 (0.33 to 2.00)	0.81 (0.69 to 0.96) 17,160 (2)	0.54 (0.43 to 0.68)	0.54 (0.43 to 0.68) 17,160 (2)	0.81 (0.38 to 1.73)	0.81 (0.72 to 0.92) 17,160 (2)	0.50 (0.33 to 0.76)	0.50 (0.42 to 0.59) 17,160 (2)
Female > 25 years	‐	‐	‐	‐	‐	1.21 (0.84 to 1.75) 3819 (1)	0.74 (0.50 to 1.09)	0.74 (0.50 to 1.09) 6778 (2)
***Gardasil‐9 vs control:** no direct or indirect evidence*								
Female < 15 years	‐	‐	‐	‐	‐	‐	‐	‐
Female 15 to 25 years	‐	‐	0.03 (0.00 to 0.55)	‐	0.82 (0.28 to 2.39)	‐	0.49 (0.27 to 0.89)	‐
Female > 25 years	‐	‐	‐	‐	‐	‐	‐	‐
***Cecolin vs control:** no direct or indirect evidence*								
***Gardasil vs Cervarix*** (indirect evidence only)								
Female < 15 years	‐	‐	‐	‐	‐	‐	‐	‐
Female 15 to 25 years	1.06 (0.37 to 3.07)	‐	0.98 (0.65 to 1.49)	‐	1.32 (0.55 to 3.18)	‐	1.47 (0.56 to 3.90)	‐
Female > 25 years	‐	‐	‐	‐	‐	‐	1.04 (0.57 to 1.89)	‐
***Gardasil‐9 vs Cervarix***(indirect evidence only)								
Female < 15 years	‐	‐	‐	‐	‐	‐	‐	‐
Female 15 to 25 years	‐	‐	0.06 (0.00 to 1.03)	‐	1.34 (0.42 to 4.26)	‐	1.46 (0.51 to 4.21)	‐
Female > 25 years	‐	‐	‐	‐	‐	‐	‐	‐
***Gardasil‐9 vs Gardasil***(Analysis 8.1; Analysis 8.2; Analysis 8.4; Analysis 8.5; Analysis 8.6)								
Female < 15 years	‐	‐	‐	‐	‐	‐	‐	‐
Female 15 to 25 years	‐	‐	0.06 (0.00 to 1.02)	***HPV 6,11,16,18:*** 0.33 (0.01 to 8.19) 11,656 (1) ***HPV 31,33,45,52,58:*** 0.07 (0.00 to 0.17) 11,892 (1)	1.01 (0.47 to 2.17)	1.01 (0.87 to 1.17) 13,754 (1)	0.99 (0.65 to 1.52)	***HPV 6,11,16,18:*** 1.20 (0.95 to 1.53) 13,754 (1) ***HPV 31,33,45,52,58:*** 0.83 (0.66 to 1.04) 13,754 (1)
Female > 25 years	‐	‐	‐	‐	‐	‐	‐	‐
***Dose schedule comparisons**: no direct or indirect evidence*								

**Abbreviations**: CI: confidence interval; CIN: cervical intraepithelial neoplasm; HPV: human papillomavirus; NMA: network meta‐analysis; RR: risk ratio *Vaccine‐matched HPV‐type indicates positive for HPV 16 and/or 18 for Cervarix and Cecolin; HPV 6, 11, 16 and/or 18 for Gardasil; HPV 6, 11, 16, 18, 31, 33, 45, 52 and/or 58 for Gardasil‐9.

In the NMA, Cervarix was found to have the highest probability (49.7%) of being the best vaccine to reduce the risk of developing CIN3+ irrespective of HPV type, followed by Gardasil (44.5%).

####### *Vaccine‐type CIN3+ (NMA)*

We carried out a NMA of five trials for vaccine‐type CIN3+ in females aged 15 to 25 years at the time of the first dose, with follow‐up ranging from 27 to 72 months (2v Harper 2004‐BRA/NA; 2v PATRICIA 2012‐INT; 4v FUTURE I/II 2010‐INT (pooled analysis of two trials); 4v9v Joura 2015‐INT); see Figure for the network map and interval plot, and Appendix 3 for the matrix of results (league table) and rank.

**4:**
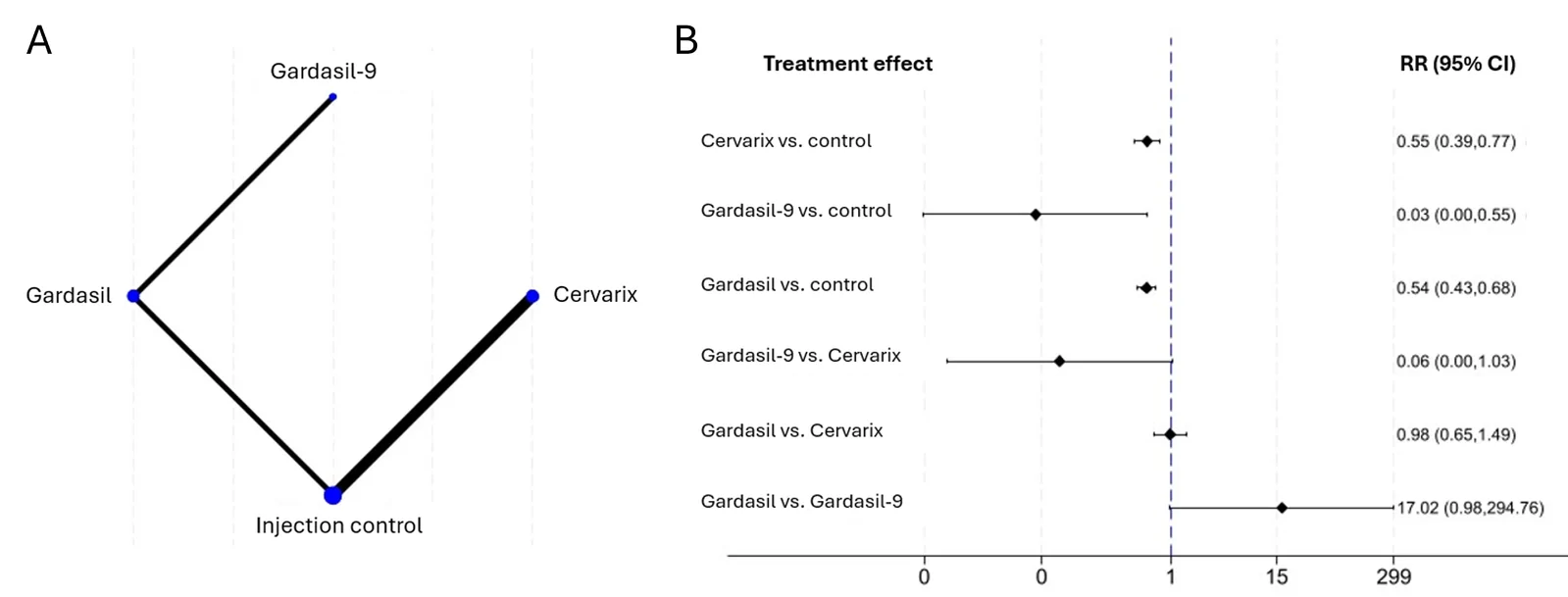
NMA of CIN3+ vaccine‐matched HPV‐type in females 15 to 25 years old: A) network map, B) interval plot

In the NMA, there was moderate‐ to high‐certainty evidence of reduced vaccine‐type CIN3+ for Cervarix compared with control (high‐certainty evidence, 7 fewer per 1000, 95% CI 4 to 10 fewer; RR 0.55, 95% CI 0.39 to 0.77), Gardasil compared with control (high‐certainty evidence, 8 fewer per 1000, 95% CI 5 to 9 fewer; RR 0.54, 95% CI 0.43 to 0.68) and Gardasil‐9 compared with control (moderate‐certainty evidence, 16 fewer per 1000, 95% CI 0 to 16 fewer; RR 0.03, 95% CI 0.00 to 0.55). There was moderate‐certainty evidence of little to no difference for Gardasil compared with Cervarix (0 more per 1000, 95% CI 4 fewer to 5 more; RR 0.98, 95% CI 0.65 to 1.49) and moderate‐certainty evidence of reduced vaccine‐type CIN3+ for Gardasil‐9 compared with Cervarix (10 fewer per 1000, 95% CI 0 to 10 fewer; RR 0.06, 95% CI 0.00 to 1.03) and Gardasil‐9 compared with Gardasil (7 fewer per 1000, 95% CI 0 to 8 fewer; RR 0.06, 95% CI 0.00 to 1.02). There was no evidence on vaccine‐type CIN3+ from trials assessing Cecolin or from trials assessing different dose schedules. See Table for standard meta‐analysis and NMA results and Table.

In the NMA, Gardasil‐9 was found to have the highest probability (97%) of being the best vaccine to reduce the risk of developing vaccine‐type CIN3+, followed by Cervarix (1.5%) and Gardasil (1.5%).

####### *CIN2+ irrespective of HPV type (NMA)*

We carried out a NMA of seven trials for CIN2+ irrespective of HPV type in females aged 15 to 25 years at the time of the first dose at up to 72 months follow‐up (2v CVT 2011‐CRI; 2v Konno 2010‐JPN; 2v PATRICIA 2012‐INT; 2v Zhu 2014‐CHNa; 4v FUTURE I/II 2010‐INT (pooled analysis of two trials); 4v9v Joura 2015‐INT); see Figure for the network map and interval plot, and Appendix 3 for the matrix of results (league table) and rank.

**5:**
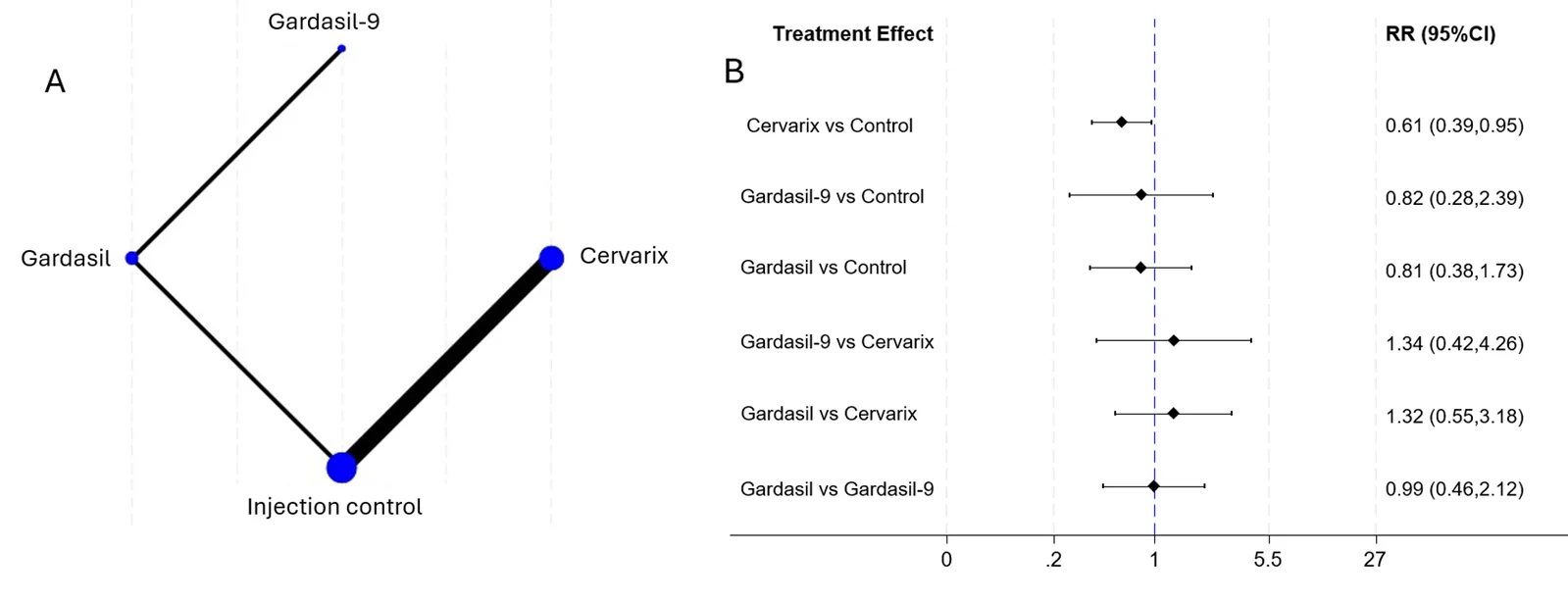
NMA of CIN2+ irrespective of HPV type in females 15 to 25 years old: A) network map, B) interval plot

In the NMA, there was low‐certainty evidence of reduced CIN2+ irrespective of HPV type for Cervarix compared with control (low‐certainty evidence, 16 fewer per 1000, 95% CI 2 to 25 fewer; RR 0.61, 95% CI 0.39 to 0.95) and Gardasil compared with control (low‐certainty evidence, 11 fewer per 1000, 95% CI 37 fewer to 44 more; RR 0.81, 95% CI 0.38 to 1.73). There was low‐certainty evidence of little to no difference in CIN2+ irrespective of HPV type for Gardasil‐9 compared with Gardasil (low‐certainty evidence, 0 fewer per 1000, 95% CI 27 fewer to 59 more; RR 1.01, 95% CI 0.47 to 2.16). The evidence is very uncertain for Gardasil‐9 compared with control (very low‐certainty evidence, 9 fewer per 1000, 95% CI 35 fewer to 67 more; RR 0.82, 95% CI 0.28 to 2.39), Gardasil compared with Cervarix (very low‐certainty evidence, 9 more per 1000, 95% CI 13 fewer to 61 more; RR 1.32, 95% CI 0.55 to 3.18) and for Gardasil‐9 compared with Cervarix (very low‐certainty evidence, 10 more per 1000, 95% CI 16 fewer to 92 more; RR 1.34, 95% CI 0.42 to 4.26). There was no evidence on CIN2+ irrespective of HPV type from trials assessing Cecolin or from trials assessing different dose schedules. See Table for standard meta‐analysis and NMA results and Table.

In the NMA, Cervarix was found to have the highest probability (61.6%) of being the best vaccine to reduce the risk of developing CIN2+ irrespective of HPV type, followed by Gardasil‐9 (24.3%) and Gardasil (13.9%).

####### *Vaccine‐type CIN2+ (NMA)*

We carried out a NMA of eight trials for vaccine‐type CIN2+ in females aged 15 to 25 years at the time of the first dose with follow‐up ranging from 48 to 72 months (2v CVT 2011‐CRI; 2v Harper 2004‐BRA/NA; 2v Konno 2010‐JPN; 2v PATRICIA 2012‐INT; 2v Zhu 2014‐CHNa; 4v FUTURE I/II 2010‐INT (pooled analysis of two trials); 4v9v Joura 2015‐INT); see Figure for the network map and interval plot, and Appendix 3 for the matrix of results (league table) and rank.

**6:**
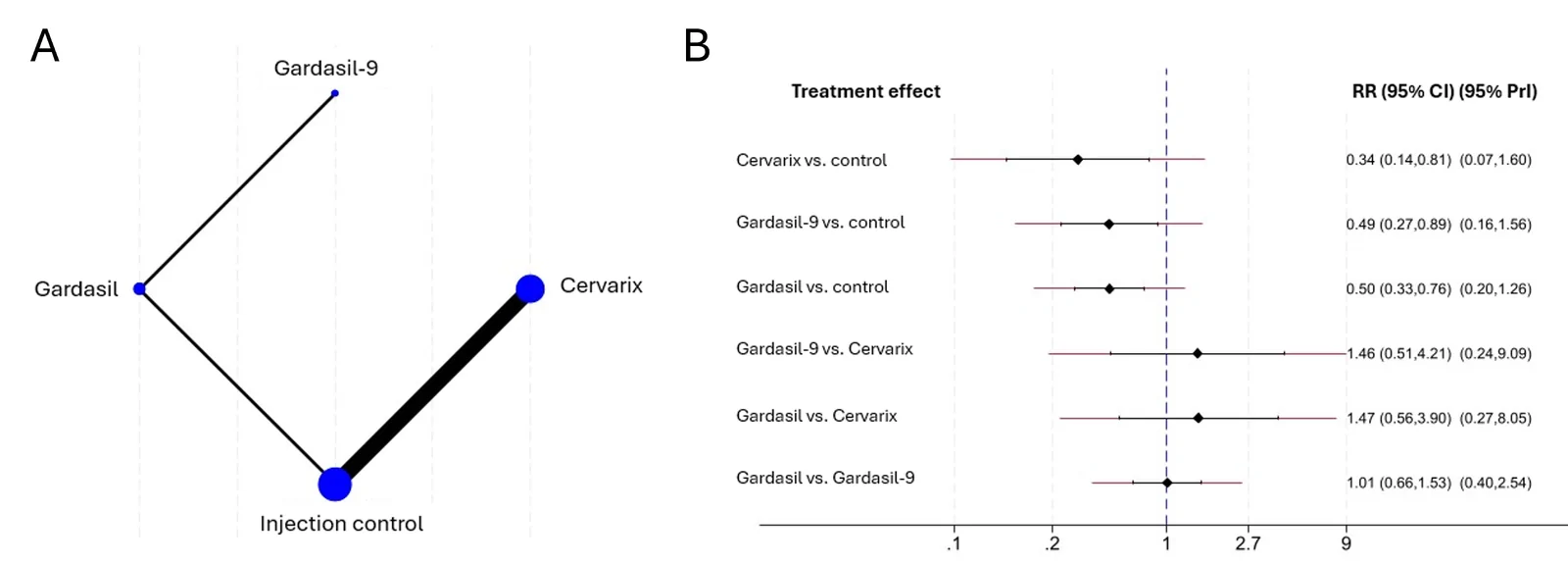
NMA of CIN2+ vaccine‐matched HPV‐type in females 15 to 25 years old: A) network map, B) interval plot

In the NMA, there was moderate‐ to high‐certainty evidence of reduced vaccine‐type CIN2+ for Cervarix compared with control (moderate‐certainty evidence, 21 fewer per 1000, 95% CI 6 to 27 fewer; RR 0.34, 95% CI 0.14 to 0.81), Gardasil compared with control (high‐certainty evidence, 16 fewer per 1000, 95% CI 8 to 21 fewer; RR 0.50, 95% CI 0.33 to 0.76) and Gardasil‐9 compared with control (moderate‐certainty evidence, 16 fewer per 1000, 95% CI 3 to 23 fewer; RR 0.34, 95% CI 0.14 to 0.81). There was low‐certainty evidence of little to no difference in vaccine‐type CIN2+ for Gardasil compared with Cervarix (low‐certainty evidence, 3 more per 1000, 95% CI 3 fewer to 19 more; RR 1.47, 95% CI 0.56 to 3.90), Gardasil‐9 compared with Cervarix (low‐certainty evidence, 3 more per 1000, 95% CI 3 fewer to 21 more; RR 1.46, 95% CI 0.51 to 4.21) and for Gardasil‐9 compared with Gardasil (low‐certainty evidence, 0 fewer per 1000, 95% CI 11 fewer to 16 more; RR 0.99, 95% CI 0.65 to 1.52). There was no evidence on CIN2+ irrespective of HPV type from trials assessing Cecolin or from trials assessing different dose schedules. See Table for standard meta‐analysis and NMA results and Table.

In the NMA, Cervarix was found to have the highest probability (72.6%) of being the best vaccine to reduce the risk of developing vaccine‐type CIN2+, followed by Gardasil‐9 (17.0%) and Gardasil (10.4%).

###### 1.4.3 Females > 25 years: high‐grade CIN+

####### *CIN3+ irrespective of HPV type (no trials)*

There were no trials that reported on CIN3+ irrespective of HPV type in females receiving their first dose at > 25 years.

####### *Vaccine‐type CIN3+ (no trials)*

There were no trials that reported on vaccine‐type CIN3+ in females receiving their first dose at > 25 years.

####### *CIN2+ irrespective of HPV type (no NMA)*

Two trials reported on CIN2+ irrespective of HPV type in females that received their first dose at > 25 years of age (2v VIVIANE 2014‐INT; 4v FUTURE III 2009‐INT). Consequently, we did not carry out NMA. The studies found little to no difference between Cervarix and control at 84 months follow‐up (moderate‐certainty evidence, 2 fewer per 1000, 95% CI 11 fewer to 9 more; RR 0.95, 95% CI 0.73 to 1.24, 5468 participants; Analysis 2.3), or between Gardasil and control at 48 months follow‐up (moderate‐certainty evidence, 6 more per 1000, 95% CI 4 fewer to 20 more; RR 1.21, 95% CI 0.84 to 1.75, 3819 participants; Analysis 3.3). There was no evidence on CIN2+ irrespective of HPV type from trials assessing Gardasil‐9, Cecolin or from trials assessing different dose schedules. See Table for results and Table.

####### *Vaccine‐type CIN2+ (NMA)*

We carried out a NMA of three trials for vaccine‐type CIN2+ in females that received their first dose at > 25 years of age with follow‐up ranging from 48 to 78 months (2v VIVIANE 2014‐INT; 4v FUTURE III 2009‐INT; 4v Wei 2019‐CHN); see Figure for the network map and interval plot, and Appendix 3 for the matrix of results (league table) and rank.

**7:**
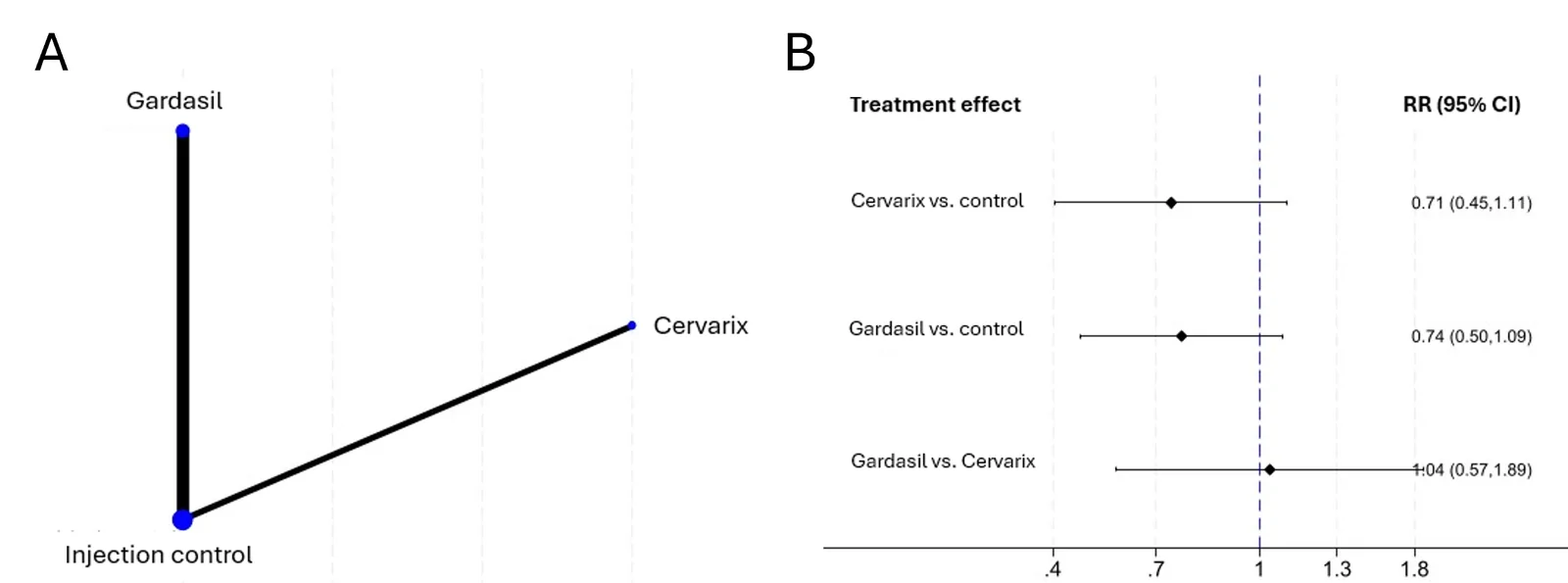
NMA of CIN2+ vaccine‐matched HPV‐type in females 25 years and older: A) network map, B) interval plot

In the NMA, there was moderate‐ to high‐certainty evidence of little to no difference between Cervarix and control (high‐certainty evidence, 5 fewer per 1000, 95% CI 9 fewer to 2 more; RR 0.71, 95% CI 0.45 to 1.11), between Gardasil and control (moderate‐certainty evidence, 4 fewer per 1000, 95% CI 8 fewer to 2 more; RR 0.74, 95% CI 0.50 to 1.09) or between Gardasil and Cervarix (moderate‐certainty evidence, 0 fewer per 1000, 95% CI 5 fewer to 10 more; RR 1.04, 95% CI 0.57 to 1.89). There was no evidence on vaccine‐type CIN2+ from trials assessing Gardasil‐9, Cecolin or from trials assessing different dose schedules. See Table for results and Table.

In the NMA, Cervarix was found to have the highest probability (54.2%) of being the best vaccine to reduce the risk of developing vaccine‐type CIN2+, followed by Gardasil (45.4%).

###### 1.4.4 HPV vaccine compared with control in all populations: high‐grade CIN+ (standard meta‐analysis)

####### *CIN3+ irrespective of HPV type*

Six trials reported on CIN3+ irrespective of HPV type and compared Cervarix or Gardasil with control (2v CVT 2011‐CRI; 2v Konno 2010‐JPN; 2v PATRICIA 2012‐INT; 2v Zhu 2014‐CHNa; 4v FUTURE I/II 2010‐INT (combining results for two trials)). The trials reported 5 fewer cases per 1000 participants in the HPV vaccine group compared with the control group rate of 23 per 1000 at up to 72 months follow‐up, but confidence intervals were also compatible with no difference (95% CI 11 fewer to 3 more per 1000, low‐certainty evidence; RR 0.78, 95% CI 0.55 to 1.12, I^2^ = 74%, 43,901 participants; Analysis 1.2; Table). One study had an effect estimate in the opposite direction to the other studies (2v Zhu 2014‐CHNa), which could have contributed to the heterogeneity. We did not detect any clinical or methodological characteristics that might explain this heterogeneity. Trials comparing Gardasil‐9 or Cecolin with control did not report on CIN3+ irrespective of HPV type.

####### *Vaccine‐type CIN3+*

Four trials reported on vaccine‐type CIN3+ and compared Cervarix or Gardasil with control (2v Harper 2004‐BRA/NA; 2v PATRICIA 2012‐INT; 4v FUTURE I/II 2010‐INT (combining results for two trials)). The trials reported 8 fewer cases per 1000 participants in the HPV vaccine group compared with the control group rate of 16 per 1000 at up to 48 months follow‐up (95% CI 6 to 9 fewer, moderate‐certainty evidence; RR 0.54, 95% CI 0.44 to 0.65, I^2^ = 0%, 35,655 participants; Analysis 1.3; Table). Trials comparing Gardasil‐9 or Cecolin with control did not report on vaccine‐type CIN3+.

####### *CIN2+ irrespective of HPV type*

Eight trials reported on CIN2+ irrespective of HPV type and compared Cervarix or Gardasil with control (2v CVT 2011‐CRI; 2v Konno 2010‐JPN; 2v PATRICIA 2012‐INT; 2v Zhu 2014‐CHNa; 4v FUTURE I/II 2010‐INT (combining results for two trials); 2v VIVIANE 2014‐INT; 4v FUTURE III 2009‐INT). The trials reported 10 fewer cases per 1000 participants (95% CI 2 to 16 fewer) in the HPV vaccine group compared with the control group rate of 46 per 1000 at up to 72 months follow‐up (moderate‐certainty evidence; RR 0.79, 95% CI 0.65 to 0.96, I^2^ = 76%, 55,911 participants; Analysis 1.4; Table). Significant subgroup differences were detected between the 15 to 25 years and over 25 years subgroups (RR 0.70 (95% CI 0.56 to 0.88) versus RR 1.04 (95% CI 0.83 to 1.30); test for subgroup differences I^2^ = 83%, P = 0.02), which may explain the heterogeneity in the overall result. Trials comparing Gardasil‐9 or Cecolin with control did not report on CIN2+ irrespective of HPV type.

####### *Vaccine‐type CIN2+*

Ten trials reported on vaccine‐type CIN2+ and compared Cervarix or Gardasil with control (2v CVT 2011‐CRI; 2v Harper 2004‐BRA/NA; 2v Konno 2010‐JPN; 2v PATRICIA 2012‐INT; 2v Zhu 2014‐CHNa; 4v FUTURE I/II 2010‐INT (combining results for two trials); 2v VIVIANE 2014‐INT; 4v FUTURE III 2009‐INT; 4v Wei 2019‐CHN). The trials reported 13 fewer cases per 1000 participants (95% CI 9 to 15 fewer) in the HPV vaccine groups compared with the control group rate of 25 per 1000 at up to 78 months follow‐up (moderate‐certainty evidence; RR 0.50, 95% CI 0.39 to 0.65, I^2^ = 54%, 59,717 participants; Analysis 1.5; Table). Significant subgroup differences were detected between the 15 to 25 years and over 25 years subgroups (RR 0.40 (95% CI 0.30 to 0.54) versus RR 0.73 (95% CI 0.54 to 0.98); test for subgroup differences I^2^ = 87.5%, P = 0.005), which may explain the heterogeneity in the overall result. Trials comparing Gardasil‐9 or Cecolin with control did not report on vaccine‐type CIN2+.

####### Subgroup and sensitivity analyses

*CIN3+ irrespective of HPV type*

We did not carry out subgroup analyses since fewer than 10 studies were included for this outcome.

Sensitivity analysis using intention‐to‐treat populations showed similar results (RR 0.78, 95% CI 0.55 to 1.11; 6 trials, 50,823 participants; analysis not shown), as did sensitivity analysis excluding pooled analyses (RR 0.76, 95% CI 0.41 to 1.41; 4 trials, 26,741 participants; analysis not shown). We did not carry out the remaining planned sensitivity analyses for this outcome because events were not rare (< 1%), risk of bias was not high for any of the included studies, there were no trials reported in abstracts only and there were no cluster trials in the meta‐analysis.

*Vaccine‐type CIN3+*

We did not carry out subgroup analyses since fewer than 10 studies were included for this outcome.

Sensitivity analysis using intention‐to‐treat populations showed similar results (RR 0.54, 95% CI 0.44 to 0.65; 4 trials, 37,379 participants; analysis not shown), as did sensitivity analysis excluding pooled analyses (RR 0.54, 95% CI 0.39 to 0.76; 2 trials, 18,495 participants; analysis not shown). We did not carry out the remaining planned sensitivity analyses for this outcome because events were not rare (< 1%), risk of bias was not high for any of the included studies, there were no trials reported in abstracts only and there were no cluster trials in the meta‐analysis.

*CIN2+ irrespective of HPV type*

We did not carry out subgroup analyses since fewer than 10 studies were included for this outcome.

Sensitivity analysis using intention‐to‐treat populations showed similar results (RR 0.79, 95% CI 0.64 to 0.96; 8 trials, 60,389 participants; analysis not shown), as did sensitivity analysis excluding pooled analyses (RR 0.76, 95% CI 0.58 to 1.01; 6 trials, 38,751 participants; analysis not shown). We did not carry out the remaining planned sensitivity analyses for this outcome because events were not rare (< 1%), risk of bias was not high for any of the included studies, there were no trials reported in abstracts only and there were no cluster trials in the meta‐analysis.

*Vaccine‐type CIN2+*

No notable subgroup differences were detected for country income setting (P = 0.27, I² = 23.4%, analysis not shown), presence of national screening programme (P = 0.20, I² = 40.4%, analysis not shown) or type of funding source (P = 0.13, I² = 57.4%, analysis not shown).

The remaining planned subgroup analyses could not be carried out. All trials reporting on this outcome had conflicts of interest and a similar interval duration between doses (month 0, 1, 6 schedule or month 0, 2, 6 schedule). For history of sexual activity and HPV DNA status at baseline, sufficient data were not reported in the trials.

Sensitivity analysis using intention‐to‐treat populations showed similar results (RR 0.50, 95% CI 0.39 to 0.65, 10 trials, 64,496 participants; analysis not shown), as did sensitivity analysis excluding pooled analyses (RR 0.48, 95% CI 0.33 to 0.70; 8 trials, 42,557 participants; analysis not shown). We did not carry out the remaining planned sensitivity analyses for this outcome because events were not rare (< 1%), risk of bias was not high for any of the included studies, there were no trials reported in abstracts only and there were no cluster trials in the meta‐analysis.

##### 1.5 Vaccine‐type high‐grade VIN or VaIN

Vaccine‐type high‐grade VIN or VaIN is a composite outcome that includes the following component outcomes: VIN grade 2, VIN grade 3, VaIN grade 2 and VaIN grade 3. High‐grade VIN or VaIN irrespective of HPV type is an important outcome reported in section 2.2 below.

###### 1.5.1 Females 14 years or younger: vaccine‐type high‐grade VIN or VaIN (no trials)

There were no trials that reported on vaccine‐type high‐grade VIN or VaIN in females receiving their first dose at 14 years or younger.

###### 1.5.2 Females 15 to 25 years: vaccine‐type high‐grade VIN or VaIN (NMA)

A NMA of five trials was carried out for vaccine‐type high‐grade VIN or VaIN in females aged 15 to 25 years at the time of the first dose at up to 72 months follow‐up (2v PATRICIA 2012‐INT; 4v9v Joura 2015‐INT; 4v Villa/FUTURE I/II 2009‐INT (pooled analysis of three trials)); see Figure for the network map and interval plot, and Appendix 3 for the matrix of results (league table) and rank.

**8:**
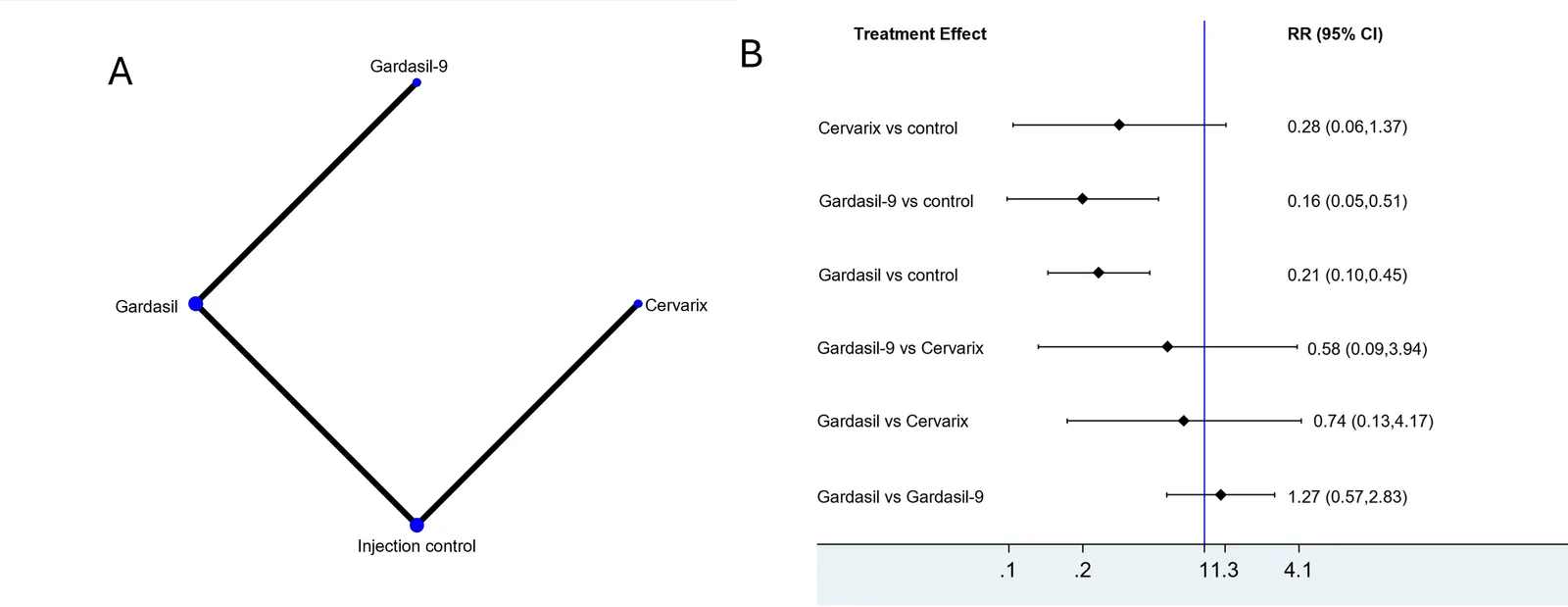
NMA of vaccine‐matched HPV‐type high‐grade VIN or VaIN in females 15 to 25 years old: A) network map, B) interval plot

In the NMA, there was moderate‐certainty evidence of a reduction in vaccine‐type high‐grade VIN or VaIN for Gardasil compared with control (RR 0.21, 95% CI 0.10 to 0.45) and Gardasil‐9 compared with control (RR 0.16, 95% CI 0.05 to 0.51). Moderate‐certainty evidence of little to no difference in vaccine‐type high‐grade VIN or VaIN was found in the NMA for Cervarix compared with control (RR 0.28, 95% CI 0.06 to 1.37), Gardasil compared with Cervarix (RR 0.74, 95% CI 0.13 to 4.17), Gardasil‐9 compared with Cervarix (RR 0.58, 95% CI 0.09 to 3.94) and for Gardasil‐9 compared with Gardasil (RR 0.79, 95% CI 0.35 to 1.75). There was no evidence on vaccine‐type high‐grade VIN or VaIN from trials assessing Cecolin or from trials assessing different dose schedules. See Table for standard meta‐analysis and NMA results and Table.

In the NMA, Gardasil‐9 was found to have the highest probability (56.2%) of being the best vaccine to reduce the risk of developing vaccine‐type high‐grade VIN or VaIN, followed by Cervarix (25.8%) and Gardasil (18.0%).

###### 1.5.3 Females > 25 years: vaccine‐type high‐grade VIN or VaIN (single trial)

One trial reported on vaccine‐type high‐grade VIN or VaIN in females that received their first dose at > 25 years of age (4v FUTURE III 2009‐INT). Consequently, NMA was not carried out. The study compared Gardasil and control at 48 months follow‐up, but the evidence was very uncertain due to very wide confidence intervals (low‐certainty evidence, RR 4.98, 95% CI 0.24 to 103.58; 3382 participants; Analysis 3.14). See Table for results and Table.

###### 1.5.4 HPV vaccine compared with control in all populations: vaccine‐type high‐grade VIN or VaIN (standard meta‐analysis)

Five trials reported on vaccine‐type high‐grade VIN or VaIN and compared Cervarix or Gardasil with control (2v PATRICIA 2012‐INT; 4v Villa/FUTURE I/II 2009‐INT (combining results for three trials); 4v FUTURE III 2009‐INT). The trials reported two fewer cases per 1000 participants in the HPV vaccine groups compared with the control group rate of three per 1000 at up to 48 months follow‐up, but confidence intervals were also compatible with no difference (95% CI 2 fewer to 1 more, moderate‐certainty evidence; RR 0.35, 95% CI 0.10 to 1.24; I^2^ = 50%; 5 trials, 36,873 participants; Analysis 1.15; Table). Trials comparing Gardasil‐9 or Cecolin with control did not report on vaccine‐type high‐grade VIN or VaIN.

####### Subgroup and sensitivity analyses

We did not carry out subgroup analyses since fewer than 10 studies were included for this outcome.

Sensitivity analysis using intention‐to‐treat populations showed similar results (RR 0.36, 95% CI 0.10 to 1.25; 5 trials, 40,637 participants; analysis not shown). Sensitivity analysis excluding pooled analyses included far fewer participants and events, leading to an estimate with very wide 95% CIs (RR 0.88, 95% CI 0.06 to 13.90; 2 trials, 18,948 participants; analysis not shown). We did not carry out the remaining planned sensitivity analyses for this outcome because events were not rare (< 1%), risk of bias was not high for any of the included studies, there were no trials reported in abstracts only and there were no cluster trials in the meta‐analysis.

##### 1.6 High‐grade AIN or PeIN irrespective of HPV type

High‐grade AIN or PeIN irrespective of HPV type is a composite outcome that includes the following component outcomes: AIN grade 2 irrespective of HPV type, AIN grade 3 irrespective of HPV type, PeIN grade 2 irrespective of HPV type and PeIN grade 3 irrespective of HPV type.

###### 1.6.1 Males 14 years or younger: high‐grade AIN or PeIN irrespective of HPV type (no trials)

There were no trials that reported on high‐grade AIN or PeIN irrespective of HPV type in males receiving their first dose at 14 years or younger.

###### 1.6.2 Males 15 to 25 years: high‐grade AIN or PeIN irrespective of HPV type (single trial)

One trial reported on high‐grade AIN irrespective of HPV type and high‐grade PeIN irrespective of HPV type in males that received their first dose at 15 to 25 years of age (4v Giuliano 2011‐INT). Consequently, we did not carry out NMA. The trial reported 54 fewer cases per 1000 participants of high‐grade AIN irrespective of HPV type in the Gardasil group compared with the control group rate of 214 per 1000 at 36 months follow‐up in a subgroup of 551 MSM, but confidence intervals also included no effect (95% CI 101 fewer to 15 more; low‐certainty evidence, RR 0.75, 95% CI 0.53 to 1.07; Analysis 3.16). The trial reported no difference per 1000 participants of high‐grade PeIN irrespective of HPV type in the Gardasil group compared with the control group rate of 2 per 1000 at 36 months follow‐up (95% CI 1 fewer to 6 more; low‐certainty evidence, RR 1.00, 95% CI 0.20 to 4.93; 3880 participants; Analysis 3.19). See Table for results and Table.

Trials comparing Cervarix, Gardasil‐9 or Cecolin with control did not report on high‐grade AIN or PeIN irrespective of HPV type. There were no trials comparing different HPV vaccines or different HPV dose schedules that reported on high‐grade AIN or PeIN irrespective of HPV type.

###### 1.6.3 Males > 25 years: high‐grade AIN or PeIN irrespective of HPV type (no trials)

There were no trials that reported on AIN or PeIN irrespective of HPV type in males receiving their first dose at > 25 years.

###### 1.6.4 HPV vaccine compared with control in all populations: high‐grade AIN or PeIN irrespective of HPV type (single trial)

Only one trial reported on this outcome; see section 1.6.2 above.

##### 1.7 Vaccine‐type high‐grade AIN or PeN

Vaccine‐type high‐grade AIN or PeIN is a composite outcome that includes the following component outcomes: vaccine‐type AIN grade 2, vaccine‐type AIN grade 3, vaccine‐type PeIN grade 2 and vaccine‐type PeIN grade 3.

###### 1.7.1 Males 14 years or younger: vaccine‐type high‐grade AIN or PeIN (no trials)

There were no trials that reported on vaccine‐type high‐grade AIN or PeIN in males receiving their first dose at 14 years or younger.

###### 1.7.2 Males 15 to 25 years: vaccine‐type high‐grade AIN or PeIN (single trial)

One trial reported on vaccine‐type high‐grade AIN and vaccine‐type high‐grade PeIN in males that received their first dose at 15 to 25 years of age (4v Giuliano 2011‐INT). Consequently, we did not carry out NMA. The trial reported 76 fewer cases per 1000 participants (95% CI 30 to 103 fewer) of vaccine‐type high‐grade AIN in the Gardasil group compared with the control group rate of 141 per 1000 at 36 months follow‐up in a subgroup of 551 MSM (low‐certainty evidence, RR 0.46, 95% CI 0.27 to 0.79; Analysis 3.17). The trial reported no difference per 1000 participants of vaccine‐type high‐grade PeIN in the Gardasil group compared with the control group rate of 2 per 1000 at 36 months follow‐up (95% CI 1 fewer to 6 more; low‐certainty evidence, RR 1.00, 95% CI 0.20 to 4.93; 3880 participants; Analysis 3.20). See Table for results and Table.

Trials comparing Cervarix, Gardasil‐9 or Cecolin with control did not report on vaccine‐type high‐grade AIN or PeIN. There were no trials comparing different HPV vaccines or different HPV dose schedules that reported on vaccine‐type high‐grade AIN or PeIN.

###### 1.7.3 Males > 25 years: vaccine‐type high‐grade AIN or PeIN (no trials)

There were no trials that reported on vaccine‐type high‐grade AIN or PeIN in males receiving their first dose at > 25 years.

###### 1.7.4 HPV vaccine compared with control in all populations: vaccine‐type high‐grade AIN or PeIN (single trial)

Only one trial reported on this outcome; see section 1.7.2 above.

##### 1.8 Serious adverse events

We collected the composite outcome serious adverse events (that are fatal, life‐threatening, result in hospitalisation, persistent or significant disability/incapacity, congenital anomaly/birth defect, or require intervention to prevent permanent impairment or damage, or as defined in the studies).

Serious adverse events that were considered by trial investigators to be possibly or probably related to vaccination are reported in Table.

**11:** Serious adverse events assessed as probably or possibly related to intervention

**Study**	**Follow‐up**	**Related SAEs**		**Type of assessment**
		**Intervention**	**Control**	
2v CVT 2011‐CRI	48 months	**Cervarix** 53/3727 (1.4%)	39/3739 (1.0%)	Blinded investigators
2v Konno 2010‐JPN	36 months	**Cervarix** 1/519 (0.2%)	0/521 (0%)	Blinded investigators
2v PATRICIA 2012‐INT	48 months	**Cervarix** 10/9319 (0.1%)	5/9325 (0.05%)	Blinded investigators
2v VIVIANE 2014‐INT	48 months	**Cervarix** 5/2877 (0.2%)	8/2870 (0.3%)	Blinded investigators
2v Zhu 2014‐CHNa	72 months	**Cervarix** 0/3026 (9%)	1/3025 (0.03%)	Unblinded investigators
4v FUTURE 2007‐INT	48 months	**Gardasil** 1/2673 (0.04%)	0/2672 (0%)	Not reported
4v Wei 2019‐CHN	90 months	**Gardasil** 0/1499 (0%)	1/1498 (0.07%)	Blinded investigators
4v9v Joura 2015‐INT	72 months	**Gardasil‐9** 4/7071 (0.06%)	**Gardasil** 3/7078 (0.04%)	Blinded investigators and monitoring board
2v Romanowski 2011‐CAN/GER	7 months	**2d. Cervarix** 0/721 (0%)	**3d. Cervarix** 1/239 (0.4%)	Investigators, blinding not reported

**Abbreviations**: 2d.: two doses; 3d.: three doses; CI: confidence interval; RR: risk ratio; SAE: serious adverse event

Due to different methods of adverse events monitoring and data collection across studies, the assumption of transitivity was not considered to be satisfied for any of the networks. Consequently, NMA was not included in the main results; however, NMA results are provided in Appendix 3 for completeness.

###### 1.8.1 Females 14 years or younger: serious adverse events (NMA not possible)

Twelve trials in females aged 14 years or younger at the time of the first dose reported on serious adverse events at up to three years follow‐up (2v4v Draper 2013‐UK; 2v4v Leung 2015‐INT; 2v9v DoRIS 2022‐TZN; 2v Kim 2010‐KOR; 2v Lin 2018‐LA; 2v Medina 2010‐INT; 2v Pedersen 2012‐NA/EU; 2v Schmeink 2011‐NLD/SWE; 2v Zhu 2014‐CHNb; 4v9v Vesikari 2015‐EU; 4v Mugo 2015‐AF; 4v NCT00411749 2006‐JPN).

Evidence ranged from high‐ to very low‐certainty, with RRs ranging from 1.00 to 1.57 for HPV vaccine compared with control, 0.50 to 1.33 for HPV vaccine compared with another HPV vaccine, and 0.20 to 2.00 for dose comparisons. The 95% CIs for all comparisons included both fewer and more cases (Table; Table). See Table for all results for all comparisons.

**12:** Adverse events results

**Population**	**RR (95% CI)** **Participants (trials)**				
	**Serious adverse events** ***NMA***	**Serious adverse events**	**Unsolicited adverse events**	**Adverse events that lead to discontinuation**	**Total adverse events**
***Any HPV vaccine vs control*** (Analysis 1.16; Analysis 1.32; Analysis 1.33; Analysis 1.34)					
All populations	‐	0.99 (0.94 to 1.04) 97,272 (39)	1.04 (0.99 to 1.09) 39,592 (20)	0.98 (0.64 to 1.50) 59,033 (31)	1.09 (1.07 to 1.12) 42,942 (29)
***Cervarix vs control*** (Analysis 2.11; Analysis 2.28; Analysis 2.29; Analysis 2.30)					
Female < 15 years	1.01 (0.63 to 1.60)	1.00 (0.62 to 1.60) 4838 (6)	1.08 (0.95 to 1.22 4838 (6)	0.20 (0.02 to 1.71 4097 (5)	1.11 (1.06 to 1.16) 2264 (5)
Female 15 to 25 years	1.00 (0.94 to 1.06)	1.00 (0.94 to 1.06) 37,802 (12)	1.04 (0.95 to 1.14) 17,169 (8)	1.13 (0.51 to 2.53) 29,274 (11)	1.05 (1.03 to 1.07) 15,188 (9)
Female > 25 years	1.07 (0.92 to 1.25)	1.07 (0.92 to 1.26) 6959 (2)	0.99 (0.93 to 1.05) 6959 (2)	1.87 (1.04 to 3.37) 6959 (2)	1.28 (0.92 to 1.76) 6876 (2)
Males 15 to 25 years	1.48 (0.16 to 13.98)	1.48 (0.16 to 13.98) 370 (1)	1.07 (0.76 to 1.51) 268 (1)	1.48 (0.06 to 36.05) 270 (1)	1.14 (1.03 to 1.25) 268 (1)
**Gardasil vs control** (Analysis 3.21; Analysis 3.39; Analysis 3.40; Analysis 3.41)					
Female and male	‐	0.98 (0.47 to 2.05) 6524 (4)	1.01 (0.86 to 1.19) 1692 (1)	0.72 (0.32 to 1.63) 4775 (3)	1.17 (1.07 to 1.27) 4832 (3)
Female < 15 years	0.64 (0.32 to 1.28)	1.57 (0.08 to 31.59) 205 (2)	‐	Not estimable, no events 98 (1)	1.03 (0.79 to 1.32) 98 (1)
Female 15 to 25 years	0.77 (0.63 to 0.94)	0.80 (0.61 to 1.04) 19,467 (6)	‐	0.94 (0.10 to 9.01) 1777 (3)	1.07 (1.03 to 1.12) 1633 (3)
Female > 25 years	0.89 (0.67 to 1.18)	0.88 (0.61 to 1.27) 6775 (2)	‐	1.62 (0.32 to 8.20) 6775 (2)	1.07 (1.05 to 1.10) 6775 (2)
Males 15 to 25 years	0.69 (0.29 to 1.65)	0.69 (0.29 to 1.65) 5008 (2)	‐	0.33 (0.12 to 0.85) 5008 (2)	1.08 (1.04 to 1.12) 5008 (2)
***Gardasil‐9 vs control*** (Analysis 4.1)					
Female < 15 years	1.23 (0.59 to 2.59)	‐	‐	‐	‐
Female 15 to 25 years	0.96 (0.76 to 1.21)	0.87 (0.56 to 1.36) 1515 (1)	‐	‐	‐
Female > 25 years		‐	‐	‐	‐
Males 15 to 25 years	0.05 (0.00 to 1.06)	‐	‐	‐	‐
***Cecolin vs control*** (Analysis 5.1; Analysis 5.12)					
Female < 15 years	‐	‐	‐	‐	‐
Female 15 to 25 years	1.22 (0.34 to 4.34)	1.22 (0.34 to 4.34) 1594 (1)	1.00 (0.86 to 1.17) 1594 (1)	‐	‐
Female > 25 years	0.99 (0.83 to 1.19)	0.99 (0.83 to 1.19) 7072 (1)	1.10 (1.04 to 1.17) 7072 (1)	‐	‐
Males 15 to 25 years	‐	‐	‐	‐	‐
***Gardasil vs Cervarix*** (Analysis 6.1; Analysis 6.9; Analysis 6.10; Analysis 6.11)					
Female < 15 years	0.64 (0.38 to 1.08)	0.59 (0.34 to 1.02) 1273 (2)	1.09 (0.88 to 1.34) 1075 (1)	1.51 (0.06 to 36.88) 1075 (1)	0.95 (0.92 to 0.98) 1072 (1)
Female 15 to 25 years	0.77 (0.63 to 0.95)	No events, 62 (1)	‐	‐	0.38 (0.17 to 0.83) 62 (1)
Female > 25 years	0.83 (0.62 to 1.11)	0.84 (0.55 to 1.28) 1106 (1)	0.86 (0.74 to 1.00) 1106 (1)	0.60 (0.14 to 2.50) 1106 (1)	0.91 (0.88 to 0.95) 1051 (1)
Males 15 to 25 years	0.47 (0.04 to 5.21)	‐	‐	‐	‐
***Gardasil‐9 vs Cervari*****x** (Analysis 7.1)					
Female < 15 years	1.23 (0.69 to 2.19)	1.33 (0.73 to 2.42) 930 (1)	‐	‐	‐
Female 15 to 25 years	0.96 (0.75 to 1.21)	0.87 (0.56 to 1.37) 1518 (1)	‐	‐	‐
Female > 25 years	‐	‐	‐	‐	‐
Males 15 to 25 years	0.04 (0.00 to 1.53)	‐	‐	‐	‐
***Gardasil‐9 vs Gardasil*** (Analysis 8.15; Analysis 8.31; Analysis 8.32)					
Female < 15 years	1. 92 (0.90 to 4.11)	0.50 (0.05 to 5.50) 599 (1)	‐	1.00 (0.06 to 15.97) 599 (1)	1.02 (0.99 to 1.06) 599 (1)
Female 15 to 25 years	1.24 (1.04 to 1.48)	1.27 (1.05 to 1.53) 14,149 (1)	‐	1.60 (0.52 to 4.89) 14,149 (1)	1.04 (1.03 to 1.05) 14,149 (1)
Female > 25 years	‐	‐	‐	‐	‐
Males 15 to 25 years	0.08 (0.00 to 1.36)	0.08 (0.00 to 1.36) 496 (1)	‐	Not estimable, no events 496 (1)	1.00 (0.93 to 1.09) 496 (1)
***Cecolin vs Cervarix*** (no direct evidence)					
Female 15 to 25 years	1.22 (0.34 to 4.34)	‐	‐	‐	‐
Female > 25 years	0.93 (0.73 to 1.18)	‐	‐	‐	‐
***Cecolin vs Gardasil*** (no direct evidence)					
Female 15 to 25 years	1.58 (0.44 to 5.71)	‐	‐	‐	‐
Female >25 years	1.12 (0.80 to 1.57)	‐	‐	‐	‐
***Cecolin vs Gardasil‐9***(no direct evidence)					
Female 15 to 25 years	1.27 (0.35 to 4.63)	‐	‐	‐	‐
***2 vs 3 doses Cervarix*** (Analysis 9.1; Analysis 9.8; Analysis 9.9)					
Female < 15 years	‐	0.67 (0.19 to 2.32) 310 (1)	‐	‐	‐
Female 15 to 25 years	‐	1.08 (0.62 to 1.89) 960 (1)	0.76 (0.64 to 0.90) 960 (1)	0.66 (0.06 to 7.28) 960 (1)	‐
***1 vs 2 doses Cervarix*** (Analysis 10.1)					
Female < 15 years	‐	2.00 (0.61 to 6.50) 310 (1)	‐	‐	‐
***1 vs 3 doses Cervarix*** (Analysis 11.1)					
Female < 15 years	‐	1.33 (0.47 to 3.75) 310 (1)	‐	‐	‐
***2 vs 3 doses Gardasil*** (Analysis 12.1; Analysis 12.5; Analysis 12.6; Analysis 12.7)					
Female < 15 years	‐	0.79 (0.36 to 1.71) 1227 (2)	0.95 (0.75 to 1.21) 716 (1)	0.33 (0.01 to 8.16) 716 (1)	0.99 (0.94 to 1.03) 713 (1)
***2 vs 3 doses Gardasil‐9*** (Analysis 13.1)					
Female < 15 years	‐	1.01 (0.49 to 2.09) 904 (2)	‐	‐	‐
***1 vs 2 doses Gardasil‐9*** (Analysis 14.1)					
Female < 15 years	‐	1.00 (0.39 to 2.60) 310 (1)	‐	‐	‐
***1 vs 3 doses Gardasil‐9*** (Analysis 15.1)					
Female < 15 years	‐	1.00 (0.39 to 2.60) 310 (1)	‐	‐	‐
***2 vs 3 doses Cecolin*** (Analysis 16.1; Analysis 16.5)					
Female < 15 years	‐	0.20 (0.01 to 4.19) 605 (1)	0.67 (0.56 to 0.81) 605 (1)	‐	‐

**Abbreviations**: CI: confidence interval; HPV: human papillomavirus; NMA: network meta‐analysis; RR: risk ratio

###### 1.8.2 Females 15 to 25 years: serious adverse events (NMA not possible)

Twenty‐one trials in females aged 15 to 25 years at the time of the first dose reported on serious adverse events at up to six years follow‐up (2v4v Sangar 2015‐IND; 2v9v KENSHE 2021‐KEN; 2v Bhatla 2010‐IND; 2v CVT 2011‐CRI; 2v Harper 2004‐BRA/NA; 2v Kim 2011‐KOR; 2v Konno 2010‐JPN; 2v Leroux‐Roels 2011‐BEL; 2v Lim 2014‐MYS; 2v Ngan 2010‐HKG; 2v PATRICIA 2012‐INT; 2v Sow 2013‐SEN/TZN; 2v Zhu 2014‐CHNa; 4v9v Joura 2015‐INT; 4v EVRI 2016‐ZAF; 4v FUTURE 2007‐INT; 4v FUTURE II 2007‐INT; 4v Kang 2008‐KOR; 4v Villa 2005‐INT; 4v Yoshikawa 2013‐JPN; Cecolin 2v Wu 2015‐CHN).

Evidence ranged from high‐ to very low‐certainty, with RRs ranging from 0.80 to 1.22 for HPV vaccine compared with control, 0.87 to 1.27 for HPV vaccine compared with another HPV vaccine, and 0.20 to 2.00 for dose comparisons. The 95% CIs for most comparisons included both fewer and more cases (Table; Table). The exception was the result for Gardasil‐9 versus Gardasil, which favoured Gardasil. See Table for all results for all comparisons.

###### 1.8.3 Females > 25 years: serious adverse events (NMA not possible)

Six trials in females aged > 25 years at the time of the first dose reported on serious adverse events at up to 90 months follow‐up (2v4v Einstein 2009‐USA; 2v VIVIANE 2014‐INT; 2v Zhu 2014‐CHNc; 4v FUTURE III 2009‐INT; 4v Wei 2019‐CHN; Cecolin 2v Qiao 2020‐CHN).

Evidence ranged from high‐ to low‐certainty, with RRs ranging from 0.84 to 1.07 and 95% CIs for all comparisons included both fewer and more cases (Table). See Table for all results for all comparisons.

###### 1.8.4 Males 15 to 25 years: serious adverse events (NMA not possible)

Four trials in males aged 15 to 25 years at the time of the first dose reported on serious adverse events at up to 57 months follow‐up (2v Petaja 2009‐FIN; 4v9v Van Damme 2016‐EU; 4v Giuliano 2011‐INT; 4v Mikamo 2019‐JPN).

Evidence ranged from moderate‐ to low‐certainty, with RRs ranging from 0.08 to 1.48 and 95% CIs for all comparisons included both fewer and more cases (Table). See Table for all results for all comparisons.

###### 1.8.5 HPV vaccine compared with control in all populations: serious adverse events (standard meta‐analysis)

Thirty‐nine trials reported on serious adverse events and compared Cervarix, Gardasil, Gardasil‐9 or Cecolin with control. The trials reported 1 fewer case per 1000 participants (95% CI 3 fewer to 2 more) in the HPV vaccine groups compared with the control group rate of 57 per 1000 at up to 72 months follow‐up (high‐certainty evidence; RR 0.99, 95% CI 0.94 to 1.04; 97,272 participants; Analysis 1.16; Table).

####### Subgroup and sensitivity analyses

In HICs, 14 trials found that HPV vaccination was associated with a potentially lower rate of serious adverse events, with 8 fewer cases per 100 participants (95% CI 2 to 12 fewer; RR 0.73, 95% CI 0.57 to 0.93; 10,376 participants; analysis not shown) whereas the 95% CIs for the other setting subgroups (LMIC, UMIC, or mixed settings) included both fewer and more cases, consistent with the overall results (Analysis 1.16).

No notable subgroup differences were detected for presence of national screening programme, type of comparison group, type of funding source or presence of conflicts of interest. The 95% CIs for all these subgroups included both fewer and more cases.

The remaining planned subgroup analyses could not be carried out. All trials reporting on this outcome had a similar interval duration between doses (month 0, 1, 6 schedule or month 0, 2, 6 schedule for three‐dose schedules, and six‐month interval between doses for two‐dose schedules). For history of sexual activity and HPV DNA status at baseline, sufficient data were not reported in the trials.

Sensitivity analysis using intention‐to‐treat populations showed similar results to the main analysis (RR 0.98, 95% CI 0.94 to 1.03; 39 trials, 98,179 participants; analysis not shown). We did not carry out the remaining planned sensitivity analyses for this outcome, since there were no pooled analyses included for this outcome, events were not rare (< 1%), risk of bias was not high for any of the included studies, there were no trials reported in abstracts only and there were no cluster trials in the meta‐analysis.

##### 1.9 Treatment rates for HPV‐related pre‐invasive disease

###### 1.9.1 Females 14 years or younger: treatment rates for HPV‐related pre‐invasive disease (no trials)

There were no trials that reported on treatment for HPV‐related pre‐invasive disease in females receiving their first dose aged 14 years or younger.

###### 1.9.2 Females 15 to 25 years: treatment rates for HPV‐related pre‐invasive disease (NMA not possible)

Three trials reported on treatment for HPV‐related pre‐invasive disease in females that received their first dose aged 15 to 25 years of age (2v CVT 2011‐CRI; 2v PATRICIA 2012‐INT; 4v9v Joura 2015‐INT). We did not carry out NMA because studies were disjointed. Two Cervarix trials found a reduction in treatment compared with control at 48 months follow‐up (moderate‐certainty evidence, RR 0.76, 95% CI 0.59 to 0.98; 25,488 participants; Analysis 2.12). A trial comparing Gardasil‐9 with Gardasil found a reduction in HPV 31, 33, 45, 52 or 58‐related treatment (RR 0.67, 95% CI 0.53 to 0.86; 13,754 participants; Analysis 8.17), but little to no difference for HPV 6, 11, 16 or 18‐related treatment (high‐certainty evidence, RR 1.05, 95% CI 0.81 to 1.37; 13,754 participants; Analysis 8.16) at 72 months follow‐up. See Table for results and Table.

**13:** Treatment rates for HPV‐related pre‐invasive disease results

**Population**	**Outcome definition**	**Follow‐up**	**Participants (trials)**	**RR (95% CI)**
***Any HPV vaccine vs control*** (Analysis 1.17)				
All populations	Various, see below	Up to 84 months	38,604 (5)	0.76 (0.65 to 0.89)
***Cervarix vs control*** (Analysis 2.12)				
Female < 15 years	‐	‐	0 (0)	‐
Female 15 to 25 years	LEEP or cervical excision	48 months	25,488 (2)	0.76 (0.59 to 0.98)
Female > 25 years	Local cervical therapy (LEEP, cone, knife or laser)	84 months	5468 (1)	0.80 (0.61 to 1.05)
Males 15 to 25 years	‐	‐	0 (0)	‐
**Gardasil vs control** (Analysis 3.22)				
Female < 15 years	‐	‐	0 (0)	‐
Female 15 to 25 years	‐	‐	0 (0)	‐
Female > 25 years	External genital or cervical definitive therapy	48 months	3768 (1)	0.91 (0.74 to 1.13)
Males 15 to 25 years	Surgical procedures for external genital lesions with a diagnosis of condyloma, PeIN or penile/perianal/perineal cancer	36 months	3880 (1)	0.59 (0.45 to 0.78)
***Gardasil‐9 vs control:** no trials*				
***Cecolin vs control:** no trials*				
***Gardasil‐9 vs Gardasil*** (Analysis 8.16; Analysis 8.17)				
Female < 15 years	‐	‐	0 (0)	‐
Female 15 to 25 years	Cervical definitive therapy related to HPV	72 months	14,042 (1)	**HPV 6,11,16,18** 1.02 (0.68 to 1.53) **HPV 31,33,45,52,58** 0.75 (0.32 to 1.78)
Female > 25 years	‐	‐	0 (0)	‐
Males 15 to 25 years	‐	‐	0 (0)	‐
***Other HPV vaccine comparisons and comparisons between dose schedules:** no trials*				

**Abbreviations**: CI: confidence interval; HPV: human papillomavirus; LEEP: loop electrosurgical excision procedure; PeIN: penile, perineal, or perianal intraepithelial neoplasia; RR: risk ratio

###### 1.9.3 Females > 25 years: treatment rates for HPV‐related pre‐invasive disease (NMA not possible)

Two trials reported on treatment for HPV‐related pre‐invasive disease in females that received their first dose > 25 years of age (2v VIVIANE 2014‐INT; 4v FUTURE III 2009‐INT). Consequently, we did not carry out NMA. The studies found little to no difference between Cervarix at 84 months follow‐up (moderate‐certainty evidence, RR 0.80, 95% CI 0.61 to 1.05; 1 trial, 5468 participants; Analysis 2.12) or Gardasil at 48 months follow‐up (moderate‐certainty evidence, RR 0.91, 95% CI 0.74 to 1.13; 1 trial, 3768 participants; Analysis 3.22) compared with control. See Table for results and Table.

###### 1.9.4 Males 15 to 25 years: treatment rates for HPV‐related pre‐invasive disease (one trial)

One trial reported on treatment for HPV‐related pre‐invasive disease in males that received their first dose at 15 to 25 years of age (4v Giuliano 2011‐INT). Consequently, we did not carry out NMA. There was high‐certainty evidence of a reduction with Gardasil compared with control (RR 0.59, 95% CI 0.45 to 0.78; 3880 participants; Analysis 3.22). See Table for results and Table.

###### 1.9.5 HPV vaccine compared with control in all populations: treatment rates for HPV‐related pre‐invasive disease (standard meta‐analysis)

Five trials reported on treatment for HPV‐related pre‐invasive disease and compared Cervarix or Gardasil with control (2v CVT 2011‐CRI; 2v PATRICIA 2012‐INT; 2v VIVIANE 2014‐INT; 4v FUTURE III 2009‐INT; 4v Giuliano 2011‐INT). The trials reported 12 fewer cases per 1000 participants (95% CI 5 to 17 fewer) in the HPV vaccine groups compared with the control group rate of 49 per 1000 at up to 84 months follow‐up (moderate‐certainty evidence, RR 0.76, 95% CI 0.65 to 0.89; I^2^ = 62%; 38,604 participants; Analysis 1.17; Table). Trials comparing Gardasil‐9 or Cecolin with control did not report on treatment for HPV‐related pre‐invasive disease.

####### Subgroup and sensitivity analyses

We did not carry out subgroup analyses since fewer than 10 studies were included for this outcome.

Sensitivity analysis using intention‐to‐treat populations showed similar results (RR 0.76, 95% CI 0.65 to 0.89; 5 trials, 39,741 participants; analysis not shown). We did not carry out the remaining planned sensitivity analyses since there were no pooled analyses included for this outcome, events were not rare (< 1%), risk of bias was not high for any of the included studies, there were no trials reported in abstracts only and there were no cluster trials in the meta‐analysis.

##### 1.10 Anogenital warts irrespective of HPV type

Vaccine‐type anogenital warts is an important outcome reported in section 2.4 below.

###### 1.10.1 Females 14 years or younger: anogenital warts irrespective of HPV type (no trials)

There were no trials that reported on anogenital warts in females receiving their first dose at 14 years or younger.

###### 1.10.2 Females 15 to 25 years: anogenital warts irrespective of HPV type (NMA)

We carried out a NMA with three trials for anogenital warts irrespective of HPV type in females receiving their first dose at 15 to 25 years (4v9v Joura 2015‐INT; 4v FUTURE I/II 2010‐INT (pooled analysis of two trials)); see Figure for the network map, interval plot, consistency plot and funnel plot, and Appendix 3 for the matrix of results (league table) and rank.

**9:**
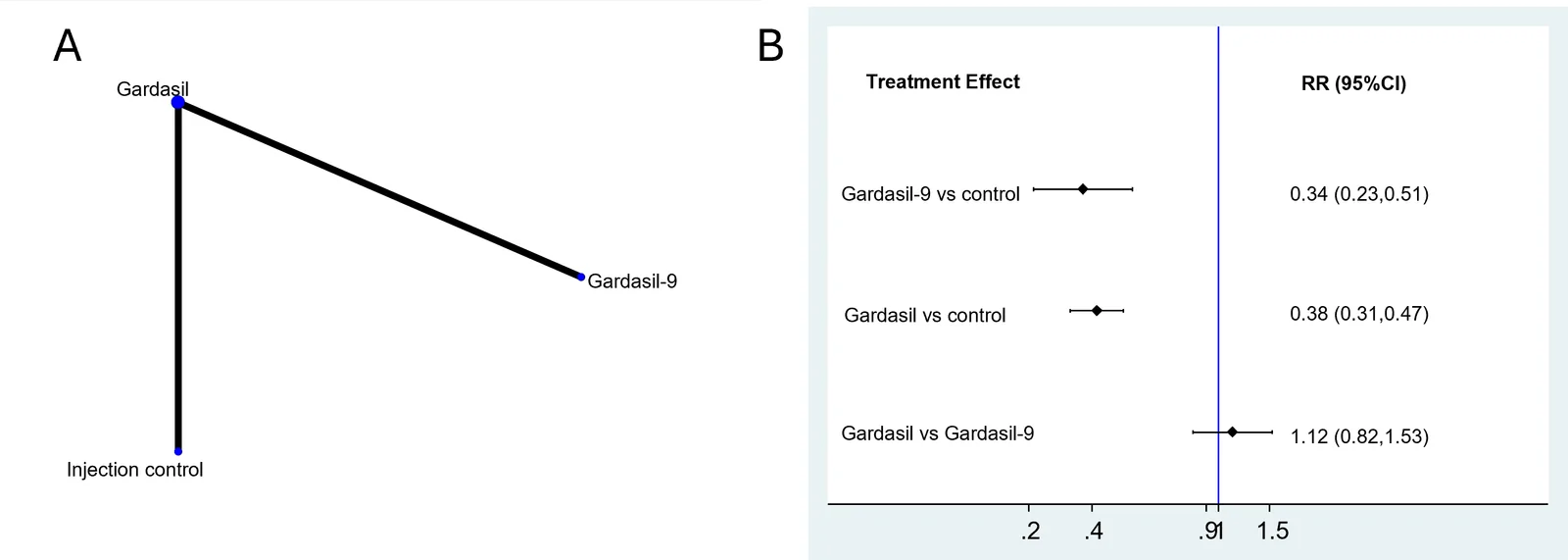
NMA of anogenital warts irrespective of HPV type in females 15 to 25 years old: A) network map, B) interval plot

In the NMA, there was high‐certainty evidence of a reduction in anogenital warts irrespective of HPV type for Gardasil compared with control (RR 0.38, 95% CI 0.31 to 0.47) and Gardasil‐9 compared with control (RR 0.34, 95% CI 0.23 to 0.51). There was high‐certainty evidence of little to no difference in anogenital warts irrespective of HPV type between Gardasil‐9 and Gardasil (RR 0.90, 95% CI 0.65 to 1.23). There was no evidence on anogenital warts irrespective of HPV type from trials assessing Cervarix or Cecolin, or from trials assessing different dose schedules. See Table for standard meta‐analysis and NMA results and Table.

**14:** Anogenital warts results

**Population**	**RR (95% CI)** **Participants (trials)**		
	**Anogenital warts irrespective of HPV type** *****NMA*****	**Anogenital warts irrespective of HPV type**	Anogenital warts, vaccine‐matched HPV‐type*
***Any HPV vaccine vs control*** (Analysis 1.18; Analysis 1.19)			
All populations	‐	0.38 (0.32 to 0.46) 21,271 (3)	0.28 (0.18 to 0.45) 25,179 (5)
***Cervarix vs control:** no trials*			
***Gardasil vs control*** (Analysis 3.23; Analysis 3.24)			
Female < 15 years	‐	‐	‐
Female 15 to 25 years	0.38 (0.31 to 0.47)	0.38 (0.31 to 0.47) 17,391 (2)	0.21 (0.16 to 0.27) 17,917 (3)
Female > 25 years	‐	‐	0.58 (0.23 to 1.47) 3392 (1)
Male 15 to 25 years	‐	0.39 (0.26 to 0.58) 3880 (1)	0.32 (0.20 to 0.51) 3880 (1)
***Gardasil‐9 vs control:** indirect evidence*			
Female < 15 years	‐	‐	‐
Female 15 to 25 years	0.34 (0.23 to 0.51)	‐	‐
Female > 25 years	‐	‐	‐
Male 15 to 25 years	‐	‐	‐
***Cecolin vs injection control**: no trials*			
***Gardasil‐9 vs Gardasil***(Analysis 8.18; Analysis 8.19; Analysis 8.20)			
Female < 15 years	‐	‐	‐
Female 15 to 25 years	0.90 (0.65 to 1.23)	0.90 (0.66 to 1.22) 14,050 (1)	**HPV 6,11,16,18:** 0.98 (0.65 to 1.47) 14,042 (1) **HPV 31,33,45,52,58:** 0.75 (0.32 to 1.78) 14,042 (1)
Female > 25 years	‐	‐	‐
Male 15 to 25 years	‐	‐	‐
***Other HPV vaccine comparisons:** no trials*			
***Dose schedule comparisons**: no trials*			

**Abbreviations**: CI: confidence interval; HPV: human papillomavirus; NMA: network meta‐analysis; RR: risk ratio *Vaccine‐matched HPV‐type indicates positive for HPV 16 and/or 18 for Cervarix and Cecolin; HPV 6, 11, 16 and/or 18 for Gardasil; HPV 6, 11, 16, 18, 31, 33, 45, 52 and/or 58 for Gardasil‐9.

In the NMA, Gardasil‐9 was found to have the highest probability (75.6%) of being the best vaccine to reduce the risk of developing anogenital warts, followed by Gardasil (24.4%).

###### 1.10.3 Females > 25 years: anogenital warts irrespective of HPV type (no trials)

There were no trials that reported on anogenital warts irrespective of HPV type in females receiving their first dose at > 25 years.

###### 1.10.4 Males 15 to 25 years: anogenital warts irrespective of HPV type (single trial)

One trial reported on anogenital warts irrespective of HPV type in males that received their first dose at 15 to 25 years of age (4v Giuliano 2011‐INT). Consequently, we did not carry out NMA. There was high‐certainty evidence of a reduction with Gardasil compared with control at 36 months follow‐up (RR 0.39, 95% CI 0.26 to 0.58; 3880 participants; Analysis 3.23). See Table for results and Table.

###### 1.10.5 HPV vaccine compared with control in all populations: anogenital warts irrespective of HPV type (standard meta‐analysis)

Three trials reported on anogenital warts irrespective of HPV type and compared Gardasil with control (4v FUTURE I/II 2010‐INT (combining results for two trials); 4v Giuliano 2011‐INT). The trials reported 25 fewer cases per 1000 participants (95% CI 22 to 28 fewer) in the HPV vaccine groups compared with the control group rate of 41 per 1000 at up to 48 months follow‐up (high‐certainty evidence, RR 0.38, 95% CI 0.32 to 0.46; 21,271 participants; Analysis 1.18; Table). Trials comparing Cervarix, Gardasil‐9 or Cecolin with control did not report on anogenital warts irrespective of HPV type.

####### Subgroup and sensitivity analyses

We did not carry out subgroup analyses since fewer than 10 studies were included for this outcome.

Sensitivity analysis using intention‐to‐treat populations was similar (RR 0.38, 95% CI 0.32 to 0.46; 3 trials, 21,687 participants; analysis not shown) as was sensitivity analysis excluding pooled analyses (RR 0.39, 95% CI 0.26 to 0.58; 1 trial, 3880 participants; analysis not shown). We did not carry out the remaining planned sensitivity analyses for this outcome because events were not rare (< 1%), risk of bias was not high for any of the included studies, there were no trials reported in abstracts only and there were no cluster trials in the meta‐analysis.

#### 2. Important outcomes

Standard meta‐analysis results are presented below for important outcomes. As planned, we did not prepare NMA or summary of findings tables for important outcomes.

##### 2.1 Cervical disease outcomes

High‐grade CIN+ and invasive cervical cancer are critical cervical disease outcomes with results reported in sections 1.1 and 1.4 above. Results for important cervical disease outcomes, AIS, CIN3 and CIN2, are reported below and in Table (component cervical disease outcome results) and Table (composite cervical disease outcome results) by population and vaccine.

###### 2.1.1 Females 14 years or younger: cervical disease outcomes

No trials in females receiving their first dose aged 14 years or younger reported on cervical disease outcomes.

###### 2.1.2 Females 15 to 25 years: cervical disease outcomes

Nine trials reported cervical disease outcomes in females aged 15 to 25 years at up to 6.4 years follow‐up, five trials compared Cervarix with control (2v CVT 2011‐CRI; 2v Harper 2004‐BRA/NA; 2v Konno 2010‐JPN; 2v PATRICIA 2012‐INT; 2v Zhu 2014‐CHNa), three compared Gardasil with control (4v FUTURE 2007‐INT; 4v FUTURE II 2007‐INT; 4v Villa 2005‐INT), and one compared Gardasil‐9 with Gardasil (4v9v Joura 2015‐INT). See Table and Table for full details of results.

- A reduction was reported in the vaccine group compared with control for the following outcomes and comparisons:
  - AIS irrespective of HPV type for Cervarix compared with control.
  - Vaccine‐type CIN3, CIN2 irrespective of HPV type and vaccine‐type CIN2 for Gardasil compared with control.
  - HPV 31, 33, 45, 52 or 58‐related CIN2 for Gardasil‐9 compared with Gardasil.
- Little to no difference was reported for the following outcomes and comparisons:
  - CIN3 irrespective of HPV type for Gardasil compared with control.
- Results for the following outcomes and comparisons included very wide 95% CIs:
  - AIS irrespective of HPV type for Gardasil compared with control.
  - Vaccine‐type AIS for Cervarix compared with control and for Gardasil compared with control.
  - Vaccine‐type CIN2 for Cervarix compared with control and HPV 6, 11, 16, 18‐related CIN2 for Gardasil‐9 compared with Gardasil.
- No events were reported for the following outcomes and comparisons:
  - Vaccine‐type CIN3 for Cervarix compared with control.

###### 2.1.3 Females > 25 years: cervical disease outcomes

No trials in females receiving their first dose aged 25 years or older reported on important cervical disease outcomes.

###### 2.1.4 HPV vaccine compared with control in all populations: cervical disease outcomes

Eleven trials comparing one of the HPV vaccines with control reported on cervical disease outcomes at up to 84 months follow‐up (2v CVT 2011‐CRI; 2v Harper 2004‐BRA/NA; 2v Konno 2010‐JPN; 2v PATRICIA 2012‐INT; 2v VIVIANE 2014‐INT; 2v Zhu 2014‐CHNa; 4v FUTURE 2007‐INT; 4v FUTURE II 2007‐INT; 4v FUTURE III 2009‐INT; 4v Villa 2005‐INT; 4v Wei 2019‐CHN). See Table and Table for full details of results.

- A reduction was found in the HPV vaccine group compared with control for AIS irrespective of HPV type, HPV‐vaccine type AIS, HPV‐vaccine type CIN3, CIN2 irrespective of HPV type and HPV‐vaccine type CIN2.
- Little to no difference between HPV vaccine and control was found for CIN2 irrespective of HPV type.

In addition, Cecolin 2v Qiao 2020‐CHN reported no cases of CIN2+, VIN2+ or VaIN2+ (as a composite outcome) in the Cecolin group and 10 cases in the control group at 42 months follow‐up (3386 participants in each group).

##### 2.2 Vulval and vaginal disease outcomes

Vaccine‐type high‐grade VIN or VaIN and vaccine‐type vulval or vaginal cancer are critical vulval and vaginal disease outcomes with results reported in sections 1.2 and 1.5 above. Results for important vulval and vaginal disease outcomes: vulval or vaginal cancer irrespective of HPV‐type and high‐grade VIN or VaIN irrespective of HPV‐type are reported below and in Table by population and vaccine.

###### 2.2.1 Females 14 years or younger: vulval and vaginal disease outcomes

No trials in females aged 14 years or younger when they received their first dose reported on vulval and vaginal disease outcomes.

###### 2.2.2 Females 15 to 25 years: vulval and vaginal disease outcomes

Four trials reported on important vulval and vaginal disease outcomes in females aged 15 to 25 years at up to 72 months follow‐up, one trial compared Cervarix with control (2v PATRICIA 2012‐INT), one pooled analysis of two trials compared Gardasil with control (4v FUTURE I/II 2010‐INT), and one trial compared Gardasil‐9 with Gardasil (4v9v Joura 2015‐INT). See Table for full details of results.

- A reduction of high‐grade VIN or VaIN irrespective of HPV type was reported for Cervarix compared with control and Gardasil compared with control.
- Little to no difference in high‐grade VIN or VaIN irrespective of HPV type was reported for Gardasil‐9 compared with Gardasil.
- Results for invasive vulval or vaginal cancer irrespective of HPV type for Gardasil compared with control had very wide 95% CIs: one case of perineal cancer negative for HPV vaccine types and 10 other oncogenic HPV types were reported in the Gardasil group.

###### 2.2.3 Females > 25 years: vulval and vaginal disease outcomes

No trials in females aged > 25 years when they received their first dose reported on important vulval and vaginal disease outcomes. See section 1.5.3 for critical vulval and vaginal disease outcomes in this population group.

###### 2.2.4 HPV vaccine compared with control in all populations: vulval and vaginal disease outcomes

Four trials reported on important vulval and vaginal disease outcomes (2v PATRICIA 2012‐INT; 4v9v Joura 2015‐INT; 4v FUTURE I/II 2010‐INT (pooled analysis of two trials)). See Table for full details of results.

- A reduction of high‐grade VIN or VaIN irrespective of HPV type was found in the HPV vaccine group compared with control at 48 months follow‐up (RR 0.48, 95% CI 0.33 to 0.70; 3 trials, 33,092 participants; Analysis 1.14)
- One case of invasive vulval or vaginal cancer irrespective of HPV type was reported in the HPV vaccine group compared with control at 48 months follow‐up. This was a case of perineal cancer negative for HPV vaccine types and 10 other oncogenic HPV types (RR 3.01, 95% CI 0.12 to 73.85; 1 trial, 5455 participants; Analysis 1.12).

In addition, Cecolin 2v Qiao 2020‐CHN reported no cases of CIN2+, VIN2+ or VaIN2+ in the Cecolin group and 10 cases in the control group at 42 months follow‐up (3386 participants in each group).

##### 2.3 Anal and penile disease outcomes

Anal or penile cancer, high‐grade AIN or PeIN irrespective of HPV type, and vaccine‐type high‐grade AIN or PeIN are critical anal and penile disease outcomes with results reported in sections 1.3 and 1.6, and 1.7 above. Results for important anal and penile disease outcome adenocarcinoma in situ (AIS) irrespective of HPV type is reported below and in Table by population and vaccine.

One included trial reported no cases of intra‐anal AIS irrespective of HPV type in a subgroup of MSM aged 15 to 25 years at 36 months follow‐up (4v Giuliano 2011‐INT; Analysis 1.6). There was no evidence on anal AIS irrespective of HPV type from trials assessing Cervarix, Gardasil‐9 or Cecolin, or in people younger than 15 years.

##### 2.4 Vaccine‐type anogenital warts

Anogenital warts irrespective of HPV type is a critical outcome with results reported in section 1.10 above. Six trials reported on vaccine‐type anogenital warts at up to 72 months follow‐up (4v9v Joura 2015‐INT; 4v FUTURE 2007‐INT; 4v FUTURE II 2007‐INT; 4v FUTURE III 2009‐INT; 4v Giuliano 2011‐INT; 4v Villa 2005‐INT); results are reported below and in Table by population and vaccine.

A reduction was found in the HPV vaccine group compared with control for HPV‐vaccine type anogenital warts in any population (RR 0.28, 95% CI 0.18 to 0.45; 5 trials, 25,179 participants; Analysis 1.19). The results for Gardasil compared with control in females 15 to 25 years and in males 12 to 25 years were similar (Table); little to no difference was found for Gardasil compared with control in females > 25 years and for Gardasil‐9 compared with Gardasil in females 15 to 25 years (Table). There was no evidence on HPV‐vaccine type anogenital warts from trials assessing Cervarix or Cecolin, or in people younger than 15 years.

##### 2.5 Participation rates in cervical screening

One trial with 832 females aged 15 to 25 years reported 54 more per 1000 (95% CI 0 to 115 more) attending cervical cancer screening in the Cervarix group compared with the control group rate of 766 per 1000 at up to 30 months follow‐up (RR 1.07, 95% CI 1.00 to 1.15; Analysis 2.13) (2v Carozzi 2016‐ITA). This was an unblinded, randomised, parallel‐group trial, and the difference in screening rates is likely an effect of increased awareness of HPV in the study group; those in the study group were offered vaccination, as opposed to the control group, who were not.

No trials comparing Gardasil, Gardasil‐9 or Cecolin with control, trials comparing one HPV vaccine with another HPV vaccine, or trials comparing different dose schedules reported on participation rates in cervical screening.

##### 2.6 Adverse pregnancy outcomes

###### 2.6.1 Any adverse pregnancy outcome

Two trials reported on any adverse pregnancy outcome and found 14 more cases per 1000 participants in the HPV vaccine group compared with the control group rate of 136 per 1000 at up to 90 months follow‐up, but 95% CIs also included fewer cases with vaccine (95% CI 38 fewer to 94 more per 1000; RR 1.10, 95% CI 0.72 to 1.69; 5281 participants; Analysis 1.20). See Table for results by vaccine and by population and for comparisons between HPV vaccines.

**15:** Adverse pregnancy outcomes results

**Population**	**RR (95% CI)** **Participants (trials)**						
	**Any adverse pregnancy outcome**	**Cervical cerclage**	**Fetal abnormality**	**Miscarriage**	**Pre‐term birth**	**PROM**	**Stillbirth**
***Any HPV vaccine vs control*** (Analysis 1.20; Analysis 1.21; Analysis 1.22; Analysis 1.23; Analysis 1.24; Analysis 1.25; Analysis 1.26)							
All populations	1.10 (0.72 to 1.69) 5281 (2)	0.20 (0.01 to 4.16) 12,157 (1)	1.20 (0.84 to 1.73) 71,662 (12)	1.01 (0.93 to 1.10) 76,019 (17)	1.13 (0.84 to 1.52) 62,017 (10)	0.72 (0.36 to 1.42) 30,715 (4)	1.08 (0.71 to 1.63) 64,089 (10)
***Cervarix vs control** (*Analysis 2.17*;*Analysis 2.18; Analysis 2.19; Analysis 2.20; Analysis 2.21; Analysis 2.22*)*							
Female < 15 years	‐	‐	‐	* 2067 (1)	‐	‐	‐
Female 15 to 25 years	1.35 (0.68 to 2.68) 1517 (1)	‐	1.07 (0.36 to 3.16) 34,314 (5)	1.06 (0.95 to 1.19) 35,763 (9)	1.17 (0.86 to 1.59) 34,990 (6)	0.58 (0.28 to 1.22) 7466 (1)	1.29 (0.64 to 2.60) 26,848 (4)
Female > 25 years	‐	‐	0.62 (0.20 to 1.90) 5747 (1)	1.03 (0.74 to 1.43) 5747 (1)	* 5747 (1)	2.99 (0.12 to 73.43) 5747 (1)	0.20 (0.01 to 4.15) 5747 (1)
***Gardasil vs control** (*Analysis 3.27; Analysis 3.28; Analysis 3.29; Analysis 3.30; Analysis 3.31; Analysis 3.32; Analysis 3.33*)*							
Female < 15 years	‐	‐	0.94 (0.04 to 22.38) 107 (1)	‐	‐	‐	‐
Female 15 to 25 years	‐	0.20 (0.01 to 4.16) 12,157 (1)	1.50 (0.59 to 3.84) 17,599 (2)	0.93 (0.81 to 1.08) 18,547 (3)	0.82 (0.24 to 2.81) 17,502 (2)	2.08 (0.27 to 16.08) 17,502 (2)	1.16 (0.49 to 2.74) 17,599 (2)
Female > 25 years	0.96 (0.82 to 1.11) 3006 (1)	‐	1.43 (0.72 to 2.82) 6823 (2)	0.98 (0.68 to 1.41) 6823 (2)	0.33 (0.01 to 8.17) 3778 (1)	‐	1.35 (0.17 to 10.74) 6823 (2)
***Gardasil‐9 vs control:** no trials*							
***Cecolin vs control** (*Analysis 5.4; Analysis 5.5; Analysis 5.6*)*							
Female < 15 years	‐	‐	‐	‐	‐	‐	‐
Female 15 to 25 years	‐	‐	‐	‐	‐	‐	‐
Female > 25 years	‐	‐	0.22 (0.01 to 4.52) 7072 (1)	1.09 (0.78 to 1.51) 7072 (1)	‐	‐	0.78 (0.34 to 1.74) 7072 (1)
***Gardasil vs Cervarix** (*Analysis 6.2; Analysis 6.3; Analysis 6.4; Analysis 6.5*)*							
Female < 15 years	‐	‐	‐	‐	‐	‐	‐
Female 15 to 25 years	‐	‐	‐	‐	‐	‐	‐
Female >25 years	‐	‐	5.00 (0.24 to 103.91) 1106 (1)	0.73 (0.34 to 1.58) 1106 (1)	1.00 (0.06 to 15.95) 1106 (1)	‐	0.33 (0.01 to 8.16) 1106 (1)
***Gardasil‐9 vs Cervarix** (*Analysis 7.4; Analysis 7.5; Analysis 7.6*)*							
Female < 15 years	‐	‐	5.33 (0.01 to 8.16) 930 (1)	2.99 (0.12 to 73.13) 920 (1)	‐	‐	‐
Female 15 to 25 years	1.27 (0.70 to 2.29) 1518 (1)	‐	‐	‐	‐	‐	‐
Female > 25 years	‐	‐	‐	‐	‐	‐	‐
***Gardasil‐9 vs Gardasil** (*Analysis 8.25; Analysis 8.26; Analysis 8.27*)*							
Female < 15 years	‐	‐	‐	‐	‐	‐	‐
Female 15 to 25 years	‐	‐	0.93 (0.56 to 1.56) 14,185 (1)	0.84 (0.67 to 1.05) 14,185 (1)	‐	‐	1.00 (0.25 to 4.00) 14,185 (1)
Female > 25 years	‐	‐	‐	‐	‐	‐	‐
***2 vs 3 doses Cervarix** (*Analysis 9.2; Analysis 9.3; Analysis 9.4*)*							
Female < 15 years	‐	‐	‐	‐	‐	‐	‐
Female 15 to 25 years	‐	‐	3.00 (0.12 to 73.08) 310 (1)	* 305 (1)	‐	‐	‐
Female > 25 years	‐	‐	1.00 (0.04 to 24.40) 960 (1)	1.99 (0.24 to 16.44) 960 (1)	1.00 (0.04 to 24.40) 960 (1)	‐	‐
***1 vs 2 doses Cervarix** (*Analysis 10.2; Analysis 10.3*)*							
Female < 15 years	‐	‐	0.33 (0.01 to 8.12) 310 (1)	* 305 (1)	‐	‐	‐
Female 15 to 25 years	‐	‐	‐	‐	‐	‐	‐
Female > 25 years	‐	‐	‐	‐	‐	‐	‐
***1 vs 3 doses Cervarix** (*Analysis 11.2; Analysis 11.3*)*							
Female < 15 years	‐	‐	* 310 (1)	* 308 (1)	‐	‐	‐
Female 15 to 25 years	‐	‐	‐	‐	‐	‐	‐
Female > 25 years	‐	‐	‐	‐	‐	‐	‐
***1 vs 2 vs 3 doses Gardasil**: no trials*							
***2 vs 3 doses Gardasil‐9** (*Analysis 13.2; Analysis 13.3*)*							
Female < 15 years	‐	‐	* 310 (1)	* 307 (1)	‐	‐	‐
Female 15 to 25 years	‐	‐	‐	‐	‐	‐	‐
Female > 25 years	‐	‐	‐	‐	‐	‐	‐
***1 vs 2 doses Gardasil‐9** (*Analysis 14.2; Analysis 14.3*)*							
Female < 15 years	‐	‐	* 310 (1)	2.98 (0.12 to 72.60) 307 (1)	‐	‐	‐
Female 15 to 25 years	‐	‐	‐	‐	‐	‐	‐
Female > 25 years	‐	‐	‐	‐	‐	‐	‐
***1 vs 3 doses Gardasil‐9** (*Analysis 15.2; Analysis 15.3*)*							
Female < 15 years	‐	‐	* 310 (1)	3.00 (0.12 to 73.07) 308 (1)	‐	‐	‐
Female 15 to 25 years	‐	‐	‐	‐	‐	‐	‐
Female > 25 years	‐	‐	‐	‐	‐	‐	‐
***Cecolin dose comparisons**: no trials*							

**Abbreviations**: CI: confidence interval; PROM: premature rupture of membranes; RR: risk ratio *RR not estimable due to zero events. ‐: no studies reported on the outcome for this population.

###### 2.6.2 Infant/fetal abnormality

Twelve trials reported on infant/fetal abnormality and found no more cases per 1000 participants (95% CI 0 to 1 more) in the HPV vaccine group compared with the control group rate of 2 per 1000 at up to 90 months follow‐up (RR 1.20, 95% CI 0.84 to 1.73; 71,662 participants; Analysis 1.21). See Table for results by vaccine and by population and for comparisons between HPV vaccines and dose schedules.

###### 2.6.3 Cervical cerclage and incompetence

One trial with females aged 15 to 25 years reported on cervical cerclage/incompetence and found no more cases per 1000 participants (95% CI 0 to 1 more) in the HPV vaccine group compared with the control group rate of 0.3 per 1000 at up to 48 months follow‐up (RR 0.20, 95% CI 0.01 to 4.16; 12,157 participants; Analysis 1.22) (4v FUTURE II 2007‐INT).

###### 2.6.4 Miscarriage/spontaneous abortion

Seventeen trials reported on miscarriage/spontaneous abortion and found no more cases per 1000 participants (95% CI 2 fewer to 3 more) in the HPV vaccine group compared with the control group rate of 28 per 1000 at up to 90 months follow‐up (RR 1.01, 95% CI 0.93 to 1.10; 76,019 participants; Analysis 1.23). See Table for results by vaccine and by population and for comparisons between HPV vaccines and dose schedules.

###### 2.6.5 Pre‐term birth

Ten trials reported on pre‐term birth and found no more cases per 1000 participants (95% CI 0 to 1 more) in the HPV vaccine group compared with the control group rate of 3 per 1000 at up to 6.4 years follow‐up (RR 1.13, 95% CI 0.84 to 1.52; 62,017 participants; Analysis 1.24). See Table for results by vaccine and by population and for comparisons between HPV vaccines and dose schedules.

###### 2.6.6 Premature rupture of the membranes (PROM)

Four trials reported on PROM and found no more cases per 1000 participants (95% CI 1 fewer to 1 more) in the HPV vaccine group compared with the control group rate of 1 per 1000 at up to 48 months follow‐up (RR 0.72, 95% CI 0.36 to 1.42; 30,715 participants; Analysis 1.25). See Table for results by vaccine and by population.

###### 2.6.7 Stillbirth

Ten trials reported on stillbirth or late fetal death and found no more cases per 1000 participants (95% CI 0 to 1 more per 1000) in the HPV vaccine group compared with the control group rate of 1 per 1000 at up to 90 months follow‐up (RR 1.08, 95% CI 0.71 to 1.63; 64,089 participants; Analysis 1.26). See Table for results by vaccine and by population and for comparisons between HPV vaccines.

##### 2.7 Local adverse events

Thirty‐four trials reported on any local adverse events after any dose. Compared with the control group rate of 617 per 1000, 161 more cases per 1000 participants (95% CI 130 to 198 more) were reported in the HPV vaccine group at up to 15 days follow‐up (RR 1.26, 95% CI 1.21 to 1.32; I^2^ = 94%; 61,326 participants; Analysis 1.27).

For the specific local adverse events pain (RR 1.30, 95% CI 1.24 to 1.36, I^2^ = 94%; 33 trials, 72,225 participants; Analysis 1.28), redness (RR 1.51, 95% CI 1.39 to 1.65, I^2^ = 81%; 31 trials, 69,066 participants; Analysis 1.29) and swelling (RR 1.86, 95% CI 1.65 to 2.11, I^2^ = 89%; 33 trials, 72,225 participants; Analysis 1.30), more cases were reported in the HPV vaccine group compared with the control group at up to 15 days follow‐up.

See Table for results by vaccine and by population, and for comparisons between HPV vaccines.

**16:** Local and systemic adverse events results

**Population**	**Participants (trials)**	**RR (95% CI)**				**Participants (trials)**	**RR (95% CI)**
		**Local AE: any**	**Local AE: pain**	**Local AE: redness**	**Local AE: swelling**	**Systemic adverse events**	
***Any HPV vaccine vs control*** (Analysis 1.27; Analysis 1.28; Analysis 1.29; Analysis 1.30; Analysis 1.31)							
All populations	72,225 (34)	1.26 (1.21 to 1.32)	1.30 (1.24 to 1.36)	1.51 (1.39 to 1.65)	1.86 (1.65 to 2.11)	55,349 (34)	1.07 (1.04 to 1.10)
***Cervarix vs control*** (Analysis 2.23; Analysis 2.24; Analysis 2.25; Analysis 2.26; Analysis 2.27)							
Female < 15 years	4820 (6)	1.28 (1.20 to 1.37)	1.33 (1.22 to 1.45)	1.88 (1.61 to 2.19)	2.25 (1.80 to 2.80)	4820 (6)	1.11 (1.07 to 1.15)
Female 15 to 25 years	16965 (11)	1.24 (1.16 to 1.32)	1.32 (1.20 to 1.45)	1.32 (1.20 to 1.45)	2.02 (1.82 to 2.23)	10,808 (10)	1.06 (1.01 to 1.11)
Female > 25 years	6871 (2)	1.42 (1.14 to 1.77)	1.43 (1.17 to 1.76)	1.43 (1.17 to 1.76)	2.52 (2.30 to 2.77)	6874 (2)	1.27 (0.98 to 1.65)
Male 15 to 25 years	268 (1)	1.83 (1.47 to 2.28)	1.99 (1.57 to 2.53)	1.99 (1.57 to 2.53)	2.51 (1.17 to 5.42)	268 (1)	1.10 (0.95 to 1.28)
***Gardasil vs control***(Analysis 3.34; Analysis 3.35; Analysis 3.36; Analysis 3.37; Analysis 3.38)							
Female and male	4014 (3)	1.65 (1.41 to 1.93)	1.46 (1.18 to 1.82)	1.54 (1.22 to 1.95)	2.20 (1.11 to 4.32)	4015 (3)	1.15 (0.96 to 1.38)
Female < 15 years	98 (1)	1.44 (0.88 to 2.37)	1.42 (0.86 to 2.33)	1.92 (0.26 to 14.47)	1.38 (0.54 to 3.53)	98 (1)	0.83 (0.53 to 1.30)
Female 15 to 25 years	7841 (4)	1.12 (1.09 to 1.16)	1.18 (1.11 to 1.25)	1.44 (1.23 to 1.69)	1.76 (1.38 to 2.26)	8017(5)	1.02 (0.98 to 1.05)
Female > 25 years	6775 (2)	1.26 (1.11 to 1.44)	1.30 (1.10 to 1.54)	1.35 (1.18 to 1.55)	1.79 (1.46 to 2.20)	6775 (5)	1.00 (0.96 to 1.05)
Males 15 to 25 years	5008 (2)	1.11 (1.06 to 1.16)	1.13 (1.07 to 1.19)	1.12 (0.99 to 1.26)	1.29 (1.04 to 1.60)	5008 (2)	1.00 (0.92 to 1.09)
***Gardasil‐9 vs control***: no trials							
***Cecolin vs control*** (Analysis 5.7; Analysis 5.8; Analysis 5.9; Analysis 5.10; Analysis 5.11)							
Female < 15 years	0 (0)	‐	‐	‐	‐	0 (0)	‐
Female 15 to 25 years	1594 (1)	1.21 (0.97 to 1.52)	1.27 (0.99 to 1.63)	1.29 (0.65 to 2.57)	0.99 (0.43 to 2.32)	1594 (1)	1.02 (0.90 to 1.15)
Female > 25 years	7072 (1)	0.98 (0.92 to 1.03)	1.03 (0.97 to 1.09)	0.72 (0.59 to 0.87)	0.62 (0.51 to 0.74)	7072 (1)	1.07 (1.02 to 1.13)
Males 15 to 25 years	0 (0)	‐	‐	‐	‐	0 (0)	‐
***Gardasil vs Cervarix***(Analysis 6.6; Analysis 6.7; Analysis 6.8)							
Female < 15 years	1270 (2)	‐	0.88 (0.85 to 0.92)	0.78 (0.69 to 0.89)	0.75 (0.53 to 1.07)	0 (0)	‐
Female 15 to 25 years	73 (2)	‐	0.53 (0.22 to 1.28)	2.77 (0.31 to 24.48)	0.20 (0.01 to 4.00)	0 (0)	‐
Female > 25 years	1048 (1)	‐	0.77 (0.73 to 0.82)	0.58 (0.48 to 0.69)	0.60 (0.49 to 0.73)	0 (0)	‐
Males 15 to 25 years	0 (0)	‐	‐	‐	‐	0 (0)	‐
***Gardasil‐9 vs Cervarix***: no trials							
***Gardasil‐9 vs Gardasil***(Analysis 8.28; Analysis 8.29; Analysis 8.30)							
Female < 15 years	599 (1)	‐	1.01 (0.96 to 1.07)	1.16 (0.92 to 1.47)	1.33 (1.10 to 1.61)	0 (0)	‐
Female 15 to 25 years	14149 (1)	‐	1.08 (1.06 to 1.09)	1.33 (1.26 to 1.40)	1.39 (1.33 to 1.46)	0 (0)	‐
Female > 25 years	0 (0)	‐	‐	‐	‐	0 (0)	‐
Males 15 to 25 years	496 (1)	‐	1.11 (1.00 to 1.23)	0.88 (0.59 to 1.32)	1.57 (0.96 to 2.56)	0 (0)	‐
***2 vs 3 doses Cervarix*** (Analysis 9.5; Analysis 9.6; Analysis 9.7)							
Female < 15 years	0 (0)	‐	‐	‐	‐	0 (0)	‐
Female 15 to 25 years	953 (1)	‐	0.99 (0.95 to 1.03)	0.79 (0.70 to 0.90)	0.74 (0.63 to 0.87)	0 (0)	‐
Female > 25 years	0 (0)	‐	‐	‐	‐	0 (0)	‐
Males 15 to 25 years	0 (0)	‐	‐	‐	‐	0 (0)	‐
***2 vs 3 doses Gardasil*** (Analysis 12.2; Analysis 12.3; Analysis 12.4)							
Female < 15 years	713 (1)	‐	0.93 (0.87 to 1.00)	0.85 (0.71 to 1.02)	0.83 (0.66 to 1.04)	0 (0)	‐
Female 15 to 25 years	0 (0)	‐	‐	‐	‐	0 (0)	‐
Female > 25 years	0 (0)	‐	‐	‐	‐	0 (0)	‐
Males 15 to 25 years	0 (0)	‐	‐	‐	‐	0 (0)	‐
***2 vs 3 doses Gardasil‐9*** (Analysis 13.4)							
Female < 15 years	594 (1)	‐	0.94 (0.66 to 1.34)	‐	‐	0 (0)	‐
Female 15 to 25 years	0 (0)	‐	‐	‐	‐	0 (0)	‐
Female > 25 years	0 (0)	‐	‐	‐	‐	0 (0)	‐
Males 15 to 25 years	0 (0)	‐	‐	‐	‐	0 (0)	‐
***2 vs 3 doses Cecolin***(Analysis 16.2; Analysis 16.3; Analysis 16.4)							
Female < 15 years	605 (1)	‐	0.97 (0.72 to 1.30)	0.58 (0.17 to 1.95)	1.09 (0.51 to 2.36)	0 (0)	‐
Female 15 to 25 years	0 (0)	‐	‐	‐	‐	0 (0)	‐
Female > 25 years	0 (0)	‐	‐	‐	‐	0 (0)	‐
Males 15 to 25 years	0 (0)	‐	‐	‐	‐	0 (0)	‐
***Other dose comparisons***: no trials							

**Abbreviations**: AE: adverse event; CI: confidence interval; HPV: human papillomavirus; RR: risk ratio

##### 2.8 Systemic adverse events

Thirty‐four trials reported on any systemic adverse events after any dose. Compared with the control group rate of 542 per 1000, 38 more cases per 1000 participants (95% CI 22 to 54 more) were reported in the HPV vaccine group at up to 15 days follow‐up (RR 1.07, 95% CI 1.04 to 1.10; I^2^ = 70%, 55,349 participants; Analysis 1.31). See Table for results by vaccine and by population.

##### 2.9 Total adverse events

Twenty‐eight trials reported on any systemic adverse events after any dose. Compared with the control group rate of 769 per 1000, 69 more cases per 1000 participants (95% CI 54 to 92 more) were reported in the HPV vaccine group at up to 30 days follow‐up (RR 1.09, 95% CI 1.07 to 1.12; I^2^ = 85%, 42,942 participants; Analysis 1.32). See Table for results by vaccine and by population, and for comparisons between HPV vaccines and dose schedules.

##### 2.10 Unsolicited adverse events

Twenty trials reported on unsolicited adverse events after any dose. Compared with the control group rate of 359 per 1000, 14 more cases per 1000 participants (95% CI 4 fewer to 32 more) were reported in the HPV vaccine groups at up to 30 days follow‐up (RR 1.04, 95% CI 0.99 to 1.09; I^2^ = 57%, 39,592 participants; Analysis 1.33). See Table for results by vaccine and by population, and for comparisons between HPV vaccines and dose schedules.

##### 2.11 Adverse events that lead to discontinuation

Thirty trials reported on adverse events that lead to discontinuation. Compared with the control group rate of 2 per 1000, no more cases per 1000 participants (95% CI 1 less to 1 more) were reported in the HPV vaccine groups at up to 90 days follow‐up (RR 0.98, 95% CI 0.64 to 1.50; I^2^ = 17%, 59,033 participants; Analysis 1.34). See Table for results by vaccine and by population, and for comparisons between HPV vaccines and dose schedules.

##### 2.12 Specific adverse events

###### 2.12.1 Postural tachycardia syndrome (POTS)

Four trials reported on POTS at up to six years follow‐up. None of these trials compared an HPV vaccine with a control but compared Gardasil with Cervarix (2v4v Einstein 2009‐USA reporting one event in the Gardasil group and 2v4v Leung 2015‐INT reporting one event in the Cervarix group), Gardasil‐9 with Gardasil (4v9v Joura 2015‐INT reporting one event in the Gardasil‐9 group), and two versus three doses of Gardasil (2v4v Leung 2015‐INT reporting zero events). See Table for detailed results.

**17:** Specific adverse events results

**Population**	**RR (95% CI)** **Participants (trials)**									
	**POTS**	**Paralysis**	**CRPS**	**Premature ovarian failure**	**Infertility**	**Chlamydia infection**	**Gonorrhoea infection**	**Herpes infection**		**HIV infection**
***Any HPV vaccine vs control*** (Analysis 1.35; Analysis 1.36; Analysis 1.37; Analysis 1.38; Analysis 1.39; Analysis 1.40; Analysis 1.41)										
All populations	‐	1.00 (0.20 to 4.95) 12,838 (3)	‐	2.99 (0.12 to 73.43) 5747 (1)	2.00 (0.18 to 22.07) 18,644 (1)	0.97 (0.90 to 1.05) 34,911 (9)	1.10 (0.74 to 1.63) 25,854 (3)	0.85 (0.61 to 1.20) 33,339 (5)		0.33 (0.01 to 8.19) 18,644 (1)
***Cervarix vs control** (*Analysis 2.31; Analysis 2.32; Analysis 2.33; Analysis 2.34; Analysis 2.35; Analysis 2.36*)*										
Female < 15 years	‐	‐	‐	‐	‐	‐	‐	‐	‐	
Female 15 to 25 years	‐	3.01 (0.31 to 28.86) 7091 (2)	‐	‐	2.00 (0.18 to 22.07) 18,664 (1)	1.03 (0.87 to 1.21) 21,759 (5)	‐	1.33 (0.51 to 3.48) 27,587 (4)	0.33 (0.01 to 8.19) 18,644 (1)	
Female > 25 years	‐	0.33 (0.03 to 3.19) 5747 (1)	‐	2.99 (0.12 to 73.43) 5747 (1)	‐	0.60 (0.14 to 2.50) 5747 (1)	‐	0.72 (0.29 to 1.80) 5747 (1)	‐	
Male 15 to 25 years	‐	‐	‐	‐	‐	1.48 (0.06 to 36.05) 270 (1)	‐	‐	‐	
***Gardasil vs control** (*Analysis 3.42; Analysis 3.43*)*										
Female < 15 years	‐	‐	‐	‐	‐	‐	‐	‐	‐	
Female 15 to 25 years	‐	‐	‐	‐	‐	‐	‐	‐	‐	
Female > 25 years	‐	‐	‐	‐	‐	0.87 (0.41 to 1.81) 2997	‐	‐	‐	
Male 15 to 25 years	‐	‐	‐	‐	‐	*Genital:* 2.00 (0.37 to 10.92) *Anal:* 1.00 (0.55 to 1.83) 3637 (1)	‐	‐	‐	
***Gardasil‐9 vs control** (*Analysis 4.3; Analysis 4.4*)*										
Female < 15 years	‐	‐	‐	‐	‐	‐	‐	‐	‐	
Female 15 to 25 years	‐	‐	‐	‐	‐	1.06 (0.82 to 1.37) 969 (1)	1.14 (0.72 to 1.80) 969 (1)	‐	‐	
Female > 25 years	‐	‐	‐	‐	‐	‐	‐	‐	‐	
Male 15 to 25 years	‐	‐	‐	‐	‐	‐	‐	‐	‐	
***Cecolin vs control:** no trials*										
***Gardasil vs Cervarix** (*Analysis 6.12; Analysis 6.13; Analysis 6.14; Analysis 6.15; Analysis 6.16*)*										
Female < 15 years	0.17 (0.01 to 4.10) 1075 (1)	‐	‐	‐	‐	‐	‐	‐	‐	
Female 15 to 25 years	‐	‐	‐	‐	‐	‐	‐	‐	‐	
Female > 25 years	3.00 (0.12 to 73.48) 1106 (1)	0.33 (0.01 to 8.16) 1106 (1)	‐	‐	3.00 (0.12 to 73.48) 1106 (1)	3.00 (0.31 to 28.75) 1106 (1)	‐	0.50 (0.05 to 5.50) 1106 (1)	‐	
Male 15 to 25 years	‐	‐	‐	‐	‐	‐	‐	‐	‐	
***Gardasil‐9 vs Cervarix** (*Analysis 7.7; Analysis 7.8*)*										
Female < 15 years	‐	‐	‐	‐	‐	‐	‐	‐	‐	
Female 15 to 25 years	‐	‐	‐	‐	‐	0.86 (0.68 to 1.10) 985 (1)	1.11 (0.70 to 1.74) 985 (1)	‐	‐	
Female > 25 years	‐	‐	‐	‐	‐	‐	‐	‐	‐	
Male 15 to 25 years	‐	‐	‐	‐	‐	‐	‐	‐	‐	
***Gardasil‐9 vs Gardasil** (*Analysis 8.33; Analysis 8.34; Analysis 8.35; Analysis 8.36; Analysis 8.37; Analysis 8.38; Analysis 8.39; Analysis 8.40*)*										
Female < 15 years	‐	‐	‐	‐	‐	‐	‐	‐	‐	
Female 15 to 25 years	3.00 (0.12 to 73.70) 14,149 (1)	1.20 (0.37 to 3.93) 14,185 (1)	1.00 (0.06 to 15.99) 14,185 (1)	0.50 (0.05 to 5.51) 14,185 (1)	1.00 (0.20 to 4.95) 14,185 (1)	0.67 (0.50 to 0.91) 14,185 (1)	1.00 (0.06 to 15.99) 14,185 (1)	RR 1.22 (0.89 to 1.68) 14,185 (1)	‐	
Female > 25 years	‐	‐	‐	‐	‐	‐	‐	‐	‐	
Male 15 to 25 years	‐	‐	‐	‐	‐	‐	‐	‐	‐	
***2 vs 3 doses Gardasil** (*Analysis 12.8*)*										
Female < 15 years	* 716 (1)	‐	‐	‐	‐	‐	‐	‐	‐	
Female 15 to 25 years	‐	‐	‐	‐	‐	‐	‐	‐	‐	
Female > 25 years	‐	‐	‐	‐	‐	‐	‐	‐	‐	
Male 15 to 25 years	‐	‐	‐	‐	‐	‐	‐	‐	‐	
*Other dose schedule comparisons: no trials*										

**Abbreviations**: CI: confidence interval; CRPS: chronic regional pain syndrome; HIV: human immunodeficiency virus; POTS: postural orthostatic tachycardia syndrome; RR: risk ratio

###### 2.12.2 Chronic fatigue syndrome/myalgic encephalomyelitis (CFS/ME)

No trials reported on this outcome.

###### 2.12.3 Paralysis

Three trials reported on paralysis and found no more cases per 1000 participants (95% CI 0 fewer to 2 more per 1000) in the HPV vaccine group compared with the control group rate of 0.5 per 1000 at up to six years follow‐up (RR 1.00, 95% CI 0.20 to 4.95; 12,838 participants; Analysis 1.35). See Table for results by vaccine and by population and for comparisons between HPV vaccines and dose schedules.

###### 2.12.4 Complex regional pain syndrome (CRPS)

One trial comparing Gardasil‐9 with Gardasil reported on CRPS at up to six years follow‐up (4v9v Joura 2015‐INT). One event was reported in each group. See Table for detailed results.

###### 2.12.5 Premature ovarian failure

One trial reported one case of premature ovarian failure in the HPV vaccine group compared to control at up to 48 months follow‐up (RR 2.99, 95% CI 0.12 to 73.43; 5747 participants; Analysis 1.36). See Table for results by vaccine and by population and for comparisons between HPV vaccines.

###### 2.12.6 Guillain‐Barré syndrome

No trials reported on this outcome.

###### 2.12.7 Infertility

Two trials reported on any adverse pregnancy outcome and found no more cases per 1000 participants (95% CI 0 fewer to 2 more per 1000) in the HPV vaccine group compared with the control group rate of 0.1 per 1000 at up to six years follow‐up (RR 2.00, 95% CI 0.18 to 22.07; 18,644 participants; Analysis 1.37). See Table for results by vaccine and by population and for comparisons between HPV vaccines and dose schedules.

###### 2.12.8 Change in sexual activity

No trials reported on change in sexual activity, but several trials comparing HPV vaccine with control reported on proxy outcomes at up to 90 months follow‐up:

- *Chlamydia trachomatis* infection (RR 0.97, 95% CI 0.90 to 1.05; 9 trials, 34,911 participants; Analysis 1.38).
- *Neisseria* gonorrhoea infection (RR 1.10, 95% CI 0.74 to 1.63; 3 trials, 25,854 participants; Analysis 1.39).
- Genital herpes infection (RR 0.85, 95% CI 0.61 to 1.20; 5 trials, 33,339 participants; Analysis 1.40).
- HIV infection (RR 0.33, 95% CI 0.01 to 8.19; 1 trial, 18,644 participants; Analysis 1.41).

See Table for results by vaccine and by population and for comparisons between HPV vaccines and dose schedules.

##### 2.13 All‐cause mortality

Participants that died were recorded in the trials as deaths, all‐cause mortality or fatal adverse events. In each of 18 of the trials, at least one trial participant died. An additional 21 trials reported that no deaths occurred within those 21 trials. Causes of death are reported in Table for transparency, with none judged to be directly attributable to vaccination. Only one death, due to Crohn's disease, was assessed by the investigator as possibly related to the study vaccination, but it would seem unlikely to be secondary to HPV vaccination, from a mechanistic perspective and based on outcomes from large‐scale epidemiological studies (Bi 2020; Tsai 2023; Willame 2016).

Compared with the control group rate of 1 per 1000, no more cases per 1000 participants (95% CI 0 to 1 more per 1000) were reported in the HPV vaccine groups at up to 90 months follow‐up (RR 1.14, 95% CI 0.71 to 1.83; 31 trials, 91,127 participants; Analysis 1.42). See Table for results by vaccine and by population, and for comparisons between HPV vaccines and dose schedules.

**18:** All‐cause mortality results

**Comparison** **(analysis)**	**Population**	**Follow‐up**	**Participants (trials)**	**Effect measure**
Any HPV vaccine vs control (Analysis 1.42)	All populations		91,127 (31)	RR 1.14, 95% CI 0.71 to 1.83
Cervarix vs control (Analysis 2.37)	Female < 15 years	7 months; 12 months	4097 (5)	Not estimable, zero events
	Female 15 to 25 years		36,629 (11)	RR 0.87, 95% CI 0.47 to 1.61
	Female > 25 years		6959 (2)	RR 2.63, 95% CI 0.99 to 7.01
	Males 15 to 25 years		270 (1)	Not estimable, zero events
Gardasil vs control (Analysis 3.44)	Female and male		4175 (2)	Not estimable, zero events
	Female < 15 years		98 (1)	Not estimable, zero events
	Female 15 to 25 years		18,450 (4)	RR 1.29, 95% CI 0.48 to 3.46
	Female > 25 years		6775 (2)	RR 6.27, 95% CI 1.12 to 35.17
	Males 15 to 25 years		5008 (2)	RR 0.31, 95% CI 0.09 to 1.01
Gardasil‐9 vs control: no trials				
Cecolin vs control (Analysis 5.13)	Female < 15 years		0 (0)	‐
	Female 15 to 25 years		1594 (1)	Not estimable, zero events
	Female > 25 years		7072 (1)	RR 1.09, 95% CI 0.22 to 5.37
	Males 15 to 25 years		0 (0)	‐
Gardasil vs Cervarix (Analysis 6.17)	Female < 15 years		1075 (1)	RR 1.51, 95% CI 0.06 to 36.88
	Female 15 to 25 years		0 (0)	‐
	Female > 25 years		1106 (1)	RR 0.33, 95% CI 0.01 to 8.16
	Males 15 to 25 years		0 (0)	‐
Gardasil‐9 vs Cervarix (Analysis 7.9)	Female < 15 years		930 (1)	RR 3.00, 95% CI 0.12 to 73.45
	Female 15 to 25 years		0 (0)	‐
	Female > 25 years		0 (0)	‐
	Males 15 to 25 years		0 (0)	‐
Gardasil‐9 vs Gardasil (Analysis 8.41)	Female < 15 years		599 (1)	Not estimable, zero events
	Female 15 to 25 years		14,149 (1)	RR 1.20, 95% CI 0.37 to 3.93
	Female > 25 years		0 (0)	‐
	Males 15 to 25 years		496 (1)	Not estimable, zero events
2 vs 3 doses Cervarix (Analysis 9.10)	Female < 15 years		310 (1)	Not estimable, zero events
	Female 15 to 25 years		960 (1)	Not estimable, zero events
	Female > 25 years		0 (0)	‐
	Males 15 to 25 years		0 (0)	‐
1 vs 2 doses Cervarix (Analysis 10.4)	Female < 15 years		310 (1)	Not estimable, zero events
	Female 15 to 25 years		0 (0)	‐
	Female > 25 years		0 (0)	‐
	Males 15 to 25 years		0 (0)	‐
1 vs 3 doses Cervarix (Analysis 11.4)	Female < 15 years		310 (1)	Not estimable, zero events
	Female 15 to 25 years		0 (0)	‐
	Female > 25 years		0 (0)	‐
	Males 15 to 25 years		0 (0)	‐
2 vs 3 doses Cervarix (Analysis 12.9)	Female < 15 years		716 (1)	RR 0.33, 95% CI 0.01 to 8.16
	Female 15 to 25 years		0 (0)	‐
	Female > 25 years		0 (0)	‐
	Males 15 to 25 years		0 (0)	‐
2 vs 3 doses Gardasil‐9 (Analysis 13.5)	Female < 15 years		904 (2)	RR 1.01, 95% CI 0.11 to 9.68
	Female 15 to 25 years		0 (0)	‐
	Female > 25 years		0 (0)	‐
	Males 15 to 25 years		0 (0)	‐
1 vs 2 doses Cervarix (Analysis 14.4)	Female < 15 years		310 (1)	RR 0.33, 95% CI 0.01 to 8.12
	Female 15 to 25 years		0 (0)	‐
	Female > 25 years		0 (0)	‐
	Males 15 to 25 years		0 (0)	‐
1 vs 3 doses Cervarix (Analysis 15.4)	Female < 15 years		310 (1)	Not estimable, zero events
	Female 15 to 25 years		0 (0)	‐
	Female > 25 years		0 (0)	‐
	Males 15 to 25 years		0 (0)	‐

**Abbreviations**: CI: confidence interval; HPV: human papillomavirus; RR: risk ratio

##### 2.14 Incident infection with vaccine HPV genotypes

Seven trials reported on incident infection with vaccine‐specific HPV types. Six of these trials compared Cervarix with control (2v Carozzi 2016‐ITA; 2v CVT 2011‐CRI; 2v Harper 2004‐BRA/NA; 2v Konno 2010‐JPN; 2v PATRICIA 2012‐INT; 2v Zhu 2014‐CHNa) and one trial compared Cecolin with control (Cecolin 2v Qiao 2020‐CHN). Both vaccines showed a relative reduction in incident infection with HPV types, ranging from 74% to 71% at up to 72 months follow‐up; see Table for full results. No trials comparing different HPV vaccines or different dose schedules reported on incident HPV infection.

**19:** Incident and persistent infection with vaccine‐matched HPV‐types* results

**Population**	**RR (95% CI)** **Participants (trials)**		
	**12‐month persistent infection**	**6‐month persistent infection**	**Incident infection**
***Cervarix vs control***(Analysis 2.14; Analysis 2.15; Analysis 2.16)			
Female < 15 years	‐	‐	‐
Female 15 to 25 years	0.09 (0.02 to 0.38) 29,697 (5)	0.13 (0.05 to 0.37) 31,844 (6)	0.26 (0.22 to 0.31) 29,368 (6)
Female > 25 years	0.42 (0.30 to 0.58) 5433 (1)	0.41 (0.32 to 0.54) 5537 (1)	‐
Male 15 to 25 years	‐	‐	‐
***Gardasil vs control***(Analysis 3.25; Analysis 3.26)			
Female < 15 years	‐	‐	‐
Female 15 to 25 years	‐	0.09 (0.04 to 0.19) 1351 (2)	‐
Female > 25 years	0.52 (0.38 to 0.71) 2959 (1)	0.62 (0.43 to 0.90) 6336 (2)	‐
Male 15 to 25 years	0.20 (0.09 to 0.45) 2792 (1)	0.49 (0.41 to 0.58) 4692 (2)	‐
***Gardasil‐9 vs control*** (Analysis 4.2)			
Female < 15 years	‐	‐	‐
Female 15 to 25 years	‐	0.53 (0.42 to 0.66) 1515 (1)	‐
Female > 25 years	‐	‐	‐
Male 15 to 25 years	‐	‐	‐
***Cecolin vs control*** (Analysis 5.2; Analysis 5.3)			
Female < 15 years	‐	‐	‐
Female 15 to 25 years	‐	‐	‐
Female > 25 years	‐	0.22 (0.13 to 0.39) 7042 (1)	0.29 (0.21 to 0.41) 6779 (1)
Male 15 to 25 years	‐	‐	‐
***Gardasil vs Cervarix:** no trials*			
***Gardasil‐9 vs Cervarix*** (Analysis 7.2; Analysis 7.3)			
Female < 15 years	‐	‐	‐
Female 15 to 25 years	‐	**HPV 16,18** 1.12 (0.71 to 1.76) 1518 (1) **HPV 16,18,31,33,45,52,58** 0.58 (0.47 to 0.73) 1518 (1)	‐
Female > 25 years	‐	‐	‐
Male 15 to 25 years	‐	‐	‐
***Gardasil‐9 vs Gardasil*** (Analysis 8.21; Analysis 8.22; Analysis 8.23; Analysis 8.24)			
Female < 15 years	‐	‐	‐
Female 15 to 25 years	**HPV 6,11,16,18** 1.05 (0.93 to 1.17) 13,584 (1) **HPV 31,33,45,52,58** 0.44 (0.40 to 0.48) 13,619 (1)	**HPV 6,11,16,18** 0.98 (0.89 to 1.08) 13,613 (1) **HPV 31,33,45,52,58** 0.43 (0.39 to 0.46) 13,641 (1)	‐
Female > 25 years	‐	‐	‐
Male 15 to 25 years	‐	‐	‐
***Dose schedule comparisons**: no trials*			

**Abbreviations**: CI: confidence interval; HPV: human papillomavirus; RR: risk ratio *Vaccine‐matched HPV‐type indicates positive for HPV 16 and/or 18 for Cervarix and Cecolin; HPV 6, 11, 16 and/or 18 for Gardasil; HPV 6, 11, 16, 18, 31, 33, 45, 52 and/or 58 for Gardasil‐9.

##### 2.15 Persistent infection with vaccine HPV genotypes

Fifteen trials reported on six‐month persistent and/or 12‐month persistent infection with vaccine‐specific HPV types. Fourteen of these trials compared HPV vaccine with control, of which seven trials assessed Cervarix (2v9v KENSHE 2021‐KEN; 2v CVT 2011‐CRI; 2v Harper 2004‐BRA/NA; 2v Konno 2010‐JPN; 2v PATRICIA 2012‐INT; 2v VIVIANE 2014‐INT; 2v Zhu 2014‐CHNa), six Gardasil (4v FUTURE III 2009‐INT; 4v Giuliano 2011‐INT; 4v Mikamo 2019‐JPN; 4v Villa 2005‐INT; 4v Wei 2019‐CHN; 4v Yoshikawa 2013‐JPN), one Gardasil‐9 (2v9v KENSHE 2021‐KEN), and one Cecolin (Cecolin 2v Qiao 2020‐CHN). Two of the trials compared one HPV vaccine with another (2v9v KENSHE 2021‐KEN; 4v9v Joura 2015‐INT). No trials comparing different dose schedules reported on persistent HPV infection.

A reduction of 12‐ or 6‐month persistent HPV infection was found for all four vaccines, ranging from 38% to 91% in different populations at up to 84 months follow‐up; see Table for full results. Trials comparing two different HPV vaccines reported little to no difference for the genotypes included in both vaccines, but a reduction ranging from 42% to 57% for the genotypes included in only one of the vaccines at up to six years follow‐up; see Table for full results.

## Discussion

### Summary of main results

Sixty trials with 157,414 participants were included in this review and 52 trials were included in quantitative analyses. We sourced and extracted clinical study reports for 33 of the trials. For the remaining trials, we extracted results from published papers and results published in online trial registries. Most trials, including all cancer or pre‐invasive disease outcomes, were conducted in females older than the target population for the HPV vaccines. Risk of bias ranged from overall some concerns to low risk.

Results for outcomes we assessed as critical were as follows:

### Cancer outcomes

Only four studies reported on cervical cancer, HPV‐related vulval or vaginal cancer, anal cancer or HPV‐related penile cancer. No cases were detected within the included trials; consequently, we did not carry out NMA. No trials reported on head and neck cancer. Follow‐up was up to six years in the trials reporting on these outcomes, which is not of sufficiently long duration for these cancers to develop. See the partner review Henschke 2025 on population‐level effects of HPV vaccination for these outcomes.

### Pre‐cancer outcomes

High‐grade pre‐invasive disease outcomes were reported in 15‐ to 25‐year‐old populations in 11 trials and in > 25‐year‐old females in three trials with up to seven years follow‐up.

For CIN3+ irrespective of HPV type, there were 5 fewer cases per 1000 participants reported in the Cervarix and Gardasil vaccine groups compared with the control groups at up to 72 months follow‐up, but CIs were also compatible with no difference (95% CI 11 fewer to 3 more, low‐certainty evidence; Table). In the NMA for females 15 to 25 years old, Cervarix was found to have the highest probability of being the best vaccine to reduce the risk of developing CIN3+ irrespective of HPV type, followed by Gardasil (Appendix 3; Table). There were no trials in younger (under 15 years old) or older (over 25 years old) females, nor Gardasil‐9 or Cecolin trials reporting on this outcome.

For vaccine‐type CIN3+, there were 8 fewer cases per 1000 participants (95% CI 6 to 9 fewer) reported in the HPV vaccine groups compared with the control groups at up to 48 months follow‐up (moderate‐certainty evidence; Table). In the NMA for females 15 to 25 years old, Gardasil‐9 was found to have the highest probability of being the best vaccine to reduce the risk of developing vaccine‐type CIN3+, followed by Cervarix and Gardasil (Appendix 3; Table). There were no trials in younger (under 15 years old) or older (over 25 years old) females, nor Cecolin trials reporting on this outcome.

For CIN2+ irrespective of HPV type, there were 10 fewer cases per 1000 participants (95% CI 2 to 16 fewer) reported in the HPV vaccine groups compared with the control groups at up to 72 months follow‐up (moderate‐certainty evidence; Table). In the NMA for females 15 to 25 years old, Cervarix was found to have the highest probability of being the best vaccine to reduce the risk of developing CIN2+ irrespective of HPV type, followed by Gardasil‐9 and Gardasil (Appendix 3; Table). There were two trials in females over 25 years that found little to no difference between Gardasil and control (moderate‐certainty evidence; Table). There were no trials in younger (under 15 years old) females, nor Cecolin trials that reported on this outcome.

For vaccine‐type CIN2+, there were 13 fewer cases per 1000 participants (95% CI 9 to 15 fewer) reported in the HPV vaccine groups compared with the control groups at up to 78 months follow‐up (moderate‐certainty evidence; Table). In the NMA for females 15 to 25 years old, Cervarix was found to have the highest probability of being the best vaccine to reduce the risk of developing vaccine‐type CIN2+, followed by Gardasil‐9 and Gardasil (Appendix 3; Table). In the NMA for females 25 years or older, Cervarix was found to have the highest probability of being the best vaccine to reduce the risk of developing vaccine‐type CIN2+, followed by Gardasil. There were no trials in younger (under 15 years old) females, nor Cecolin trials that reported on this outcome.

In 15‐ to 25‐year‐old females, Gardasil‐9 was found to have the highest probability of being the best vaccine to reduce the risk of developing vaccine‐type high‐grade VIN or VaIN at four years (from 3 cases to 1 case per 1000 people), followed by Cervarix and Gardasil (Appendix 3; Table). There was one trial in females over 25 years with very uncertain evidence due to very wide confidence intervals between Gardasil and control (low‐certainty evidence; Table). There were no trials in younger (under 15 years old) females or Cecolin trials reporting on this outcome.

Data for high‐grade PeIN or AIN analysed both as HPV type and irrespective of HPV type were available from a single trial. There was low certainty for these outcomes, which generally lacked sufficient power to provide meaningful results (Table).

### Treatment rates for HPV‐related pre‐invasive disease

We did not carry out NMA due to trials being split between networks or disjointed within networks. Five trials reported 12 fewer cases per 1000 participants (95% CI 5 to 17 fewer) in the HPV vaccine groups compared with the control groups at up to 84 months follow‐up (moderate‐certainty evidence; Table). The results in females 15 to 25 years old and in males were consistent with the overall effect. In females over 25 years old, the effect was smaller with 95% CIs including both fewer and more cases (Table). There were no trials in younger (under 15 years old) females or Cecolin trials reporting on this outcome.

### Anogenital warts

In the NMA for females 15 to 25 years old, Gardasil‐9 was found to have the highest probability of being the best vaccine to reduce the risk of developing anogenital warts (Appendix 3; Table). There were no trials in younger (under 15 years old) or older (over 25 years old) females, nor Cervarix or Cecolin trials reporting on this outcome.

### Serious adverse events

We did not carry out NMA due to concerns about transitivity. Thirty‐nine trials reported 1 fewer case per 1000 participants (95% CI 3 fewer to 2 more) in the HPV vaccine groups compared with the control groups at up to 72 months follow‐up (high‐certainty evidence; Table). The results in females 14 years or younger, females 15 to 25 years old, females over 25 years and in males were consistent with the overall effect (Table; Table), but the certainty of the evidence for the different comparisons within these population groups ranged from high to very low.

### Completeness

We have performed an extensive review of the literature and engaged with clinicians and experts in this area to perform a rigorous and comprehensive search for the literature in this field. We also obtained information identified by Jorgensen et al. (Jørgensen 2018a; Jørgensen 2018c), included open access clinical study reports (CSRs) from pharmaceutical companies and made applications to the European Medicines Agency for CSRs for other studies. Despite our extensive efforts, there may be missing data (see Potential biases in the review process), but whether this would make a material difference to the main results, as we have included studies with 157,414 participants, is a moot point. Ideally, we would have longer‐term follow‐up data of more than 10 years from previous studies, but it is unlikely that these data will be forthcoming due to challenges in successfully obtaining follow‐up and funding for such studies.

### Applicability

This review demonstrates the limitations of RCTs in examining important long‐term and/or rare outcomes, be they harms or benefits, of an intervention for disease with a long natural history. In addition, RCTs are unable to estimate the effects of vaccination strategies at a population level, where reducing the level of infection within a population can benefit both those vaccinated and those unvaccinated, if coverage is sufficient to induce a degree of herd immunity, so the magnitude of effect of vaccination may be underestimated at an individual level. There are, of course, well‐recognised concerns regarding the applicability of results that arise from selected participants in research settings, and whether similar findings could be obtained across diverse communities and healthcare systems. This review should therefore be read in conjunction with its partner review (Henschke 2025), which examines the population‐level effects of HPV vaccination in a variety of different non‐RCT types.

The natural history of HPV infection, CIN and cervical cancer (as well as of HPV‐dependent VIN and vulval cancer) is well‐understood (McCredie 2008). Surrogate short‐term outcomes were used in many of the studies, as agreed by International Agency for Research on Cancer/WHO guidance (IARC 2014), including immunological responses, hrHPV infection rates and development of CIN2+ and CIN3+ for vaccine‐type HPV. The purpose of this was to "accelerate vaccine development and evaluation", in order to make informed decisions about likely longer‐term efficacy, without awaiting a generation for the clinically relevant outcomes to develop. This limits the applicability of the studies, since they were performed in older adolescents and people who were more likely to have been exposed to HPV prior to vaccination. However, it would have been ethically and clinically inappropriate to subject younger females (e.g. under 15 years of age), in whom CIN, VIN and cervical and vulvo‐vaginal cancers due to HPV are extremely rare (and not normally screened for), to invasive testing for development of CIN. This is compensated for to some degree by some of the studies recruiting HPV‐negative participants, but it is likely that the older cohorts in many of the studies have resulted in an underestimation of the efficacy of HPV vaccination for preventing CIN. It is interesting, and reassuring, that the results of the short‐term outcomes in RCTs are now starting to be mirrored in the long‐term outcomes in population‐level studies, which are able to provide some insights into the effects of vaccination prior to hrHPV exposure (Falcaro 2021; Kjaer 2021; Lei 2020). Whilst RCTs have the innate strength of randomisation and prospective follow‐up, their follow‐up time may be insufficient for certain important outcomes. In some trials, control groups were subsequently offered the vaccine, which obstructs longer‐term follow‐up. Limited follow‐up is also compounded by relatively narrow coverage of the eligible population. These gaps in our knowledge can be usefully supplemented with population‐level studies that are able to achieve extended follow‐up of a wider, more representative sample of the community.

In terms of harms, RCTs, even with large numbers, may not be able to detect changes in rare outcomes. Again, we refer the reader to the companion population‐based study review (Henschke 2025). From both the RCT data in this review and longer‐term population‐level studies with very high person‐year follow‐up (Hviid 2020; Thomsen 2020; Yoon 2021), there does not appear to be an increase overall in serious adverse events or harms with HPV vaccination. With longer‐term follow‐up of RCTs, the balance of risks and benefits would be expected to change further in favour of HPV vaccination, as the reduction in long‐term adverse events due to CIN and cancer, and the complications of their treatment in the HPV vaccination arm (Kyrgiou 2017), would become more apparent.

### Quality of the evidence

The overall risk of bias for the critical outcomes ranged from some concerns to low. Outcome reporting bias (failing to report on a planned outcome) was not detected for any of the critical outcomes (see Risk of bias in included studies and Table).

Our certainty in the evidence for critical outcomes ranged from high to low certainty. We downgraded the certainty of evidence for some outcomes for limitations in design because it was not clear whether studies had concealed allocation to the different groups, there were missing data for more than 5% of participants, per‐protocol analyses were used with no data available for intention‐to‐treat populations, or studies had not planned to analyse some of the outcomes. We also downgraded some outcomes for imprecision due to wide 95% CIs that included both benefit and harm. Imprecision was mainly due to small sample sizes or few events. Finally, we downgraded some outcomes due to inconsistency when different studies in a meta‐analysis showed different directions of effect, and we could not explain the differences by examining effect modifiers and other characteristics of the studies.

### Access to CSRs

Since CSRs are more comprehensive and transparent than journal publications or online trial registry data (Jørgensen 2020a), they were chosen as the primary source of data for our review. The proportion of CSRs relating to individual studies we included varied by vaccine. Cervarix CSRs are readily available online in the GSK study register (GSK 2023), and we retrieved 25 GSK CSRs. We did not apply directly to manufacturers since accessing reports through them would significantly limit the use of the data (Jørgensen 2018b; Jørgensen 2020b). We applied to the European Medicines Agency (EMA) for CSRs and at the time of publishing this review we had received six CSRs from the EMA. We also retrieved two CSRs from Health Canada. To date, we have accessed and extracted data from 33 CSRs: 74% (25/34) of trials assessing Cervarix, 32% (9/28) of trials assessing Gardasil, 38% (3/8) of trials assessing Gardasil‐9 and none of the three trials assessing Cecolin. This has led to a higher quantity and transparency of data for Cervarix than the other vaccines. However, the CSRs available to us were heavily redacted in some sections.

### Use of published combined analyses

For some outcomes, we used data published in analyses combining data from more than one trial (4v FUTURE I/II 2010‐INT; 4v Villa/FUTURE I/II 2009‐INT; 4v Yoshikawa/NCT00411749 2013‐JPN). For critical outcomes that included pooled analyses (CIN3+ irrespective of HPV type, vaccine‐type high‐grade VIN or VaIN, and anogenital warts irrespective of HPV type), we carried out sensitivity analyses excluding the pooled analyses. No important differences were found compared with the main meta‐analyses, except wider 95% CIs in the sensitivity analyses due to fewer participants and events.

### Varying time points

We combined data from studies with different durations of follow‐up, which could introduce methodological, statistical and clinical heterogeneity into our analyses. This is most apparent in the meta‐analyses of serious adverse events where follow‐up ranged from two weeks after the last dose to eight years in the different trials. We examined whether the duration of follow‐up had any effect on the estimates. We found no pattern of gradually increasing or decreasing effect with duration of follow‐up, although some groups showed a decreased risk with HPV vaccine. Surprisingly, we found no pattern of gradually higher control group rate with increasing duration of follow‐up. We did find a higher rate in the three to four years follow‐up group and assumed this was due to different methods of defining, monitoring and collecting serious adverse events because the rate dropped back down in the ≥ 5 years follow‐up studies. We found this assumption difficult to ascertain due to inconsistent or sparse reporting on serious adverse events collection methods.

### Transitivity for serious adverse events

We had concerns about transitivity for serious adverse events due to different methods of measuring and collecting data across trials. Consequently, we did not use NMA results for serious adverse events, but report on standard meta‐analysis results. Our review of the adverse events measuring and collection methods revealed a multitude of methods and definitions (Table).

### Agreements and disagreements with other studies or reviews

A previous Cochrane review on the safety and efficacy of HPV vaccines that included evidence from RCTs concluded that *"HPV vaccines protect against cervical precancer in adolescent girls and women who are vaccinated between 15 and 26 years of age"* and that *"The vaccines do not increase the risk of serious adverse events, miscarriage or pregnancy termination"* (Arbyn 2018). We reached similar conclusions for some pre‐cancers and harms. We were able to include data from more unpublished sources, and more trials had completed by our more recent search date. We extended the generalisability of the evidence by including effects in males as well as females, and the inclusion of dose comparisons.

A systematic review of CSRs concluded that *"At 4 years follow‐up, the HPV vaccines decreased HPV‐related cancer precursors and treatment procedures"* but *"increased serious nervous system disorders (exploratory analysis) and general harms"* (Jørgensen 2020b). We reached similar conclusions regarding some benefits but not regarding serious nervous system disorders. We were able to access more CSRs and included more trials and participants. Nevertheless, very few cases of serious nervous system disorders were reported in the trials, and we did not carry out exploratory analyses to retrospectively diagnose cases. Conclusions about serious nervous system disorders could not be drawn in our review.

An overview of systematic reviews concluded that *"the available HPV vaccines are safe, effective, and efficacious against vaccine‐type HPV infection and HPV‐associated cellular changes, including precancerous and benign lesions"* (Villa 2020). This agrees with the conclusions of our review.

A recent narrative review of HPV and its role in carcinogenesis stated that *"International, randomized, controlled trials involving female adolescents and women 15 to 26 years of age have shown vaccine efficacy of at least 96% for the prevention of cervical precancers [CIN2+] owing to vaccine‐targeted HPV types in per‐protocol populations ― women who had no evidence of infection with or exposure to a given HPV type at the time of vaccination and had received all three vaccine doses."* (Markowitz 2023). This is not inconsistent with our findings, but selectively reports a subset of study participants from some of the studies (2v PATRICIA 2012‐INT; 4v FUTURE 2007‐INT). Within population immunisation programmes, HPV negativity is not a pre‐requisite for vaccination, hence why we did not include this specific subgroup in our pre‐defined analyses. As discussed, the results from our review (and the included RCTs, which included older participants and those with known and unknown hrHPV status at enrolment) likely underestimate the true magnitude of the effect of HPV vaccination in a population with high HPV naïvety (pre‐adolescents aged 9 to 13) targetted for immunisation programmes.

## Authors' conclusions

**Implications for practice**

It is likely that clinical trials of human papillomavirus (HPV) vaccination underestimate the impact on development of cervical pre‐cancer and cancer, as the incidence of cervical intraepithelial neoplasia (CIN) within clinical trials mimics well‐screened populations, due to participants undergoing surveillance to prevent cervical cancer as part of study follow‐up. HPV vaccination will prevent morbidity from screening and in those who do not have screen‐detected cancer. However, HPV vaccination is likely to have the most impact in under‐screened populations.

In May 2018, the World Health Organization (WHO) published a call to action to eliminate cervical cancer (Adhanom‐Ghebreyesus 2018) and in November 2020 published its global health strategy to achieve this aim (WHO 2023). Their strategy included a combination of primary prevention, effective screening and effective treatment. Their data demonstrated that, as of 2020, less than a quarter of low‐income countries had introduced the HPV vaccine into national immunisation schedules compared with more than 85% of high‐income countries (WHO 2023).

Effective cervical screening programmes can reduce deaths from cervical cancer (Peto 2004), but come at a significant cost, with morbidity involved in treating lesions that may not have developed into cancer. Many countries have no effective cervical screening programme, due to the costs and infrastructure required, which contribute to the differing levels of incidence of, and mortality from, cervical cancer seen worldwide. A recent Global Cancer Observatory (GLOBOCAN) analysis demonstrated that cervical cancer incidence was three‐fold higher in countries with a low Human Development Index (HDI) than in countries with very high HDI (Singh 2023). There is an even greater disparity in mortality rates, which were six‐fold higher in low HDI countries versus very high HDI countries, due to a combination of fewer cancers being screen‐detected, hence more higher‐stage disease, and lack of access to effective treatment (Singh 2023).

Longer term, as cervical cancer and high‐grade CIN rates are already falling in populations with high vaccine coverage (Falcaro 2021), there are implications for clinical practice. Reducing incidence will have knock‐on effects on service delivery and design of screening programmes (Castanon 2018; Landy 2018), and the need for consideration of further centralisation of care for those diagnosed with cervical cancer, as this becomes a rare disease. However, in populations used to frequent cervical screening, careful communication will be required to reduce anxiety due to increased screening intervals (Rickford 2023).

Studies with HPV 6 and 11 within the vaccine demonstrate good efficacy for prevention of anogenital warts. Although these are not malignant lesions, they can be the cause of significant physical disease and psychological distress, especially in immunocompromised people, be that due to HIV, medication (e.g. transplant recipients) or pregnancy. Treatment can be painful and disfiguring and primary prevention with HPV vaccination is likely to have a significant effect on disease burden.

The efficacy and cost‐efficacy of vaccination programmes are likely to differ in different age cohorts. For example, the randomised controlled trial (RCT) data in women aged over 25 years, even in studies where participants were HPV‐negative, do not show an improvement in CIN2+ rates in this cohort.

**Implications for research**

Data on the longer‐term effectiveness of single‐dose vaccine strategies are needed, although short‐term RCT data suggest that this may be similarly effective. The advantages of a single‐dose strategy include cost and coverage, so a small reduction in effectiveness may be compensated for in a cost‐effective analysis once all factors are taken into consideration.

Changes in the incidence of CIN and cervical cancer will change the cost‐benefit analysis of current screening programmes and research is required to understand how best to screen populations with high vaccine coverage, since screening intervals may be able to be significantly reduced in those who have been vaccinated as a young adolescent and are HPV‐negative on screening as young adults (Falcaro 2021). More data are needed to consider how this may best be achieved.

Other areas for research include the use of self‐sampling for HPV, especially in populations with lower prevalence and for populations where delivery of conventional cervical screening, requiring intimate examination by a health professional, may be difficult to deliver, due to lack of resources and/or social determinants. Studies to develop screening programmes for non‐cervical HPV‐dependent cancers are also needed, especially as many are increasing in incidence.

Twelve ongoing studies recruiting just under 63,000 people will be considered for inclusion in this review when their results are available.

## History

Protocol first published: Issue 5, 2022

## Risk of bias

**Table:** Risk of bias for analysis 1.2 CIN3+ irrespective of HPV type

**Study**	**Bias**											
	**Randomisation process**		**Deviations from intended interventions**		**Missing outcome data**		**Measurement of the outcome**		**Selection of the reported results**		**Overall**	
	**Authors' judgement**	**Support for judgement**	**Authors' judgement**	**Support for judgement**	**Authors' judgement**	**Support for judgement**	**Authors' judgement**	**Support for judgement**	**Authors' judgement**	**Support for judgement**	**Authors' judgement**	**Support for judgement**
**Subgroup 1.2.1 Females 14 years or younger**												
**Subgroup 1.2.2 Females 15‐25 years**												
2v CVT 2011‐CRI	Low risk of bias	Randomisation made by the Data Management Center; sequence probably random, allocation concealed.	Some concerns	Blinded study (participants and personnel or carers, or both). Per‐protocol analysis, not considered appropriate to assess effect of assignment to intervention.	Some concerns	Data were not available for all participants or nearly all participants that were randomised: 2617 analysed of 7466 randomised; 65% missing data balanced between groups.	Low risk of bias	Method of measuring outcome probably appropriate. Probable that measurement or ascertainment of outcome did not differ between groups. Outcome assessors were unaware of intervention allocations.	Some concerns	The prospective registry was available. Outcome was not prespecified. No information on whether the result was selected from multiple outcome measurements or analyses of the data.	Some concerns	We judged the study as having some concerns in at least one domain for this result, but not to be at high risk of bias for any domain.
2v Konno 2010‐JPN	Low risk of bias	Centralised internet randomisation system; sequence random, allocation concealed. Imbalances in baseline characteristics appear to be compatible with chance.	Low risk of bias	Blinded study (participants and personnel or carers, or both). Analysis was carried out on participants who received at least one dose of the intervention (total vaccinated cohort) and no participants were excluded from analysis due to protocol violations.	Some concerns	Data were not available for all participants or nearly all participants that were randomised: 927 analysed of 1046 randomised; 11% missing data balanced between groups.	Low risk of bias	Method of measuring outcome probably appropriate. Probable that measurement or ascertainment of outcome did not differ between groups. Outcome assessors were unaware of intervention allocations.	Low risk of bias	The prospective protocol and registry were available. Outcome analysed as prespecified.	Some concerns	We judged the study as having some concerns in at least one domain for this result, but not to be at high risk of bias for any domain.
2v PATRICIA 2012‐INT	Low risk of bias	Centralised internet randomisation system; sequence random, allocation concealed. Imbalances in baseline characteristics appear to be compatible with chance.	Low risk of bias	Blinded study (participants and personnel or carers, or both). Appropriate analysis: total vaccinated cohort.	Some concerns	Data were not available for all participants or nearly all participants that were randomised: 17402 analysed of 18644 randomised; 7% missing data balanced between groups.	Low risk of bias	Method of measuring outcome probably appropriate. Probable that measurement or ascertainment of outcome did not differ between groups. Outcome assessors were unaware of intervention allocations.	Low risk of bias	The protocol and registry were available, finalised before unblinding of the data. Outcome analysed as prespecified.	Some concerns	We judged the study as having some concerns in at least one domain for this result, but not to be at high risk of bias for any domain.
2v Zhu 2014‐CHNa	Low risk of bias	Centralised internet randomisation system; sequence random, allocation concealed. Baseline charcteristics were similar between groups.	Low risk of bias	Blinded study (participants and personnel or carers, or both). Appropriate analysis of participants who received at least one dose of the intervention (total vaccinated cohort).	Low risk of bias	Data available for nearly all participants randomised (5795/6051).	Low risk of bias	Method of measuring outcome probably appropriate. Probable that measurement or ascertainment of outcome did not differ between groups. Outcome assessors were unaware of intervention allocations.	Some concerns	The prospective protocol and registry were available. Protocol was modified more than once to add outcomes and timepoints not planned in the original study plan. CIN3+ was not included in protocol or amendments but first reported in 72‐month follow‐up (June 2017 clinical study report).	Some concerns	We judged the study as having some concerns in at least one domain for this result, but not to be at high risk of bias for any domain.
4v FUTURE I/II 2010‐INT	Low risk of bias	Analysis pooling two RCTs. Both studies used a computer‐generated random sequence with allocation concealed through interactive voice response system.	Low risk of bias	Both studies were blinded (participants and personnel or carers, or both). Appropriate analysis used: at least one vaccination with at least one follow‐up.	Low risk of bias	Data analysed for nearly all randomized participants (17,160/17,662).	Low risk of bias	Method of measuring outcome probably appropriate. Probable that measurement or ascertainment of outcome did not differ between groups. Outcome assessors were unaware of intervention allocations.	Some concerns	Prospective registries for the two studies were available (NCT record). None of the studies had planned to analyse this outcome.	Some concerns	We judged the study as having some concerns in at least one domain for this result, but not to be at high risk of bias for any domain.
**Subgroup 1.2.3 Females 25 years or older**

**Table:** Risk of bias for analysis 1.14 High‐grade VIN or VaIN irrespective of HPV type

**Study**	**Bias**											
	**Randomisation process**		**Deviations from intended interventions**		**Missing outcome data**		**Measurement of the outcome**		**Selection of the reported results**		**Overall**	
	**Authors' judgement**	**Support for judgement**	**Authors' judgement**	**Support for judgement**	**Authors' judgement**	**Support for judgement**	**Authors' judgement**	**Support for judgement**	**Authors' judgement**	**Support for judgement**	**Authors' judgement**	**Support for judgement**
**Subgroup 1.14.1 Females 14 years or younger**												
**Subgroup 1.14.2 Females 15‐25 years**												
2v PATRICIA 2012‐INT	Low risk of bias	Centralised internet randomisation system; sequence random, allocation concealed. Imbalances in baseline characteristics appear to be compatible with chance.	Some concerns	Blinded study (participants and personnel or carers, or both). Per‐protocol analysis, not considered appropriate to assess effect of assignment to intervention.	Some concerns	Data were not available for all participants or nearly all participants that were randomised: 15701 analysed of 18644 randomised; 16% missing data balanced between groups.	Low risk of bias	Method of measuring outcome probably appropriate. Probable that measurement or ascertainment of outcome did not differ between groups. Outcome assessors were unaware of intervention allocations.	Low risk of bias	The protocol and registry were available, finalised before unblinding of the data. Outcome analysed as prespecified.	Some concerns	We judged the study as having some concerns in at least one domain for this result, but not to be at high risk of bias for any domain.
4v FUTURE I/II 2010‐INT	Low risk of bias	Analysis pooling two RCTs. Both studies used a computer‐generated random sequence with allocation concealed through interactive voice response system.	Low risk of bias	Both studies were blinded (participants and personnel or carers, or both). Appropriate analysis used: at least one vaccination with at least one follow‐up.	Low risk of bias	Data analysed for nearly all randomized participants (17,391/17,662).	Low risk of bias	Method of measuring outcome probably appropriate. Probable that measurement or ascertainment of outcome did not differ between groups. Outcome assessors were unaware of intervention allocations.	Some concerns	Prospective registries for the two studies were available (NCT record). One of the studies had not planned to analyse this outcome.	Some concerns	We judged the study as having some concerns in at least one domain for this result, but not to be at high risk of bias for any domain.
**Subgroup 1.14.3 Females 25 years or older**

**Table:** Risk of bias for analysis 1.18 Anogenital warts irrespective of HPV type

**Study**	**Bias**											
	**Randomisation process**		**Deviations from intended interventions**		**Missing outcome data**		**Measurement of the outcome**		**Selection of the reported results**		**Overall**	
	**Authors' judgement**	**Support for judgement**	**Authors' judgement**	**Support for judgement**	**Authors' judgement**	**Support for judgement**	**Authors' judgement**	**Support for judgement**	**Authors' judgement**	**Support for judgement**	**Authors' judgement**	**Support for judgement**
**Subgroup 1.18.1 Females 14 years or younger**												
**Subgroup 1.18.2 Females 15‐25 years**												
4v FUTURE I/II 2010‐INT	Low risk of bias	Analysis pooling two RCTs. Both studies used a computer‐generated random sequence with allocation concealed through interactive voice response system.	Low risk of bias	Both studies were blinded (participants and personnel or carers, or both). Appropriate analysis used: at least one vaccination with at least 1 follow‐up.	Low risk of bias	Data analysed for nearly all randomized participants (17,391/17,662).	Low risk of bias	Method of measuring outcome probably appropriate. Probable that measurement or ascertainment of outcome did not differ between groups. Outcome assessors were unaware of intervention allocations.	Some concerns	Prospective registries for the two studies were available (NCT record). One of the studies had not planned to analyse this outcome.	Some concerns	We judged the study as having some concerns in at least one domain for this result, but not to be at high risk of bias for any domain.
**Subgroup 1.18.3 Females 25 years or older**												
**Subgroup 1.18.4 Females and males**												
**Subgroup 1.18.5 Males 15‐25 years**												
4v Giuliano 2011‐INT	Low risk of bias	Central randomisation; sequence random, allocation concealed. Imbalances in baseline characteristics appear to be compatible with chance.	Low risk of bias	Blinded study (participants and personnel or carers, or both). Appropriate analysis (full analysis set).	Low risk of bias	Data available for nearly all participants randomised (3880/4065).	Low risk of bias	Method of measuring outcome probably appropriate. Probable that measurement or ascertainment of outcome did not differ between groups. Outcome assessors were unaware of intervention allocations.	Low risk of bias	The prospective protocol and registry were available. Outcome analysed as prespecified.	Low risk of bias	We judged the study to be at low risk of bias for all domains for this result.

**Table:** Risk of bias for analysis 1.19 Anogenital warts associated with vaccine‐matched HPV types

**Study**	**Bias**											
	**Randomisation process**		**Deviations from intended interventions**		**Missing outcome data**		**Measurement of the outcome**		**Selection of the reported results**		**Overall**	
	**Authors' judgement**	**Support for judgement**	**Authors' judgement**	**Support for judgement**	**Authors' judgement**	**Support for judgement**	**Authors' judgement**	**Support for judgement**	**Authors' judgement**	**Support for judgement**	**Authors' judgement**	**Support for judgement**
**Subgroup 1.19.1 Females 14 years or younger**												
**Subgroup 1.19.2 Females 15‐25 years**												
4v FUTURE I/II 2010‐INT	Low risk of bias	Analysis pooling two RCTs. Both studies used a computer‐generated random sequence with allocation concealed through interactive voice response system.	Low risk of bias	Both studies were blinded (participants and personnel or carers, or both). Appropriate analysis used: at least one vaccination with at least one follow‐up.	Low risk of bias	Data analysed for nearly all randomized participants (17,391/17,662).	Low risk of bias	Method of measuring outcome probably appropriate. Probable that measurement or ascertainment of outcome did not differ between groups. Outcome assessors were unaware of intervention allocations.	Some concerns	Prospective registries for the two studies were available (NCT record). One of the studies had not planned to analyse this outcome.	Some concerns	We judged the study as having some concerns in at least one domain for this result, but not to be at high risk of bias for any domain.
4v Villa 2005‐INT	Some concerns	Randomisation schedules were computer generated by use of a blocking factor of eight; sequence random, no information on allocation concealment. Imbalances in baseline characteristics appear to be compatible with chance.	Some concerns	Blinded study (participants and personnel or carers, or both). Per‐protocol analysis was used (in the study it was labelled modified ITT) that included participants who were seronegative and DNA negative to the relevant HPV type at enrolment and who had had at least one vaccination. Per‐protocol analysis is not considered appropriate to assess effect of assignment to intervention.	Low risk of bias	Data available for nearly all participants randomised (526/552).	Low risk of bias	Method of measuring outcome probably appropriate. Probable that measurement or ascertainment of outcome did not differ between groups. Outcome assessors were unaware of intervention allocations.	Some concerns	The study was registered six years after commencement. No information on whether the result was selected from multiple outcome measurements or analyses of the data.	Some concerns	We judged the study as having some concerns in at least one domain for this result, but not to be at high risk of bias for any domain.
**Subgroup 1.19.3 Females 25 years or older**												
4v FUTURE III 2009‐INT	Low risk of bias	Computer‐generated allocation schedule and an interactive voice response system; sequence random, allocation concealed. Groups similar for baseline characteristics.	Low risk of bias	Blinded study (participants and personnel or carers, or both). Appropriate analysis used (participants who received at least one dose and returned for follow‐up).	Some concerns	Data were not available for all participants or nearly all participants that were randomised: 3382 analysed of 3819 randomised; 11% missing data balanced between groups.	Low risk of bias	Method of measuring outcome probably appropriate. Probable that measurement or ascertainment of outcome did not differ between groups. Outcome assessors were unaware of intervention allocations.	Low risk of bias	The prospective registry was available. Outcome analysed as prespecified.	Some concerns	We judged the study as having some concerns in at least one domain for this result, but not to be at high risk of bias for any domain.
**Subgroup 1.19.4 Females and males**												
**Subgroup 1.19.5 Males 15‐25 years**												
4v Giuliano 2011‐INT	Low risk of bias	Central randomisation; sequence random, allocation concealed. Imbalances in baseline characteristics appear to be compatible with chance.	Low risk of bias	Blinded study (participants and personnel or carers, or both). Appropriate analysis (full analysis set).	Low risk of bias	Data available for nearly all participants randomised (3880/4065).	Low risk of bias	Method of measuring outcome probably appropriate. Probable that measurement or ascertainment of outcome did not differ between groups. Outcome assessors were unaware of intervention allocations.	Low risk of bias	The prospective protocol and registry were available. Outcome analysed as prespecified.	Low risk of bias	We judged the study to be at low risk of bias for all domains for this result.

**Table:** Risk of bias for analysis 2.1 CIN3+ irrespective of HPV type

**Study**	**Bias**											
	**Randomisation process**		**Deviations from intended interventions**		**Missing outcome data**		**Measurement of the outcome**		**Selection of the reported results**		**Overall**	
	**Authors' judgement**	**Support for judgement**	**Authors' judgement**	**Support for judgement**	**Authors' judgement**	**Support for judgement**	**Authors' judgement**	**Support for judgement**	**Authors' judgement**	**Support for judgement**	**Authors' judgement**	**Support for judgement**
**Subgroup 2.1.1 Females 14 years or younger**												
**Subgroup 2.1.2 Females 15‐25 years**												
2v CVT 2011‐CRI	Low risk of bias	Randomisation made by the Data Management Center; sequence probably random, allocation concealed.	Some concerns	Blinded study (participants and personnel or carers, or both). Per‐protocol analysis, not considered appropriate to assess effect of assignment to intervention.	Some concerns	Data were not available for all participants or nearly all participants that were randomised: 2617 analysed of 7466 randomised; 65% missing data balanced between groups.	Low risk of bias	Method of measuring outcome probably appropriate. Probable that measurement or ascertainment of outcome did not differ between groups. Outcome assessors were unaware of intervention allocations.	Some concerns	The prospective registry was available. Outcome was not prespecified. No information on whether the result was selected from multiple outcome measurements or analyses of the data.	Some concerns	We judged the study as having some concerns in at least one domain for this result, but not to be at high risk of bias for any domain.
2v Konno 2010‐JPN	Low risk of bias	Centralised internet randomisation system; sequence random, allocation concealed. Imbalances in baseline characteristics appear to be compatible with chance.	Low risk of bias	Blinded study (participants and personnel or carers, or both). Analysis was carried out on participants who received at least one dose of the intervention (total vaccinated cohort) and no participants were excluded from analysis due to protocol violations.	Some concerns	Data were not available for all participants or nearly all participants that were randomised: 927 analysed of 1046 randomised; 11% missing data balanced between groups.	Low risk of bias	Method of measuring outcome probably appropriate. Probable that measurement or ascertainment of outcome did not differ between groups. Outcome assessors were unaware of intervention allocations.	Low risk of bias	The prospective protocol and registry were available. Outcome analysed as prespecified.	Some concerns	We judged the study as having some concerns in at least one domain for this result, but not to be at high risk of bias for any domain.
2v PATRICIA 2012‐INT	Low risk of bias	Centralised internet randomisation system; sequence random, allocation concealed. Imbalances in baseline characteristics appear to be compatible with chance.	Low risk of bias	Blinded study (participants and personnel or carers, or both). Appropriate analysis: total vaccinated cohort.	Some concerns	Data were not available for all participants or nearly all participants that were randomised: 17402 analysed of 18644 randomised; 7% missing data balanced between groups.	Low risk of bias	Method of measuring outcome probably appropriate. Probable that measurement or ascertainment of outcome did not differ between groups. Outcome assessors were unaware of intervention allocations.	Low risk of bias	The protocol and registry were available, finalised before unblinding of the data. Outcome analysed as prespecified.	Some concerns	We judged the study as having some concerns in at least one domain for this result, but not to be at high risk of bias for any domain.
2v Zhu 2014‐CHNa	Low risk of bias	Centralised internet randomisation system; sequence random, allocation concealed. Baseline charcteristics were similar between groups.	Low risk of bias	Blinded study (participants and personnel or carers, or both). Appropriate analysis of participants who received at least one dose of the intervention (total vaccinated cohort).	Low risk of bias	Data available for nearly all participants randomised (5795/6051).	Low risk of bias	Method of measuring outcome probably appropriate. Probable that measurement or ascertainment of outcome did not differ between groups. Outcome assessors were unaware of intervention allocations.	Some concerns	The prospective protocol and registry were available. Protocol was modified more than once to add outcomes and timepoints not planned in the original study plan. CIN3+ was not included in protocol or amendments but first reported in 72‐month follow‐up (June 2017 clinical study report).	Some concerns	We judged the study as having some concerns in at least one domain for this result, but not to be at high risk of bias for any domain.
**Subgroup 2.1.3 Females 25 years or older**

**Table:** Risk of bias for analysis 2.2 CIN3+ associated with HPV 16 and/or 18

**Study**	**Bias**											
	**Randomisation process**		**Deviations from intended interventions**		**Missing outcome data**		**Measurement of the outcome**		**Selection of the reported results**		**Overall**	
	**Authors' judgement**	**Support for judgement**	**Authors' judgement**	**Support for judgement**	**Authors' judgement**	**Support for judgement**	**Authors' judgement**	**Support for judgement**	**Authors' judgement**	**Support for judgement**	**Authors' judgement**	**Support for judgement**
**Subgroup 2.2.1 Females 14 years or younger**												
**Subgroup 2.2.2 Females 15‐25 years**												
2v Harper 2004‐BRA/NA	Low risk of bias	Centralised internet randomisation system; sequence random, allocation concealed. Imbalances in baseline characteristics appear to be compatible with chance.	Low risk of bias	Blinded study (participants and personnel or carers, or both). Appropriate analysis: total vaccinated cohort.	Low risk of bias	Data available for all 1113 randomised participants.	Low risk of bias	Method of measuring outcome probably appropriate. Probable that measurement or ascertainment of outcome did not differ between groups. Outcome assessors were unaware of intervention allocations.	Low risk of bias	The prospective protocol and registry were available. Outcome analysed as prespecified.	Low risk of bias	We judged the study to be at low risk of bias for all domains for this result.
2v PATRICIA 2012‐INT	Low risk of bias	Centralised internet randomisation system; sequence random, allocation concealed. Imbalances in baseline characteristics appear to be compatible with chance.	Low risk of bias	Blinded study (participants and personnel or carers, or both). Appropriate analysis: total vaccinated cohort.	Some concerns	Data were not available for all participants or nearly all participants that were randomised: 17402 analysed of 18644 randomised; 7% missing data balanced between groups.	Low risk of bias	Method of measuring outcome probably appropriate. Probable that measurement or ascertainment of outcome did not differ between groups. Outcome assessors were unaware of intervention allocations.	Low risk of bias	The protocol and registry were available, finalised before unblinding of the data. Outcome analysed as prespecified.	Some concerns	We judged the study as having some concerns in at least one domain for this result, but not to be at high risk of bias for any domain.
**Subgroup 2.2.3 Females 25 years or older**

**Table:** Risk of bias for analysis 2.9 High‐grade VIN or VaIN irrespective of HPV type

**Study**	**Bias**											
	**Randomisation process**		**Deviations from intended interventions**		**Missing outcome data**		**Measurement of the outcome**		**Selection of the reported results**		**Overall**	
	**Authors' judgement**	**Support for judgement**	**Authors' judgement**	**Support for judgement**	**Authors' judgement**	**Support for judgement**	**Authors' judgement**	**Support for judgement**	**Authors' judgement**	**Support for judgement**	**Authors' judgement**	**Support for judgement**
**Subgroup 2.9.1 Females 14 years or younger**												
**Subgroup 2.9.2 Females 15‐25 years**												
2v PATRICIA 2012‐INT	Low risk of bias	Centralised internet randomisation system; sequence random, allocation concealed. Imbalances in baseline characteristics appear to be compatible with chance.	Some concerns	Blinded study (participants and personnel or carers, or both). Per‐protocol analysis, not considered appropriate to assess effect of assignment to intervention.	Some concerns	Data were not available for all participants or nearly all participants that were randomised: 15701 analysed of 18644 randomised; 16% missing data balanced between groups.	Low risk of bias	Method of measuring outcome probably appropriate. Probable that measurement or ascertainment of outcome did not differ between groups. Outcome assessors were unaware of intervention allocations.	Low risk of bias	The protocol and registry were available, finalised before unblinding of the data. Outcome analysed as prespecified.	Some concerns	We judged the study as having some concerns in at least one domain for this result, but not to be at high risk of bias for any domain.
**Subgroup 2.9.3 Females 25 years or older**

**Table:** Risk of bias for analysis 2.10 High‐grade VIN or VaIN associated with HPV 16 and/or 18

**Study**	**Bias**											
	**Randomisation process**		**Deviations from intended interventions**		**Missing outcome data**		**Measurement of the outcome**		**Selection of the reported results**		**Overall**	
	**Authors' judgement**	**Support for judgement**	**Authors' judgement**	**Support for judgement**	**Authors' judgement**	**Support for judgement**	**Authors' judgement**	**Support for judgement**	**Authors' judgement**	**Support for judgement**	**Authors' judgement**	**Support for judgement**
**Subgroup 2.10.1 Females 14 years or younger**												
**Subgroup 2.10.2 Females 15‐25 years**												
2v PATRICIA 2012‐INT	Low risk of bias	Centralised internet randomisation system; sequence random, allocation concealed. Imbalances in baseline characteristics appear to be compatible with chance.	Some concerns	Blinded study (participants and personnel or carers, or both). Per‐protocol analysis, not considered appropriate to assess effect of assignment to intervention.	Some concerns	Data were not available for all participants or nearly all participants that were randomised: 15566 analysed of 18644 randomised; 17% missing data balanced between groups.	Low risk of bias	Method of measuring outcome probably appropriate. Probable that measurement or ascertainment of outcome did not differ between groups. Outcome assessors were unaware of intervention allocations.	Low risk of bias	The protocol and registry were available, finalised before unblinding of the data. Outcome analysed as prespecified.	Some concerns	We judged the study as having some concerns in at least one domain for this result, but not to be at high risk of bias for any domain.
**Subgroup 2.10.3 Females 25 years or older**

**Table:** Risk of bias for analysis 2.11 Serious adverse events

**Study**	**Bias**											
	**Randomisation process**		**Deviations from intended interventions**		**Missing outcome data**		**Measurement of the outcome**		**Selection of the reported results**		**Overall**	
	**Authors' judgement**	**Support for judgement**	**Authors' judgement**	**Support for judgement**	**Authors' judgement**	**Support for judgement**	**Authors' judgement**	**Support for judgement**	**Authors' judgement**	**Support for judgement**	**Authors' judgement**	**Support for judgement**
**Subgroup 2.11.1 Females 14 years or younger**												
2v Kim 2010‐KOR	Low risk of bias	Central randomisation system on internet; sequence random, allocation concealed. Imbalances in baseline characteristics appear to be compatible with chance.	Low risk of bias	Blinded study (participants and personnel or carers, or both). Appropriate analysis: no participant cross‐over, analysis performed using the total vaccinated cohort.	Low risk of bias	Data available for nearly all participants randomised (319/321).	Low risk of bias	Method of measuring outcome probably appropriate. Probable that measurement or ascertainment of outcome did not differ between groups. Outcome assessors were unaware of intervention allocations.	Low risk of bias	The prospective protocol/registry was available. Outcome analysed as prespecified.	Low risk of bias	We judged the study to be at low risk of bias for all domains for this result.
2v Lin 2018‐LA	Low risk of bias	Central randomisation software was used; sequence random, allocation concealed. Imbalances in baseline characteristics appear to be compatible with chance.	Low risk of bias	Partially blinded study (participants). Appropriate analysis: no participant cross‐over, analysis performed of participants who received at least one dose: total vaccinated cohort.	Low risk of bias	Data available for all 148 randomised participants.	Some concerns	Method of measuring outcome probably appropriate. Unblinded study (outcome assessor). This outcome requires clinical judgement and could be affected by knowledge of intervention assignment.	Low risk of bias	The prospective protocol and trial registries were available (NCT record, EUDR record, protocol in CSR). Outcome analysed as prespecified.	Some concerns	We judged the study as having some concerns in at least one domain for this result, but not to be at high risk of bias for any domain.
2v Medina 2010‐INT	Low risk of bias	Internet‐based randomisation system was used (SBIR); sequence random, allocation concealed. Imbalances in baseline characteristics appear to be compatible with chance.	Low risk of bias	Blinded study (participants and personnel or carers, or both). Appropriate analysis: total vaccinated cohort.	Low risk of bias	Data available for all 2067 participants randomised.	Low risk of bias	Method of measuring outcome probably appropriate. Probable that measurement or ascertainment of outcome did not differ between groups. Outcome assessors were unaware of intervention allocations.	Low risk of bias	The prospective protocol/registry was available. Outcome analysed as prespecified.	Low risk of bias	We judged the study to be at low risk of bias for all domains for this result.
2v Pedersen 2012‐NA/EU	Low risk of bias	Central randomisation system on internet (SBIR); sequence random; allocation concealed. Imbalances in baseline characteristics appear to be compatible with chance.	Low risk of bias	Unblinded study (participants and personnel or carers, or both). No participant cross‐over; deviations did probably not arise because of the trial context. Appropriate analysis: total vaccinated cohort.	Low risk of bias	All 813 randomised participants were analysed for serious adverse events.	Some concerns	Method of measuring outcome probably appropriate. Unblinded study (outcome assessor). This outcome requires clinical judgement and could be affected by knowledge of intervention assignment.	Low risk of bias	Prospective protocol and registry were available. Unclear if the final safety timepoint (month 12) was prespecified or not. However, we do not consider the reporting of this outcome to be selective since serious adverse events should be reported in all clinical trials for full duration of the study.	Some concerns	We judged the study as having some concerns in at least one domain for this result, but not to be at high risk of bias for any domain.
2v Schmeink 2011‐NLD/SWE	Low risk of bias	Computer‐generated list with central randomisation system on the internet; sequence random, allocation concealed. Imbalances in baseline characteristics appear to be compatible with chance.	Low risk of bias	Unblinded study (participants and personnel or carers, or both). No participant cross‐over; deviations did probably not arise because of the trial context. Appropriate analysis: ITT.	Low risk of bias	Data available for all 741 randomised participants.	Some concerns	Method of measuring outcome probably appropriate. Unblinded study (outcome assessor). This outcome requires clinical judgement and could be affected by knowledge of intervention assignment.	Low risk of bias	The prospective protocol and registry were available. Outcome analysed as prespecified.	Some concerns	We judged the study as having some concerns in at least one domain for this result, but not to be at high risk of bias for any domain.
2v Zhu 2014‐CHNb	Low risk of bias	A central randomisation system on internet (SBIR) was used; sequence random, allocation concealed. No information about imbalances in baseline characteristics.	Low risk of bias	Blinded study (participants and personnel or carers, or both). Appropriate analysis: total vaccinated cohort.	Low risk of bias	Data available for nearly all participants randomised (749/750).	Low risk of bias	Method of measuring outcome probably appropriate. Probable that measurement or ascertainment of outcome did not differ between groups. Outcome assessors were unaware of intervention allocations.	Low risk of bias	The prospective protocol or registry was available. Outcome analysed as prespecified	Low risk of bias	We judged the study to be at low risk of bias for all domains for this result.
**Subgroup 2.11.2 Females 15‐25 years**												
2v Bhatla 2010‐IND	Low risk of bias	Computer‐generated random sequence. Allocation probably concealed through numbers distributed by the study monitor. Imbalances in baseline characteristics appear to be compatible with chance.	Low risk of bias	Blinded study (participants and personnel or carers, or both). Appropriate analysis: total vaccinated cohort.	Low risk of bias	Data available for all 354 randomised participants.	Low risk of bias	Method of measuring outcome probably appropriate. Probable that measurement or ascertainment of outcome did not differ between groups. Outcome assessors were unaware of intervention allocations.	Low risk of bias	The prospective protocol and registry were available (trial record and analysis plan). Outcome analysed as prespecified.	Low risk of bias	We judged the study to be at low risk of bias for all domains for this result.
2v CVT 2011‐CRI	Low risk of bias	Randomisation made by the Data Management Center; sequence probably random, allocation concealed.	Low risk of bias	Blinded study (participants and personnel or carers, or both). Appropriate analysis: total vaccinated cohort.	Low risk of bias	Data available for all 7466 randomised participants.	Low risk of bias	Method of measuring outcome probably appropriate. Probable that measurement or ascertainment of outcome did not differ between groups. Outcome assessors were unaware of intervention allocations.	Low risk of bias	The prospective registry was available. Outcome analysed as prespecified.	Low risk of bias	We judged the study to be at low risk of bias for all domains for this result.
2v Harper 2004‐BRA/NA	Low risk of bias	Centralised internet randomisation system; sequence random, allocation concealed. Imbalances in baseline characteristics appear to be compatible with chance.	Low risk of bias	Blinded study (participants and personnel or carers, or both). Appropriate analysis: total vaccinated cohort.	Low risk of bias	Data available for all 1113 randomised participants.	Low risk of bias	Method of measuring outcome probably appropriate. Probable that measurement or ascertainment of outcome did not differ between groups. Outcome assessors were unaware of intervention allocations.	Low risk of bias	The prospective protocol was available. Outcome analysed as prespecified.	Low risk of bias	We judged the study to be at low risk of bias for all domains for this result.
2v Kim 2011‐KOR	Low risk of bias	Centralised internet randomisation system; sequence random, allocation concealed. Imbalances in baseline characteristics appear to be compatible with chance.	Low risk of bias	Blinded study (participants and personnel or carers, or both). Appropriate analysis: ITT.	Low risk of bias	Data available for all 225 randomised participants.	Low risk of bias	Method of measuring outcome probably appropriate. Probable that measurement or ascertainment of outcome did not differ between groups. Outcome assessors were unaware of intervention allocations.	Low risk of bias	The prospective protocol and registry were available. Outcome analysed as prespecified.	Low risk of bias	We judged the study to be at low risk of bias for all domains for this result.
2v Konno 2010‐JPN	Low risk of bias	Centralised internet randomisation system; sequence random, allocation concealed. Imbalances in baseline characteristics appear to be compatible with chance.	Low risk of bias	Blinded study (participants and personnel or carers, or both). Analysis was carried out on participants who received at least one dose of the intervention (total vaccinated cohort) and no participants were excluded from analysis due to protocol violations.	Low risk of bias	Data available for nearly all participants randomised (1040/1046).	Low risk of bias	Method of measuring outcome probably appropriate. Probable that measurement or ascertainment of outcome did not differ between groups. Outcome assessors were unaware of intervention allocations.	Low risk of bias	The prospective protocol and registry were available. Outcome analysed as prespecified.	Low risk of bias	We judged the study to be at low risk of bias for all domains for this result.
2v Leroux‐Roels 2011‐BEL	Low risk of bias	Central randomisation system used; sequence random, allocation concealed. Imbalances in baseline characteristics appear to be compatible with chance.	Low risk of bias	Unblinded study (participants and personnel or carers, or both). No participant cross‐over; deviations did probably not arise because of the trial context. Appropriate analysis used: ITT.	Low risk of bias	Data available for nearly all participants randomised (151/152).	Some concerns	Method of measuring outcome probably appropriate. Unblinded study (outcome assessor). This outcome requires clinical judgement and could be affected by knowledge of intervention assignment.	Low risk of bias	The prospective protocol and registry (NCT record) were available. Outcome analysed as prespecified.	Some concerns	We judged the study as having some concerns in at least one domain for this result, but not to be at high risk of bias for any domain.
2v Lim 2014‐MYS	Low risk of bias	Internet randomisation http://www.randomize.net (Ottawa, ON, Canada); sequence random, allocation probably concealed.	Low risk of bias	Blinded study (participants and personnel or carers, or both). Appropriate analysis of those who received at least one dose of the intervention.	Low risk of bias	Data available for nearly all participants randomised (267/271).	Low risk of bias	Method of measuring outcome probably appropriate. Probable that measurement or ascertainment of outcome did not differ between groups. Outcome assessors were unaware of intervention allocations.	Low risk of bias	The prospective protocol and registry were available. Outcome analysed as prespecified.	Low risk of bias	We judged the study to be at low risk of bias for all domains for this result.
2v Ngan 2010‐HKG	Low risk of bias	Central randomisation system on internet (SBIR); sequence random, allocation concealed. Imbalances in baseline characteristics appear to be compatible with a chance.	Low risk of bias	Blinded study (participants and personnel or carers, or both). Appropriate analysis: total vaccinated cohort.	Low risk of bias	Data available for all 300 randomised participants.	Low risk of bias	Method of measuring outcome probably appropriate. Probable that measurement or ascertainment of outcome did not differ between groups. Outcome assessors were unaware of intervention allocations.	Low risk of bias	The prospective protocol and registry were available. Outcome analysed as prespecified.	Low risk of bias	We judged the study to be at low risk of bias for all domains for this result.
2v PATRICIA 2012‐INT	Low risk of bias	Centralised internet randomisation system; sequence random, allocation concealed. Imbalances in baseline characteristics appear to be compatible with chance.	Low risk of bias	Blinded study (participants and personnel or carers, or both). Appropriate analysis: total vaccinated cohort.	Low risk of bias	Data analysed for all 18644 randomised participants.	Low risk of bias	Method of measuring outcome probably appropriate. Probable that measurement or ascertainment of outcome did not differ between groups. Outcome assessors were unaware of intervention allocations.	Low risk of bias	The protocol and registry were available, finalised before unblinding of the data. Outcome analysed as prespecified.	Low risk of bias	We judged the study to be at low risk of bias for all domains for this result.
2v Sow 2013‐SEN/TZN	Low risk of bias	Computer‐generated sequence with assignment through internet‐based system: sequence random, allocation concealed. Imbalances in baseline characteristics appear to be compatible with chance.	Low risk of bias	Blinded study (participants and personnel or carers, or both). Appropriate ITT analysis was conducted for safety outcomes.	Low risk of bias	Data available for all 676 randomised participants.	Low risk of bias	Method of measuring outcome probably appropriate. Probable that measurement or ascertainment of outcome did not differ between groups. Outcome assessors were unaware of intervention allocations.	Low risk of bias	The prospective protocol and registry were available. Outcome analysed as prespecified.	Low risk of bias	We judged the study to be at low risk of bias for all domains for this result.
2v Zhu 2014‐CHNa	Low risk of bias	Centralised internet randomisation system; sequence random, allocation concealed. Baseline charcteristics were similar between groups.	Low risk of bias	Blinded study (participants and personnel or carers, or both). Appropriate analysis of participants who received at least one dose of the intervention (total vaccinated cohort).	Low risk of bias	All 6051 randomised participants were analysed.	Low risk of bias	Method of measuring outcome probably appropriate. Probable that measurement or ascertainment of outcome did not differ between groups. Outcome assessors were unaware of intervention allocations.	Low risk of bias	The prospective protocol and registry were available. Outcome analysed as prespecified.	Low risk of bias	We judged the study to be at low risk of bias for all domains for this result.
2v9v KENSHE 2021‐KEN	Low risk of bias	Internet randomisation (http://www.randomize.net); sequence random, allocation probably concealed. Comparable baseline characteristics between the groups.	Low risk of bias	Blinded study (participants and personnel or carers, or both). Appropriate analysis used: ITT.	Low risk of bias	Data analysed for all 2275 randomised participants.	Low risk of bias	Method of measuring outcome probably appropriate. Probable that measurement or ascertainment of outcome did not differ between groups. Outcome assessors were unaware of intervention allocations.	Low risk of bias	The prospective registry and protocol were available. Outcome analysed as prespecified.	Low risk of bias	We judged the study to be at low risk of bias for all domains for this result.
**Subgroup 2.11.3 Females 25 years or older**												
2v VIVIANE 2014‐INT	Low risk of bias	Randomisation was internet based, list generated with SAS; sequence random, allocation concealed. Imbalances in baseline characteristics appear to be compatible with chance.	Low risk of bias	Blinded study (participants and personnel or carers, or both). Appropriate analysis: total vaccinated cohort.	Low risk of bias	All 5747 randomised participants were analysed.	Low risk of bias	Method of measuring outcome probably appropriate. Probable that measurement or ascertainment of outcome did not differ between groups. Outcome assessors were unaware of intervention allocations.	Low risk of bias	The prospective protocol, registry, and statistical analysis plan were available. Outcome analysed as prespecified.	Low risk of bias	We judged the study to be at low risk of bias for all domains for this result.
2v Zhu 2014‐CHNc	Low risk of bias	Central randomisation system on internet (SBIR); sequence random, allocation concealed. No information about imbalances in baseline characteristics	Low risk of bias	Partially blinded study (participants). No participant cross‐over; deviations did probably not arise because of the trial context. Appropriate analysis: total vaccinated cohort.	Low risk of bias	All 1212 randomised participants were analysed.	Low risk of bias	Method of measuring outcome probably appropriate. Probable that measurement or ascertainment of outcome did not differ between groups. Outcome assessors were unaware of intervention allocations.	Low risk of bias	The prospective protocol and registry were available. Outcome analysed as prespecified.	Low risk of bias	We judged the study to be at low risk of bias for all domains for this result.
**Subgroup 2.11.4 Females and males**												
**Subgroup 2.11.5 Males 15‐25 years**												
2v Petaja 2009‐FIN	Low risk of bias	Randomisation using a standard SAS program; sequence random, allocation probably concealed. Imbalances in baseline characteristics appear to be compatible with chance.	Low risk of bias	Blinded study (particpants and personnel or carers, or both). Appropriate analysis: total vaccinated cohort.	Low risk of bias	Data available for all 270 participants randomised.	Low risk of bias	Method of measuring outcome probably appropriate. Probable that measurement or ascertainment of outcome did not differ between groups. Outcome assessors were unaware of intervention allocations.	Low risk of bias	The prospective protocol and registry were available. Outcome analysed as prespecified.	Low risk of bias	We judged the study to be at low risk of bias for all domains for this result.

**Table:** Risk of bias for analysis 3.5 Invasive cervical cancer

**Study**	**Bias**											
	**Randomisation process**		**Deviations from intended interventions**		**Missing outcome data**		**Measurement of the outcome**		**Selection of the reported results**		**Overall**	
	**Authors' judgement**	**Support for judgement**	**Authors' judgement**	**Support for judgement**	**Authors' judgement**	**Support for judgement**	**Authors' judgement**	**Support for judgement**	**Authors' judgement**	**Support for judgement**	**Authors' judgement**	**Support for judgement**
**Subgroup 3.5.1 Females 14 years or younger**												
**Subgroup 3.5.2 Females 15‐25 years**												
4v FUTURE 2007‐INT	Low risk of bias	Computer‐generated random sequence; allocation concealed through interactive voice response system. Imbalances in baseline characteristics appear to be compatible with chance.	Low risk of bias	Blinded study (participants and personnel or carers, or both). Appropriate analysis used: ITT.	Low risk of bias	Data analysed for all 5455 randomised participants.	Low risk of bias	Method of measuring outcome probably appropriate. Probable that measurement or ascertainment of outcome did not differ between groups. Outcome assessors were unaware of intervention allocations.	Low risk of bias	The prospective registry was available (NCT record). Outcome analysed as prespecified.	Low risk of bias	We judged the study to be at low risk of bias for all domains for this result.
4v FUTURE II 2007‐INT	Low risk of bias	Computer‐generated random sequence with concealed allocation using an interactive voice response system. Imbalances in baseline characteristics appear to be compatible with chance.	Low risk of bias	Blinded study (participants and personnel or carers, or both). Appropriate analysis: ITT.	Low risk of bias	Data analysed for all 12167 randomised participants.	Low risk of bias	Method of measuring outcome probably appropriate. Probable that measurement or ascertainment of outcome did not differ between groups. Outcome assessors were unaware of intervention allocations.	Low risk of bias	The prospective registry was available. Outcome analysed as prespecified.	Low risk of bias	We judged the study to be at low risk of bias for all domains for this result.
**Subgroup 3.5.3 Females 25 years or older**

**Table:** Risk of bias for analysis 3.15 Invasive anal cancer

**Study**	**Bias**											
	**Randomisation process**		**Deviations from intended interventions**		**Missing outcome data**		**Measurement of the outcome**		**Selection of the reported results**		**Overall**	
	**Authors' judgement**	**Support for judgement**	**Authors' judgement**	**Support for judgement**	**Authors' judgement**	**Support for judgement**	**Authors' judgement**	**Support for judgement**	**Authors' judgement**	**Support for judgement**	**Authors' judgement**	**Support for judgement**
**Subgroup 3.15.1 Females 14 years or younger**												
**Subgroup 3.15.2 Females 15‐25 years**												
**Subgroup 3.15.3 Females 25 years or older**												
**Subgroup 3.15.4 Females and males**												
**Subgroup 3.15.5 Males 15‐25 years**												
4v Giuliano 2011‐INT	Low risk of bias	Central randomisation; sequence random, allocation concealed. Imbalances in baseline characteristics appear to be compatible with chance.	Low risk of bias	Blinded study (participants and personnel or carers, or both). Appropriate analysis (full analysis set).	Low risk of bias	4065 participants randomised; planned MSM subgroup of 551 analysed. Data available for nearly all participants randomised within the planned subgroup.	Low risk of bias	Method of measuring outcome probably appropriate. Probable that measurement or ascertainment of outcome did not differ between groups. Outcome assessors were unaware of intervention allocations.	Low risk of bias	The prospective protocol and registry were available. Outcome analysed as prespecified for this subgroup.	Low risk of bias	We judged the study to be at low risk of bias for all domains for this result.

**Table:** Risk of bias for analysis 3.18 Invasive penile cancer

**Study**	**Bias**											
	**Randomisation process**		**Deviations from intended interventions**		**Missing outcome data**		**Measurement of the outcome**		**Selection of the reported results**		**Overall**	
	**Authors' judgement**	**Support for judgement**	**Authors' judgement**	**Support for judgement**	**Authors' judgement**	**Support for judgement**	**Authors' judgement**	**Support for judgement**	**Authors' judgement**	**Support for judgement**	**Authors' judgement**	**Support for judgement**
**Subgroup 3.18.1 Males 15‐25 years**												
4v Giuliano 2011‐INT	Low risk of bias	Central randomisation; sequence random, allocation concealed. Imbalances in baseline characteristics appear to be compatible with chance.	Low risk of bias	Blinded study (participants and personnel or carers, or both). Appropriate analysis (full analysis set).	Low risk of bias	Data available for nearly all participants randomised (3880/4065).	Low risk of bias	Method of measuring outcome probably appropriate. Probable that measurement or ascertainment of outcome did not differ between groups. Outcome assessors were unaware of intervention allocations.	Low risk of bias	The prospective protocol and registry were available. Outcome analysed as prespecified.	Low risk of bias	We judged the study to be at low risk of bias for all domains for this result.

**Table:** Risk of bias for analysis 3.21 Serious adverse events

**Study**	**Bias**											
	**Randomisation process**		**Deviations from intended interventions**		**Missing outcome data**		**Measurement of the outcome**		**Selection of the reported results**		**Overall**	
	**Authors' judgement**	**Support for judgement**	**Authors' judgement**	**Support for judgement**	**Authors' judgement**	**Support for judgement**	**Authors' judgement**	**Support for judgement**	**Authors' judgement**	**Support for judgement**	**Authors' judgement**	**Support for judgement**
**Subgroup 3.21.1 Females 14 years or younger**												
4v Mugo 2015‐AF	Some concerns	Trial declared as randomised, but no additional information provided; sequence probably random, no information on allocation concealment. No clear information about baseline imbalances.	Low risk of bias	Blinded study (participants and personnel or carers, or both). Appropriate analysis: total vaccinated cohort.	Low risk of bias	Data available for nearly all participants randomised (98/100).	Low risk of bias	Method of measuring outcome probably appropriate. Probable that measurement or ascertainment of outcome did not differ between groups. Outcome assessors were unaware of intervention allocations.	Low risk of bias	Prospective registry was available. Outcome analysed as prespecified.	Some concerns	We judged the study as having some concerns in at least one domain for this result, but not to be at high risk of bias for any domain.
4v NCT00411749 2006‐JPN	Some concerns	Trial declared as randomised, but no additional information provided; sequence probably random, no information on allocation concealment. No information on baseline differences.	Low risk of bias	Blinded study (participants and personnel). Appropriate analysis: participants who receive at least one dose were included in analysis.	Low risk of bias	Data analysed for all 107 randomised participants.	Low risk of bias	Method of measuring outcome probably appropriate. Probable that measurement or ascertainment of outcome did not differ between groups. Outcome assessors were unaware of intervention allocations.	Low risk of bias	The prospective registry was available. Serious adverse events outcome was not prespecified, however, we do not consider the reporting of this outcome to be selective since serious adverse events should be reported in all clinical trials.	Some concerns	We judged the study as having some concerns in at least one domain for this result, but not to be at high risk of bias for any domain.
**Subgroup 3.21.2 Females 15‐25 years**												
4v EVRI 2016‐ZAF	Some concerns	Trial declared as randomised, but no additional information provided; sequence probably random, no information on allocation concealment. Imbalances in baseline characteristics appear to be compatible with chance.	Low risk of bias	Blinded study (participants and personnel or carers, or both). Appropriate analysis used: ITT.	Low risk of bias	Data available for all 402 randomised participants.	Low risk of bias	Method of measuring outcome probably appropriate. Probable that measurement or ascertainment of outcome did not differ between groups. Outcome assessors were unaware of intervention allocations.	Low risk of bias	The prospective registry was available. Serious adverse events outcome was not prespecified, however, we do not consider the reporting of this outcome to be selective since serious adverse events should be reported in all clinical trials.	Some concerns	We judged the study as having some concerns in at least one domain for this result, but not to be at high risk of bias for any domain.
4v FUTURE 2007‐INT	Low risk of bias	Computer‐generated random sequence; allocation concealed through interactive voice response system. Imbalances in baseline characteristics appear to be compatible with chance.	Low risk of bias	Blinded study (participants and personnel or carers, or both). Appropriate analysis used (all participants vaccinated with a follow‐up).	Low risk of bias	Data available for nearly all participants randomised (5345/5455).	Low risk of bias	Method of measuring outcome probably appropriate. Probable that measurement or ascertainment of outcome did not differ between groups. Outcome assessors were unaware of intervention allocations.	Low risk of bias	The prospective registry was available (NCT record). Outcome analysed as prespecified.	Low risk of bias	We judged the study to be at low risk of bias for all domains for this result.
4v FUTURE II 2007‐INT	Low risk of bias	Computer‐generated random sequence with concealed allocation using an interactive voice response system. Imbalances in baseline characteristics appear to be compatible with chance.	Low risk of bias	Blinded study (participants and personnel or carers, or both). Appropriate analysis (all participants with follow‐up data).	Low risk of bias	Data available for nearly all participants randomised (12050/12167).	Low risk of bias	Method of measuring outcome probably appropriate. Probable that measurement or ascertainment of outcome did not differ between groups. Outcome assessors were unaware of intervention allocations.	Low risk of bias	The prospective registry was available. Serious adverse events outcome was not prespecified, however, we do not consider the reporting of this outcome to be selective since serious adverse events should be reported in all clinical trials.	Low risk of bias	We judged the study to be at low risk of bias for all domains for this result.
4v Kang 2008‐KOR	Some concerns	Randomisation was performed by the study centers using the block method with decreasing block sizes; sequence random, no information on allocation concealment. Imbalances in baseline characteristics appear to be compatible with chance.	Low risk of bias	Blinded study (participants and personnel or carers, or both). Appropriate analysis used: ITT.	Low risk of bias	Data analysed for all 176 randomised participants.	Low risk of bias	Method of measuring outcome probably appropriate. Probable that measurement or ascertainment of outcome did not differ between groups. Outcome assessors were unaware of intervention allocations.	Low risk of bias	The prospective registry was available. Outcome analysed as prespecified.	Some concerns	We judged the study as having some concerns in at least one domain for this result, but not to be at high risk of bias for any domain.
4v Villa 2005‐INT	Some concerns	Randomisation schedules were computer generated by use of a blocking factor of eight; sequence random, no information on allocation concealment. Imbalances in baseline characteristics appear to be compatible with chance.	Some concerns	Blinded study (participants and personnel or carers, or both). Per‐protocol analysis was used (in the study it was labelled modified ITT) that included participants who were seronegative and DNA negative to the relevant HPV type at enrolment and who had had at least one vaccination. Per‐protocol analysis is not considered appropriate to assess effect of assignment to intervention.	Low risk of bias	Data available for nearly all participants randomised (546/552).	Low risk of bias	Method of measuring outcome probably appropriate. Probable that measurement or ascertainment of outcome did not differ between groups. Outcome assessors were unaware of intervention allocations.	Low risk of bias	The study was registered six years after commencement. Serious adverse events outcome was not prespecified, however, we do not consider the reporting of this outcome to be selective since serious adverse events should be reported in all clinical trials.	Some concerns	We judged the study as having some concerns in at least one domain for this result, but not to be at high risk of bias for any domain.
4v Yoshikawa 2013‐JPN	Some concerns	Randomisation schedule generated by permuted block method; sequence random, no information about allocation concealment. Imbalances in baseline characteristics appear to be compatible with chance.	Low risk of bias	Blinded study (participants and personnel or carers, or both). Appropriate analysis: all participants who received at least one study vaccination and had follow‐up data.	Some concerns	Data were not available for all participants or nearly all participants that were randomised: 948 analysed of 1021 randomised; 7% missing data balanced between groups.	Low risk of bias	Method of measuring outcome probably appropriate. Probable that measurement or ascertainment of outcome did not differ between groups. Outcome assessors were unaware of intervention allocations.	Low risk of bias	Registry was available, safety outcomes were not prespecified. We do not consider the reporting of serious adverse events to be selective since this outcome should be reported in all clinical trials.	Some concerns	We judged the study as having some concerns in at least one domain for this result, but not to be at high risk of bias for any domain.
**Subgroup 3.21.3 Females 25 years or older**												
4v FUTURE III 2009‐INT	Low risk of bias	Computer‐generated allocation schedule and an interactive voice response system; sequence random, allocation concealed. Groups similar for baseline characteristics.	Low risk of bias	Blinded study (participants and personnel carers). Appropriate analysis used (participants who received at least one dose and returned for follow‐up).	Low risk of bias	Data available for nearly all participants randomised (3778/3819).	Low risk of bias	Method of measuring outcome probably appropriate. Probable that measurement or ascertainment of outcome did not differ between groups. Outcome assessors were unaware of intervention allocations.	Low risk of bias	The prospective registry was available. Outcome analysed as prespecified.	Low risk of bias	We judged the study to be at low risk of bias for all domains for this result.
4v Wei 2019‐CHN	Some concerns	Block randomisation scheme; sequence probably random, no information about allocation concealment. Imbalances in baseline characteristics appear to be compatible with chance.	Low risk of bias	Blinded study (participants and personnel or carers, or both). Appropriate analysis: total vaccinated cohort.	Low risk of bias	Data available for nearly all participants randomised (2997/3006).	Low risk of bias	Method of measuring outcome probably appropriate. Probable that measurement or ascertainment of outcome did not differ between groups. Outcome assessors were unaware of intervention allocations.	Low risk of bias	The prospective protocol and registry were available. Outcome analysed as prespecified.	Some concerns	We judged the study as having some concerns in at least one domain for this result, but not to be at high risk of bias for any domain.
**Subgroup 3.21.4 Females and males**												
4v Chang 2020‐USA	Low risk of bias	Randomisation using an interactive voice response system; sequence random, allocation concealed. Imbalances in baseline characteristics appear to be compatible with chance.	Low risk of bias	Unblinded study (participants and personnel or carers, or both). No participant cross‐over; deviations did probably not arise because of the trial context. Appropriate analysis: total vaccinated cohort.	Low risk of bias	Data available for nearly all participants randomised (1692/1715).	Some concerns	Method of measuring outcome probably appropriate. Unblinded study (outcome assessor). This outcome requires clinical judgement and could be affected by knowledge of intervention assignment.	Low risk of bias	The prospective registry was available. Outcome analysed as prespecified.	Some concerns	We judged the study as having some concerns in at least one domain for this result, but not to be at high risk of bias for any domain.
4v Li 2012‐CHN	Some concerns	Trial declared as randomised, but no additional information provided; sequence probably random, no information on allocation concealment. Minor imbalances in baseline characteristics do not suggest problems with the randomisation process.	Low risk of bias	Blinded study (participants and personnel or carers, or both). Appropriate analysis: ITT.	Low risk of bias	Data analysed for all 600 randomised participants.	Low risk of bias	Method of measuring outcome probably appropriate. Probable that measurement or ascertainment of outcome did not differ between groups. Outcome assessors were unaware of intervention allocations.	Low risk of bias	Protocol and prospective registry were available. Outcome analysed as prespecified.	Some concerns	We judged the study as having some concerns in at least one domain for this result, but not to be at high risk of bias for any domain.
4v Reisinger 2007‐INT	Low risk of bias	Computer‐generated random sequence with interactive voice response system to allocate study participants and assign allocation numbers; sequence random, allocation concealed. Groups were similar for baseline characteristics.	Low risk of bias	Blinded study (particpants and personnel or carers, or both). Appropriate analysis: total vaccinated cohort.	Low risk of bias	Data available for nearly all participants randomised (1749/1781).	Low risk of bias	Method of measuring outcome probably appropriate. Probable that measurement or ascertainment of outcome did not differ between groups. Outcome assessors were unaware of intervention allocations.	Low risk of bias	Prospective registry or protocol was not available. Serious adverse events outcome was not prespecified, however, we do not consider the reporting of this outcome to be selective since serious adverse events should be reported in all clinical trials.	Low risk of bias	We judged the study to be at low risk of bias for all domains for this result.
4v Senders 2016‐USA	Some concerns	Trial declared to be randomised but no details on randomisation process were provided; sequence probably random, no information on allocation concealment.	Some concerns	Blinded study (participants and personnel or carers, or both). Per‐protocol analysis, not considered appropriate to assess effect of assignment to intervention.	Low risk of bias	Data available for nearly all participants randomised (2483/2499).	Low risk of bias	Method of measuring outcome probably appropriate. Probable that measurement or ascertainment of outcome did not differ between groups. Outcome assessors were unaware of intervention allocations.	Low risk of bias	The prospective registry was available. Serious adverse events not prespecified but adverse events were. Result was probably not selected based on multiple outcome measurements or analyses.	Some concerns	We judged the study as having some concerns in at least one domain for this result, but not to be at high risk of bias for any domain.
**Subgroup 3.21.5 Males 15‐25 years**												
4v Giuliano 2011‐INT	Low risk of bias	Central randomisation; sequence random, allocation concealed. Imbalances in baseline characteristics appear to be compatible with chance.	Low risk of bias	Blinded study (participants and personnel or carers, or both). Appropriate analysis (all vaccinated participants).	Low risk of bias	Data available for nearly all participants randomised (3895/4065).	Low risk of bias	Method of measuring outcome probably appropriate. Probable that measurement or ascertainment of outcome did not differ between groups. Outcome assessors were unaware of intervention allocations.	Low risk of bias	The prospective protocol and registry were available. Outcome analysed as prespecified.	Low risk of bias	We judged the study to be at low risk of bias for all domains for this result.
4v Mikamo 2019‐JPN	Low risk of bias	Randomisation via a central integrated web response system; sequence random, allocation probably concealed.	Low risk of bias	Blinded study (participants and personnel or carers, or both). Appropriate analysis: total vaccinated cohort.	Low risk of bias	Data available for nearly all participants randomised (1113/1124).	Low risk of bias	Method of measuring outcome probably appropriate. Probable that measurement or ascertainment of outcome did not differ between groups. Outcome assessors were unaware of intervention allocations.	Low risk of bias	Prospective protocol and registry were available. Outcome analysed as prespecified.	Low risk of bias	We judged the study to be at low risk of bias for all domains for this result.

**Table:** Risk of bias for analysis 3.22 Treatment for HPV‐related pre‐invasive disease

**Study**	**Bias**											
	**Randomisation process**		**Deviations from intended interventions**		**Missing outcome data**		**Measurement of the outcome**		**Selection of the reported results**		**Overall**	
	**Authors' judgement**	**Support for judgement**	**Authors' judgement**	**Support for judgement**	**Authors' judgement**	**Support for judgement**	**Authors' judgement**	**Support for judgement**	**Authors' judgement**	**Support for judgement**	**Authors' judgement**	**Support for judgement**
**Subgroup 3.22.1 Females 14 years or younger**												
**Subgroup 3.22.2 Females 15‐25 years**												
**Subgroup 3.22.3 Females 25 years or older**												
4v FUTURE III 2009‐INT	Low risk of bias	Computer‐generated allocation schedule and an interactive voice response system; sequence random, allocation concealed. Groups similar for baseline characteristics.	Low risk of bias	Blinded study (participants and personnel or carers, or both). Appropriate analysis used (participants who received at least one dose and returned for follow‐up).	Low risk of bias	Data available for nearly all participants randomised (3768/3819).	Low risk of bias	Method of measuring outcome probably appropriate. Probable that measurement or ascertainment of outcome did not differ between groups. Outcome assessors were unaware of intervention allocations.	Some concerns	The prospective registry was available. Outcome not prespecified. No information on whether the result was selected from multiple outcome measurements or analyses of the data.	Some concerns	We judged the study as having some concerns in at least one domain for this result, but not to be at high risk of bias for any domain.
**Subgroup 3.22.4 Females and males**												
**Subgroup 3.22.5 Males 15‐25 years**												
4v Giuliano 2011‐INT	Low risk of bias	Central randomisation; sequence random, allocation concealed. Imbalances in baseline characteristics appear to be compatible with chance.	Low risk of bias	Blinded study (participants and personnel or carers, or both). Appropriate analysis (full analysis set).	Low risk of bias	Data available for nearly all participants randomised (3880/4065).	Low risk of bias	Method of measuring outcome probably appropriate. Probable that measurement or ascertainment of outcome did not differ between groups. Outcome assessors were unaware of intervention allocations.	Some concerns	Prospective registry was available. Outcome not prespecified. No information on whether the result was selected from multiple outcome measurements or analyses of the data.	Some concerns	We judged the study as having some concerns in at least one domain for this result, but not to be at high risk of bias for any domain.

**Table:** Risk of bias for analysis 3.23 Anogenital warts irrespective of HPV type

**Study**	**Bias**											
	**Randomisation process**		**Deviations from intended interventions**		**Missing outcome data**		**Measurement of the outcome**		**Selection of the reported results**		**Overall**	
	**Authors' judgement**	**Support for judgement**	**Authors' judgement**	**Support for judgement**	**Authors' judgement**	**Support for judgement**	**Authors' judgement**	**Support for judgement**	**Authors' judgement**	**Support for judgement**	**Authors' judgement**	**Support for judgement**
**Subgroup 3.23.1 Females 14 years or younger**												
**Subgroup 3.23.2 Females 15‐25 years**												
4v FUTURE I/II 2010‐INT	Low risk of bias	Analysis pooling two RCTs. Both studies used a computer‐generated random sequence with allocation concealed through interactive voice response system.	Low risk of bias	Both studies were blinded (participants and personnel or carers, or both). Appropriate analysis used: at least one vaccination with at least 1 follow‐up.	Low risk of bias	Data analysed for nearly all randomized participants (17,391/17,662).	Low risk of bias	Method of measuring outcome probably appropriate. Probable that measurement or ascertainment of outcome did not differ between groups. Outcome assessors were unaware of intervention allocations.	Some concerns	Prospective registries for the two studies were available (NCT record). One of the studies had not planned to analyse this outcome.	Some concerns	We judged the study as having some concerns in at least one domain for this result, but not to be at high risk of bias for any domain.
**Subgroup 3.23.3 Females 25 years or older**												
**Subgroup 3.23.4 Females and males**												
**Subgroup 3.23.5 Males 15‐25 years**												
4v Giuliano 2011‐INT	Low risk of bias	Central randomisation; sequence random, allocation concealed. Imbalances in baseline characteristics appear to be compatible with chance.	Low risk of bias	Blinded study (participants and personnel or carers, or both). Appropriate analysis (full analysis set).	Low risk of bias	Data available for nearly all participants randomised (3880/4065).	Low risk of bias	Method of measuring outcome probably appropriate. Probable that measurement or ascertainment of outcome did not differ between groups. Outcome assessors were unaware of intervention allocations.	Low risk of bias	The prospective protocol and registry were available. Outcome analysed as prespecified.	Low risk of bias	We judged the study to be at low risk of bias for all domains for this result.

**Table:** Risk of bias for analysis 3.24 Anogenital warts associated with HPV 6, 11, 16 and/or 18

**Study**	**Bias**											
	**Randomisation process**		**Deviations from intended interventions**		**Missing outcome data**		**Measurement of the outcome**		**Selection of the reported results**		**Overall**	
	**Authors' judgement**	**Support for judgement**	**Authors' judgement**	**Support for judgement**	**Authors' judgement**	**Support for judgement**	**Authors' judgement**	**Support for judgement**	**Authors' judgement**	**Support for judgement**	**Authors' judgement**	**Support for judgement**
**Subgroup 3.24.1 Females 14 years or younger**												
**Subgroup 3.24.2 Females 15‐25 years**												
4v FUTURE I/II 2010‐INT	Low risk of bias	Analysis pooling two RCTs. Both studies used a computer‐generated random sequence with allocation concealed through interactive voice response system.	Low risk of bias	Both studies were blinded (participants and personnel or carers, or both). Appropriate analysis used: at least one vaccination with at least one follow‐up.	Low risk of bias	Data analysed for nearly all randomized participants (17,391/17,662).	Low risk of bias	Method of measuring outcome probably appropriate. Probable that measurement or ascertainment of outcome did not differ between groups. Outcome assessors were unaware of intervention allocations.	Some concerns	Prospective registries for the two studies were available (NCT record). One of the studies had not planned to analyse this outcome.	Some concerns	We judged the study as having some concerns in at least one domain for this result, but not to be at high risk of bias for any domain.
4v Villa 2005‐INT	Some concerns	Randomisation schedules were computer generated by use of a blocking factor of eight; sequence random, no information on allocation concealment. Imbalances in baseline characteristics appear to be compatible with chance.	Some concerns	Blinded study (participants and personnel or carers, or both). Per‐protocol analysis was used (in the study it was labelled modified ITT) that included participants who were seronegative and DNA negative to the relevant HPV type at enrolment and who had had at least one vaccination. Per‐protocol analysis is not considered appropriate to assess effect of assignment to intervention.	Low risk of bias	Data available for nearly all participants randomised (526/552).	Low risk of bias	Method of measuring outcome probably appropriate. Probable that measurement or ascertainment of outcome did not differ between groups. Outcome assessors were unaware of intervention allocations.	Some concerns	The study was registered six years after commencement. No information on whether the result was selected from multiple outcome measurements or analyses of the data.	Some concerns	We judged the study as having some concerns in at least one domain for this result, but not to be at high risk of bias for any domain.
**Subgroup 3.24.3 Females 25 years or older**												
4v FUTURE III 2009‐INT	Low risk of bias	Computer‐generated allocation schedule and an interactive voice response system; sequence random, allocation concealed. Groups similar for baseline characteristics.	Low risk of bias	Blinded study (participants and personnel or carers, or both). Appropriate analysis used (participants who received at least one dose and returned for follow‐up).	Some concerns	Data were not available for all participants or nearly all participants that were randomised: 3382 analysed of 3819 randomised; 11% missing data balanced between groups.	Low risk of bias	Method of measuring outcome probably appropriate. Probable that measurement or ascertainment of outcome did not differ between groups. Outcome assessors were unaware of intervention allocations.	Low risk of bias	The prospective registry was available. Outcome analysed as prespecified.	Some concerns	We judged the study as having some concerns in at least one domain for this result, but not to be at high risk of bias for any domain.
**Subgroup 3.24.4 Females and males**												
**Subgroup 3.24.5 Males 15‐25 years**												
4v Giuliano 2011‐INT	Low risk of bias	Central randomisation; sequence random, allocation concealed. Imbalances in baseline characteristics appear to be compatible with chance.	Low risk of bias	Blinded study (participants and personnel or carers, or both). Appropriate analysis (full analysis set).	Low risk of bias	Data available for nearly all participants randomised (3880/4065).	Low risk of bias	Method of measuring outcome probably appropriate. Probable that measurement or ascertainment of outcome did not differ between groups. Outcome assessors were unaware of intervention allocations.	Low risk of bias	The prospective protocol and registry were available. Outcome analysed as prespecified.	Low risk of bias	We judged the study to be at low risk of bias for all domains for this result.

## Figures and Tables

**10:**
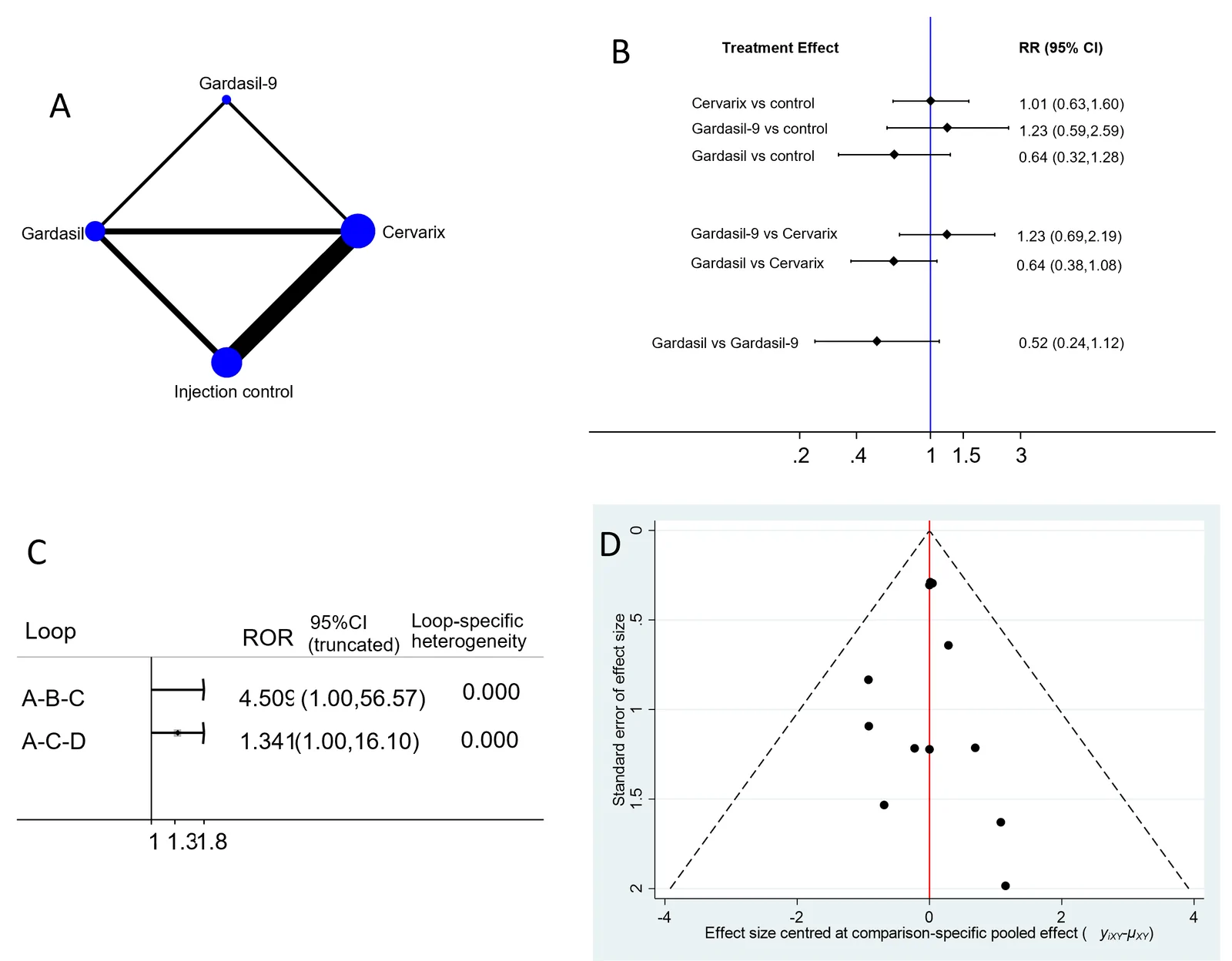
NMA of SAEs in females up to 15 years old: A) network map, B) interval plot, C) consistency plot and D) funnel plot

**11:**
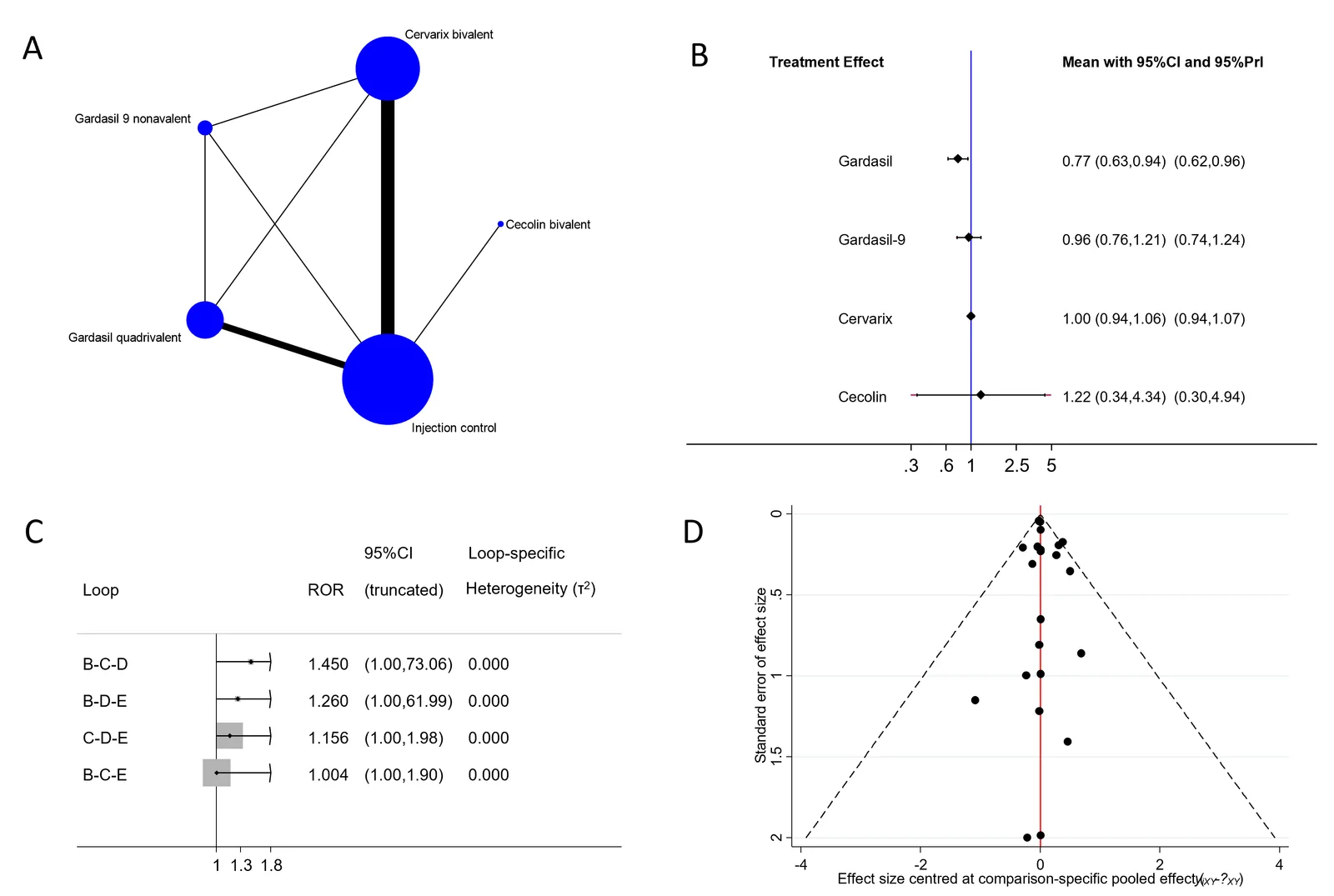
NMA of SAEs in females 15 to 25 years old: A) network map, B) interval plot, C) consistency plot and D) funnel plot

**12:**
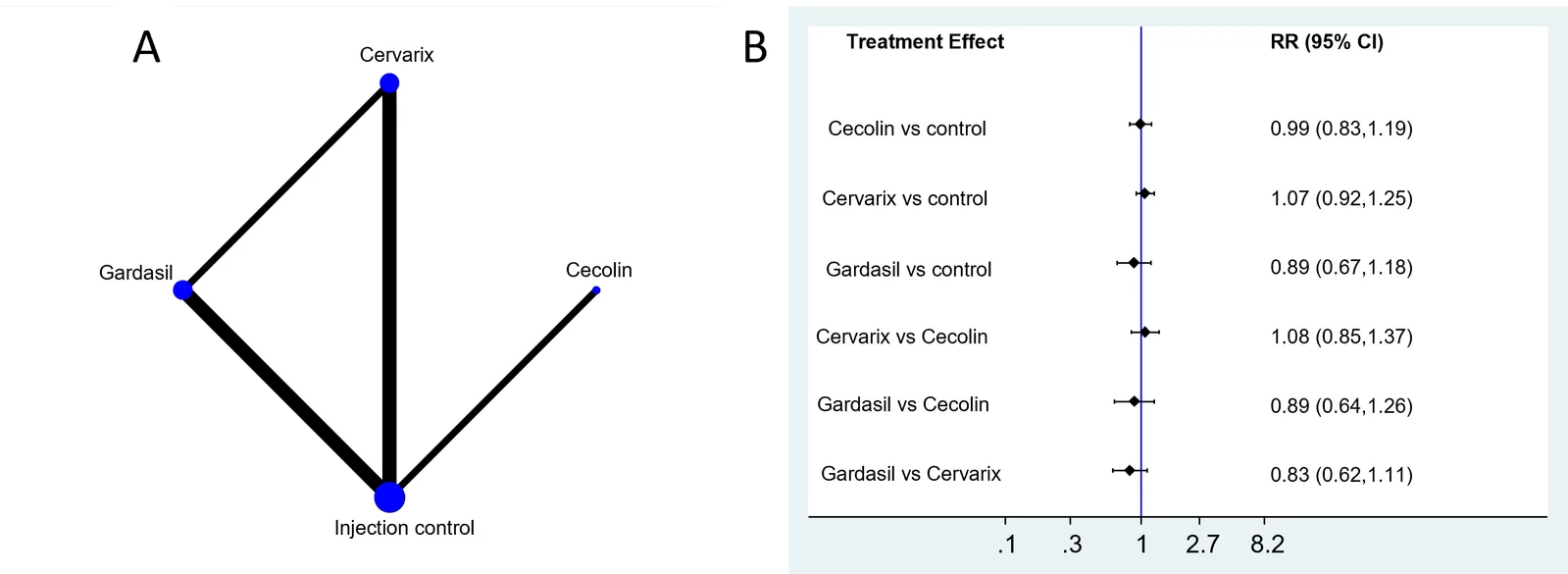
NMA of SAEs in females 25 years and older: A) network map, B) interval plot

**13:**
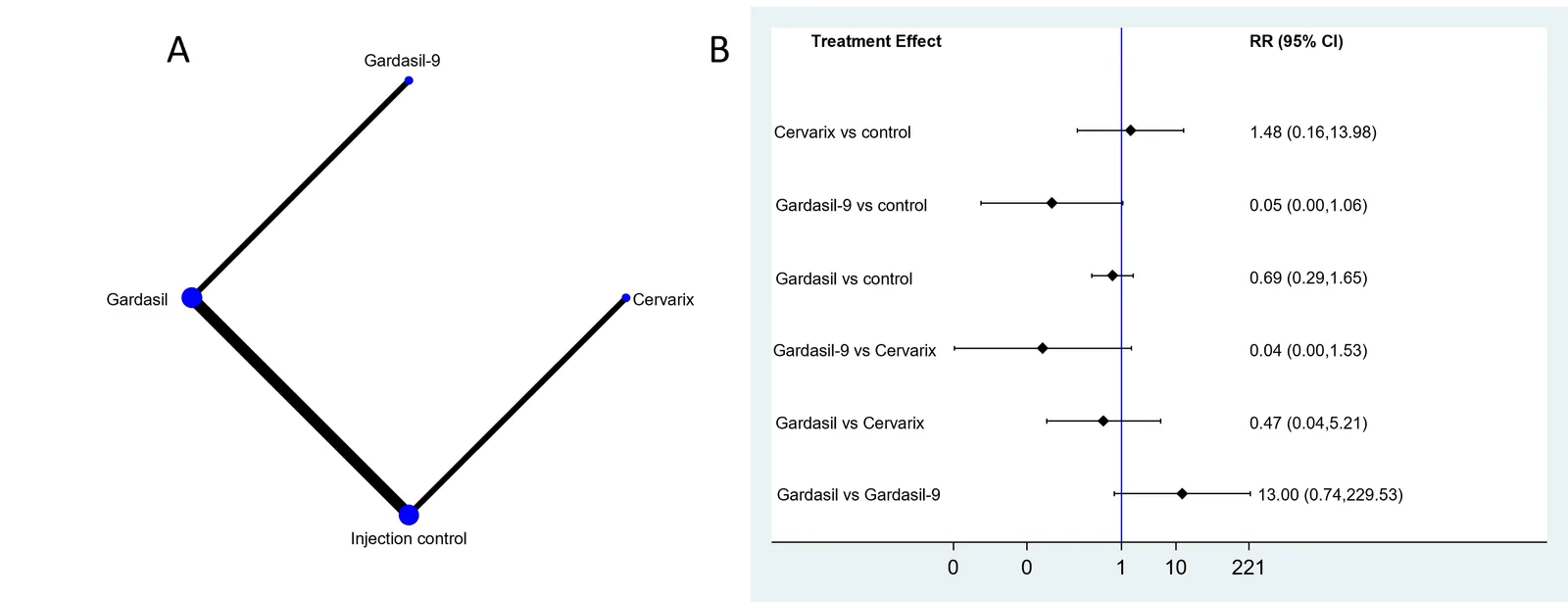
NMA of SAEs in males 15 to 25 years old: A) network map, B) interval plot

## Appendices

### Appendix 1. Analysis of social media reporting of adverse events following HPV vaccination

We sought to identify adverse events that were potentially related to HPV vaccination, which were commonly mentioned on social media.

Firstly, we screened all the reviews on WebMD of HPV vaccines to identify mentions of adverse events. We coded each mention of a personal experience where possible to MedDRA preferred terms.

There were 276 adverse events mentioned and annotated. The most common adverse events were injection site pain, headaches and missed periods.

**Table:**

**WebMD adverse event mentions** **(rank order of frequency)**	**Adverse event**
1	injection site pain
2	headache
3	missing periods
4	dizziness
5	fatigue
6	nausea
7	myalgia
8	fever
9	malaise
10	pain
11	syncope
12	abdominal pain
13	influenza‐like illness
14	alopecia
15	cramping
16	dyspnoea
17	rash
18	tremor
19	vomiting
20	anxiety
21	arthralgia
22	chest pain
23	cough
24	diarrhoea
25	infertility
26	syncope (recurrent)
27	tingling
28	aluminium toxicity
29	back pain
30	death
31	dehydration
32	hives
33	hypoaesthesia
34	insomnia
35	migraine
36	shoulder pain
37	swollen glands
38	seizure
39	auto‐immune disease

We also investigated an analysis of "Tweets" on X (formerly known as Twitter). Recent news events with the release of the results of a clinical trial and activity on Twitter related to the COVID‐19 vaccines meant that recent posts suffered from a lot of noise. Many posts mentioning adverse events were also doing so to promote an anti‐HPV vaccination stance rather than personal experience, with accounts dedicated to promoting HPV side effect information (@HPVSideEffects) and reference to the vaccine as ‘Human Paralysis inducing Vaccine’. Refusal of the vaccine was also stated to be related to parents not wanting to promote sexual activity in their children.

We were able to uncover 46 recent adverse event experience mentions.

**Table:**

**Twitter (now called X) adverse event mentions** **(rank order of frequency)**	**Adverse event**
1	death
2	auto‐immune disease
3	chronic fatigue syndrome
4	inability to walk
5	infertility
6	myalgic encephalomyelitis
7	paralysed
8	seizures/epilepsy
9	tremors
10	aluminium toxicity
11	anxiety
12	chronic kidney disease
13	encephalitis
14	epilepsy
15	Epstein Barr
16	functional neurologic disorder
17	Hashimoto's disease
18	heart problem
19	missing periods
20	myocarditis
21	nervous breakdown
22	pain
23	postural orthostatic tachycardia syndrome
24	stuttering
25	syncope
26	systemic lupus erythematosus
27	weakness
28	amyotrophic lateral sclerosis

### Appendix 2. Search strategies

**MEDLINE Ovid** (2000 to 18 September 2024)

1. exp Papillomavirus Vaccines/ 2. gardasil*.mp. 3. cervarix*.mp. 4. ((human papilloma virus* or human papiloma virus*) adj (vaccin* or immuni*)).tw. 5. ((human papillomavirus* or human papilomavirus*) adj (vaccin* or immuni*)).tw. 6. (HPV* adj3 (vaccin* or immuni*)).tw. 7. 1 or 2 or 3 or 4 or 5 or 6 8. randomized controlled trial.pt. 9. controlled clinical trial.pt. 10. randomized.ab. 11. placebo.ab. 12. drug therapy.fs. 13. randomly.ab. 14. trial.ti. 15. groups.ab. 16. 8 or 9 or 10 or 11 or 12 or 13 or 14 or 15 17. (animals not (humans and animals)).sh. 18. 16 not 17 19. 7 and 18

**Embase Ovid** (2000 to 18 September 2024)

1. exp Wart virus vaccine/  2. gardasil*.mp. 3. cervarix*.mp.  4. ((human papilloma virus* or human papiloma virus*) adj (vaccin* or immuni*)).tw.  5. ((human papillomavirus* or human papilomavirus*) adj (vaccin* or immuni*)).tw.  6. (HPV* adj3 (vaccin* or immuni*)).tw.  7. 1 or 2 or 3 or 4 or 5 or 6  8. crossover procedure/  9. double‐blind procedure/  10. randomized controlled trial/  11. single‐blind procedure/  12. random*.mp.  13. factorial*.mp.  14. (crossover* or cross over* or cross‐over*).mp.  15. placebo*.mp.  16. (double* adj blind*).mp.  17. (singl* adj blind*).mp.  18. assign*.mp.  19. allocat*.mp.  20. volunteer*.mp.  21. 8 or 9 or 10 or 11 or 12 or 13 or 14 or 15 or 16 or 17 or 18 or 19 or 20  22. 7 and 21

**Cochrane Central Register of Controlled Trials (CENTRAL) in the Cochrane Library** (2024, Issue 9)

#1 MeSH descriptor: [Papillomavirus Vaccines] explode all trees  #2 gardasil*  #3 cervarix*  #4 ((human papilloma virus* or human papiloma virus*) near (vaccin* or immuni*))  #5 ((human papillomavirus* or human papilomavirus*) near (vaccin* or immuni*))  #6 (HPV* near/3 (vaccin* or immuni*))  #7 #1 or #2 or #3 or #4 or #5 or #6

### Appendix 3. NMA results

#### 1. Females < 15 years

NMA was carried out for serious adverse events in this population. Other critical outcomes were not reported in this population.

##### 1.1 Serious adverse events

###### 1.1.1 Matrix of results (league table)

**Table:**

**Cervarix**	**Gardasil‐9**	**Gardasil**	**Control**
**Cervarix**	1.23 (0.69 to 2.19)	0.64 (0.38 to 1.08)	0.99 (0.63 to 1.58)
0.82 (0.46 to 1.46)	**Gardasil‐9**	0.52 (0.24 to 1.12)	0.81 (0.39 to 1.70)
1.56 (0.92 to 2.64)	1.92 (0.90 to 4.11)	**Gardasil**	1.55 (0.78 to 3.08)
1.01 (0.63 to 1.60)	1.23 (0.59 to 2.59)	0.64 (0.32 to 1.28)	**Control**

Values in table are risk ratios (RRs) and 95% confidence intervals (CIs).

###### 1.1.2 Probability of being the best vaccine (rank)

**Table:**

**Intervention**	**SUCRA**	**Probability of being best (%)**	**Mean rank**
Gardasil	93.4	85.8%	1.2
Control	44.5	9.0%	2.7
Cervarix	43.3	1.8%	2.7
Gardasil‐9	18.9	3.4%	3.4

See Figure for network map, interval plot, consistency plot and funnel plot. Publication bias, heterogeneity and incoherence were not detected.

#### 2. Females 15 to 25 years

NMA was carried out for CIN3+, CIN2+, vaccine‐matched HPV‐type high‐grade VIN or VaIN, anogenital warts and serious adverse events in this population. Two or fewer trials reported on the other critical outcomes in this population. Consequently, NMA was not carried out for those outcomes.

##### 2.1 CIN3+ irrespective of HPV type

###### 2.1.1 Matrix of results (league table)

**Table:**

**Cervarix**	**Gardasil**	**Control**
**Cervarix**	1.06 (0.37 to 3.07)	1.31 (0.75 to 2.29)
0.94 (0.33 to 2.71)	**Gardasil**	1.23 (0.50 to 3.02
0.76 (0.44 to 1.34	0.81 (0.33 to 2.00)	**Control**

Values in table are RR (95% CI).

###### 2.1.2 Probability of being the best vaccine (rank)

**Table:**

**Intervention**	**SUCRA**	**Probability of being best (%)**	**Mean rank**
Cervarix	68.1	49.7%	1.6
Gardasil	57.3	44.5%	1.9
Control	24.6	5.8%	2.5

See Figure for network map, interval plot, consistency plot and funnel plot. Publication bias, heterogeneity and incoherence were not detected.

##### 2.1 CIN3+ vaccine‐matched HPV type

###### 2.1.1 Matrix of results (league table)

**Table:**

**Cervarix**	**Gardasil‐9**	**Gardasil**	**Control**
**Cervarix**	0.06 (0.00 to 1.03)	0.98 (0.65 to 1.49)	1.83 (1.31 to 2.57)
17.28 (0.97 to 308.25)	**Gardasil‐9**	17.02 (0.98 to 294.76)	31.65 (1.81 to 553.56)
1.02 (0.67 to 1.53)	0.06 (0.00 to 1.02)	**Gardasil**	1.86 (1.47 to 2.35)
0.55 (0.39 to 0.77)	0.03 (0.00 to 0.55)	0.54 (0.43 to 0.68)	**Control**

Values in table are RR (95% CI).

###### 2.1.2 Probability of being the best vaccine (rank)

**Table:**

**Intervention**	**SUCRA**	**Probability of being best (%)**	**Mean rank**
Gardasil‐9	97.9	97%	1.1
Gardasil	52	1.5%	2.4
Cervarix	49.8	1.5%	2.5
Control	0.3	0%	4

See Figure for network map, interval plot, consistency plot and funnel plot. Publication bias, heterogeneity and incoherence were not detected.

##### 2.1 CIN2+ irrespective of HPV type

###### 2.1.1 Matrix of results (league table)

**Table:**

**Cervarix**	**Gardasil**	**Gardasil‐9**	**Control**
**Cervarix**	1.32 (0.55 to 3.18)	1.34 (0.42 to 4.26)	1.63 (1.05 to 2.53)
0.75 (0.31 to 1.81)	**Gardasil**	1.01 (0.47 to 2.16)	1.23 (0.58 to 2.62)
0.75 (0.23 to 2.38)	0.99 (0.46 to 2.12)	**Gardasil‐9**	1.22 (0.42 to 3.55)
0.61 (0.39 to 0.95)	0.81 (0.38 to 1.73)	0.82 (0.28 to 2.39)	**Control**

Values in table are RR (95% CI).

###### 2.1.2 Probability of being the best vaccine (rank)

**Table:**

**Intervention**	**SUCRA**	**Probability of being best (%)**	**Mean rank**
Cervarix	80.5	61.6%	1.6
Gardasil	49.5	13.9%	2.5
Gardasil‐9	47.4	24.3%	2.6
Control	22.6	0.3%	3.3

See Figure for network map, interval plot, consistency plot and funnel plot. Publication bias, heterogeneity and incoherence were not detected.

##### 2.1 CIN2+ vaccine‐matched HPV type

###### 2.1.1 Matrix of results (league table)

**Table:**

**Cervarix**	**Gardasil‐9**	**Gardasil**	**Control**
**Cervarix**	1.46 (0.51 to 4.21)	1.47 (0.56 to 3.90)	2.96 (1.23 to 7.10)
0.68 (0.24 to 1.97)	**Gardasil‐9**	1.01 (0.66 to 1.53)	2.03 (1.12 to 3.67)
0.68 (0.26 to 1.79)	0.99 (0.65 to 1.50)	**Gardasil**	2.01 (1.32 to 3.06)
0.34 (0.14 to 0.81)	0.49 (0.27 to 0.89)	0.50 (0.33 to 0.76)	**Control**

Values in table are RR (95% CI).

###### 2.1.2 Probability of being the best vaccine (rank)

**Table:**

**Intervention**	**SUCRA**	**Probability of being best (%)**	**Mean rank**
Cervarix	84.8	72.6%	1.5
Gardasil‐9	57.6	17.0%	2.3
Gardasil	57.0	10.4%	2.3
Control	0.7	0.0%	4

See Figure for network map, interval plot, consistency plot and funnel plot. Publication bias, heterogeneity and incoherence were not detected.

##### 2.2 Vaccine‐matched HPV‐type high‐grade VIN or VaIN

###### 2.2.1 Matrix of results (league table)

**Table:**

**Cervarix**	**Gardasil‐9**	**Gardasil**	**Control**
**Cervarix**	0.58 (0.09 to 3.94)	0.74 (0.13 to 4.17)	3.52 (0.73 to 17.00)
1.72 (0.25 to 11.71)	**Gardasil‐9**	1.27 (0.57 to 2.83)	6.07 (1.97 to 18.69)
1.36 (0.24 to 7.66)	0.79 (0.35 to 1.75)	**Gardasil**	4.77 (2.24 to 10.15)
0.28 (0.06 to 1.37)	0.16 (0.05 to 0.51)	0.21 (0.10 to 0.45)	**Control**

Values in table are RR (95% CI).

###### 2.2.2 Probability of being the best vaccine (rank)

**Table:**

**Intervention**	**SUCRA**	**Probability of being best (%)**	**Mean rank**
Gardasil‐9	80.9	56.2%	1.6
Gardasil	64.0	18%	2.1
Cervarix	53.2	25.8%	2.4
Control	2.0	0.0%	3.9

See Figure for network map, interval plot, consistency plot and funnel plot. Publication bias, heterogeneity and incoherence were not detected.

##### 2.3 Anogenital warts

###### 2.3.1 Matrix of results (league table)

**Table:**

**Gardasil‐9**	**Gardasil**	**Control**
**Gardasil‐9**	1.12 (0.82 to 1.53)	2.92 (1.98 to 4.31)
0.90 (0.65 to 1.23)	**Gardasil**	2.62 (2.12 to 3.23)
0.34 (0.23 to 0.51)	0.38 (0.31 to 0.47)	**Control**

###### 2.3.2 Probability of being the best vaccine (rank)

**Table:**

**Intervention**	**SUCRA**	**Probability of being best (%)**	**Mean rank**
Gardasil‐9	87.8	75.6%	1.2
Gardasil	62.2	24.4%	1.8
Control	0.0	0.0%	3.0

See Figure for network map, interval plot, consistency plot and funnel plot. Publication bias, heterogeneity and incoherence were not detected.

##### 2.4 Serious adverse events

###### 2.4.1 Matrix of results (league table)

**Table:**

**Cecolin**	**Cervarix**	**Gardasil‐9**	**Gardasil**	**Control**
**Cecolin**	0.82 (0.23 to 2.94)	0.79 (0.22 to 2.87)	0.63 (0.18 to 2.30)	0.82 (0.23 to 2.93)
1.21 (0.34 to 4.34)	**Cervarix**	0.96 (0.75 to 1.21)	0.77 (0.63 to 0.95)	1.00 (0.94 to 1.06)
1.27 (0.35 to 4.63)	1.05 (0.82 to 1.33)	**Gardasil‐9**	0.81 (0.68 to 0.96)	1.05 (0.83 to 1.32)
1.58 (0.44 to 5.71)	1.30 (1.06 to 1.59)	1.24 (1.04 to 1.48)	**Gardasil**	1.30 (1.06 to 1.58)
1.22 (0.34 to 4.34)	1.00 (0.94 to 1.06)	0.96 (0.76 to 1.21)	0.77 (0.63 to 0.94)	**Control**

###### 2.4.2 Probability of being the best vaccine (rank)

**Table:**

**Intervention**	**SUCRA**	**Probability of being best (%)**	**Mean rank**
Gardasil	93.4	74.6%	1.3
Gardasil‐9	48.6	0.7%	3.1
Control	37.4	0.3%	3.5
Cervarix	36.8	0.2%	3.5
Cecolin	33.8	24.2%	3.6

See Figure for network map, interval plot, consistency plot and funnel plot. Publication bias, heterogeneity and incoherence were not detected.

#### 3. Females > 25 years

NMA was carried out for serious adverse events in this population. Two or fewer trials reported on the other critical outcomes in this population. Consequently, NMA was not carried out for those outcomes.

##### 3.1 CIN2+ vaccine‐matched HPV type

###### 3.1.1 Matrix of results (league table)

**Table:**

**Cervarix**	**Gardasil**	**Control**
**Cervarix**	1.04 (0.57 to 1.89)	1.41 (0.90 to 2.21)
0.96 (0.53 to 1.74)	**Gardasil**	1.35 (0.91 to 2.00)
0.71 (0.45 to 1.11)	0.74 (0.50 to 1.09)	**Control**

Values in table are RR (95% CI)

###### 3.1.2 Probability of being the best vaccine (rank)

**Table:**

**Intervention**	**SUCRA**	**Probability of being best (%)**	**Mean rank**
Cervarix	73.8	54.2%	1.5
Gardasil	69.5	45.4%	1.6
Control	6.6	1.6%	2.9

See Figure for network map, interval plot, consistency plot and funnel plot. Publication bias, heterogeneity and incoherence were not detected.

##### 3.2 Serious adverse events

###### 3.2.1 Matrix of results (league table)

**Table:**

**Cecolin**	**Cervarix**	**Gardasil**	**Control**
**Cecolin**	1.08 (0.85 to 1.37)	0.89 (0.64 to 1.26)	1.01 (0.84 to 1.21)
0.93 (0.73 to 1.18)	**Cervarix**	0.83 (0.62 to 1.11)	0.93 (0.80 to 1.09)
1.12 (0.80 to 1.57)	1.20 (0.90 to 1.61)	**Gardasil**	1.12 (0.85 to 1.50)
0.99 (0.83 to 1.19)	1.07 (0.92 to 1.25)	0.89 (0.67 to 1.18)	**Control**

###### 3.2.2 Probability of being the best vaccine (rank)

**Table:**

**Intervention**	**SUCRA**	**Probability of being best (%)**	**Mean rank**
Gardasil	80.8	68.4%	1.6
Cecolin	51.0	20.2%	2.5
Control	49.5	8.9%	2.5
Cervarix	18.7	2.4%	3.4

See Figure for network map, interval plot, consistency plot and funnel plot. Publication bias, heterogeneity and incoherence were not detected.

#### 4. Males 15 to 25 years

NMA was carried out for serious adverse events in this population. Other critical outcomes were reported by only one trial in this population.

##### 4.1 Serious adverse events

###### 4.1.1 Matrix of results (league table)

**Table:**

**Cervarix**	**Gardasil‐9**	**Gardasil**	**Control**
**Cervarix**	0.04 (0.00 to 1.53)	0.47 (0.04 to 5.21)	0.68 (0.07 to 6.42)
27.86 (0.66 to 1185.14)	**Gardasil‐9**	13.00 (0.74 to 229.53)	18.89 (0.94 to 379.84)
2.14 (0.19 to 23.93)	0.08 (0.00 to 1.36)	**Gardasil**	1.45 (0.61 to 3.48)
1.48 (0.16 to 13.98)	0.05 (0.00 to 1.06)	0.69 (0.29 to 1.65)	**Control**

###### 4.1.2 Probability of being the best vaccine (rank)

**Table:**

**Intervention**	**SUCRA**	**Probability of being best (%)**	**Mean rank**
Gardasil‐9	96.3	93.4%	1.1
Gardasil	52.3	2.6%	2.4
Control	28.8	0.7%	3.1
Cervarix	22.6	3.3%	3.3

See Figure for network map, interval plot, consistency plot and funnel plot. Publication bias, heterogeneity and incoherence were not detected.

## Data and analyses

**Comparison 1:** HPV vaccine versus control

Outcome or subgroup title	No. of studies	No. of participants	Statistical method	Effect size
1.1 Invasive cervical cancer	2	17622	Risk Ratio (M‐H, Random, 95% CI)	Not estimable
1.1.1 Females 14 years or younger	0	0	Risk Ratio (M‐H, Random, 95% CI)	Not estimable
1.1.2 Females 15‐25 years	2	17622	Risk Ratio (M‐H, Random, 95% CI)	Not estimable
1.1.3 Females 25 years or older	0	0	Risk Ratio (M‐H, Random, 95% CI)	Not estimable
1.2 CIN3+ irrespective of HPV type	5	43901	Risk Ratio (M‐H, Random, 95% CI)	0.78 [0.55, 1.12]
1.2.1 Females 14 years or younger	0	0	Risk Ratio (M‐H, Random, 95% CI)	Not estimable
1.2.2 Females 15‐25 years	5	43901	Risk Ratio (M‐H, Random, 95% CI)	0.78 [0.55, 1.12]
1.2.3 Females 25 years or older	0	0	Risk Ratio (M‐H, Random, 95% CI)	Not estimable
1.3 CIN3+ associated with vaccine‐matched HPV types	3	35655	Risk Ratio (M‐H, Random, 95% CI)	0.54 [0.44, 0.65]
1.3.1 Females 14 years or younger	0	0	Risk Ratio (M‐H, Random, 95% CI)	Not estimable
1.3.2 Females 15 to 25 years	3	35655	Risk Ratio (M‐H, Random, 95% CI)	0.54 [0.44, 0.65]
1.3.3 Females 25 years or older	0	0	Risk Ratio (M‐H, Random, 95% CI)	Not estimable
1.4 CIN2+ irrespective of HPV type	7	55911	Risk Ratio (M‐H, Random, 95% CI)	0.79 [0.65, 0.96]
1.4.1 Females 14 years or younger	0	0	Risk Ratio (M‐H, Random, 95% CI)	Not estimable
1.4.2 Females 15‐25 years	5	46624	Risk Ratio (M‐H, Random, 95% CI)	0.70 [0.56, 0.88]
1.4.3 Females 25 years or older	2	9287	Risk Ratio (M‐H, Random, 95% CI)	1.04 [0.83, 1.30]
1.5 CIN2+ associated with vaccine‐matched HPV types	9	59717	Risk Ratio (M‐H, Random, 95% CI)	0.50 [0.39, 0.65]
1.5.1 Females 14 years or younger	0	0	Risk Ratio (M‐H, Random, 95% CI)	Not estimable
1.5.2 Females 15 to 25 years	6	47462	Risk Ratio (M‐H, Random, 95% CI)	0.40 [0.30, 0.54]
1.5.3 Females 25 years or older	3	12255	Risk Ratio (M‐H, Random, 95% CI)	0.73 [0.54, 0.98]
1.6 Adenocarcinoma in situ irrespective of HPV type	4		Risk Ratio (M‐H, Random, 95% CI)	Subtotals only
1.6.1 Females 14 years or younger	0	0	Risk Ratio (M‐H, Random, 95% CI)	Not estimable
1.6.2 Females 15‐25 years	3	35024	Risk Ratio (M‐H, Random, 95% CI)	0.36 [0.16, 0.78]
1.6.3 Females 25 years or older	0	0	Risk Ratio (M‐H, Random, 95% CI)	Not estimable
1.6.4 Females and males	0	0	Risk Ratio (M‐H, Random, 95% CI)	Not estimable
1.6.5 Males 15‐25 years	1	551	Risk Ratio (M‐H, Random, 95% CI)	Not estimable
1.7 Adenocarcinoma in situ associated with vaccine‐matched HPV types	2	35085	Risk Ratio (M‐H, Random, 95% CI)	0.36 [0.17, 0.78]
1.8 CIN3 irrespective of HPV type	2	17622	Risk Ratio (M‐H, Random, 95% CI)	0.91 [0.66, 1.27]
1.8.1 Females 14 years or younger	0	0	Risk Ratio (M‐H, Random, 95% CI)	Not estimable
1.8.2 Females 15‐25 years	2	17622	Risk Ratio (M‐H, Random, 95% CI)	0.91 [0.66, 1.27]
1.8.3 Females 25 years or older	0	0	Risk Ratio (M‐H, Random, 95% CI)	Not estimable
1.9 CIN3 associated with vaccine‐matched HPV types	2	18796	Risk Ratio (M‐H, Random, 95% CI)	0.55 [0.43, 0.70]
1.10 CIN2 irrespective of HPV type	1	17160	Risk Ratio (M‐H, Random, 95% CI)	0.81 [0.70, 0.94]
1.10.1 Females 14 years or younger	0	0	Risk Ratio (M‐H, Random, 95% CI)	Not estimable
1.10.2 Females 15‐25 years	1	17160	Risk Ratio (M‐H, Random, 95% CI)	0.81 [0.70, 0.94]
1.10.3 Females 25 years or older	0	0	Risk Ratio (M‐H, Random, 95% CI)	Not estimable
1.11 CIN2 associated with vaccine‐matched HPV types	2	18796	Risk Ratio (M‐H, Random, 95% CI)	0.44 [0.34, 0.57]
1.12 Vulval or vaginal cancer irrespective of HPV type	1	5455	Risk Ratio (M‐H, Random, 95% CI)	3.01 [0.12, 73.85]
1.13 Vulval or vaginal cancer associated with vaccine‐matched HPV types	1	5455	Risk Ratio (M‐H, Random, 95% CI)	Not estimable
1.14 High‐grade VIN or VaIN irrespective of HPV type	2	33092	Risk Ratio (M‐H, Random, 95% CI)	0.48 [0.33, 0.70]
1.14.1 Females 14 years or younger	0	0	Risk Ratio (M‐H, Random, 95% CI)	Not estimable
1.14.2 Females 15‐25 years	2	33092	Risk Ratio (M‐H, Random, 95% CI)	0.48 [0.33, 0.70]
1.14.3 Females 25 years or older	0	0	Risk Ratio (M‐H, Random, 95% CI)	Not estimable
1.15 High‐grade VIN or VaIN associated with vaccine‐matched HPV types	3	36873	Risk Ratio (M‐H, Random, 95% CI)	0.35 [0.10, 1.24]
1.15.1 Females 14 years or younger	0	0	Risk Ratio (M‐H, Random, 95% CI)	Not estimable
1.15.2 Females 15‐25 years	2	33491	Risk Ratio (M‐H, Random, 95% CI)	0.22 [0.11, 0.42]
1.15.3 Females 25 years or older	1	3382	Risk Ratio (M‐H, Random, 95% CI)	4.98 [0.24, 103.58]
1.16 Serious adverse events	39	97272	Risk Ratio (M‐H, Random, 95% CI)	0.99 [0.94, 1.04]
1.16.1 Females 14 years or younger	8	5043	Risk Ratio (M‐H, Random, 95% CI)	1.01 [0.63, 1.61]
1.16.2 Females 15‐25 years	19	59621	Risk Ratio (M‐H, Random, 95% CI)	0.96 [0.89, 1.03]
1.16.3 Females 25 years or older	5	20806	Risk Ratio (M‐H, Random, 95% CI)	1.02 [0.91, 1.14]
1.16.4 Females and males	4	6524	Risk Ratio (M‐H, Random, 95% CI)	0.98 [0.47, 2.05]
1.16.5 Males 15‐25 years	3	5278	Risk Ratio (M‐H, Random, 95% CI)	0.76 [0.34, 1.72]
1.17 Treatment for HPV‐related pre‐invasive disease	5	38604	Risk Ratio (M‐H, Random, 95% CI)	0.76 [0.65, 0.89]
1.17.1 Females 14 years or younger	0	0	Risk Ratio (M‐H, Random, 95% CI)	Not estimable
1.17.2 Females 15‐25 years	2	25488	Risk Ratio (M‐H, Random, 95% CI)	0.76 [0.59, 0.98]
1.17.3 Females 25 years or older	2	9236	Risk Ratio (M‐H, Random, 95% CI)	0.87 [0.74, 1.03]
1.17.4 Females and males	0	0	Risk Ratio (M‐H, Random, 95% CI)	Not estimable
1.17.5 Males 15‐25 years	1	3880	Risk Ratio (M‐H, Random, 95% CI)	0.59 [0.45, 0.78]
1.18 Anogenital warts irrespective of HPV type	2	21271	Risk Ratio (M‐H, Random, 95% CI)	0.38 [0.32, 0.46]
1.18.1 Females 14 years or younger	0	0	Risk Ratio (M‐H, Random, 95% CI)	Not estimable
1.18.2 Females 15‐25 years	1	17391	Risk Ratio (M‐H, Random, 95% CI)	0.38 [0.31, 0.47]
1.18.3 Females 25 years or older	0	0	Risk Ratio (M‐H, Random, 95% CI)	Not estimable
1.18.4 Females and males	0	0	Risk Ratio (M‐H, Random, 95% CI)	Not estimable
1.18.5 Males 15‐25 years	1	3880	Risk Ratio (M‐H, Random, 95% CI)	0.39 [0.26, 0.58]
1.19 Anogenital warts associated with vaccine‐matched HPV types	4	25179	Risk Ratio (M‐H, Random, 95% CI)	0.28 [0.18, 0.45]
1.19.1 Females 14 years or younger	0	0	Risk Ratio (M‐H, Random, 95% CI)	Not estimable
1.19.2 Females 15‐25 years	2	17917	Risk Ratio (M‐H, Random, 95% CI)	0.21 [0.16, 0.27]
1.19.3 Females 25 years or older	1	3382	Risk Ratio (M‐H, Random, 95% CI)	0.58 [0.23, 1.47]
1.19.4 Females and males	0	0	Risk Ratio (M‐H, Random, 95% CI)	Not estimable
1.19.5 Males 15‐25 years	1	3880	Risk Ratio (M‐H, Random, 95% CI)	0.32 [0.20, 0.51]
1.20 Adverse pregnancy outcome: any adverse pregnancy outcome	2	5281	Risk Ratio (M‐H, Random, 95% CI)	1.10 [0.72, 1.69]
1.20.1 Females 14 years or younger	0	0	Risk Ratio (M‐H, Random, 95% CI)	Not estimable
1.20.2 Females 15‐25 years	1	2275	Risk Ratio (M‐H, Random, 95% CI)	1.53 [0.84, 2.78]
1.20.3 Females 25 years or older	1	3006	Risk Ratio (M‐H, Random, 95% CI)	0.96 [0.82, 1.11]
1.21 Adverse pregnancy outcome: infant/fetal abnormality	12	71662	Risk Ratio (M‐H, Random, 95% CI)	1.20 [0.84, 1.73]
1.21.1 Females 14 years or younger	1	107	Risk Ratio (M‐H, Random, 95% CI)	0.94 [0.04, 22.38]
1.21.2 Females 15‐25 years	7	51913	Risk Ratio (M‐H, Random, 95% CI)	1.28 [0.71, 2.30]
1.21.3 Females 25 years or older	4	19642	Risk Ratio (M‐H, Random, 95% CI)	1.08 [0.61, 1.90]
1.22 Adverse pregnancy outcome: cervical incompetence	1	12157	Risk Ratio (M‐H, Random, 95% CI)	0.20 [0.01, 4.16]
1.22.1 Females 14 years or younger	0	0	Risk Ratio (M‐H, Random, 95% CI)	Not estimable
1.22.2 Females 15‐25 years	1	12157	Risk Ratio (M‐H, Random, 95% CI)	0.20 [0.01, 4.16]
1.22.3 Females 25 years or older	0	0	Risk Ratio (M‐H, Random, 95% CI)	Not estimable
1.23 Adverse pregnancy outcome: miscarriage	17	76019	Risk Ratio (M‐H, Random, 95% CI)	1.01 [0.93, 1.10]
1.23.1 Females 14 years or younger	1	2067	Risk Ratio (M‐H, Random, 95% CI)	Not estimable
1.23.2 Females 15‐25 years	12	54310	Risk Ratio (M‐H, Random, 95% CI)	1.01 [0.92, 1.11]
1.23.3 Females 25 years or older	4	19642	Risk Ratio (M‐H, Random, 95% CI)	1.03 [0.85, 1.26]
1.24 Adverse pregnancy outcome: pre‐term birth	10	62017	Risk Ratio (M‐H, Random, 95% CI)	1.13 [0.84, 1.52]
1.24.1 Females 14 years or younger	0	0	Risk Ratio (M‐H, Random, 95% CI)	Not estimable
1.24.2 Females 15‐25 years	8	52492	Risk Ratio (M‐H, Random, 95% CI)	1.14 [0.85, 1.54]
1.24.3 Females 25 years or older	2	9525	Risk Ratio (M‐H, Random, 95% CI)	0.33 [0.01, 8.17]
1.25 Adverse pregnancy outcome: PROM	4	30715	Risk Ratio (M‐H, Random, 95% CI)	0.72 [0.36, 1.42]
1.25.1 Females 14 years or younger	0	0	Risk Ratio (M‐H, Random, 95% CI)	Not estimable
1.25.2 Females 15‐25 years	3	24968	Risk Ratio (M‐H, Random, 95% CI)	0.67 [0.34, 1.35]
1.25.3 Females 25 years or older	1	5747	Risk Ratio (M‐H, Random, 95% CI)	2.99 [0.12, 73.43]
1.26 Adverse pregnancy outcome: stillbirth/late fetal death	10	64089	Risk Ratio (M‐H, Random, 95% CI)	1.08 [0.71, 1.63]
1.26.1 Females 14 years or younger	0	0	Risk Ratio (M‐H, Random, 95% CI)	Not estimable
1.26.2 Females 15‐25 years	6	44447	Risk Ratio (M‐H, Random, 95% CI)	1.26 [0.76, 2.08]
1.26.3 Females 25 years or older	4	19642	Risk Ratio (M‐H, Random, 95% CI)	0.79 [0.38, 1.62]
1.27 Local adverse events: any	34	61326	Risk Ratio (M‐H, Random, 95% CI)	1.26 [1.21, 1.32]
1.27.1 Females 14 years or younger	7	4918	Risk Ratio (M‐H, Random, 95% CI)	1.28 [1.21, 1.37]
1.27.2 Females 15‐25 years	16	26400	Risk Ratio (M‐H, Random, 95% CI)	1.20 [1.14, 1.25]
1.27.3 Females 25 years or older	5	20718	Risk Ratio (M‐H, Random, 95% CI)	1.26 [1.11, 1.43]
1.27.4 Females and males	3	4014	Risk Ratio (M‐H, Random, 95% CI)	1.65 [1.41, 1.93]
1.27.5 Males 15‐25 years	3	5276	Risk Ratio (M‐H, Random, 95% CI)	1.26 [1.04, 1.52]
1.28 Local adverse events: pain	33	72225	Risk Ratio (M‐H, Random, 95% CI)	1.30 [1.24, 1.36]
1.28.1 Females 14 years or younger	6	4107	Risk Ratio (M‐H, Random, 95% CI)	1.33 [1.22, 1.45]
1.28.2 Females 15‐25 years	16	37292	Risk Ratio (M‐H, Random, 95% CI)	1.28 [1.20, 1.37]
1.28.3 Females 25 years or older	5	20718	Risk Ratio (M‐H, Random, 95% CI)	1.29 [1.15, 1.44]
1.28.4 Females and males	3	4832	Risk Ratio (M‐H, Random, 95% CI)	1.46 [1.18, 1.82]
1.28.5 Males 15‐25 years	3	5276	Risk Ratio (M‐H, Random, 95% CI)	1.31 [1.05, 1.64]
1.29 Local adverse events: redness	31	69066	Risk Ratio (M‐H, Random, 95% CI)	1.51 [1.39, 1.65]
1.29.1 Females 14 years or younger	6	4107	Risk Ratio (M‐H, Random, 95% CI)	1.88 [1.64, 2.16]
1.29.2 Females 15‐25 years	15	36616	Risk Ratio (M‐H, Random, 95% CI)	1.54 [1.47, 1.62]
1.29.3 Females 25 years or older	5	20718	Risk Ratio (M‐H, Random, 95% CI)	1.36 [0.94, 1.97]
1.29.4 Females and males	2	2349	Risk Ratio (M‐H, Random, 95% CI)	1.54 [1.22, 1.95]
1.29.5 Males 15‐25 years	3	5276	Risk Ratio (M‐H, Random, 95% CI)	1.15 [1.01, 1.31]
1.30 Local adverse events: swelling	33	72225	Risk Ratio (M‐H, Random, 95% CI)	1.86 [1.65, 2.11]
1.30.1 Females 14 years or younger	6	4107	Risk Ratio (M‐H, Random, 95% CI)	2.20 [1.77, 2.73]
1.30.2 Females 15‐25 years	16	37292	Risk Ratio (M‐H, Random, 95% CI)	1.91 [1.72, 2.13]
1.30.3 Females 25 years or older	5	20718	Risk Ratio (M‐H, Random, 95% CI)	1.67 [0.99, 2.82]
1.30.4 Females and males	3	4832	Risk Ratio (M‐H, Random, 95% CI)	2.20 [1.11, 4.32]
1.30.5 Males 15‐25 years	3	5276	Risk Ratio (M‐H, Random, 95% CI)	1.40 [1.06, 1.85]
1.31 Systemic adverse events	34	55349	Risk Ratio (M‐H, Random, 95% CI)	1.07 [1.04, 1.10]
1.31.1 Females 14 years or younger	7	4918	Risk Ratio (M‐H, Random, 95% CI)	1.11 [1.07, 1.15]
1.31.2 Females 15‐25 years	16	20419	Risk Ratio (M‐H, Random, 95% CI)	1.04 [1.01, 1.08]
1.31.3 Females 25 years or older	5	20721	Risk Ratio (M‐H, Random, 95% CI)	1.09 [1.01, 1.17]
1.31.4 Females and males	3	4015	Risk Ratio (M‐H, Random, 95% CI)	1.15 [0.96, 1.38]
1.31.5 Males 15‐25 years	3	5276	Risk Ratio (M‐H, Random, 95% CI)	1.03 [0.95, 1.11]
1.32 Adverse events	28	42942	Risk Ratio (M‐H, Random, 95% CI)	1.09 [1.07, 1.12]
1.32.1 Females 14 years or younger	6	2362	Risk Ratio (M‐H, Random, 95% CI)	1.11 [1.06, 1.15]
1.32.2 Females 15‐25 years	12	16821	Risk Ratio (M‐H, Random, 95% CI)	1.05 [1.03, 1.07]
1.32.3 Females 25 years or older	4	13651	Risk Ratio (M‐H, Random, 95% CI)	1.16 [1.07, 1.26]
1.32.4 Females and males	3	4832	Risk Ratio (M‐H, Random, 95% CI)	1.17 [1.07, 1.27]
1.32.5 Males 15‐25 years	3	5276	Risk Ratio (M‐H, Random, 95% CI)	1.09 [1.05, 1.13]
1.33 Unsolicited adverse events	20	39592	Risk Ratio (M‐H, Random, 95% CI)	1.04 [0.99, 1.09]
1.33.1 Females 14 years or younger	6	4838	Risk Ratio (M‐H, Random, 95% CI)	1.08 [0.95, 1.22]
1.33.2 Females 15‐25 years	9	18763	Risk Ratio (M‐H, Random, 95% CI)	1.04 [0.96, 1.12]
1.33.3 Females 25 years or older	3	14031	Risk Ratio (M‐H, Random, 95% CI)	1.04 [0.94, 1.15]
1.33.4 Females and males	1	1692	Risk Ratio (M‐H, Random, 95% CI)	1.01 [0.86, 1.19]
1.33.5 Males 15‐25 years	1	268	Risk Ratio (M‐H, Random, 95% CI)	1.07 [0.76, 1.51]
1.34 Adverse events that lead to discontinuation	30	59033	Risk Ratio (M‐H, Random, 95% CI)	0.98 [0.64, 1.50]
1.34.1 Females 14 years or younger	6	4195	Risk Ratio (M‐H, Random, 95% CI)	0.20 [0.02, 1.71]
1.34.2 Females 15‐25 years	14	31051	Risk Ratio (M‐H, Random, 95% CI)	1.11 [0.52, 2.36]
1.34.3 Females 25 years or older	4	13734	Risk Ratio (M‐H, Random, 95% CI)	1.84 [1.09, 3.11]
1.34.4 Females and males	3	4775	Risk Ratio (M‐H, Random, 95% CI)	0.72 [0.32, 1.63]
1.34.5 Males 15‐25 years	3	5278	Risk Ratio (M‐H, Random, 95% CI)	0.37 [0.15, 0.93]
1.35 Specific adverse events: paralysis	3	12838	Risk Ratio (M‐H, Random, 95% CI)	1.00 [0.20, 4.95]
1.35.1 Females 14 years or younger	0	0	Risk Ratio (M‐H, Random, 95% CI)	Not estimable
1.35.2 Females 15‐25 years	2	7091	Risk Ratio (M‐H, Random, 95% CI)	3.01 [0.31, 28.86]
1.35.3 Females 25 years or older	1	5747	Risk Ratio (M‐H, Random, 95% CI)	0.33 [0.03, 3.19]
1.35.4 Females and males	0	0	Risk Ratio (M‐H, Random, 95% CI)	Not estimable
1.35.5 Males 15‐25 years	0	0	Risk Ratio (M‐H, Random, 95% CI)	Not estimable
1.36 Specific adverse events: ovarian failure	1	5747	Risk Ratio (M‐H, Random, 95% CI)	2.99 [0.12, 73.43]
1.36.1 Females 14 years or younger	0	0	Risk Ratio (M‐H, Random, 95% CI)	Not estimable
1.36.2 Females 15‐25 years	0	0	Risk Ratio (M‐H, Random, 95% CI)	Not estimable
1.36.3 Females 25 years or older	1	5747	Risk Ratio (M‐H, Random, 95% CI)	2.99 [0.12, 73.43]
1.37 Specific adverse events: infertility	1	18644	Risk Ratio (M‐H, Random, 95% CI)	2.00 [0.18, 22.07]
1.37.1 Females 14 years or younger	0	0	Risk Ratio (M‐H, Random, 95% CI)	Not estimable
1.37.2 Females 15‐25 years	1	18644	Risk Ratio (M‐H, Random, 95% CI)	2.00 [0.18, 22.07]
1.37.3 Females 25 years or older	0	0	Risk Ratio (M‐H, Random, 95% CI)	Not estimable
1.37.4 Females and males	0	0	Risk Ratio (M‐H, Random, 95% CI)	Not estimable
1.37.5 Males 15‐25 years	0	0	Risk Ratio (M‐H, Random, 95% CI)	Not estimable
1.38 Specific adverse events: sexual behaviour: chlamydia infection	9	34911	Risk Ratio (M‐H, Random, 95% CI)	0.97 [0.90, 1.05]
1.38.1 Females 14 years or younger	0	0	Risk Ratio (M‐H, Random, 95% CI)	Not estimable
1.38.2 Females 15‐25 years	5	22255	Risk Ratio (M‐H, Random, 95% CI)	0.98 [0.90, 1.06]
1.38.3 Females 25 years or older	2	8749	Risk Ratio (M‐H, Random, 95% CI)	0.80 [0.42, 1.54]
1.38.4 Females and males	0	0	Risk Ratio (M‐H, Random, 95% CI)	Not estimable
1.38.5 Males 15‐25 years	2	3907	Risk Ratio (M‐H, Random, 95% CI)	1.88 [0.42, 8.38]
1.39 Specific adverse events: sexual behaviour: gonorrhoea infection	3	25854	Risk Ratio (M‐H, Random, 95% CI)	1.10 [0.74, 1.63]
1.39.1 Females 14 years or younger	0	0	Risk Ratio (M‐H, Random, 95% CI)	Not estimable
1.39.2 Females 15‐25 years	2	20102	Risk Ratio (M‐H, Random, 95% CI)	1.08 [0.72, 1.61]
1.39.3 Females 25 years or older	1	5752	Risk Ratio (M‐H, Random, 95% CI)	1.99 [0.18, 21.97]
1.39.4 Females and males	0	0	Risk Ratio (M‐H, Random, 95% CI)	Not estimable
1.39.5 Males 15‐25 years	0	0	Risk Ratio (M‐H, Random, 95% CI)	Not estimable
1.40 Specific adverse events: sexual behaviour: herpes infection	5	33339	Risk Ratio (M‐H, Random, 95% CI)	0.85 [0.61, 1.20]
1.40.1 Females 14 years or younger	0	0	Risk Ratio (M‐H, Random, 95% CI)	Not estimable
1.40.2 Females 15‐25 years	4	27587	Risk Ratio (M‐H, Random, 95% CI)	1.33 [0.51, 3.48]
1.40.3 Females 25 years or older	1	5752	Risk Ratio (M‐H, Random, 95% CI)	0.72 [0.29, 1.80]
1.40.4 Females and males	0	0	Risk Ratio (M‐H, Random, 95% CI)	Not estimable
1.40.5 Males 15‐25 years	0	0	Risk Ratio (M‐H, Random, 95% CI)	Not estimable
1.41 Specific adverse events: sexual behaviour: HIV infection	1	18644	Risk Ratio (M‐H, Random, 95% CI)	0.33 [0.01, 8.19]
1.41.1 Females 14 years or younger	0	0	Risk Ratio (M‐H, Random, 95% CI)	Not estimable
1.41.2 Females 15‐25 years	1	18644	Risk Ratio (M‐H, Random, 95% CI)	0.33 [0.01, 8.19]
1.41.3 Females 25 years or older	0	0	Risk Ratio (M‐H, Random, 95% CI)	Not estimable
1.41.4 Females and males	0	0	Risk Ratio (M‐H, Random, 95% CI)	Not estimable
1.41.5 Males 15‐25 years	0	0	Risk Ratio (M‐H, Random, 95% CI)	Not estimable
1.42 All‐cause mortality	31	91127	Risk Ratio (M‐H, Random, 95% CI)	1.14 [0.71, 1.83]
1.42.1 Females 14 years or younger	6	4195	Risk Ratio (M‐H, Random, 95% CI)	Not estimable
1.42.2 Females 15‐25 years	15	56673	Risk Ratio (M‐H, Random, 95% CI)	0.97 [0.58, 1.64]
1.42.3 Females 25 years or older	5	20806	Risk Ratio (M‐H, Random, 95% CI)	2.55 [1.20, 5.41]
1.42.4 Females and males	2	4175	Risk Ratio (M‐H, Random, 95% CI)	Not estimable
1.42.5 Males 15‐25 years	3	5278	Risk Ratio (M‐H, Random, 95% CI)	0.31 [0.09, 1.01]
1.43 Incident HPV 16 and/or 18 infection	7	36147	Risk Ratio (M‐H, Random, 95% CI)	0.26 [0.23, 0.31]
1.43.1 Females 14 years or younger	0	0	Risk Ratio (M‐H, Random, 95% CI)	Not estimable
1.43.2 Females 15‐25 years	6	29368	Risk Ratio (M‐H, Random, 95% CI)	0.26 [0.22, 0.31]
1.43.3 Females 25 years or older	1	6779	Risk Ratio (M‐H, Random, 95% CI)	0.29 [0.21, 0.41]
1.43.4 Females and males	0	0	Risk Ratio (M‐H, Random, 95% CI)	Not estimable
1.43.5 Males 15‐25 years	0	0	Risk Ratio (M‐H, Random, 95% CI)	Not estimable
1.44 12‐month persistent HPV 16 and/or 18 infection	6	35130	Risk Ratio (M‐H, Random, 95% CI)	0.14 [0.05, 0.37]
1.44.1 Females 14 years or younger	0	0	Risk Ratio (M‐H, Random, 95% CI)	Not estimable
1.44.2 Females 15‐25 years	5	29697	Risk Ratio (M‐H, Random, 95% CI)	0.09 [0.02, 0.38]
1.44.3 Females 25 years or older	1	5433	Risk Ratio (M‐H, Random, 95% CI)	0.42 [0.30, 0.58]
1.44.4 Females and males	0	0	Risk Ratio (M‐H, Random, 95% CI)	Not estimable
1.44.5 Males 15‐25 years	0	0	Risk Ratio (M‐H, Random, 95% CI)	Not estimable
1.45 6‐month persistent HPV 16 and/or 18 infection	8	45181	Risk Ratio (M‐H, Random, 95% CI)	0.18 [0.09, 0.34]
1.45.1 Females 14 years or younger	0	0	Risk Ratio (M‐H, Random, 95% CI)	Not estimable
1.45.2 Females 15‐25 years	6	32602	Risk Ratio (M‐H, Random, 95% CI)	0.14 [0.05, 0.35]
1.45.3 Females 25 years or older	2	12579	Risk Ratio (M‐H, Random, 95% CI)	0.32 [0.17, 0.58]
1.45.4 Females and males	0	0	Risk Ratio (M‐H, Random, 95% CI)	Not estimable
1.45.5 Males 15‐25 years	0	0	Risk Ratio (M‐H, Random, 95% CI)	Not estimable

**Comparison 2:** Cervarix versus control

Outcome or subgroup title	No. of studies	No. of participants	Statistical method	Effect size
2.1 CIN3+ irrespective of HPV type	4		Risk Ratio (M‐H, Random, 95% CI)	Subtotals only
2.1.1 Females 14 years or younger	0	0	Risk Ratio (M‐H, Random, 95% CI)	Not estimable
2.1.2 Females 15‐25 years	4	26741	Risk Ratio (M‐H, Random, 95% CI)	0.76 [0.41, 1.41]
2.1.3 Females 25 years or older	0	0	Risk Ratio (M‐H, Random, 95% CI)	Not estimable
2.2 CIN3+ associated with HPV 16 and/or 18	2		Risk Ratio (M‐H, Random, 95% CI)	Subtotals only
2.2.1 Females 14 years or younger	0	0	Risk Ratio (M‐H, Random, 95% CI)	Not estimable
2.2.2 Females 15‐25 years	2	18495	Risk Ratio (M‐H, Random, 95% CI)	0.54 [0.39, 0.76]
2.2.3 Females 25 years or older	0	0	Risk Ratio (M‐H, Random, 95% CI)	Not estimable
2.3 CIN2+ irrespective of HPV type	5		Risk Ratio (M‐H, Random, 95% CI)	Subtotals only
2.3.1 Females 14 years or younger	0	0	Risk Ratio (M‐H, Random, 95% CI)	Not estimable
2.3.2 Females 15‐25 years	4	29464	Risk Ratio (M‐H, Random, 95% CI)	0.63 [0.44, 0.90]
2.3.3 Females 25 years or older	1	5468	Risk Ratio (M‐H, Random, 95% CI)	0.95 [0.73, 1.24]
2.4 CIN2+ associated with HPV 16 and/or 18	6		Risk Ratio (M‐H, Random, 95% CI)	Subtotals only
2.4.1 Females 14 years or younger	0	0	Risk Ratio (M‐H, Random, 95% CI)	Not estimable
2.4.2 Females 15‐25 years	5	30302	Risk Ratio (M‐H, Random, 95% CI)	0.33 [0.21, 0.50]
2.4.3 Females 25 years or older	1	5477	Risk Ratio (M‐H, Random, 95% CI)	0.71 [0.45, 1.11]
2.5 Adenocarcinoma in situ irrespective of HPV type	1		Risk Ratio (M‐H, Random, 95% CI)	Subtotals only
2.5.1 Females 14 years or younger	0	0	Risk Ratio (M‐H, Random, 95% CI)	Not estimable
2.5.2 Females 15‐25 years	1	17402	Risk Ratio (M‐H, Random, 95% CI)	0.23 [0.07, 0.81]
2.5.3 Females 25 years or older	0	0	Risk Ratio (M‐H, Random, 95% CI)	Not estimable
2.5.4 Females and males	0	0	Risk Ratio (M‐H, Random, 95% CI)	Not estimable
2.5.5 Males 15‐25 years	0	0	Risk Ratio (M‐H, Random, 95% CI)	Not estimable
2.6 Adenocarcinoma in situ associated with HPV 16 and/or 18	1		Risk Ratio (M‐H, Random, 95% CI)	Subtotals only
2.6.1 Females 14 years or younger	0	0	Risk Ratio (M‐H, Random, 95% CI)	Not estimable
2.6.2 Females 15‐25 years	1	17402	Risk Ratio (M‐H, Random, 95% CI)	0.30 [0.08, 1.09]
2.6.3 Females 25 years or older	0	0	Risk Ratio (M‐H, Random, 95% CI)	Not estimable
2.7 CIN3 associated with HPV 16 and/or 18	1		Risk Ratio (M‐H, Random, 95% CI)	Subtotals only
2.7.1 Females 14 years or younger	0	0	Risk Ratio (M‐H, Random, 95% CI)	Not estimable
2.7.2 Females 15‐25 years	1	1113	Risk Ratio (M‐H, Random, 95% CI)	Not estimable
2.7.3 Females 25 years or older	0	0	Risk Ratio (M‐H, Random, 95% CI)	Not estimable
2.8 CIN2 associated with HPV 16 and/or 18	1		Risk Ratio (M‐H, Random, 95% CI)	Subtotals only
2.8.1 Females 14 years or younger	0	0	Risk Ratio (M‐H, Random, 95% CI)	Not estimable
2.8.2 Females 15‐25 years	1	1113	Risk Ratio (M‐H, Random, 95% CI)	0.14 [0.01, 2.72]
2.8.3 Females 25 years or older	0	0	Risk Ratio (M‐H, Random, 95% CI)	Not estimable
2.9 High‐grade VIN or VaIN irrespective of HPV type	1		Risk Ratio (M‐H, Random, 95% CI)	Subtotals only
2.9.1 Females 14 years or younger	0	0	Risk Ratio (M‐H, Random, 95% CI)	Not estimable
2.9.2 Females 15‐25 years	1	15701	Risk Ratio (M‐H, Random, 95% CI)	0.43 [0.20, 0.93]
2.9.3 Females 25 years or older	0	0	Risk Ratio (M‐H, Random, 95% CI)	Not estimable
2.10 High‐grade VIN or VaIN associated with HPV 16 and/or 18	1		Risk Ratio (M‐H, Random, 95% CI)	Subtotals only
2.10.1 Females 14 years or younger	0	0	Risk Ratio (M‐H, Random, 95% CI)	Not estimable
2.10.2 Females 15‐25 years	1	15566	Risk Ratio (M‐H, Random, 95% CI)	0.28 [0.06, 1.37]
2.10.3 Females 25 years or older	0	0	Risk Ratio (M‐H, Random, 95% CI)	Not estimable
2.11 Serious adverse events	21		Risk Ratio (M‐H, Random, 95% CI)	Subtotals only
2.11.1 Females 14 years or younger	6	4838	Risk Ratio (M‐H, Random, 95% CI)	1.00 [0.62, 1.60]
2.11.2 Females 15‐25 years	12	37802	Risk Ratio (M‐H, Random, 95% CI)	1.00 [0.94, 1.06]
2.11.3 Females 25 years or older	2	6959	Risk Ratio (M‐H, Random, 95% CI)	1.07 [0.92, 1.26]
2.11.4 Females and males	0	0	Risk Ratio (M‐H, Random, 95% CI)	Not estimable
2.11.5 Males 15‐25 years	1	270	Risk Ratio (M‐H, Random, 95% CI)	1.48 [0.16, 13.98]
2.12 Treatment for HPV‐related pre‐invasive disease	3		Risk Ratio (M‐H, Random, 95% CI)	Subtotals only
2.12.1 Females 14 years or younger	0	0	Risk Ratio (M‐H, Random, 95% CI)	Not estimable
2.12.2 Females 15‐25 years	2	25488	Risk Ratio (M‐H, Random, 95% CI)	0.76 [0.59, 0.98]
2.12.3 Females 25 years or older	1	5468	Risk Ratio (M‐H, Random, 95% CI)	0.80 [0.61, 1.05]
2.12.4 Females and males	0	0	Risk Ratio (M‐H, Random, 95% CI)	Not estimable
2.12.5 Males 15‐25 years	0	0	Risk Ratio (M‐H, Random, 95% CI)	Not estimable
2.13 Cervical screening attendance	1		Risk Ratio (M‐H, Random, 95% CI)	Subtotals only
2.13.1 Females 14 years or younger	0	0	Risk Ratio (M‐H, Random, 95% CI)	Not estimable
2.13.2 Females 15‐25 years	1	832	Risk Ratio (M‐H, Random, 95% CI)	1.07 [1.00, 1.15]
2.13.3 Females 25 years or older	0	0	Risk Ratio (M‐H, Random, 95% CI)	Not estimable
2.14 12‐month persistent HPV 16 and/or 18 infection	6		Risk Ratio (M‐H, Random, 95% CI)	Subtotals only
2.14.1 Females 14 years or younger	0	0	Risk Ratio (M‐H, Random, 95% CI)	Not estimable
2.14.2 Females 15‐25 years	5	29697	Risk Ratio (M‐H, Random, 95% CI)	0.09 [0.02, 0.38]
2.14.3 Females 25 years or older	1	5433	Risk Ratio (M‐H, Random, 95% CI)	0.42 [0.30, 0.58]
2.14.4 Females and males	0	0	Risk Ratio (M‐H, Random, 95% CI)	Not estimable
2.14.5 Males 15‐25 years	0	0	Risk Ratio (M‐H, Random, 95% CI)	Not estimable
2.15 6‐month persistent HPV 16 and/or 18 infection	7		Risk Ratio (M‐H, Random, 95% CI)	Subtotals only
2.15.1 Females 14 years or younger	0	0	Risk Ratio (M‐H, Random, 95% CI)	Not estimable
2.15.2 Females 15‐25 years	6	31844	Risk Ratio (M‐H, Random, 95% CI)	0.13 [0.05, 0.37]
2.15.3 Females 25 years or older	1	5537	Risk Ratio (M‐H, Random, 95% CI)	0.41 [0.32, 0.54]
2.15.4 Females and males	0	0	Risk Ratio (M‐H, Random, 95% CI)	Not estimable
2.15.5 Males 15‐25 years	0	0	Risk Ratio (M‐H, Random, 95% CI)	Not estimable
2.16 Incident HPV 16 and/or 18 infection	6		Risk Ratio (M‐H, Random, 95% CI)	Subtotals only
2.16.1 Females 14 years or younger	0	0	Risk Ratio (M‐H, Random, 95% CI)	Not estimable
2.16.2 Females 15‐25 years	6	29368	Risk Ratio (M‐H, Random, 95% CI)	0.26 [0.22, 0.31]
2.16.3 Females 25 years or older	0	0	Risk Ratio (M‐H, Random, 95% CI)	Not estimable
2.16.4 Females and males	0	0	Risk Ratio (M‐H, Random, 95% CI)	Not estimable
2.16.5 Males 15‐25 years	0	0	Risk Ratio (M‐H, Random, 95% CI)	Not estimable
2.17 Adverse pregnancy outcome: any adverse pregnancy outcome	1		Risk Ratio (M‐H, Random, 95% CI)	Subtotals only
2.17.1 Females 14 years or younger	0	0	Risk Ratio (M‐H, Random, 95% CI)	Not estimable
2.17.2 Females 15‐25 years	1	1517	Risk Ratio (M‐H, Random, 95% CI)	1.35 [0.68, 2.68]
2.17.3 Females 25 years or older	0	0	Risk Ratio (M‐H, Random, 95% CI)	Not estimable
2.18 Adverse pregnancy outcome: fetal abnormality	6		Risk Ratio (M‐H, Random, 95% CI)	Subtotals only
2.18.1 Females 14 years or younger	0	0	Risk Ratio (M‐H, Random, 95% CI)	Not estimable
2.18.2 Females 15‐25 years	5	34314	Risk Ratio (M‐H, Random, 95% CI)	1.07 [0.36, 3.16]
2.18.3 Females 25 years or older	1	5747	Risk Ratio (M‐H, Random, 95% CI)	0.62 [0.20, 1.90]
2.19 Adverse pregnancy outcome: miscarriage	11		Risk Ratio (M‐H, Random, 95% CI)	Subtotals only
2.19.1 Females 14 years or younger	1	2067	Risk Ratio (M‐H, Random, 95% CI)	Not estimable
2.19.2 Females 15‐25 years	9	35763	Risk Ratio (M‐H, Random, 95% CI)	1.06 [0.95, 1.19]
2.19.3 Females 25 years or older	1	5747	Risk Ratio (M‐H, Random, 95% CI)	1.03 [0.74, 1.43]
2.20 Adverse pregnancy outcome: pre‐term birth	7		Risk Ratio (M‐H, Random, 95% CI)	Subtotals only
2.20.1 Females 14 years or younger	0	0	Risk Ratio (M‐H, Random, 95% CI)	Not estimable
2.20.2 Females 15‐25 years	6	34990	Risk Ratio (M‐H, Random, 95% CI)	1.17 [0.86, 1.59]
2.20.3 Females 25 years or older	1	5747	Risk Ratio (M‐H, Random, 95% CI)	Not estimable
2.21 Adverse pregnancy outcome: PROM	2		Risk Ratio (M‐H, Random, 95% CI)	Subtotals only
2.21.1 Females 14 years or younger	0	0	Risk Ratio (M‐H, Random, 95% CI)	Not estimable
2.21.2 Females 15‐25 years	1	7466	Risk Ratio (M‐H, Random, 95% CI)	0.58 [0.28, 1.22]
2.21.3 Females 25 years or older	1	5747	Risk Ratio (M‐H, Random, 95% CI)	2.99 [0.12, 73.43]
2.22 Adverse pregnancy outcome: stillbirth	5		Risk Ratio (M‐H, Random, 95% CI)	Subtotals only
2.22.1 Females 14 years or younger	0	0	Risk Ratio (M‐H, Random, 95% CI)	Not estimable
2.22.2 Females 15‐25 years	4	26848	Risk Ratio (M‐H, Random, 95% CI)	1.29 [0.64, 2.60]
2.22.3 Females 25 years or older	1	5747	Risk Ratio (M‐H, Random, 95% CI)	0.20 [0.01, 4.15]
2.23 Local adverse events: any	20		Risk Ratio (M‐H, Random, 95% CI)	Subtotals only
2.23.1 Females 14 years or younger	6	4820	Risk Ratio (M‐H, Random, 95% CI)	1.28 [1.20, 1.37]
2.23.2 Females 15‐25 years	11	16965	Risk Ratio (M‐H, Random, 95% CI)	1.24 [1.16, 1.32]
2.23.3 Females 25 years or older	2	6871	Risk Ratio (M‐H, Random, 95% CI)	1.42 [1.14, 1.77]
2.23.4 Females and males	0	0	Risk Ratio (M‐H, Random, 95% CI)	Not estimable
2.23.5 Males 15‐25 years	1	268	Risk Ratio (M‐H, Random, 95% CI)	1.83 [1.47, 2.28]
2.24 Local adverse events: pain	19		Risk Ratio (M‐H, Random, 95% CI)	Subtotals only
2.24.1 Females 14 years or younger	5	4009	Risk Ratio (M‐H, Random, 95% CI)	1.33 [1.22, 1.45]
2.24.2 Females 15‐25 years	11	16965	Risk Ratio (M‐H, Random, 95% CI)	1.32 [1.20, 1.45]
2.24.3 Females 25 years or older	2	6871	Risk Ratio (M‐H, Random, 95% CI)	1.43 [1.17, 1.76]
2.24.4 Females and males	0	0	Risk Ratio (M‐H, Random, 95% CI)	Not estimable
2.24.5 Males 15‐25 years	1	268	Risk Ratio (M‐H, Random, 95% CI)	1.99 [1.57, 2.53]
2.25 Local adverse events: redness	18		Risk Ratio (M‐H, Random, 95% CI)	Subtotals only
2.25.1 Females 14 years or younger	5	4009	Risk Ratio (M‐H, Random, 95% CI)	1.88 [1.61, 2.19]
2.25.2 Females 15‐25 years	10	16289	Risk Ratio (M‐H, Random, 95% CI)	1.58 [1.52, 1.65]
2.25.3 Females 25 years or older	2	6871	Risk Ratio (M‐H, Random, 95% CI)	1.97 [1.81, 2.14]
2.25.4 Females and males	0	0	Risk Ratio (M‐H, Random, 95% CI)	Not estimable
2.25.5 Males 15‐25 years	1	268	Risk Ratio (M‐H, Random, 95% CI)	1.66 [0.99, 2.79]
2.26 Local adverse events: swelling	19		Risk Ratio (M‐H, Random, 95% CI)	Subtotals only
2.26.1 Females 14 years or younger	5	4009	Risk Ratio (M‐H, Random, 95% CI)	2.25 [1.80, 2.80]
2.26.2 Females 15‐25 years	11	16965	Risk Ratio (M‐H, Random, 95% CI)	2.02 [1.82, 2.23]
2.26.3 Females 25 years or older	2	6871	Risk Ratio (M‐H, Random, 95% CI)	2.52 [2.30, 2.77]
2.26.4 Females and males	0	0	Risk Ratio (M‐H, Random, 95% CI)	Not estimable
2.26.5 Males 15‐25 years	1	268	Risk Ratio (M‐H, Random, 95% CI)	2.51 [1.17, 5.42]
2.27 Systemic adverse events: any	19		Risk Ratio (M‐H, Random, 95% CI)	Subtotals only
2.27.1 Females 14 years or younger	6	4820	Risk Ratio (M‐H, Random, 95% CI)	1.11 [1.07, 1.15]
2.27.2 Females 15‐25 years	10	10808	Risk Ratio (M‐H, Random, 95% CI)	1.06 [1.01, 1.11]
2.27.3 Females 25 years or older	2	6874	Risk Ratio (M‐H, Random, 95% CI)	1.27 [0.98, 1.65]
2.27.4 Females and males	0	0	Risk Ratio (M‐H, Random, 95% CI)	Not estimable
2.27.5 Males 15‐25 years	1	268	Risk Ratio (M‐H, Random, 95% CI)	1.10 [0.95, 1.28]
2.28 Adverse events	17		Risk Ratio (M‐H, Random, 95% CI)	Subtotals only
2.28.1 Females 14 years or younger	5	2264	Risk Ratio (M‐H, Random, 95% CI)	1.11 [1.06, 1.16]
2.28.2 Females 15‐25 years	9	15188	Risk Ratio (M‐H, Random, 95% CI)	1.05 [1.03, 1.07]
2.28.3 Females 25 years or older	2	6876	Risk Ratio (M‐H, Random, 95% CI)	1.28 [0.92, 1.76]
2.28.4 Females and males	0	0	Risk Ratio (M‐H, Random, 95% CI)	Not estimable
2.28.5 Males 15‐25 years	1	268	Risk Ratio (M‐H, Random, 95% CI)	1.14 [1.03, 1.25]
2.29 Unsolicited adverse events	17		Risk Ratio (M‐H, Random, 95% CI)	Subtotals only
2.29.1 Females 14 years or younger	6	4838	Risk Ratio (M‐H, Random, 95% CI)	1.08 [0.95, 1.22]
2.29.2 Females 15‐25 years	8	17169	Risk Ratio (M‐H, Random, 95% CI)	1.04 [0.95, 1.14]
2.29.3 Females 25 years or older	2	6959	Risk Ratio (M‐H, Random, 95% CI)	0.99 [0.93, 1.05]
2.29.4 Females and males	0	0	Risk Ratio (M‐H, Random, 95% CI)	Not estimable
2.29.5 Males 15‐25 years	1	268	Risk Ratio (M‐H, Random, 95% CI)	1.07 [0.76, 1.51]
2.30 Adverse events that led to discontinuation	19		Risk Ratio (M‐H, Random, 95% CI)	Subtotals only
2.30.1 Females 14 years or younger	5	4097	Risk Ratio (M‐H, Random, 95% CI)	0.20 [0.02, 1.71]
2.30.2 Females 15‐25 years	11	29274	Risk Ratio (M‐H, Random, 95% CI)	1.13 [0.51, 2.53]
2.30.3 Females 25 years or older	2	6959	Risk Ratio (M‐H, Random, 95% CI)	1.87 [1.04, 3.37]
2.30.4 Females and males	0	0	Risk Ratio (M‐H, Random, 95% CI)	Not estimable
2.30.5 Males 15‐25 years	1	270	Risk Ratio (M‐H, Random, 95% CI)	1.48 [0.06, 36.05]
2.31 Specific adverse events: paralysis	3		Risk Ratio (M‐H, Random, 95% CI)	Subtotals only
2.31.1 Females 14 years or younger	0	0	Risk Ratio (M‐H, Random, 95% CI)	Not estimable
2.31.2 Females 15‐25 years	2	7091	Risk Ratio (M‐H, Random, 95% CI)	3.01 [0.31, 28.86]
2.31.3 Females 25 years or older	1	5747	Risk Ratio (M‐H, Random, 95% CI)	0.33 [0.03, 3.19]
2.31.4 Females and males	0	0	Risk Ratio (M‐H, Random, 95% CI)	Not estimable
2.31.5 Males 15‐25 years	0	0	Risk Ratio (M‐H, Random, 95% CI)	Not estimable
2.32 Specific adverse events: ovarian failure	1		Risk Ratio (M‐H, Random, 95% CI)	Subtotals only
2.32.1 Females 14 years or younger	0	0	Risk Ratio (M‐H, Random, 95% CI)	Not estimable
2.32.2 Females 15‐25 years	0	0	Risk Ratio (M‐H, Random, 95% CI)	Not estimable
2.32.3 Females 25 years or older	1	5747	Risk Ratio (M‐H, Random, 95% CI)	2.99 [0.12, 73.43]
2.33 Specific adverse events: infertility	1		Risk Ratio (M‐H, Random, 95% CI)	Subtotals only
2.33.1 Females 14 years or younger	0	0	Risk Ratio (M‐H, Random, 95% CI)	Not estimable
2.33.2 Females 15‐25 years	1	18644	Risk Ratio (M‐H, Random, 95% CI)	2.00 [0.18, 22.07]
2.33.3 Females 25 years or older	0	0	Risk Ratio (M‐H, Random, 95% CI)	Not estimable
2.33.4 Females and males	0	0	Risk Ratio (M‐H, Random, 95% CI)	Not estimable
2.33.5 Males 15‐25 years	0	0	Risk Ratio (M‐H, Random, 95% CI)	Not estimable
2.34 Specific adverse events: sexual behaviour: chlamydia infection	7		Risk Ratio (M‐H, Random, 95% CI)	Subtotals only
2.34.1 Females 14 years or younger	0	0	Risk Ratio (M‐H, Random, 95% CI)	Not estimable
2.34.2 Females 15‐25 years	5	21759	Risk Ratio (M‐H, Random, 95% CI)	1.03 [0.87, 1.21]
2.34.3 Females 25 years or older	1	5752	Risk Ratio (M‐H, Random, 95% CI)	0.60 [0.14, 2.50]
2.34.4 Females and males	0	0	Risk Ratio (M‐H, Random, 95% CI)	Not estimable
2.34.5 Males 15‐25 years	1	270	Risk Ratio (M‐H, Random, 95% CI)	1.48 [0.06, 36.05]
2.35 Specific adverse events: sexual behaviour: genital herpes infection	5		Risk Ratio (M‐H, Random, 95% CI)	Subtotals only
2.35.1 Females 14 years or younger	0	0	Risk Ratio (M‐H, Random, 95% CI)	Not estimable
2.35.2 Females 15‐25 years	4	27587	Risk Ratio (M‐H, Random, 95% CI)	1.33 [0.51, 3.48]
2.35.3 Females 25 years or older	1	5752	Risk Ratio (M‐H, Random, 95% CI)	0.72 [0.29, 1.80]
2.35.4 Females and males	0	0	Risk Ratio (M‐H, Random, 95% CI)	Not estimable
2.35.5 Males 15‐25 years	0	0	Risk Ratio (M‐H, Random, 95% CI)	Not estimable
2.36 Specific adverse events: sexual behaviour: HIV infection	1	18644	Risk Ratio (M‐H, Random, 95% CI)	0.33 [0.01, 8.19]
2.36.1 Females 14 years or younger	0	0	Risk Ratio (M‐H, Random, 95% CI)	Not estimable
2.36.2 Females 15‐25 years	1	18644	Risk Ratio (M‐H, Random, 95% CI)	0.33 [0.01, 8.19]
2.36.3 Females 25 years or older	0	0	Risk Ratio (M‐H, Random, 95% CI)	Not estimable
2.36.4 Females and males	0	0	Risk Ratio (M‐H, Random, 95% CI)	Not estimable
2.36.5 Males 15‐25 years	0	0	Risk Ratio (M‐H, Random, 95% CI)	Not estimable
2.37 All‐cause mortality	19	47955	Risk Ratio (M‐H, Random, 95% CI)	1.21 [0.66, 2.22]
2.37.1 Females 14 years or younger	5	4097	Risk Ratio (M‐H, Random, 95% CI)	Not estimable
2.37.2 Females 15‐25 years	11	36629	Risk Ratio (M‐H, Random, 95% CI)	0.87 [0.47, 1.61]
2.37.3 Females 25 years or older	2	6959	Risk Ratio (M‐H, Random, 95% CI)	2.63 [0.99, 7.01]
2.37.4 Females and males	0	0	Risk Ratio (M‐H, Random, 95% CI)	Not estimable
2.37.5 Males 15‐25 years	1	270	Risk Ratio (M‐H, Random, 95% CI)	Not estimable

**Comparison 3:** Gardasil versus control

Outcome or subgroup title	No. of studies	No. of participants	Statistical method	Effect size
3.1 CIN3+ irrespective of HPV type	1		Risk Ratio (M‐H, Random, 95% CI)	Subtotals only
3.1.1 Females 14 years or younger	0	0	Risk Ratio (M‐H, Random, 95% CI)	Not estimable
3.1.2 Females 15‐25 years	1	17160	Risk Ratio (M‐H, Random, 95% CI)	0.81 [0.69, 0.96]
3.1.3 Females 25 years or older	0	0	Risk Ratio (M‐H, Random, 95% CI)	Not estimable
3.2 CIN3+ associated with HPV 6, 11, 16 and/or 18	1		Risk Ratio (M‐H, Random, 95% CI)	Subtotals only
3.2.1 Females 14 years or younger	0	0	Risk Ratio (M‐H, Random, 95% CI)	Not estimable
3.2.2 Females 15‐25 years	1	17160	Risk Ratio (M‐H, Random, 95% CI)	0.54 [0.43, 0.68]
3.2.3 Females 25 years or older	0	0	Risk Ratio (M‐H, Random, 95% CI)	Not estimable
3.3 CIN2+ irrespective of HPV type	2		Risk Ratio (M‐H, Random, 95% CI)	Subtotals only
3.3.1 Females 14 years or younger	0	0	Risk Ratio (M‐H, Random, 95% CI)	Not estimable
3.3.2 Females 15‐25 years	1	17160	Risk Ratio (M‐H, Random, 95% CI)	0.81 [0.72, 0.92]
3.3.3 Females 25 years or older	1	3819	Risk Ratio (M‐H, Random, 95% CI)	1.21 [0.84, 1.75]
3.4 CIN2+ associated with HPV 6, 11, 16 and/or 18	3		Risk Ratio (M‐H, Random, 95% CI)	Subtotals only
3.4.1 Females 14 years or younger	0	0	Risk Ratio (M‐H, Random, 95% CI)	Not estimable
3.4.2 Females 15‐25 years	1	17160	Risk Ratio (M‐H, Random, 95% CI)	0.50 [0.42, 0.59]
3.4.3 Females 25 years or older	2	6778	Risk Ratio (M‐H, Random, 95% CI)	0.74 [0.50, 1.09]
3.5 Invasive cervical cancer	2		Risk Ratio (M‐H, Random, 95% CI)	Subtotals only
3.5.1 Females 14 years or younger	0	0	Risk Ratio (M‐H, Random, 95% CI)	Not estimable
3.5.2 Females 15‐25 years	2	17622	Risk Ratio (M‐H, Random, 95% CI)	Not estimable
3.5.3 Females 25 years or older	0	0	Risk Ratio (M‐H, Random, 95% CI)	Not estimable
3.6 Adenocarcinoma in situ irrespective of HPV type	3		Risk Ratio (M‐H, Random, 95% CI)	Subtotals only
3.6.1 Females 14 years or younger	0	0	Risk Ratio (M‐H, Random, 95% CI)	Not estimable
3.6.2 Females 15‐25 years	2	17622	Risk Ratio (M‐H, Random, 95% CI)	0.44 [0.14, 1.40]
3.6.3 Females 25 years or older	0	0	Risk Ratio (M‐H, Random, 95% CI)	Not estimable
3.6.4 Females and males	0	0	Risk Ratio (M‐H, Random, 95% CI)	Not estimable
3.6.5 Males 15‐25 years	1	551	Risk Ratio (M‐H, Random, 95% CI)	Not estimable
3.7 Adenocarcinoma in situ associated with HPV 6, 11, 16 and/or 18	1		Risk Ratio (M‐H, Random, 95% CI)	Subtotals only
3.7.1 Females 14 years or younger	0	0	Risk Ratio (M‐H, Random, 95% CI)	Not estimable
3.7.2 Females 15‐25 years	1	17683	Risk Ratio (M‐H, Random, 95% CI)	0.40 [0.16, 1.03]
3.7.3 Females 25 years or older	0	0	Risk Ratio (M‐H, Random, 95% CI)	Not estimable
3.7.4 Females and males	0	0	Risk Ratio (M‐H, Random, 95% CI)	Not estimable
3.7.5 Males 15‐25 years	0	0	Risk Ratio (M‐H, Random, 95% CI)	Not estimable
3.8 CIN3 irrespective of HPV type	2		Risk Ratio (M‐H, Random, 95% CI)	Subtotals only
3.8.1 Females 14 years or younger	0	0	Risk Ratio (M‐H, Random, 95% CI)	Not estimable
3.8.2 Females 15‐25 years	2	17622	Risk Ratio (M‐H, Random, 95% CI)	0.91 [0.66, 1.27]
3.8.3 Females 25 years or older	0	0	Risk Ratio (M‐H, Random, 95% CI)	Not estimable
3.9 CIN3 associated with HPV 6, 11, 16 and/or 18	1		Risk Ratio (M‐H, Random, 95% CI)	Subtotals only
3.9.1 Females 14 years or younger	0	0	Risk Ratio (M‐H, Random, 95% CI)	Not estimable
3.9.2 Females 15‐25 years	1	17683	Risk Ratio (M‐H, Random, 95% CI)	0.55 [0.43, 0.70]
3.9.3 Females 25 years or older	0	0	Risk Ratio (M‐H, Random, 95% CI)	Not estimable
3.10 CIN2 irrespective of HPV type	1		Risk Ratio (M‐H, Random, 95% CI)	Subtotals only
3.10.1 Females 14 years or younger	0	0	Risk Ratio (M‐H, Random, 95% CI)	Not estimable
3.10.2 Females 15‐25 years	1	17160	Risk Ratio (M‐H, Random, 95% CI)	0.81 [0.70, 0.94]
3.10.3 Females 25 years or older	0	0	Risk Ratio (M‐H, Random, 95% CI)	Not estimable
3.11 CIN2 associated with HPV 6, 11, 16 and/or 18	1		Risk Ratio (M‐H, Random, 95% CI)	Subtotals only
3.11.1 Females 14 years or younger	0	0	Risk Ratio (M‐H, Random, 95% CI)	Not estimable
3.11.2 Females 15‐25 years	1	17683	Risk Ratio (M‐H, Random, 95% CI)	0.44 [0.34, 0.58]
3.11.3 Females 25 years or older	0	0	Risk Ratio (M‐H, Random, 95% CI)	Not estimable
3.12 Invasive vaginal or vulvar cancer	1		Risk Ratio (M‐H, Random, 95% CI)	Subtotals only
3.12.1 Females 14 years or younger	0	0	Risk Ratio (M‐H, Random, 95% CI)	Not estimable
3.12.2 Females 15‐25 years	1	5455	Risk Ratio (M‐H, Random, 95% CI)	3.01 [0.12, 73.85]
3.12.3 Females 25 years or older	0	0	Risk Ratio (M‐H, Random, 95% CI)	Not estimable
3.13 High‐grade VIN or VaIN irrespective of HPV type	1		Risk Ratio (M‐H, Random, 95% CI)	Subtotals only
3.13.1 Females 14 years or younger	0	0	Risk Ratio (M‐H, Random, 95% CI)	Not estimable
3.13.2 Females 15‐25 years	1	17391	Risk Ratio (M‐H, Random, 95% CI)	0.49 [0.32, 0.76]
3.13.3 Females 25 years or older	0	0	Risk Ratio (M‐H, Random, 95% CI)	Not estimable
3.14 High‐grade VIN or VaIN associated with HPV 6, 11, 16 and/or 18	2		Risk Ratio (M‐H, Random, 95% CI)	Subtotals only
3.14.1 Females 14 years or younger	0	0	Risk Ratio (M‐H, Random, 95% CI)	Not estimable
3.14.2 Females 15‐25 years	1	17925	Risk Ratio (M‐H, Random, 95% CI)	0.21 [0.10, 0.43]
3.14.3 Females 25 years or older	1	3382	Risk Ratio (M‐H, Random, 95% CI)	4.98 [0.24, 103.58]
3.15 Invasive anal cancer	1		Risk Ratio (M‐H, Random, 95% CI)	Subtotals only
3.15.1 Females 14 years or younger	0	0	Risk Ratio (M‐H, Random, 95% CI)	Not estimable
3.15.2 Females 15‐25 years	0	0	Risk Ratio (M‐H, Random, 95% CI)	Not estimable
3.15.3 Females 25 years or older	0	0	Risk Ratio (M‐H, Random, 95% CI)	Not estimable
3.15.4 Females and males	0	0	Risk Ratio (M‐H, Random, 95% CI)	Not estimable
3.15.5 Males 15‐25 years	1	551	Risk Ratio (M‐H, Random, 95% CI)	Not estimable
3.16 High‐grade AIN irrespective of HPV type	1		Risk Ratio (M‐H, Random, 95% CI)	Subtotals only
3.16.1 Females 14 years or younger	0	0	Risk Ratio (M‐H, Random, 95% CI)	Not estimable
3.16.2 Females 15‐25 years	0	0	Risk Ratio (M‐H, Random, 95% CI)	Not estimable
3.16.3 Females 25 years or older	0	0	Risk Ratio (M‐H, Random, 95% CI)	Not estimable
3.16.4 Females and males	0	0	Risk Ratio (M‐H, Random, 95% CI)	Not estimable
3.16.5 Males 15‐25 years	1	551	Risk Ratio (M‐H, Random, 95% CI)	0.75 [0.53, 1.07]
3.17 High‐grade AIN associated with HPV 6, 11, 16 and/or 18	1		Risk Ratio (M‐H, Random, 95% CI)	Subtotals only
3.17.1 Females 14 years or younger	0	0	Risk Ratio (M‐H, Random, 95% CI)	Not estimable
3.17.2 Females 15‐25 years	0	0	Risk Ratio (M‐H, Random, 95% CI)	Not estimable
3.17.3 Females 25 years or older	0	0	Risk Ratio (M‐H, Random, 95% CI)	Not estimable
3.17.4 Females and males	0	0	Risk Ratio (M‐H, Random, 95% CI)	Not estimable
3.17.5 Males 15‐25 years	1	551	Risk Ratio (M‐H, Random, 95% CI)	0.46 [0.27, 0.79]
3.18 Invasive penile cancer	1		Risk Ratio (M‐H, Random, 95% CI)	Subtotals only
3.18.1 Males 15‐25 years	1	3880	Risk Ratio (M‐H, Random, 95% CI)	Not estimable
3.19 High‐grade PeIN irrespective of HPV type	1		Risk Ratio (M‐H, Random, 95% CI)	Subtotals only
3.19.1 Males 15‐25 years	1	3880	Risk Ratio (M‐H, Random, 95% CI)	1.00 [0.20, 4.93]
3.20 High‐grade PeIN associated with HPV 6, 11, 16 and/or 18	1		Risk Ratio (M‐H, Random, 95% CI)	Subtotals only
3.20.1 Males 15‐25 years	1	3880	Risk Ratio (M‐H, Random, 95% CI)	1.00 [0.20, 4.93]
3.21 Serious adverse events	16		Risk Ratio (M‐H, Random, 95% CI)	Subtotals only
3.21.1 Females 14 years or younger	2	205	Risk Ratio (M‐H, Random, 95% CI)	1.57 [0.08, 31.59]
3.21.2 Females 15‐25 years	6	19467	Risk Ratio (M‐H, Random, 95% CI)	0.80 [0.61, 1.04]
3.21.3 Females 25 years or older	2	6775	Risk Ratio (M‐H, Random, 95% CI)	0.88 [0.61, 1.27]
3.21.4 Females and males	4	6524	Risk Ratio (M‐H, Random, 95% CI)	0.98 [0.47, 2.05]
3.21.5 Males 15‐25 years	2	5008	Risk Ratio (M‐H, Random, 95% CI)	0.69 [0.29, 1.65]
3.22 Treatment for HPV‐related pre‐invasive disease	2		Risk Ratio (M‐H, Random, 95% CI)	Subtotals only
3.22.1 Females 14 years or younger	0	0	Risk Ratio (M‐H, Random, 95% CI)	Not estimable
3.22.2 Females 15‐25 years	0	0	Risk Ratio (M‐H, Random, 95% CI)	Not estimable
3.22.3 Females 25 years or older	1	3768	Risk Ratio (M‐H, Random, 95% CI)	0.91 [0.74, 1.13]
3.22.4 Females and males	0	0	Risk Ratio (M‐H, Random, 95% CI)	Not estimable
3.22.5 Males 15‐25 years	1	3880	Risk Ratio (M‐H, Random, 95% CI)	0.59 [0.45, 0.78]
3.23 Anogenital warts irrespective of HPV type	2		Risk Ratio (M‐H, Random, 95% CI)	Subtotals only
3.23.1 Females 14 years or younger	0	0	Risk Ratio (M‐H, Random, 95% CI)	Not estimable
3.23.2 Females 15‐25 years	1	17391	Risk Ratio (M‐H, Random, 95% CI)	0.38 [0.31, 0.47]
3.23.3 Females 25 years or older	0	0	Risk Ratio (M‐H, Random, 95% CI)	Not estimable
3.23.4 Females and males	0	0	Risk Ratio (M‐H, Random, 95% CI)	Not estimable
3.23.5 Males 15‐25 years	1	3880	Risk Ratio (M‐H, Random, 95% CI)	0.39 [0.26, 0.58]
3.24 Anogenital warts associated with HPV 6, 11, 16 and/or 18	4		Risk Ratio (M‐H, Random, 95% CI)	Subtotals only
3.24.1 Females 14 years or younger	0	0	Risk Ratio (M‐H, Random, 95% CI)	Not estimable
3.24.2 Females 15‐25 years	2	17917	Risk Ratio (M‐H, Random, 95% CI)	0.21 [0.16, 0.27]
3.24.3 Females 25 years or older	1	3382	Risk Ratio (M‐H, Random, 95% CI)	0.58 [0.23, 1.47]
3.24.4 Females and males	0	0	Risk Ratio (M‐H, Random, 95% CI)	Not estimable
3.24.5 Males 15‐25 years	1	3880	Risk Ratio (M‐H, Random, 95% CI)	0.32 [0.20, 0.51]
3.25 12‐month persistent HPV 6, 11, 16 and/or 18 infection	2		Risk Ratio (M‐H, Random, 95% CI)	Subtotals only
3.25.1 Females 14 years or younger	0	0	Risk Ratio (M‐H, Random, 95% CI)	Not estimable
3.25.2 Females 15‐25 years	0	0	Risk Ratio (M‐H, Random, 95% CI)	Not estimable
3.25.3 Females 25 years or older	1	2959	Risk Ratio (M‐H, Random, 95% CI)	0.52 [0.38, 0.71]
3.25.4 Females and males	0	0	Risk Ratio (M‐H, Random, 95% CI)	Not estimable
3.25.5 Males 15‐25 years	1	2792	Risk Ratio (M‐H, Random, 95% CI)	0.20 [0.09, 0.45]
3.26 6‐month persistent HPV 6, 11, 16 and/or 18 infection	6		Risk Ratio (M‐H, Random, 95% CI)	Subtotals only
3.26.1 Females 14 years or younger	0	0	Risk Ratio (M‐H, Random, 95% CI)	Not estimable
3.26.2 Females 15‐25 years	2	1351	Risk Ratio (M‐H, Random, 95% CI)	0.09 [0.04, 0.19]
3.26.3 Females 25 years or older	2	6336	Risk Ratio (M‐H, Random, 95% CI)	0.62 [0.43, 0.90]
3.26.4 Females and males	0	0	Risk Ratio (M‐H, Random, 95% CI)	Not estimable
3.26.5 Males 15‐25 years	2	4692	Risk Ratio (M‐H, Random, 95% CI)	0.49 [0.41, 0.58]
3.27 Adverse pregnancy outcome: any adverse pregnancy outcome	1		Risk Ratio (M‐H, Random, 95% CI)	Subtotals only
3.27.1 Females 14 years or younger	0	0	Risk Ratio (M‐H, Random, 95% CI)	Not estimable
3.27.2 Females 15‐25 years	0	0	Risk Ratio (M‐H, Random, 95% CI)	Not estimable
3.27.3 Females 25 years or older	1	3006	Risk Ratio (M‐H, Random, 95% CI)	0.96 [0.82, 1.11]
3.28 Adverse pregnancy outcome: cervical incompetence	1		Risk Ratio (M‐H, Random, 95% CI)	Subtotals only
3.28.1 Females 14 years or younger	0	0	Risk Ratio (M‐H, Random, 95% CI)	Not estimable
3.28.2 Females 15‐25 years	1	12157	Risk Ratio (M‐H, Random, 95% CI)	0.20 [0.01, 4.16]
3.28.3 Females 25 years or older	0	0	Risk Ratio (M‐H, Random, 95% CI)	Not estimable
3.29 Adverse pregnancy outcome: infant/fetal abnormality	5		Risk Ratio (M‐H, Random, 95% CI)	Subtotals only
3.29.1 Females 14 years or younger	1	107	Risk Ratio (M‐H, Random, 95% CI)	0.94 [0.04, 22.38]
3.29.2 Females 15‐25 years	2	17599	Risk Ratio (M‐H, Random, 95% CI)	1.50 [0.59, 3.84]
3.29.3 Females 25 years or older	2	6823	Risk Ratio (M‐H, Random, 95% CI)	1.43 [0.72, 2.82]
3.30 Adverse pregnancy outcome: miscarriage	5		Risk Ratio (M‐H, Random, 95% CI)	Subtotals only
3.30.1 Females 14 years or younger	0	0	Risk Ratio (M‐H, Random, 95% CI)	Not estimable
3.30.2 Females 15‐25 years	3	18547	Risk Ratio (M‐H, Random, 95% CI)	0.93 [0.81, 1.08]
3.30.3 Females 25 years or older	2	6823	Risk Ratio (M‐H, Random, 95% CI)	0.98 [0.68, 1.41]
3.31 Adverse pregnancy outcome: pre‐term birth	3		Risk Ratio (M‐H, Random, 95% CI)	Subtotals only
3.31.1 Females 14 years or younger	0	0	Risk Ratio (M‐H, Random, 95% CI)	Not estimable
3.31.2 Females 15‐25 years	2	17502	Risk Ratio (M‐H, Random, 95% CI)	0.82 [0.24, 2.81]
3.31.3 Females 25 years or older	1	3778	Risk Ratio (M‐H, Random, 95% CI)	0.33 [0.01, 8.17]
3.32 Adverse pregnancy outcome: PROM	2		Risk Ratio (M‐H, Random, 95% CI)	Subtotals only
3.32.1 Females 14 years or younger	0	0	Risk Ratio (M‐H, Random, 95% CI)	Not estimable
3.32.2 Females 15‐25 years	2	17502	Risk Ratio (M‐H, Random, 95% CI)	2.08 [0.27, 16.08]
3.32.3 Females 25 years or older	0	0	Risk Ratio (M‐H, Random, 95% CI)	Not estimable
3.33 Adverse pregnancy outcome: stillbirth	4		Risk Ratio (M‐H, Random, 95% CI)	Subtotals only
3.33.1 Females 14 years or younger	0	0	Risk Ratio (M‐H, Random, 95% CI)	Not estimable
3.33.2 Females 15‐25 years	2	17599	Risk Ratio (M‐H, Random, 95% CI)	1.16 [0.49, 2.74]
3.33.3 Females 25 years or older	2	6823	Risk Ratio (M‐H, Random, 95% CI)	1.35 [0.17, 10.74]
3.34 Local adverse events: any	12		Risk Ratio (M‐H, Random, 95% CI)	Subtotals only
3.34.1 Females 14 years or younger	1	98	Risk Ratio (M‐H, Random, 95% CI)	1.44 [0.88, 2.37]
3.34.2 Females 15‐25 years	4	7841	Risk Ratio (M‐H, Random, 95% CI)	1.12 [1.09, 1.16]
3.34.3 Females 25 years or older	2	6775	Risk Ratio (M‐H, Random, 95% CI)	1.26 [1.11, 1.44]
3.34.4 Females and males	3	4014	Risk Ratio (M‐H, Random, 95% CI)	1.65 [1.41, 1.93]
3.34.5 Males 15‐25 years	2	5008	Risk Ratio (M‐H, Random, 95% CI)	1.11 [1.06, 1.16]
3.35 Local adverse events: pain	12		Risk Ratio (M‐H, Random, 95% CI)	Subtotals only
3.35.1 Females 14 years or younger	1	98	Risk Ratio (M‐H, Random, 95% CI)	1.42 [0.86, 2.33]
3.35.2 Females 15‐25 years	4	18733	Risk Ratio (M‐H, Random, 95% CI)	1.18 [1.11, 1.25]
3.35.3 Females 25 years or older	2	6775	Risk Ratio (M‐H, Random, 95% CI)	1.30 [1.10, 1.54]
3.35.4 Females and males	3	4832	Risk Ratio (M‐H, Random, 95% CI)	1.46 [1.18, 1.82]
3.35.5 Males 15‐25 years	2	5008	Risk Ratio (M‐H, Random, 95% CI)	1.13 [1.07, 1.19]
3.36 Local adverse events: redness	11		Risk Ratio (M‐H, Random, 95% CI)	Subtotals only
3.36.1 Females 14 years or younger	1	98	Risk Ratio (M‐H, Random, 95% CI)	1.92 [0.26, 14.47]
3.36.2 Females 15‐25 years	4	18733	Risk Ratio (M‐H, Random, 95% CI)	1.44 [1.23, 1.69]
3.36.3 Females 25 years or older	2	6775	Risk Ratio (M‐H, Random, 95% CI)	1.35 [1.18, 1.55]
3.36.4 Females and males	2	2349	Risk Ratio (M‐H, Random, 95% CI)	1.54 [1.22, 1.95]
3.36.5 Males 15‐25 years	2	5008	Risk Ratio (M‐H, Random, 95% CI)	1.12 [0.99, 1.26]
3.37 Local adverse events: swelling	12		Risk Ratio (M‐H, Random, 95% CI)	Subtotals only
3.37.1 Females 14 years or younger	1	98	Risk Ratio (M‐H, Random, 95% CI)	1.38 [0.54, 3.53]
3.37.2 Females 15‐25 years	4	18733	Risk Ratio (M‐H, Random, 95% CI)	1.76 [1.38, 2.26]
3.37.3 Females 25 years or older	2	6775	Risk Ratio (M‐H, Random, 95% CI)	1.79 [1.46, 2.20]
3.37.4 Females and males	3	4832	Risk Ratio (M‐H, Random, 95% CI)	2.20 [1.11, 4.32]
3.37.5 Males 15‐25 years	2	5008	Risk Ratio (M‐H, Random, 95% CI)	1.29 [1.04, 1.60]
3.38 Systemic adverse events: any	13	23913	Risk Ratio (M‐H, Random, 95% CI)	1.03 [0.98, 1.10]
3.38.1 Females 14 years or younger	1	98	Risk Ratio (M‐H, Random, 95% CI)	0.83 [0.53, 1.30]
3.38.2 Females 15‐25 years	5	8017	Risk Ratio (M‐H, Random, 95% CI)	1.02 [0.98, 1.05]
3.38.3 Females 25 years or older	2	6775	Risk Ratio (M‐H, Random, 95% CI)	1.00 [0.96, 1.05]
3.38.4 Females and males	3	4015	Risk Ratio (M‐H, Random, 95% CI)	1.15 [0.96, 1.38]
3.38.5 Males 15‐25 years	2	5008	Risk Ratio (M‐H, Random, 95% CI)	1.00 [0.92, 1.09]
3.39 Adverse events	11		Risk Ratio (M‐H, Random, 95% CI)	Subtotals only
3.39.1 Females 14 years or younger	1	98	Risk Ratio (M‐H, Random, 95% CI)	1.03 [0.79, 1.32]
3.39.2 Females 15‐25 years	3	1633	Risk Ratio (M‐H, Random, 95% CI)	1.07 [1.03, 1.12]
3.39.3 Females 25 years or older	2	6775	Risk Ratio (M‐H, Random, 95% CI)	1.07 [1.05, 1.10]
3.39.4 Females and males	3	4832	Risk Ratio (M‐H, Random, 95% CI)	1.17 [1.07, 1.27]
3.39.5 Males 15‐25 years	2	5008	Risk Ratio (M‐H, Random, 95% CI)	1.08 [1.04, 1.12]
3.40 Unsolicited adverse events	1		Risk Ratio (M‐H, Random, 95% CI)	Subtotals only
3.40.1 Females 14 years or younger	0	0	Risk Ratio (M‐H, Random, 95% CI)	Not estimable
3.40.2 Females 15‐25 years	0	0	Risk Ratio (M‐H, Random, 95% CI)	Not estimable
3.40.3 Females 25 years or older	0	0	Risk Ratio (M‐H, Random, 95% CI)	Not estimable
3.40.4 Females and males	1	1692	Risk Ratio (M‐H, Random, 95% CI)	1.01 [0.86, 1.19]
3.40.5 Males 15‐25 years	0	0	Risk Ratio (M‐H, Random, 95% CI)	Not estimable
3.41 Adverse events that led to discontinuation	11		Risk Ratio (M‐H, Random, 95% CI)	Subtotals only
3.41.1 Females 14 years or younger	1	98	Risk Ratio (M‐H, Random, 95% CI)	Not estimable
3.41.2 Females 15‐25 years	3	1777	Risk Ratio (M‐H, Random, 95% CI)	0.94 [0.10, 9.01]
3.41.3 Females 25 years or older	2	6775	Risk Ratio (M‐H, Random, 95% CI)	1.62 [0.32, 8.20]
3.41.4 Females and males	3	4775	Risk Ratio (M‐H, Random, 95% CI)	0.72 [0.32, 1.63]
3.41.5 Males 15‐25 years	2	5008	Risk Ratio (M‐H, Random, 95% CI)	0.33 [0.12, 0.85]
3.42 Specific adverse events: sexual behaviour: chlamydia infection (genital)	2		Risk Ratio (M‐H, Random, 95% CI)	Subtotals only
3.42.1 Females 14 years or younger	0	0	Risk Ratio (M‐H, Random, 95% CI)	Not estimable
3.42.2 Females 15‐25 years	0	0	Risk Ratio (M‐H, Random, 95% CI)	Not estimable
3.42.3 Females 25 years or older	1	2997	Risk Ratio (M‐H, Random, 95% CI)	0.87 [0.41, 1.81]
3.42.4 Females and males	0	0	Risk Ratio (M‐H, Random, 95% CI)	Not estimable
3.42.5 Males 15‐25 years	1	3637	Risk Ratio (M‐H, Random, 95% CI)	2.00 [0.37, 10.92]
3.43 Specific adverse events: sexual behaviour: chlamydia infection (anal)	1		Risk Ratio (M‐H, Random, 95% CI)	Subtotals only
3.43.1 Females 14 years or younger	0	0	Risk Ratio (M‐H, Random, 95% CI)	Not estimable
3.43.2 Females 15‐25 years	0	0	Risk Ratio (M‐H, Random, 95% CI)	Not estimable
3.43.3 Females 25 years or older	0	0	Risk Ratio (M‐H, Random, 95% CI)	Not estimable
3.43.4 Females and males	0	0	Risk Ratio (M‐H, Random, 95% CI)	Not estimable
3.43.5 Males 15‐25 years	1	3637	Risk Ratio (M‐H, Random, 95% CI)	1.00 [0.55, 1.83]
3.44 All‐cause mortality	10		Risk Ratio (M‐H, Random, 95% CI)	Subtotals only
3.44.1 Females 14 years or younger	1	98	Risk Ratio (M‐H, Random, 95% CI)	Not estimable
3.44.2 Females 15‐25 years	3	18450	Risk Ratio (M‐H, Random, 95% CI)	1.29 [0.48, 3.46]
3.44.3 Females 25 years or older	2	6775	Risk Ratio (M‐H, Random, 95% CI)	6.27 [1.12, 35.17]
3.44.4 Females and males	2	4175	Risk Ratio (M‐H, Random, 95% CI)	Not estimable
3.44.5 Males 15‐25 years	2	5008	Risk Ratio (M‐H, Random, 95% CI)	0.31 [0.09, 1.01]

**Comparison 4:** Gardasil‐9 versus control

Outcome or subgroup title	No. of studies	No. of participants	Statistical method	Effect size
4.1 Serious adverse events	1		Risk Ratio (M‐H, Random, 95% CI)	Subtotals only
4.1.1 Females 14 years or younger	0	0	Risk Ratio (M‐H, Random, 95% CI)	Not estimable
4.1.2 Females 15‐25 years	1	1515	Risk Ratio (M‐H, Random, 95% CI)	0.87 [0.56, 1.36]
4.1.3 Females 25 years or older	0	0	Risk Ratio (M‐H, Random, 95% CI)	Not estimable
4.1.4 Females and males	0	0	Risk Ratio (M‐H, Random, 95% CI)	Not estimable
4.1.5 Males 15‐25 years	0	0	Risk Ratio (M‐H, Random, 95% CI)	Not estimable
4.2 6‐month persistent HPV 16, 18, 31, 33, 45, 52 and/or 58 infection	1		Risk Ratio (M‐H, Random, 95% CI)	Subtotals only
4.2.1 Females 14 years or younger	0	0	Risk Ratio (M‐H, Random, 95% CI)	Not estimable
4.2.2 Females 15‐25 years	1	1515	Risk Ratio (M‐H, Random, 95% CI)	0.53 [0.42, 0.66]
4.2.3 Females 25 years or older	0	0	Risk Ratio (M‐H, Random, 95% CI)	Not estimable
4.2.4 Females and males	0	0	Risk Ratio (M‐H, Random, 95% CI)	Not estimable
4.2.5 Males 15‐25 years	0	0	Risk Ratio (M‐H, Random, 95% CI)	Not estimable
4.3 Specific adverse events: sexual behaviour: chlamydia infection	1		Risk Ratio (M‐H, Random, 95% CI)	Subtotals only
4.3.1 Females 14 years or younger	0	0	Risk Ratio (M‐H, Random, 95% CI)	Not estimable
4.3.2 Females 15‐25 years	1	969	Risk Ratio (M‐H, Random, 95% CI)	1.06 [0.82, 1.37]
4.3.3 Females 25 years or older	0	0	Risk Ratio (M‐H, Random, 95% CI)	Not estimable
4.3.4 Females and males	0	0	Risk Ratio (M‐H, Random, 95% CI)	Not estimable
4.3.5 Males 15‐25 years	0	0	Risk Ratio (M‐H, Random, 95% CI)	Not estimable
4.4 Specific adverse events: sexual behaviour: gonorrhoea infection	1		Risk Ratio (M‐H, Random, 95% CI)	Subtotals only
4.4.1 Females 14 years or younger	0	0	Risk Ratio (M‐H, Random, 95% CI)	Not estimable
4.4.2 Females 15‐25 years	1	969	Risk Ratio (M‐H, Random, 95% CI)	1.14 [0.72, 1.80]
4.4.3 Females 25 years or older	0	0	Risk Ratio (M‐H, Random, 95% CI)	Not estimable
4.4.4 Females and males	0	0	Risk Ratio (M‐H, Random, 95% CI)	Not estimable
4.4.5 Males 15‐25 years	0	0	Risk Ratio (M‐H, Random, 95% CI)	Not estimable

**Comparison 5:** Cecolin versus control

Outcome or subgroup title	No. of studies	No. of participants	Statistical method	Effect size
5.1 Serious adverse events	2		Risk Ratio (M‐H, Random, 95% CI)	Subtotals only
5.1.1 Females 14 years or younger	0	0	Risk Ratio (M‐H, Random, 95% CI)	Not estimable
5.1.2 Females 15‐25 years	1	1594	Risk Ratio (M‐H, Random, 95% CI)	1.22 [0.34, 4.34]
5.1.3 Females 25 years or older	1	7072	Risk Ratio (M‐H, Random, 95% CI)	0.99 [0.83, 1.19]
5.1.4 Females and males	0	0	Risk Ratio (M‐H, Random, 95% CI)	Not estimable
5.1.5 Males 15‐25 years	0	0	Risk Ratio (M‐H, Random, 95% CI)	Not estimable
5.2 6‐month persistent HPV 16 and/or 18 infection	1		Risk Ratio (M‐H, Random, 95% CI)	Subtotals only
5.2.1 Females 14 years or younger	0	0	Risk Ratio (M‐H, Random, 95% CI)	Not estimable
5.2.2 Females 15‐25 years	0	0	Risk Ratio (M‐H, Random, 95% CI)	Not estimable
5.2.3 Females 25 years or older	1	7042	Risk Ratio (M‐H, Random, 95% CI)	0.22 [0.13, 0.39]
5.2.4 Females and males	0	0	Risk Ratio (M‐H, Random, 95% CI)	Not estimable
5.2.5 Males 15‐25 years	0	0	Risk Ratio (M‐H, Random, 95% CI)	Not estimable
5.3 Incident HPV 16 and/or 18 infection	1		Risk Ratio (M‐H, Random, 95% CI)	Subtotals only
5.3.1 Females 14 years or younger	0	0	Risk Ratio (M‐H, Random, 95% CI)	Not estimable
5.3.2 Females 15‐25 years	0	0	Risk Ratio (M‐H, Random, 95% CI)	Not estimable
5.3.3 Females 25 years or older	1	6779	Risk Ratio (M‐H, Random, 95% CI)	0.29 [0.21, 0.41]
5.3.4 Females and males	0	0	Risk Ratio (M‐H, Random, 95% CI)	Not estimable
5.3.5 Males 15‐25 years	0	0	Risk Ratio (M‐H, Random, 95% CI)	Not estimable
5.4 Adverse pregnancy outcome: infant/fetal abnormality	1		Risk Ratio (M‐H, Random, 95% CI)	Subtotals only
5.4.1 Females 14 years or younger	0	0	Risk Ratio (M‐H, Random, 95% CI)	Not estimable
5.4.2 Females 15‐25 years	0	0	Risk Ratio (M‐H, Random, 95% CI)	Not estimable
5.4.3 Females 25 years or older	1	7072	Risk Ratio (M‐H, Random, 95% CI)	0.22 [0.01, 4.52]
5.5 Adverse pregnancy outcome: miscarriage	1		Risk Ratio (M‐H, Random, 95% CI)	Subtotals only
5.5.1 Females 14 years or younger	0	0	Risk Ratio (M‐H, Random, 95% CI)	Not estimable
5.5.2 Females 15‐25 years	0	0	Risk Ratio (M‐H, Random, 95% CI)	Not estimable
5.5.3 Females 25 years or older	1	7072	Risk Ratio (M‐H, Random, 95% CI)	1.09 [0.78, 1.51]
5.6 Adverse pregnancy outcome: stillbirth	1		Risk Ratio (M‐H, Random, 95% CI)	Subtotals only
5.6.1 Females 14 years or younger	0	0	Risk Ratio (M‐H, Random, 95% CI)	Not estimable
5.6.2 Females 15‐25 years	0	0	Risk Ratio (M‐H, Random, 95% CI)	Not estimable
5.6.3 Females 25 years or older	1	7072	Risk Ratio (M‐H, Random, 95% CI)	0.78 [0.34, 1.74]
5.7 Local adverse events: any	2		Risk Ratio (M‐H, Random, 95% CI)	Subtotals only
5.7.1 Females 14 years or younger	0	0	Risk Ratio (M‐H, Random, 95% CI)	Not estimable
5.7.2 Females 15‐25 years	1	1594	Risk Ratio (M‐H, Random, 95% CI)	1.21 [0.97, 1.52]
5.7.3 Females 25 years or older	1	7072	Risk Ratio (M‐H, Random, 95% CI)	0.98 [0.92, 1.03]
5.7.4 Females and males	0	0	Risk Ratio (M‐H, Random, 95% CI)	Not estimable
5.7.5 Males 15‐25 years	0	0	Risk Ratio (M‐H, Random, 95% CI)	Not estimable
5.8 Local adverse events: pain	2		Risk Ratio (M‐H, Random, 95% CI)	Subtotals only
5.8.1 Females 14 years or younger	0	0	Risk Ratio (M‐H, Random, 95% CI)	Not estimable
5.8.2 Females 15‐25 years	1	1594	Risk Ratio (M‐H, Random, 95% CI)	1.27 [0.99, 1.63]
5.8.3 Females 25 years or older	1	7072	Risk Ratio (M‐H, Random, 95% CI)	1.03 [0.97, 1.09]
5.8.4 Females and males	0	0	Risk Ratio (M‐H, Random, 95% CI)	Not estimable
5.8.5 Males 15‐25 years	0	0	Risk Ratio (M‐H, Random, 95% CI)	Not estimable
5.9 Local adverse events: redness	2		Risk Ratio (M‐H, Random, 95% CI)	Subtotals only
5.9.1 Females 14 years or younger	0	0	Risk Ratio (M‐H, Random, 95% CI)	Not estimable
5.9.2 Females 15‐25 years	1	1594	Risk Ratio (M‐H, Random, 95% CI)	1.29 [0.65, 2.57]
5.9.3 Females 25 years or older	1	7072	Risk Ratio (M‐H, Random, 95% CI)	0.72 [0.59, 0.87]
5.9.4 Females and males	0	0	Risk Ratio (M‐H, Random, 95% CI)	Not estimable
5.9.5 Males 15‐25 years	0	0	Risk Ratio (M‐H, Random, 95% CI)	Not estimable
5.10 Local adverse events: swelling	2		Risk Ratio (M‐H, Random, 95% CI)	Subtotals only
5.10.1 Females 14 years or younger	0	0	Risk Ratio (M‐H, Random, 95% CI)	Not estimable
5.10.2 Females 15‐25 years	1	1594	Risk Ratio (M‐H, Random, 95% CI)	0.99 [0.43, 2.32]
5.10.3 Females 25 years or older	1	7072	Risk Ratio (M‐H, Random, 95% CI)	0.62 [0.51, 0.74]
5.10.4 Females and males	0	0	Risk Ratio (M‐H, Random, 95% CI)	Not estimable
5.10.5 Males 15‐25 years	0	0	Risk Ratio (M‐H, Random, 95% CI)	Not estimable
5.11 Systemic adverse events: any	2		Risk Ratio (M‐H, Random, 95% CI)	Subtotals only
5.11.1 Females 14 years or younger	0	0	Risk Ratio (M‐H, Random, 95% CI)	Not estimable
5.11.2 Females 15‐25 years	1	1594	Risk Ratio (M‐H, Random, 95% CI)	1.02 [0.90, 1.15]
5.11.3 Females 25 years or older	1	7072	Risk Ratio (M‐H, Random, 95% CI)	1.07 [1.02, 1.13]
5.11.4 Females and males	0	0	Risk Ratio (M‐H, Random, 95% CI)	Not estimable
5.11.5 Males 15‐25 years	0	0	Risk Ratio (M‐H, Random, 95% CI)	Not estimable
5.12 Unsolicited adverse events	2		Risk Ratio (M‐H, Random, 95% CI)	Subtotals only
5.12.1 Females 14 years or younger	0	0	Risk Ratio (M‐H, Random, 95% CI)	Not estimable
5.12.2 Females 15‐25 years	1	1594	Risk Ratio (M‐H, Random, 95% CI)	1.00 [0.86, 1.17]
5.12.3 Females 25 years or older	1	7072	Risk Ratio (M‐H, Random, 95% CI)	1.10 [1.04, 1.17]
5.12.4 Females and males	0	0	Risk Ratio (M‐H, Random, 95% CI)	Not estimable
5.12.5 Males 15‐25 years	0	0	Risk Ratio (M‐H, Random, 95% CI)	Not estimable
5.13 All‐cause mortality	2		Risk Ratio (M‐H, Random, 95% CI)	Subtotals only
5.13.1 Females 14 years or younger	0	0	Risk Ratio (M‐H, Random, 95% CI)	Not estimable
5.13.2 Females 15‐25 years	1	1594	Risk Ratio (M‐H, Random, 95% CI)	Not estimable
5.13.3 Females 25 years or older	1	7072	Risk Ratio (M‐H, Random, 95% CI)	1.09 [0.22, 5.37]
5.13.4 Females and males	0	0	Risk Ratio (M‐H, Random, 95% CI)	Not estimable
5.13.5 Males 15‐25 years	0	0	Risk Ratio (M‐H, Random, 95% CI)	Not estimable

**Comparison 6:** Gardasil versus Cervarix

Outcome or subgroup title	No. of studies	No. of participants	Statistical method	Effect size
6.1 Serious adverse events	4		Risk Ratio (M‐H, Random, 95% CI)	Subtotals only
6.1.1 Females 14 years or younger	2	1273	Risk Ratio (M‐H, Random, 95% CI)	0.59 [0.34, 1.02]
6.1.2 Females 15‐25 years	1	62	Risk Ratio (M‐H, Random, 95% CI)	Not estimable
6.1.3 Females 25 years or older	1	1106	Risk Ratio (M‐H, Random, 95% CI)	0.84 [0.55, 1.28]
6.1.4 Females and males	0	0	Risk Ratio (M‐H, Random, 95% CI)	Not estimable
6.1.5 Males 15‐25 years	0	0	Risk Ratio (M‐H, Random, 95% CI)	Not estimable
6.2 Adverse pregnancy outcome: infant/fetal abnormality	1		Risk Ratio (M‐H, Random, 95% CI)	Subtotals only
6.2.1 Females 14 years or younger	0	0	Risk Ratio (M‐H, Random, 95% CI)	Not estimable
6.2.2 Females 15‐25 years	0	0	Risk Ratio (M‐H, Random, 95% CI)	Not estimable
6.2.3 Females 25 years or older	1	1106	Risk Ratio (M‐H, Random, 95% CI)	5.00 [0.24, 103.91]
6.3 Adverse pregnancy outcome: miscarriage	1		Risk Ratio (M‐H, Random, 95% CI)	Subtotals only
6.3.1 Females 14 years or younger	0	0	Risk Ratio (M‐H, Random, 95% CI)	Not estimable
6.3.2 Females 15‐25 years	0	0	Risk Ratio (M‐H, Random, 95% CI)	Not estimable
6.3.3 Females 25 years or older	1	1106	Risk Ratio (M‐H, Random, 95% CI)	0.73 [0.34, 1.58]
6.4 Adverse pregnancy outcome: pre‐term birth	1		Risk Ratio (M‐H, Random, 95% CI)	Subtotals only
6.4.1 Females 14 years or younger	0	0	Risk Ratio (M‐H, Random, 95% CI)	Not estimable
6.4.2 Females 15‐25 years	0	0	Risk Ratio (M‐H, Random, 95% CI)	Not estimable
6.4.3 Females 25 years or older	1	1106	Risk Ratio (M‐H, Random, 95% CI)	1.00 [0.06, 15.95]
6.5 Adverse pregnancy outcome: stillbirth	1		Risk Ratio (M‐H, Random, 95% CI)	Subtotals only
6.5.1 Females 14 years or younger	0	0	Risk Ratio (M‐H, Random, 95% CI)	Not estimable
6.5.2 Females 15‐25 years	0	0	Risk Ratio (M‐H, Random, 95% CI)	Not estimable
6.5.3 Females 25 years or older	1	1106	Risk Ratio (M‐H, Random, 95% CI)	0.33 [0.01, 8.16]
6.6 Local adverse events: pain	5		Risk Ratio (M‐H, Random, 95% CI)	Subtotals only
6.6.1 Females 14 years or younger	2	1270	Risk Ratio (M‐H, Random, 95% CI)	0.88 [0.85, 0.92]
6.6.2 Females 15‐25 years	2	73	Risk Ratio (M‐H, Random, 95% CI)	0.53 [0.22, 1.28]
6.6.3 Females 25 years or older	1	1048	Risk Ratio (M‐H, Random, 95% CI)	0.77 [0.73, 0.82]
6.6.4 Females and males	0	0	Risk Ratio (M‐H, Random, 95% CI)	Not estimable
6.6.5 Males 15‐25 years	0	0	Risk Ratio (M‐H, Random, 95% CI)	Not estimable
6.7 Local adverse events: redness	5		Risk Ratio (M‐H, Random, 95% CI)	Subtotals only
6.7.1 Females 14 years or younger	2	1270	Risk Ratio (M‐H, Random, 95% CI)	0.78 [0.69, 0.89]
6.7.2 Females 15‐25 years	2	73	Risk Ratio (M‐H, Random, 95% CI)	2.77 [0.31, 24.48]
6.7.3 Females 25 years or older	1	1048	Risk Ratio (M‐H, Random, 95% CI)	0.58 [0.48, 0.69]
6.7.4 Females and males	0	0	Risk Ratio (M‐H, Random, 95% CI)	Not estimable
6.7.5 Males 15‐25 years	0	0	Risk Ratio (M‐H, Random, 95% CI)	Not estimable
6.8 Local adverse events: swelling	5		Risk Ratio (M‐H, Random, 95% CI)	Subtotals only
6.8.1 Females 14 years or younger	2	1270	Risk Ratio (M‐H, Random, 95% CI)	0.75 [0.53, 1.07]
6.8.2 Females 15‐25 years	2	73	Risk Ratio (M‐H, Random, 95% CI)	0.20 [0.01, 4.00]
6.8.3 Females 25 years or older	1	1048	Risk Ratio (M‐H, Random, 95% CI)	0.60 [0.49, 0.73]
6.8.4 Females and males	0	0	Risk Ratio (M‐H, Random, 95% CI)	Not estimable
6.8.5 Males 15‐25 years	0	0	Risk Ratio (M‐H, Random, 95% CI)	Not estimable
6.9 Adverse events	3		Risk Ratio (M‐H, Random, 95% CI)	Subtotals only
6.9.1 Females 14 years or younger	1	1072	Risk Ratio (M‐H, Random, 95% CI)	0.95 [0.92, 0.98]
6.9.2 Females 15‐25 years	1	62	Risk Ratio (M‐H, Random, 95% CI)	0.38 [0.17, 0.83]
6.9.3 Females 25 years or older	1	1051	Risk Ratio (M‐H, Random, 95% CI)	0.91 [0.88, 0.95]
6.9.4 Females and males	0	0	Risk Ratio (M‐H, Random, 95% CI)	Not estimable
6.9.5 Males 15‐25 years	0	0	Risk Ratio (M‐H, Random, 95% CI)	Not estimable
6.10 Unsolicited adverse events	2		Risk Ratio (M‐H, Random, 95% CI)	Subtotals only
6.10.1 Females 14 years or younger	1	1075	Risk Ratio (M‐H, Random, 95% CI)	1.09 [0.88, 1.34]
6.10.2 Females 15‐25 years	0	0	Risk Ratio (M‐H, Random, 95% CI)	Not estimable
6.10.3 Females 25 years or older	1	1106	Risk Ratio (M‐H, Random, 95% CI)	0.86 [0.74, 1.00]
6.10.4 Females and males	0	0	Risk Ratio (M‐H, Random, 95% CI)	Not estimable
6.10.5 Males 15‐25 years	0	0	Risk Ratio (M‐H, Random, 95% CI)	Not estimable
6.11 Adverse events that led to discontinuation	2		Risk Ratio (M‐H, Random, 95% CI)	Subtotals only
6.11.1 Females 14 years or younger	1	1075	Risk Ratio (M‐H, Random, 95% CI)	1.51 [0.06, 36.88]
6.11.2 Females 15‐25 years	0	0	Risk Ratio (M‐H, Random, 95% CI)	Not estimable
6.11.3 Females 25 years or older	1	1106	Risk Ratio (M‐H, Random, 95% CI)	0.60 [0.14, 2.50]
6.11.4 Females and males	0	0	Risk Ratio (M‐H, Random, 95% CI)	Not estimable
6.11.5 Males 15‐25 years	0	0	Risk Ratio (M‐H, Random, 95% CI)	Not estimable
6.12 Specific adverse events: POTS	2		Risk Ratio (M‐H, Random, 95% CI)	Subtotals only
6.12.1 Females 14 years or younger	1	1075	Risk Ratio (M‐H, Random, 95% CI)	0.17 [0.01, 4.10]
6.12.2 Females 15‐25 years	0	0	Risk Ratio (M‐H, Random, 95% CI)	Not estimable
6.12.3 Females 25 years or older	1	1106	Risk Ratio (M‐H, Random, 95% CI)	3.00 [0.12, 73.48]
6.12.4 Females and males	0	0	Risk Ratio (M‐H, Random, 95% CI)	Not estimable
6.12.5 Males 15‐25 years	0	0	Risk Ratio (M‐H, Random, 95% CI)	Not estimable
6.13 Specific adverse events: paralysis	1		Risk Ratio (M‐H, Random, 95% CI)	Subtotals only
6.13.1 Females 14 years or younger	0	0	Risk Ratio (M‐H, Random, 95% CI)	Not estimable
6.13.2 Females 15‐25 years	0	0	Risk Ratio (M‐H, Random, 95% CI)	Not estimable
6.13.3 Females 25 years or older	1	1106	Risk Ratio (M‐H, Random, 95% CI)	0.33 [0.01, 8.16]
6.13.4 Females and males	0	0	Risk Ratio (M‐H, Random, 95% CI)	Not estimable
6.13.5 Males 15‐25 years	0	0	Risk Ratio (M‐H, Random, 95% CI)	Not estimable
6.14 Specific adverse events: infertility	1		Risk Ratio (M‐H, Random, 95% CI)	Subtotals only
6.14.1 Females 14 years or younger	0	0	Risk Ratio (M‐H, Random, 95% CI)	Not estimable
6.14.2 Females 15‐25 years	0	0	Risk Ratio (M‐H, Random, 95% CI)	Not estimable
6.14.3 Females 25 years or older	1	1106	Risk Ratio (M‐H, Random, 95% CI)	3.00 [0.12, 73.48]
6.14.4 Females and males	0	0	Risk Ratio (M‐H, Random, 95% CI)	Not estimable
6.14.5 Males 15‐25 years	0	0	Risk Ratio (M‐H, Random, 95% CI)	Not estimable
6.15 Specific adverse events: sexual behaviour: chlamydia infection	1		Risk Ratio (M‐H, Random, 95% CI)	Subtotals only
6.15.1 Females 14 years or younger	0	0	Risk Ratio (M‐H, Random, 95% CI)	Not estimable
6.15.2 Females 15‐25 years	0	0	Risk Ratio (M‐H, Random, 95% CI)	Not estimable
6.15.3 Females 25 years or older	1	1106	Risk Ratio (M‐H, Random, 95% CI)	3.00 [0.31, 28.75]
6.15.4 Females and males	0	0	Risk Ratio (M‐H, Random, 95% CI)	Not estimable
6.15.5 Males 15‐25 years	0	0	Risk Ratio (M‐H, Random, 95% CI)	Not estimable
6.16 Specific adverse events: sexual behaviour: genital herpes infection	1	1106	Risk Ratio (M‐H, Random, 95% CI)	0.50 [0.05, 5.50]
6.16.1 Females 14 years or younger	0	0	Risk Ratio (M‐H, Random, 95% CI)	Not estimable
6.16.2 Females 15‐25 years	0	0	Risk Ratio (M‐H, Random, 95% CI)	Not estimable
6.16.3 Females 25 years or older	1	1106	Risk Ratio (M‐H, Random, 95% CI)	0.50 [0.05, 5.50]
6.16.4 Females and males	0	0	Risk Ratio (M‐H, Random, 95% CI)	Not estimable
6.16.5 Males 15‐25 years	0	0	Risk Ratio (M‐H, Random, 95% CI)	Not estimable
6.17 All‐cause mortality	2		Risk Ratio (M‐H, Random, 95% CI)	Subtotals only
6.17.1 Females 14 years or younger	1	1075	Risk Ratio (M‐H, Random, 95% CI)	1.51 [0.06, 36.88]
6.17.2 Females 15‐25 years	0	0	Risk Ratio (M‐H, Random, 95% CI)	Not estimable
6.17.3 Females 25 years or older	1	1106	Risk Ratio (M‐H, Random, 95% CI)	0.33 [0.01, 8.16]
6.17.4 Females and males	0	0	Risk Ratio (M‐H, Random, 95% CI)	Not estimable
6.17.5 Males 15‐25 years	0	0	Risk Ratio (M‐H, Random, 95% CI)	Not estimable

**Comparison 7:** Gardasil‐9 versus Cervarix

Outcome or subgroup title	No. of studies	No. of participants	Statistical method	Effect size
7.1 Serious adverse events	2		Risk Ratio (M‐H, Random, 95% CI)	Subtotals only
7.1.1 Females 14 years or younger	1	930	Risk Ratio (M‐H, Random, 95% CI)	1.33 [0.73, 2.42]
7.1.2 Females 15‐25 years	1	1518	Risk Ratio (M‐H, Random, 95% CI)	0.87 [0.56, 1.37]
7.1.3 Females 25 years or older	0	0	Risk Ratio (M‐H, Random, 95% CI)	Not estimable
7.1.4 Females and males	0	0	Risk Ratio (M‐H, Random, 95% CI)	Not estimable
7.1.5 Males 15‐25 years	0	0	Risk Ratio (M‐H, Random, 95% CI)	Not estimable
7.2 6‐month persistent HPV 16 and/or 18 infection	1		Risk Ratio (M‐H, Random, 95% CI)	Subtotals only
7.2.1 Females 14 years or younger	0	0	Risk Ratio (M‐H, Random, 95% CI)	Not estimable
7.2.2 Females 15‐25 years	1	1518	Risk Ratio (M‐H, Random, 95% CI)	1.12 [0.71, 1.76]
7.2.3 Females 25 years or older	0	0	Risk Ratio (M‐H, Random, 95% CI)	Not estimable
7.2.4 Females and males	0	0	Risk Ratio (M‐H, Random, 95% CI)	Not estimable
7.2.5 Males 15‐25 years	0	0	Risk Ratio (M‐H, Random, 95% CI)	Not estimable
7.3 6‐month persistent HPV 16, 18, 31, 33, 45, 52 and/or 58 infection	1		Risk Ratio (M‐H, Random, 95% CI)	Subtotals only
7.3.1 Females 14 years or younger	0	0	Risk Ratio (M‐H, Random, 95% CI)	Not estimable
7.3.2 Females 15‐25 years	1	1518	Risk Ratio (M‐H, Random, 95% CI)	0.58 [0.47, 0.73]
7.3.3 Females 25 years or older	0	0	Risk Ratio (M‐H, Random, 95% CI)	Not estimable
7.3.4 Females and males	0	0	Risk Ratio (M‐H, Random, 95% CI)	Not estimable
7.3.5 Males 15‐25 years	0	0	Risk Ratio (M‐H, Random, 95% CI)	Not estimable
7.4 Adverse pregnancy outcome: any adverse pregnancy outcome	1		Risk Ratio (M‐H, Random, 95% CI)	Subtotals only
7.4.1 Females 14 years or younger	0	0	Risk Ratio (M‐H, Random, 95% CI)	Not estimable
7.4.2 Females 15‐25 years	1	1518	Risk Ratio (M‐H, Random, 95% CI)	1.27 [0.70, 2.29]
7.4.3 Females 25 years or older	0	0	Risk Ratio (M‐H, Random, 95% CI)	Not estimable
7.5 Adverse pregnancy outcome: infant/fetal abnormality	1		Risk Ratio (M‐H, Random, 95% CI)	Subtotals only
7.5.1 Females 14 years or younger	1	930	Risk Ratio (M‐H, Random, 95% CI)	0.33 [0.01, 8.16]
7.5.2 Females 15‐25 years	0	0	Risk Ratio (M‐H, Random, 95% CI)	Not estimable
7.5.3 Females 25 years or older	0	0	Risk Ratio (M‐H, Random, 95% CI)	Not estimable
7.6 Adverse pregnancy outcome: miscarriage	1		Risk Ratio (M‐H, Random, 95% CI)	Subtotals only
7.6.1 Females 14 years or younger	1	920	Risk Ratio (M‐H, Random, 95% CI)	2.99 [0.12, 73.13]
7.6.2 Females 15‐25 years	0	0	Risk Ratio (M‐H, Random, 95% CI)	Not estimable
7.6.3 Females 25 years or older	0	0	Risk Ratio (M‐H, Random, 95% CI)	Not estimable
7.7 Specific adverse events: sexual behaviour: chlamydia infection	1		Risk Ratio (M‐H, Random, 95% CI)	Subtotals only
7.7.1 Females 14 years or younger	0	0	Risk Ratio (M‐H, Random, 95% CI)	Not estimable
7.7.2 Females 15‐25 years	1	985	Risk Ratio (M‐H, Random, 95% CI)	0.86 [0.68, 1.10]
7.7.3 Females 25 years or older	0	0	Risk Ratio (M‐H, Random, 95% CI)	Not estimable
7.7.4 Females and males	0	0	Risk Ratio (M‐H, Random, 95% CI)	Not estimable
7.7.5 Males 15‐25 years	0	0	Risk Ratio (M‐H, Random, 95% CI)	Not estimable
7.8 Specific adverse events: sexual behaviour: gonorrhoea infection	1		Risk Ratio (M‐H, Random, 95% CI)	Subtotals only
7.8.1 Females 14 years or younger	0	0	Risk Ratio (M‐H, Random, 95% CI)	Not estimable
7.8.2 Females 15‐25 years	1	985	Risk Ratio (M‐H, Random, 95% CI)	1.11 [0.70, 1.74]
7.8.3 Females 25 years or older	0	0	Risk Ratio (M‐H, Random, 95% CI)	Not estimable
7.8.4 Females and males	0	0	Risk Ratio (M‐H, Random, 95% CI)	Not estimable
7.8.5 Males 15‐25 years	0	0	Risk Ratio (M‐H, Random, 95% CI)	Not estimable
7.9 All‐cause mortality	1		Risk Ratio (M‐H, Random, 95% CI)	Subtotals only
7.9.1 Females 14 years or younger	1	930	Risk Ratio (M‐H, Random, 95% CI)	3.00 [0.12, 73.45]
7.9.2 Females 15‐25 years	0	0	Risk Ratio (M‐H, Random, 95% CI)	Not estimable
7.9.3 Females 25 years or older	0	0	Risk Ratio (M‐H, Random, 95% CI)	Not estimable
7.9.4 Females and males	0	0	Risk Ratio (M‐H, Random, 95% CI)	Not estimable
7.9.5 Males 15‐25 years	0	0	Risk Ratio (M‐H, Random, 95% CI)	Not estimable

**Comparison 8:** Gardasil‐9 versus Gardasil

Outcome or subgroup title	No. of studies	No. of participants	Statistical method	Effect size
8.1 CIN3+ associated with HPV 6, 11, 16 and/or 18	1		Risk Ratio (M‐H, Random, 95% CI)	Subtotals only
8.1.1 Females 14 years or younger	0	0	Risk Ratio (M‐H, Random, 95% CI)	Not estimable
8.1.2 Females 15‐25 years	1	11656	Risk Ratio (M‐H, Random, 95% CI)	0.33 [0.01, 8.19]
8.1.3 Females 25 years or older	0	0	Risk Ratio (M‐H, Random, 95% CI)	Not estimable
8.2 CIN3+ associated with HPV 31, 33, 45, 52 and/or 58	1		Risk Ratio (M‐H, Random, 95% CI)	Subtotals only
8.2.1 Females 14 years or younger	0	0	Risk Ratio (M‐H, Random, 95% CI)	Not estimable
8.2.2 Females 15‐25 years	1	11892	Risk Ratio (M‐H, Random, 95% CI)	0.07 [0.00, 1.17]
8.2.3 Females 25 years or older	0	0	Risk Ratio (M‐H, Random, 95% CI)	Not estimable
8.3 CIN3+ associated with HPV 6, 11, 16, 18, 31, 33, 45, 52 and/or 58	1		Risk Ratio (M‐H, Random, 95% CI)	Subtotals only
8.3.1 Females 14 years or younger	0	0	Risk Ratio (M‐H, Random, 95% CI)	Not estimable
8.3.2 Females 15‐25 years	1	11892	Risk Ratio (M‐H, Random, 95% CI)	0.06 [0.00, 1.02]
8.3.3 Females 25 years or older	0	0	Risk Ratio (M‐H, Random, 95% CI)	Not estimable
8.4 CIN2+ irrespective of HPV type	1		Risk Ratio (M‐H, Random, 95% CI)	Subtotals only
8.4.1 Females 14 years or younger	0	0	Risk Ratio (M‐H, Random, 95% CI)	Not estimable
8.4.2 Females 15‐25 years	1	13754	Risk Ratio (M‐H, Random, 95% CI)	1.01 [0.87, 1.17]
8.4.3 Females 25 years or older	0	0	Risk Ratio (M‐H, Random, 95% CI)	Not estimable
8.5 CIN2+ associated with HPV 6, 11, 16 and/or 18	1		Risk Ratio (M‐H, Random, 95% CI)	Subtotals only
8.5.1 Females 14 years or younger	0	0	Risk Ratio (M‐H, Random, 95% CI)	Not estimable
8.5.2 Females 15‐25 years	1	13754	Risk Ratio (M‐H, Random, 95% CI)	1.20 [0.95, 1.53]
8.5.3 Females 25 years or older	0	0	Risk Ratio (M‐H, Random, 95% CI)	Not estimable
8.6 CIN2+ associated with HPV 31, 33, 45, 52 and/or 58	1		Risk Ratio (M‐H, Random, 95% CI)	Subtotals only
8.6.1 Females 14 years or younger	0	0	Risk Ratio (M‐H, Random, 95% CI)	Not estimable
8.6.2 Females 15‐25 years	1	13754	Risk Ratio (M‐H, Random, 95% CI)	0.83 [0.66, 1.04]
8.6.3 Females 25 years or older	0	0	Risk Ratio (M‐H, Random, 95% CI)	Not estimable
8.7 CIN2+ associated with HPV 6, 11, 16, 18, 31, 33, 45, 52 and/or 58	1		Risk Ratio (M‐H, Random, 95% CI)	Subtotals only
8.7.1 Females 14 years or younger	0	0	Risk Ratio (M‐H, Random, 95% CI)	Not estimable
8.7.2 Females 15‐25 years	1	13754	Risk Ratio (M‐H, Random, 95% CI)	0.99 [0.84, 1.16]
8.7.3 Females 25 years or older	0	0	Risk Ratio (M‐H, Random, 95% CI)	Not estimable
8.8 Invasive cervical cancer	1		Risk Ratio (M‐H, Random, 95% CI)	Subtotals only
8.8.1 Females 14 years or younger	0	0	Risk Ratio (M‐H, Random, 95% CI)	Not estimable
8.8.2 Females 15‐25 years	1	11386	Risk Ratio (M‐H, Random, 95% CI)	Not estimable
8.8.3 Females 25 years or older	0	0	Risk Ratio (M‐H, Random, 95% CI)	Not estimable
8.9 CIN2 associated with HPV 6, 11, 16 and/or 18	1		Risk Ratio (M‐H, Random, 95% CI)	Subtotals only
8.9.1 Females 14 years or younger	0	0	Risk Ratio (M‐H, Random, 95% CI)	Not estimable
8.9.2 Females 15‐25 years	1	11656	Risk Ratio (M‐H, Random, 95% CI)	3.00 [0.12, 73.73]
8.9.3 Females 25 years or older	0	0	Risk Ratio (M‐H, Random, 95% CI)	Not estimable
8.10 CIN2 associated with HPV 31, 33, 45, 52 and/or 58	1		Risk Ratio (M‐H, Random, 95% CI)	Subtotals only
8.10.1 Females 14 years or younger	0	0	Risk Ratio (M‐H, Random, 95% CI)	Not estimable
8.10.2 Females 15‐25 years	1	11892	Risk Ratio (M‐H, Random, 95% CI)	0.03 [0.00, 0.23]
8.10.3 Females 25 years or older	0	0	Risk Ratio (M‐H, Random, 95% CI)	Not estimable
8.11 Invasive vaginal or vulvar cancer	1		Risk Ratio (M‐H, Random, 95% CI)	Subtotals only
8.11.1 Females 14 years or younger	0	0	Risk Ratio (M‐H, Random, 95% CI)	Not estimable
8.11.2 Females 15‐25 years	1	11509	Risk Ratio (M‐H, Random, 95% CI)	Not estimable
8.11.3 Females 25 years or older	0	0	Risk Ratio (M‐H, Random, 95% CI)	Not estimable
8.12 High‐grade VIN or VaIN irrespective of HPV type	1		Risk Ratio (M‐H, Random, 95% CI)	Subtotals only
8.12.1 Females 14 years or younger	0	0	Risk Ratio (M‐H, Random, 95% CI)	Not estimable
8.12.2 Females 15‐25 years	1	14052	Risk Ratio (M‐H, Random, 95% CI)	0.88 [0.50, 1.56]
8.12.3 Females 25 years or older	0	0	Risk Ratio (M‐H, Random, 95% CI)	Not estimable
8.13 High‐grade VIN or VaIN associated with HPV 6, 11, 16 and/or 18	1		Risk Ratio (M‐H, Random, 95% CI)	Subtotals only
8.13.1 Females 14 years or younger	0	0	Risk Ratio (M‐H, Random, 95% CI)	Not estimable
8.13.2 Females 15‐25 years	1	14042	Risk Ratio (M‐H, Random, 95% CI)	0.79 [0.36, 1.73]
8.13.3 Females 25 years or older	0	0	Risk Ratio (M‐H, Random, 95% CI)	Not estimable
8.14 High‐grade VIN or VaIN associated with HPV 31, 33, 45, 52 and/or 58	1		Risk Ratio (M‐H, Random, 95% CI)	Subtotals only
8.14.1 Females 14 years or younger	0	0	Risk Ratio (M‐H, Random, 95% CI)	Not estimable
8.14.2 Females 15‐25 years	1	14042	Risk Ratio (M‐H, Random, 95% CI)	0.71 [0.23, 2.25]
8.14.3 Females 25 years or older	0	0	Risk Ratio (M‐H, Random, 95% CI)	Not estimable
8.15 Serious adverse events	3		Risk Ratio (M‐H, Random, 95% CI)	Subtotals only
8.15.1 Females 14 years or younger	1	599	Risk Ratio (M‐H, Random, 95% CI)	0.50 [0.05, 5.50]
8.15.2 Females 15‐25 years	1	14149	Risk Ratio (M‐H, Random, 95% CI)	1.27 [1.05, 1.53]
8.15.3 Females 25 years or older	0	0	Risk Ratio (M‐H, Random, 95% CI)	Not estimable
8.15.4 Females and males	0	0	Risk Ratio (M‐H, Random, 95% CI)	Not estimable
8.15.5 Males 15‐25 years	1	496	Risk Ratio (M‐H, Random, 95% CI)	0.08 [0.00, 1.36]
8.16 Treatment for HPV 6, 11, 16 and/or 18‐related pre‐invasive disease	1		Risk Ratio (M‐H, Random, 95% CI)	Subtotals only
8.16.1 Females 14 years or younger	0	0	Risk Ratio (M‐H, Random, 95% CI)	Not estimable
8.16.2 Females 15‐25 years	1	13754	Risk Ratio (M‐H, Random, 95% CI)	1.05 [0.81, 1.37]
8.16.3 Females 25 years or older	0	0	Risk Ratio (M‐H, Random, 95% CI)	Not estimable
8.16.4 Females and males	0	0	Risk Ratio (M‐H, Random, 95% CI)	Not estimable
8.16.5 Males 15‐25 years	0	0	Risk Ratio (M‐H, Random, 95% CI)	Not estimable
8.17 Treatment for HPV 31, 33, 45, 52 and/or 58‐related pre‐invasive disease	1		Risk Ratio (M‐H, Random, 95% CI)	Subtotals only
8.17.1 Females 14 years or younger	0	0	Risk Ratio (M‐H, Random, 95% CI)	Not estimable
8.17.2 Females 15‐25 years	1	13754	Risk Ratio (M‐H, Random, 95% CI)	0.67 [0.53, 0.86]
8.17.3 Females 25 years or older	0	0	Risk Ratio (M‐H, Random, 95% CI)	Not estimable
8.17.4 Females and males	0	0	Risk Ratio (M‐H, Random, 95% CI)	Not estimable
8.17.5 Males 15‐25 years	0	0	Risk Ratio (M‐H, Random, 95% CI)	Not estimable
8.18 Anogenital warts irrespective of HPV type	1		Risk Ratio (M‐H, Random, 95% CI)	Subtotals only
8.18.1 Females 14 years or younger	0	0	Risk Ratio (M‐H, Random, 95% CI)	Not estimable
8.18.2 Females 15‐25 years	1	14050	Risk Ratio (M‐H, Random, 95% CI)	0.90 [0.66, 1.22]
8.18.3 Females 25 years or older	0	0	Risk Ratio (M‐H, Random, 95% CI)	Not estimable
8.18.4 Females and males	0	0	Risk Ratio (M‐H, Random, 95% CI)	Not estimable
8.18.5 Males 15‐25 years	0	0	Risk Ratio (M‐H, Random, 95% CI)	Not estimable
8.19 Anogenital warts associated with HPV 6, 11, 16 and/or 18	1		Risk Ratio (M‐H, Random, 95% CI)	Subtotals only
8.19.1 Females 14 years or younger	0	0	Risk Ratio (M‐H, Random, 95% CI)	Not estimable
8.19.2 Females 15‐25 years	1	14042	Risk Ratio (M‐H, Random, 95% CI)	0.98 [0.65, 1.47]
8.19.3 Females 25 years or older	0	0	Risk Ratio (M‐H, Random, 95% CI)	Not estimable
8.19.4 Females and males	0	0	Risk Ratio (M‐H, Random, 95% CI)	Not estimable
8.19.5 Males 15‐25 years	0	0	Risk Ratio (M‐H, Random, 95% CI)	Not estimable
8.20 Anogenital warts associated with HPV 31, 33, 45, 52 and/or 58	1		Risk Ratio (M‐H, Random, 95% CI)	Subtotals only
8.20.1 Females 14 years or younger	0	0	Risk Ratio (M‐H, Random, 95% CI)	Not estimable
8.20.2 Females 15‐25 years	1	14042	Risk Ratio (M‐H, Random, 95% CI)	0.75 [0.32, 1.78]
8.20.3 Females 25 years or older	0	0	Risk Ratio (M‐H, Random, 95% CI)	Not estimable
8.20.4 Females and males	0	0	Risk Ratio (M‐H, Random, 95% CI)	Not estimable
8.20.5 Males 15‐25 years	0	0	Risk Ratio (M‐H, Random, 95% CI)	Not estimable
8.21 12‐month persistent HPV 6, 11, 16 and/or 18 infection	1		Risk Ratio (M‐H, Random, 95% CI)	Subtotals only
8.21.1 Females 14 years or younger	0	0	Risk Ratio (M‐H, Random, 95% CI)	Not estimable
8.21.2 Females 15‐25 years	1	13584	Risk Ratio (M‐H, Random, 95% CI)	1.05 [0.93, 1.17]
8.21.3 Females 25 years or older	0	0	Risk Ratio (M‐H, Random, 95% CI)	Not estimable
8.21.4 Females and males	0	0	Risk Ratio (M‐H, Random, 95% CI)	Not estimable
8.21.5 Males 15‐25 years	0	0	Risk Ratio (M‐H, Random, 95% CI)	Not estimable
8.22 12‐month persistent HPV 31, 33, 45, 52 and/or 58 infection	1		Risk Ratio (M‐H, Random, 95% CI)	Subtotals only
8.22.1 Females 14 years or younger	0	0	Risk Ratio (M‐H, Random, 95% CI)	Not estimable
8.22.2 Females 15‐25 years	1	13619	Risk Ratio (M‐H, Random, 95% CI)	0.44 [0.40, 0.48]
8.22.3 Females 25 years or older	0	0	Risk Ratio (M‐H, Random, 95% CI)	Not estimable
8.22.4 Females and males	0	0	Risk Ratio (M‐H, Random, 95% CI)	Not estimable
8.22.5 Males 15‐25 years	0	0	Risk Ratio (M‐H, Random, 95% CI)	Not estimable
8.23 6‐month persistent HPV 6, 11, 16 and/or 18 infection	1		Risk Ratio (M‐H, Random, 95% CI)	Subtotals only
8.23.1 Females 14 years or younger	0	0	Risk Ratio (M‐H, Random, 95% CI)	Not estimable
8.23.2 Females 15‐25 years	1	13613	Risk Ratio (M‐H, Random, 95% CI)	0.98 [0.89, 1.08]
8.23.3 Females 25 years or older	0	0	Risk Ratio (M‐H, Random, 95% CI)	Not estimable
8.23.4 Females and males	0	0	Risk Ratio (M‐H, Random, 95% CI)	Not estimable
8.23.5 Males 15‐25 years	0	0	Risk Ratio (M‐H, Random, 95% CI)	Not estimable
8.24 6‐month persistent HPV 31, 33, 45, 52 and/or 58 infection	1		Risk Ratio (M‐H, Random, 95% CI)	Subtotals only
8.24.1 Females 14 years or younger	0	0	Risk Ratio (M‐H, Random, 95% CI)	Not estimable
8.24.2 Females 15‐25 years	1	13641	Risk Ratio (M‐H, Random, 95% CI)	0.43 [0.39, 0.46]
8.24.3 Females 25 years or older	0	0	Risk Ratio (M‐H, Random, 95% CI)	Not estimable
8.24.4 Females and males	0	0	Risk Ratio (M‐H, Random, 95% CI)	Not estimable
8.24.5 Males 15‐25 years	0	0	Risk Ratio (M‐H, Random, 95% CI)	Not estimable
8.25 Adverse pregnancy outcome: infant/fetal abnormality	1		Risk Ratio (M‐H, Random, 95% CI)	Subtotals only
8.25.1 Females 14 years or younger	0	0	Risk Ratio (M‐H, Random, 95% CI)	Not estimable
8.25.2 Females 15‐25 years	1	14185	Risk Ratio (M‐H, Random, 95% CI)	0.93 [0.56, 1.56]
8.25.3 Females 25 years or older	0	0	Risk Ratio (M‐H, Random, 95% CI)	Not estimable
8.26 Adverse pregnancy outcome: miscarriage	1		Risk Ratio (M‐H, Random, 95% CI)	Subtotals only
8.26.1 Females 14 years or younger	0	0	Risk Ratio (M‐H, Random, 95% CI)	Not estimable
8.26.2 Females 15‐25 years	1	14185	Risk Ratio (M‐H, Random, 95% CI)	0.84 [0.67, 1.05]
8.26.3 Females 25 years or older	0	0	Risk Ratio (M‐H, Random, 95% CI)	Not estimable
8.27 Adverse pregnancy outcome: stillbirth	1		Risk Ratio (M‐H, Random, 95% CI)	Subtotals only
8.27.1 Females 14 years or younger	0	0	Risk Ratio (M‐H, Random, 95% CI)	Not estimable
8.27.2 Females 15‐25 years	1	14185	Risk Ratio (M‐H, Random, 95% CI)	1.00 [0.25, 4.00]
8.27.3 Females 25 years or older	0	0	Risk Ratio (M‐H, Random, 95% CI)	Not estimable
8.28 Local adverse events: pain	3		Risk Ratio (M‐H, Random, 95% CI)	Subtotals only
8.28.1 Females 14 years or younger	1	599	Risk Ratio (M‐H, Random, 95% CI)	1.01 [0.96, 1.07]
8.28.2 Females 15‐25 years	1	14149	Risk Ratio (M‐H, Random, 95% CI)	1.08 [1.06, 1.09]
8.28.3 Females 25 years or older	0	0	Risk Ratio (M‐H, Random, 95% CI)	Not estimable
8.28.4 Females and males	0	0	Risk Ratio (M‐H, Random, 95% CI)	Not estimable
8.28.5 Males 15‐25 years	1	496	Risk Ratio (M‐H, Random, 95% CI)	1.11 [1.00, 1.23]
8.29 Local adverse events: redness	3		Risk Ratio (M‐H, Random, 95% CI)	Subtotals only
8.29.1 Females 14 years or younger	1	599	Risk Ratio (M‐H, Random, 95% CI)	1.16 [0.92, 1.47]
8.29.2 Females 15‐25 years	1	14149	Risk Ratio (M‐H, Random, 95% CI)	1.33 [1.26, 1.40]
8.29.3 Females 25 years or older	0	0	Risk Ratio (M‐H, Random, 95% CI)	Not estimable
8.29.4 Females and males	0	0	Risk Ratio (M‐H, Random, 95% CI)	Not estimable
8.29.5 Males 15‐25 years	1	496	Risk Ratio (M‐H, Random, 95% CI)	0.88 [0.59, 1.32]
8.30 Local adverse events: swelling	3		Risk Ratio (M‐H, Random, 95% CI)	Subtotals only
8.30.1 Females 14 years or younger	1	599	Risk Ratio (M‐H, Random, 95% CI)	1.33 [1.10, 1.61]
8.30.2 Females 15‐25 years	1	14149	Risk Ratio (M‐H, Random, 95% CI)	1.39 [1.33, 1.46]
8.30.3 Females 25 years or older	0	0	Risk Ratio (M‐H, Random, 95% CI)	Not estimable
8.30.4 Females and males	0	0	Risk Ratio (M‐H, Random, 95% CI)	Not estimable
8.30.5 Males 15‐25 years	1	496	Risk Ratio (M‐H, Random, 95% CI)	1.57 [0.96, 2.56]
8.31 Adverse events	3		Risk Ratio (M‐H, Random, 95% CI)	Subtotals only
8.31.1 Females 14 years or younger	1	599	Risk Ratio (M‐H, Random, 95% CI)	1.02 [0.99, 1.06]
8.31.2 Females 15‐25 years	1	14149	Risk Ratio (M‐H, Random, 95% CI)	1.04 [1.03, 1.05]
8.31.3 Females 25 years or older	0	0	Risk Ratio (M‐H, Random, 95% CI)	Not estimable
8.31.4 Females and males	0	0	Risk Ratio (M‐H, Random, 95% CI)	Not estimable
8.31.5 Males 15‐25 years	1	496	Risk Ratio (M‐H, Random, 95% CI)	1.00 [0.93, 1.09]
8.32 Adverse events that led to discontinuation	3		Risk Ratio (M‐H, Random, 95% CI)	Subtotals only
8.32.1 Females 14 years or younger	1	599	Risk Ratio (M‐H, Random, 95% CI)	1.00 [0.06, 15.97]
8.32.2 Females 15‐25 years	1	14149	Risk Ratio (M‐H, Random, 95% CI)	1.60 [0.52, 4.89]
8.32.3 Females 25 years or older	0	0	Risk Ratio (M‐H, Random, 95% CI)	Not estimable
8.32.4 Females and males	0	0	Risk Ratio (M‐H, Random, 95% CI)	Not estimable
8.32.5 Males 15‐25 years	1	496	Risk Ratio (M‐H, Random, 95% CI)	Not estimable
8.33 Specific adverse events: POTS	1		Risk Ratio (M‐H, Random, 95% CI)	Subtotals only
8.33.1 Females 14 years or younger	0	0	Risk Ratio (M‐H, Random, 95% CI)	Not estimable
8.33.2 Females 15‐25 years	1	14149	Risk Ratio (M‐H, Random, 95% CI)	3.00 [0.12, 73.70]
8.33.3 Females 25 years or older	0	0	Risk Ratio (M‐H, Random, 95% CI)	Not estimable
8.33.4 Females and males	0	0	Risk Ratio (M‐H, Random, 95% CI)	Not estimable
8.33.5 Males 15‐25 years	0	0	Risk Ratio (M‐H, Random, 95% CI)	Not estimable
8.34 Specific adverse events: paralysis	1		Risk Ratio (M‐H, Random, 95% CI)	Subtotals only
8.34.1 Females 14 years or younger	0	0	Risk Ratio (M‐H, Random, 95% CI)	Not estimable
8.34.2 Females 15‐25 years	1	14185	Risk Ratio (M‐H, Random, 95% CI)	1.20 [0.37, 3.93]
8.34.3 Females 25 years or older	0	0	Risk Ratio (M‐H, Random, 95% CI)	Not estimable
8.34.4 Females and males	0	0	Risk Ratio (M‐H, Random, 95% CI)	Not estimable
8.34.5 Males 15‐25 years	0	0	Risk Ratio (M‐H, Random, 95% CI)	Not estimable
8.35 Specific adverse events: CRPS	1		Risk Ratio (M‐H, Random, 95% CI)	Subtotals only
8.35.1 Females 14 years or younger	0	0	Risk Ratio (M‐H, Random, 95% CI)	Not estimable
8.35.2 Females 15‐25 years	1	14185	Risk Ratio (M‐H, Random, 95% CI)	1.00 [0.06, 15.99]
8.35.3 Females 25 years or older	0	0	Risk Ratio (M‐H, Random, 95% CI)	Not estimable
8.35.4 Females and males	0	0	Risk Ratio (M‐H, Random, 95% CI)	Not estimable
8.35.5 Males 15‐25 years	0	0	Risk Ratio (M‐H, Random, 95% CI)	Not estimable
8.36 Specific adverse events: ovarian failure	1		Risk Ratio (M‐H, Random, 95% CI)	Subtotals only
8.36.1 Females 14 years or younger	0	0	Risk Ratio (M‐H, Random, 95% CI)	Not estimable
8.36.2 Females 15‐25 years	1	14185	Risk Ratio (M‐H, Random, 95% CI)	0.50 [0.05, 5.51]
8.36.3 Females 25 years or older	0	0	Risk Ratio (M‐H, Random, 95% CI)	Not estimable
8.37 Specific adverse events: infertility	1		Risk Ratio (M‐H, Random, 95% CI)	Subtotals only
8.37.1 Females 14 years or younger	0	0	Risk Ratio (M‐H, Random, 95% CI)	Not estimable
8.37.2 Females 15‐25 years	1	14185	Risk Ratio (M‐H, Random, 95% CI)	1.00 [0.20, 4.95]
8.37.3 Females 25 years or older	0	0	Risk Ratio (M‐H, Random, 95% CI)	Not estimable
8.37.4 Females and males	0	0	Risk Ratio (M‐H, Random, 95% CI)	Not estimable
8.37.5 Males 15‐25 years	0	0	Risk Ratio (M‐H, Random, 95% CI)	Not estimable
8.38 Specific adverse events: sexual behaviour: chlamydia infection	1		Risk Ratio (M‐H, Random, 95% CI)	Subtotals only
8.38.1 Females 14 years or younger	0	0	Risk Ratio (M‐H, Random, 95% CI)	Not estimable
8.38.2 Females 15‐25 years	1	14185	Risk Ratio (M‐H, Random, 95% CI)	0.67 [0.50, 0.91]
8.38.3 Females 25 years or older	0	0	Risk Ratio (M‐H, Random, 95% CI)	Not estimable
8.38.4 Females and males	0	0	Risk Ratio (M‐H, Random, 95% CI)	Not estimable
8.38.5 Males 15‐25 years	0	0	Risk Ratio (M‐H, Random, 95% CI)	Not estimable
8.39 Specific adverse events: sexual behaviour: gonorrhoea infection	1		Risk Ratio (M‐H, Random, 95% CI)	Subtotals only
8.39.1 Females 14 years or younger	0	0	Risk Ratio (M‐H, Random, 95% CI)	Not estimable
8.39.2 Females 15‐25 years	1	14185	Risk Ratio (M‐H, Random, 95% CI)	1.00 [0.06, 15.99]
8.39.3 Females 25 years or older	0	0	Risk Ratio (M‐H, Random, 95% CI)	Not estimable
8.39.4 Females and males	0	0	Risk Ratio (M‐H, Random, 95% CI)	Not estimable
8.39.5 Males 15‐25 years	0	0	Risk Ratio (M‐H, Random, 95% CI)	Not estimable
8.40 Specific adverse events: sexual behaviour: genital herpes infection	1		Risk Ratio (M‐H, Random, 95% CI)	Subtotals only
8.40.1 Females 14 years or younger	0	0	Risk Ratio (M‐H, Random, 95% CI)	Not estimable
8.40.2 Females 15‐25 years	1	14185	Risk Ratio (M‐H, Random, 95% CI)	1.22 [0.89, 1.68]
8.40.3 Females 25 years or older	0	0	Risk Ratio (M‐H, Random, 95% CI)	Not estimable
8.40.4 Females and males	0	0	Risk Ratio (M‐H, Random, 95% CI)	Not estimable
8.40.5 Males 15‐25 years	0	0	Risk Ratio (M‐H, Random, 95% CI)	Not estimable
8.41 All‐cause mortality	3		Risk Ratio (M‐H, Random, 95% CI)	Subtotals only
8.41.1 Females 14 years or younger	1	599	Risk Ratio (M‐H, Random, 95% CI)	Not estimable
8.41.2 Females 15‐25 years	1	14149	Risk Ratio (M‐H, Random, 95% CI)	1.20 [0.37, 3.93]
8.41.3 Females 25 years or older	0	0	Risk Ratio (M‐H, Random, 95% CI)	Not estimable
8.41.4 Females and males	0	0	Risk Ratio (M‐H, Random, 95% CI)	Not estimable
8.41.5 Males 15‐25 years	1	496	Risk Ratio (M‐H, Random, 95% CI)	Not estimable

**Comparison 9:** Cervarix 2 doses versus Cervarix 3 doses

Outcome or subgroup title	No. of studies	No. of participants	Statistical method	Effect size
9.1 Serious adverse events	2		Risk Ratio (M‐H, Random, 95% CI)	Subtotals only
9.1.1 Females 14 years or younger	1	310	Risk Ratio (M‐H, Random, 95% CI)	0.67 [0.19, 2.32]
9.1.2 Females 15‐25 years	1	960	Risk Ratio (M‐H, Random, 95% CI)	1.08 [0.62, 1.89]
9.1.3 Females 25 years or older	0	0	Risk Ratio (M‐H, Random, 95% CI)	Not estimable
9.1.4 Females and males	0	0	Risk Ratio (M‐H, Random, 95% CI)	Not estimable
9.1.5 Males 15‐25 years	0	0	Risk Ratio (M‐H, Random, 95% CI)	Not estimable
9.2 Adverse pregnancy outcome: miscarriage	2		Risk Ratio (M‐H, Random, 95% CI)	Subtotals only
9.2.1 Females 14 years or younger	1	305	Risk Ratio (M‐H, Random, 95% CI)	Not estimable
9.2.2 Females 15‐25 years	1	960	Risk Ratio (M‐H, Random, 95% CI)	1.99 [0.24, 16.44]
9.2.3 Females 25 years or older	0	0	Risk Ratio (M‐H, Random, 95% CI)	Not estimable
9.3 Adverse pregnancy outcome: infant/fetal abnormality	2		Risk Ratio (M‐H, Random, 95% CI)	Subtotals only
9.3.1 Females 14 years or younger	1	310	Risk Ratio (M‐H, Random, 95% CI)	3.00 [0.12, 73.08]
9.3.2 Females 15‐25 years	1	960	Risk Ratio (M‐H, Random, 95% CI)	1.00 [0.04, 24.40]
9.3.3 Females 25 years or older	0	0	Risk Ratio (M‐H, Random, 95% CI)	Not estimable
9.4 Adverse pregnancy outcome: pre‐term birth	1		Risk Ratio (M‐H, Random, 95% CI)	Subtotals only
9.4.1 Females 14 years or younger	0	0	Risk Ratio (M‐H, Random, 95% CI)	Not estimable
9.4.2 Females 15‐25 years	1	960	Risk Ratio (M‐H, Random, 95% CI)	1.00 [0.04, 24.40]
9.4.3 Females 25 years or older	0	0	Risk Ratio (M‐H, Random, 95% CI)	Not estimable
9.5 Local adverse events: pain	1		Risk Ratio (M‐H, Random, 95% CI)	Subtotals only
9.5.1 Females 14 years or younger	0	0	Risk Ratio (M‐H, Random, 95% CI)	Not estimable
9.5.2 Females 15‐25 years	1	953	Risk Ratio (M‐H, Random, 95% CI)	0.99 [0.95, 1.03]
9.5.3 Females 25 years or older	0	0	Risk Ratio (M‐H, Random, 95% CI)	Not estimable
9.5.4 Females and males	0	0	Risk Ratio (M‐H, Random, 95% CI)	Not estimable
9.5.5 Males 15‐25 years	0	0	Risk Ratio (M‐H, Random, 95% CI)	Not estimable
9.6 Local adverse events: redness	1		Risk Ratio (M‐H, Random, 95% CI)	Subtotals only
9.6.1 Females 14 years or younger	0	0	Risk Ratio (M‐H, Random, 95% CI)	Not estimable
9.6.2 Females 15‐25 years	1	953	Risk Ratio (M‐H, Random, 95% CI)	0.79 [0.70, 0.90]
9.6.3 Females 25 years or older	0	0	Risk Ratio (M‐H, Random, 95% CI)	Not estimable
9.6.4 Females and males	0	0	Risk Ratio (M‐H, Random, 95% CI)	Not estimable
9.6.5 Males 15‐25 years	0	0	Risk Ratio (M‐H, Random, 95% CI)	Not estimable
9.7 Local adverse events: swelling	1		Risk Ratio (M‐H, Random, 95% CI)	Subtotals only
9.7.1 Females 14 years or younger	0	0	Risk Ratio (M‐H, Random, 95% CI)	Not estimable
9.7.2 Females 15‐25 years	1	953	Risk Ratio (M‐H, Random, 95% CI)	0.74 [0.63, 0.87]
9.7.3 Females 25 years or older	0	0	Risk Ratio (M‐H, Random, 95% CI)	Not estimable
9.7.4 Females and males	0	0	Risk Ratio (M‐H, Random, 95% CI)	Not estimable
9.7.5 Males 15‐25 years	0	0	Risk Ratio (M‐H, Random, 95% CI)	Not estimable
9.8 Unsolicited adverse events	1		Risk Ratio (M‐H, Random, 95% CI)	Subtotals only
9.8.1 Females 14 years or younger	0	0	Risk Ratio (M‐H, Random, 95% CI)	Not estimable
9.8.2 Females 15‐25 years	1	960	Risk Ratio (M‐H, Random, 95% CI)	0.76 [0.64, 0.90]
9.8.3 Females 25 years or older	0	0	Risk Ratio (M‐H, Random, 95% CI)	Not estimable
9.8.4 Females and males	0	0	Risk Ratio (M‐H, Random, 95% CI)	Not estimable
9.8.5 Males 15‐25 years	0	0	Risk Ratio (M‐H, Random, 95% CI)	Not estimable
9.9 Adverse events that led to discontinuation	1		Risk Ratio (M‐H, Random, 95% CI)	Subtotals only
9.9.1 Females 14 years or younger	0	0	Risk Ratio (M‐H, Random, 95% CI)	Not estimable
9.9.2 Females 15‐25 years	1	960	Risk Ratio (M‐H, Random, 95% CI)	0.66 [0.06, 7.28]
9.9.3 Females 25 years or older	0	0	Risk Ratio (M‐H, Random, 95% CI)	Not estimable
9.9.4 Females and males	0	0	Risk Ratio (M‐H, Random, 95% CI)	Not estimable
9.9.5 Males 15‐25 years	0	0	Risk Ratio (M‐H, Random, 95% CI)	Not estimable
9.10 All‐cause mortality	2		Risk Ratio (M‐H, Random, 95% CI)	Subtotals only
9.10.1 Females 14 years or younger	1	310	Risk Ratio (M‐H, Random, 95% CI)	Not estimable
9.10.2 Females 15‐25 years	1	960	Risk Ratio (M‐H, Random, 95% CI)	Not estimable
9.10.3 Females 25 years or older	0	0	Risk Ratio (M‐H, Random, 95% CI)	Not estimable
9.10.4 Females and males	0	0	Risk Ratio (M‐H, Random, 95% CI)	Not estimable
9.10.5 Males 15‐25 years	0	0	Risk Ratio (M‐H, Random, 95% CI)	Not estimable

**Comparison 10:** Cervarix 1 dose versus Cervarix 2 doses

Outcome or subgroup title	No. of studies	No. of participants	Statistical method	Effect size
10.1 Serious adverse events	1		Risk Ratio (M‐H, Random, 95% CI)	Subtotals only
10.1.1 Females 14 years or younger	1	310	Risk Ratio (M‐H, Random, 95% CI)	2.00 [0.61, 6.50]
10.1.2 Females 15‐25 years	0	0	Risk Ratio (M‐H, Random, 95% CI)	Not estimable
10.1.3 Females 25 years or older	0	0	Risk Ratio (M‐H, Random, 95% CI)	Not estimable
10.1.4 Females and males	0	0	Risk Ratio (M‐H, Random, 95% CI)	Not estimable
10.1.5 Males 15‐25 years	0	0	Risk Ratio (M‐H, Random, 95% CI)	Not estimable
10.2 Adverse pregnancy outcome: infant/fetal abnormality	1		Risk Ratio (M‐H, Random, 95% CI)	Subtotals only
10.2.1 Females 14 years or younger	1	310	Risk Ratio (M‐H, Random, 95% CI)	0.33 [0.01, 8.12]
10.2.2 Females 15‐25 years	0	0	Risk Ratio (M‐H, Random, 95% CI)	Not estimable
10.2.3 Females 25 years or older	0	0	Risk Ratio (M‐H, Random, 95% CI)	Not estimable
10.3 Adverse pregnancy outcome: miscarriage	1		Risk Ratio (M‐H, Random, 95% CI)	Subtotals only
10.3.1 Females 14 years or younger	1	305	Risk Ratio (M‐H, Random, 95% CI)	Not estimable
10.3.2 Females 15‐25 years	0	0	Risk Ratio (M‐H, Random, 95% CI)	Not estimable
10.3.3 Females 25 years or older	0	0	Risk Ratio (M‐H, Random, 95% CI)	Not estimable
10.4 All‐cause mortality	1		Risk Ratio (M‐H, Random, 95% CI)	Subtotals only
10.4.1 Females 14 years or younger	1	310	Risk Ratio (M‐H, Random, 95% CI)	Not estimable
10.4.2 Females 15‐25 years	0	0	Risk Ratio (M‐H, Random, 95% CI)	Not estimable
10.4.3 Females 25 years or older	0	0	Risk Ratio (M‐H, Random, 95% CI)	Not estimable
10.4.4 Females and males	0	0	Risk Ratio (M‐H, Random, 95% CI)	Not estimable
10.4.5 Males 15‐25 years	0	0	Risk Ratio (M‐H, Random, 95% CI)	Not estimable

**Comparison 11:** Cervarix 1 dose versus Cervarix 3 doses

Outcome or subgroup title	No. of studies	No. of participants	Statistical method	Effect size
11.1 Serious adverse events	1		Risk Ratio (M‐H, Random, 95% CI)	Subtotals only
11.1.1 Females 14 years or younger	1	310	Risk Ratio (M‐H, Random, 95% CI)	1.33 [0.47, 3.75]
11.1.2 Females 15‐25 years	0	0	Risk Ratio (M‐H, Random, 95% CI)	Not estimable
11.1.3 Females 25 years or older	0	0	Risk Ratio (M‐H, Random, 95% CI)	Not estimable
11.1.4 Females and males	0	0	Risk Ratio (M‐H, Random, 95% CI)	Not estimable
11.1.5 Males 15‐25 years	0	0	Risk Ratio (M‐H, Random, 95% CI)	Not estimable
11.2 Adverse pregnancy outcome: infant/fetal abnormality	1		Risk Ratio (M‐H, Random, 95% CI)	Subtotals only
11.2.1 Females 14 years or younger	1	310	Risk Ratio (M‐H, Random, 95% CI)	Not estimable
11.2.2 Females 15‐25 years	0	0	Risk Ratio (M‐H, Random, 95% CI)	Not estimable
11.2.3 Females 25 years or older	0	0	Risk Ratio (M‐H, Random, 95% CI)	Not estimable
11.3 Adverse pregnancy outcome: miscarriage	1		Risk Ratio (M‐H, Random, 95% CI)	Subtotals only
11.3.1 Females 14 years or younger	1	308	Risk Ratio (M‐H, Random, 95% CI)	Not estimable
11.3.2 Females 15‐25 years	0	0	Risk Ratio (M‐H, Random, 95% CI)	Not estimable
11.3.3 Females 25 years or older	0	0	Risk Ratio (M‐H, Random, 95% CI)	Not estimable
11.4 All‐cause mortality	1		Risk Ratio (M‐H, Random, 95% CI)	Subtotals only
11.4.1 Females 14 years or younger	1	310	Risk Ratio (M‐H, Random, 95% CI)	Not estimable
11.4.2 Females 15‐25 years	0	0	Risk Ratio (M‐H, Random, 95% CI)	Not estimable
11.4.3 Females 25 years or older	0	0	Risk Ratio (M‐H, Random, 95% CI)	Not estimable
11.4.4 Females and males	0	0	Risk Ratio (M‐H, Random, 95% CI)	Not estimable
11.4.5 Males 15‐25 years	0	0	Risk Ratio (M‐H, Random, 95% CI)	Not estimable

**Comparison 12:** Gardasil 2 doses versus Gardasil 3 doses

Outcome or subgroup title	No. of studies	No. of participants	Statistical method	Effect size
12.1 Serious adverse events	2		Risk Ratio (M‐H, Random, 95% CI)	Subtotals only
12.1.1 Females 14 years or younger	2	1227	Risk Ratio (M‐H, Random, 95% CI)	0.79 [0.36, 1.71]
12.1.2 Females 15‐25 years	0	0	Risk Ratio (M‐H, Random, 95% CI)	Not estimable
12.1.3 Females 25 years or older	0	0	Risk Ratio (M‐H, Random, 95% CI)	Not estimable
12.1.4 Females and males	0	0	Risk Ratio (M‐H, Random, 95% CI)	Not estimable
12.1.5 Males 15‐25 years	0	0	Risk Ratio (M‐H, Random, 95% CI)	Not estimable
12.2 Local adverse events: pain	1		Risk Ratio (M‐H, Random, 95% CI)	Subtotals only
12.2.1 Females 14 years or younger	1	713	Risk Ratio (M‐H, Random, 95% CI)	0.93 [0.87, 1.00]
12.2.2 Females 15‐25 years	0	0	Risk Ratio (M‐H, Random, 95% CI)	Not estimable
12.2.3 Females 25 years or older	0	0	Risk Ratio (M‐H, Random, 95% CI)	Not estimable
12.2.4 Females and males	0	0	Risk Ratio (M‐H, Random, 95% CI)	Not estimable
12.2.5 Males 15‐25 years	0	0	Risk Ratio (M‐H, Random, 95% CI)	Not estimable
12.3 Local adverse events: redness	1		Risk Ratio (M‐H, Random, 95% CI)	Subtotals only
12.3.1 Females 14 years or younger	1	713	Risk Ratio (M‐H, Random, 95% CI)	0.85 [0.71, 1.02]
12.3.2 Females 15‐25 years	0	0	Risk Ratio (M‐H, Random, 95% CI)	Not estimable
12.3.3 Females 25 years or older	0	0	Risk Ratio (M‐H, Random, 95% CI)	Not estimable
12.3.4 Females and males	0	0	Risk Ratio (M‐H, Random, 95% CI)	Not estimable
12.3.5 Males 15‐25 years	0	0	Risk Ratio (M‐H, Random, 95% CI)	Not estimable
12.4 Local adverse events: swelling	1		Risk Ratio (M‐H, Random, 95% CI)	Subtotals only
12.4.1 Females 14 years or younger	1	713	Risk Ratio (M‐H, Random, 95% CI)	0.83 [0.66, 1.04]
12.4.2 Females 15‐25 years	0	0	Risk Ratio (M‐H, Random, 95% CI)	Not estimable
12.4.3 Females 25 years or older	0	0	Risk Ratio (M‐H, Random, 95% CI)	Not estimable
12.4.4 Females and males	0	0	Risk Ratio (M‐H, Random, 95% CI)	Not estimable
12.4.5 Males 15‐25 years	0	0	Risk Ratio (M‐H, Random, 95% CI)	Not estimable
12.5 Adverse events	1		Risk Ratio (M‐H, Random, 95% CI)	Subtotals only
12.5.1 Females 14 years or younger	1	713	Risk Ratio (M‐H, Random, 95% CI)	0.99 [0.94, 1.03]
12.5.2 Females 15‐25 years	0	0	Risk Ratio (M‐H, Random, 95% CI)	Not estimable
12.5.3 Females 25 years or older	0	0	Risk Ratio (M‐H, Random, 95% CI)	Not estimable
12.5.4 Females and males	0	0	Risk Ratio (M‐H, Random, 95% CI)	Not estimable
12.5.5 Males 15‐25 years	0	0	Risk Ratio (M‐H, Random, 95% CI)	Not estimable
12.6 Unsolicited adverse events	1		Risk Ratio (M‐H, Random, 95% CI)	Subtotals only
12.6.1 Females 14 years or younger	1	716	Risk Ratio (M‐H, Random, 95% CI)	0.95 [0.75, 1.21]
12.6.2 Females 15‐25 years	0	0	Risk Ratio (M‐H, Random, 95% CI)	Not estimable
12.6.3 Females 25 years or older	0	0	Risk Ratio (M‐H, Random, 95% CI)	Not estimable
12.6.4 Females and males	0	0	Risk Ratio (M‐H, Random, 95% CI)	Not estimable
12.6.5 Males 15‐25 years	0	0	Risk Ratio (M‐H, Random, 95% CI)	Not estimable
12.7 Adverse events that led to discontinuation	1		Risk Ratio (M‐H, Random, 95% CI)	Subtotals only
12.7.1 Females 14 years or younger	1	716	Risk Ratio (M‐H, Random, 95% CI)	0.33 [0.01, 8.16]
12.7.2 Females 15‐25 years	0	0	Risk Ratio (M‐H, Random, 95% CI)	Not estimable
12.7.3 Females 25 years or older	0	0	Risk Ratio (M‐H, Random, 95% CI)	Not estimable
12.7.4 Females and males	0	0	Risk Ratio (M‐H, Random, 95% CI)	Not estimable
12.7.5 Males 15‐25 years	0	0	Risk Ratio (M‐H, Random, 95% CI)	Not estimable
12.8 Specific adverse events: POTS	1		Risk Ratio (M‐H, Random, 95% CI)	Subtotals only
12.8.1 Females 14 years or younger	1	716	Risk Ratio (M‐H, Random, 95% CI)	Not estimable
12.8.2 Females 15‐25 years	0	0	Risk Ratio (M‐H, Random, 95% CI)	Not estimable
12.8.3 Females 25 years or older	0	0	Risk Ratio (M‐H, Random, 95% CI)	Not estimable
12.8.4 Females and males	0	0	Risk Ratio (M‐H, Random, 95% CI)	Not estimable
12.8.5 Males 15‐25 years	0	0	Risk Ratio (M‐H, Random, 95% CI)	Not estimable
12.9 All‐cause mortality	1		Risk Ratio (M‐H, Random, 95% CI)	Subtotals only
12.9.1 Females 14 years or younger	1	716	Risk Ratio (M‐H, Random, 95% CI)	0.33 [0.01, 8.16]
12.9.2 Females 15‐25 years	0	0	Risk Ratio (M‐H, Random, 95% CI)	Not estimable
12.9.3 Females 25 years or older	0	0	Risk Ratio (M‐H, Random, 95% CI)	Not estimable
12.9.4 Females and males	0	0	Risk Ratio (M‐H, Random, 95% CI)	Not estimable
12.9.5 Males 15‐25 years	0	0	Risk Ratio (M‐H, Random, 95% CI)	Not estimable

**Comparison 13:** Gardasil‐9 2 doses versus Gardasil‐9 3 doses

Outcome or subgroup title	No. of studies	No. of participants	Statistical method	Effect size
13.1 Serious adverse events	2		Risk Ratio (M‐H, Random, 95% CI)	Subtotals only
13.1.1 Females 14 years or younger	2	904	Risk Ratio (M‐H, Random, 95% CI)	1.01 [0.49, 2.09]
13.1.2 Females 15‐25 years	0	0	Risk Ratio (M‐H, Random, 95% CI)	Not estimable
13.1.3 Females 25 years or older	0	0	Risk Ratio (M‐H, Random, 95% CI)	Not estimable
13.1.4 Females and males	0	0	Risk Ratio (M‐H, Random, 95% CI)	Not estimable
13.1.5 Males 15‐25 years	0	0	Risk Ratio (M‐H, Random, 95% CI)	Not estimable
13.2 Adverse pregnancy outcome: infant/fetal abnormality	1		Risk Ratio (M‐H, Random, 95% CI)	Subtotals only
13.2.1 Females 14 years or younger	1	310	Risk Ratio (M‐H, Random, 95% CI)	Not estimable
13.2.2 Females 15‐25 years	0	0	Risk Ratio (M‐H, Random, 95% CI)	Not estimable
13.2.3 Females 25 years or older	0	0	Risk Ratio (M‐H, Random, 95% CI)	Not estimable
13.3 Adverse pregnancy outcome: miscarriage	1		Risk Ratio (M‐H, Random, 95% CI)	Subtotals only
13.3.1 Females 14 years or younger	1	307	Risk Ratio (M‐H, Random, 95% CI)	Not estimable
13.3.2 Females 15‐25 years	0	0	Risk Ratio (M‐H, Random, 95% CI)	Not estimable
13.3.3 Females 25 years or older	0	0	Risk Ratio (M‐H, Random, 95% CI)	Not estimable
13.4 Local adverse events: pain	1		Risk Ratio (M‐H, Random, 95% CI)	Subtotals only
13.4.1 Females 14 years or younger	1	594	Risk Ratio (M‐H, Random, 95% CI)	0.94 [0.66, 1.34]
13.4.2 Females 15‐25 years	0	0	Risk Ratio (M‐H, Random, 95% CI)	Not estimable
13.4.3 Females 25 years or older	0	0	Risk Ratio (M‐H, Random, 95% CI)	Not estimable
13.4.4 Females and males	0	0	Risk Ratio (M‐H, Random, 95% CI)	Not estimable
13.4.5 Males 15‐25 years	0	0	Risk Ratio (M‐H, Random, 95% CI)	Not estimable
13.5 All‐cause mortality	2		Risk Ratio (M‐H, Random, 95% CI)	Subtotals only
13.5.1 Females 14 years or younger	2	904	Risk Ratio (M‐H, Random, 95% CI)	1.01 [0.11, 9.68]
13.5.2 Females 15‐25 years	0	0	Risk Ratio (M‐H, Random, 95% CI)	Not estimable
13.5.3 Females 25 years or older	0	0	Risk Ratio (M‐H, Random, 95% CI)	Not estimable
13.5.4 Females and males	0	0	Risk Ratio (M‐H, Random, 95% CI)	Not estimable
13.5.5 Males 15‐25 years	0	0	Risk Ratio (M‐H, Random, 95% CI)	Not estimable

**Comparison 14:** Gardasil‐9 1 dose versus Gardasil‐9 2 doses

Outcome or subgroup title	No. of studies	No. of participants	Statistical method	Effect size
14.1 Serious adverse events	1		Risk Ratio (M‐H, Random, 95% CI)	Subtotals only
14.1.1 Females 14 years or younger	1	310	Risk Ratio (M‐H, Random, 95% CI)	1.00 [0.39, 2.60]
14.1.2 Females 15‐25 years	0	0	Risk Ratio (M‐H, Random, 95% CI)	Not estimable
14.1.3 Females 25 years or older	0	0	Risk Ratio (M‐H, Random, 95% CI)	Not estimable
14.1.4 Females and males	0	0	Risk Ratio (M‐H, Random, 95% CI)	Not estimable
14.1.5 Males 15‐25 years	0	0	Risk Ratio (M‐H, Random, 95% CI)	Not estimable
14.2 Adverse pregnancy outcome: infant/fetal abnormality	1		Risk Ratio (M‐H, Random, 95% CI)	Subtotals only
14.2.1 Females 14 years or younger	1	310	Risk Ratio (M‐H, Random, 95% CI)	Not estimable
14.2.2 Females 15‐25 years	0	0	Risk Ratio (M‐H, Random, 95% CI)	Not estimable
14.2.3 Females 25 years or older	0	0	Risk Ratio (M‐H, Random, 95% CI)	Not estimable
14.3 Adverse pregnancy outcome: miscarriage	1		Risk Ratio (M‐H, Random, 95% CI)	Subtotals only
14.3.1 Females 14 years or younger	1	307	Risk Ratio (M‐H, Random, 95% CI)	2.98 [0.12, 72.60]
14.3.2 Females 15‐25 years	0	0	Risk Ratio (M‐H, Random, 95% CI)	Not estimable
14.3.3 Females 25 years or older	0	0	Risk Ratio (M‐H, Random, 95% CI)	Not estimable
14.4 All‐cause mortality	1		Risk Ratio (M‐H, Random, 95% CI)	Subtotals only
14.4.1 Females 14 years or younger	1	310	Risk Ratio (M‐H, Random, 95% CI)	0.33 [0.01, 8.12]
14.4.2 Females 15‐25 years	0	0	Risk Ratio (M‐H, Random, 95% CI)	Not estimable
14.4.3 Females 25 years or older	0	0	Risk Ratio (M‐H, Random, 95% CI)	Not estimable
14.4.4 Females and males	0	0	Risk Ratio (M‐H, Random, 95% CI)	Not estimable
14.4.5 Males 15‐25 years	0	0	Risk Ratio (M‐H, Random, 95% CI)	Not estimable

**Comparison 15:** Gardasil‐9 1 dose versus Gardasil‐9 3 doses

Outcome or subgroup title	No. of studies	No. of participants	Statistical method	Effect size
15.1 Serious adverse events	1		Risk Ratio (M‐H, Random, 95% CI)	Subtotals only
15.1.1 Females 14 years or younger	1	310	Risk Ratio (M‐H, Random, 95% CI)	1.00 [0.39, 2.60]
15.1.2 Females 15‐25 years	0	0	Risk Ratio (M‐H, Random, 95% CI)	Not estimable
15.1.3 Females 25 years or older	0	0	Risk Ratio (M‐H, Random, 95% CI)	Not estimable
15.1.4 Females and males	0	0	Risk Ratio (M‐H, Random, 95% CI)	Not estimable
15.1.5 Males 15‐25 years	0	0	Risk Ratio (M‐H, Random, 95% CI)	Not estimable
15.2 Adverse pregnancy outcome: infant/fetal abnormality	1		Risk Ratio (M‐H, Random, 95% CI)	Subtotals only
15.2.1 Females 14 years or younger	1	310	Risk Ratio (M‐H, Random, 95% CI)	Not estimable
15.2.2 Females 15‐25 years	0	0	Risk Ratio (M‐H, Random, 95% CI)	Not estimable
15.2.3 Females 25 years or older	0	0	Risk Ratio (M‐H, Random, 95% CI)	Not estimable
15.3 Adverse pregnancy outcome: miscarriage	1		Risk Ratio (M‐H, Random, 95% CI)	Subtotals only
15.3.1 Females 14 years or younger	1	308	Risk Ratio (M‐H, Random, 95% CI)	3.00 [0.12, 73.07]
15.3.2 Females 15‐25 years	0	0	Risk Ratio (M‐H, Random, 95% CI)	Not estimable
15.3.3 Females 25 years or older	0	0	Risk Ratio (M‐H, Random, 95% CI)	Not estimable
15.4 All‐cause mortality	1		Risk Ratio (M‐H, Random, 95% CI)	Subtotals only
15.4.1 Females 14 years or younger	1	310	Risk Ratio (M‐H, Random, 95% CI)	Not estimable
15.4.2 Females 15‐25 years	0	0	Risk Ratio (M‐H, Random, 95% CI)	Not estimable
15.4.3 Females 25 years or older	0	0	Risk Ratio (M‐H, Random, 95% CI)	Not estimable
15.4.4 Females and males	0	0	Risk Ratio (M‐H, Random, 95% CI)	Not estimable
15.4.5 Males 15‐25 years	0	0	Risk Ratio (M‐H, Random, 95% CI)	Not estimable

**Comparison 16:** Cecolin 2 doses versus Cecolin 3 doses

Outcome or subgroup title	No. of studies	No. of participants	Statistical method	Effect size
16.1 Serious adverse events	1		Risk Ratio (M‐H, Random, 95% CI)	Subtotals only
16.1.1 Females 14 years or younger	1	605	Risk Ratio (M‐H, Random, 95% CI)	0.20 [0.01, 4.19]
16.1.2 Females 15‐25 years	0	0	Risk Ratio (M‐H, Random, 95% CI)	Not estimable
16.1.3 Females 25 years or older	0	0	Risk Ratio (M‐H, Random, 95% CI)	Not estimable
16.1.4 Females and males	0	0	Risk Ratio (M‐H, Random, 95% CI)	Not estimable
16.1.5 Males 15‐25 years	0	0	Risk Ratio (M‐H, Random, 95% CI)	Not estimable
16.2 Local adverse events: pain	1		Risk Ratio (M‐H, Random, 95% CI)	Subtotals only
16.2.1 Females 14 years or younger	1	605	Risk Ratio (M‐H, Random, 95% CI)	0.97 [0.72, 1.30]
16.2.2 Females 15‐25 years	0	0	Risk Ratio (M‐H, Random, 95% CI)	Not estimable
16.2.3 Females 25 years or older	0	0	Risk Ratio (M‐H, Random, 95% CI)	Not estimable
16.2.4 Females and males	0	0	Risk Ratio (M‐H, Random, 95% CI)	Not estimable
16.2.5 Males 15‐25 years	0	0	Risk Ratio (M‐H, Random, 95% CI)	Not estimable
16.3 Local adverse events: redness	1		Risk Ratio (M‐H, Random, 95% CI)	Subtotals only
16.3.1 Females 14 years or younger	1	605	Risk Ratio (M‐H, Random, 95% CI)	0.58 [0.17, 1.95]
16.3.2 Females 15‐25 years	0	0	Risk Ratio (M‐H, Random, 95% CI)	Not estimable
16.3.3 Females 25 years or older	0	0	Risk Ratio (M‐H, Random, 95% CI)	Not estimable
16.3.4 Females and males	0	0	Risk Ratio (M‐H, Random, 95% CI)	Not estimable
16.3.5 Males 15‐25 years	0	0	Risk Ratio (M‐H, Random, 95% CI)	Not estimable
16.4 Local adverse events: swelling	1		Risk Ratio (M‐H, Random, 95% CI)	Subtotals only
16.4.1 Females 14 years or younger	1	605	Risk Ratio (M‐H, Random, 95% CI)	1.09 [0.51, 2.36]
16.4.2 Females 15‐25 years	0	0	Risk Ratio (M‐H, Random, 95% CI)	Not estimable
16.4.3 Females 25 years or older	0	0	Risk Ratio (M‐H, Random, 95% CI)	Not estimable
16.4.4 Females and males	0	0	Risk Ratio (M‐H, Random, 95% CI)	Not estimable
16.4.5 Males 15‐25 years	0	0	Risk Ratio (M‐H, Random, 95% CI)	Not estimable
16.5 Unsolicited adverse events	1		Risk Ratio (M‐H, Random, 95% CI)	Subtotals only
16.5.1 Females 14 years or younger	1	605	Risk Ratio (M‐H, Random, 95% CI)	0.67 [0.56, 0.81]
16.5.2 Females 15‐25 years	0	0	Risk Ratio (M‐H, Random, 95% CI)	Not estimable
16.5.3 Females 25 years or older	0	0	Risk Ratio (M‐H, Random, 95% CI)	Not estimable
16.5.4 Females and males	0	0	Risk Ratio (M‐H, Random, 95% CI)	Not estimable
16.5.5 Males 15‐25 years	0	0	Risk Ratio (M‐H, Random, 95% CI)	Not estimable

## Characteristics of studies

### Characteristics of included studies [ordered by study ID]

**Table:** 2v4v Draper 2013‐UK

***Study characteristics***	
Methods	Randomised, parallel group trial
Participants	198 females: 12 to 15 years old at first dose
Interventions	2‐valent Cervarix (GSK): 3 doses at months 0, 1, 6 4‐valent Gardasil (Merck): 3 doses at months 0, 1, 6
Outcomes	Adverse events: serious, local, systemic Follow‐up: 7, 12, 15 months
Setting	2 study centres in the UK (HIC) National screening programme: yes; coverage: at least 70%
Notes	Funding: public/non‐profit: UK Medical Research Council; UK Department of Health Conflicts of interest: no

**Table:** 2v4v Einstein 2009‐USA

***Study characteristics***	
Methods	Randomised, parallel group trial
Participants	1106 females: 18 to 45 years old at first dose
Interventions	2‐valent Cervarix (GSK): 3 doses at months 0, 1, 6 + placebo at month 2 4‐valent Gardasil (Merck): 3 doses at months 0, 2, 6 + placebo at month 1
Outcomes	Adverse events: fatal, serious, leading to discontinuation, total, unsolicited, local, systemic Pregnancy outcomes Follow‐up: month 7, month 12, month 18, month 24, month 36, month 48 and month 60
Setting	40 study centres in the USA (HIC) National screening programme: yes; coverage: at least 70%
Notes	Funding: private/industry: GlaxoSmithKline Biologicals Conflicts of interest: yes, trial authors include employees and stock options holders of GSK

**Table:** 2v4v Gilca 2015‐CAN

***Study characteristics***	
Methods	Randomised, parallel group trial
Participants	366 females: 9 to 10 years old at first dose
Interventions	4‐valent Gardasil (Merck): 3 doses at month 0, 6, 42 4‐valent Gardasil (Merck): 2 doses at month 0, 6 + 2‐valent Cervarix (GSK): 1 dose at month 42
Outcomes	Adverse events: serious, local, systemic Follow‐up: 12 months
Setting	1 study centre in Canada (HIC) National screening programme: yes; coverage: at least 70%
Notes	Funding: public/non‐profit: Quebec Ministry of Health and Social Services Conflicts of interest: no

**Table:** 2v4v Leung 2015‐INT

***Study characteristics***	
Methods	Randomised, parallel group trial
Participants	1075 females: 9 to 14 years old at first dose
Interventions	2‐valent Cervarix (GSK): 2 doses at months 0, 6 + placebo at month 2 4‐valent Gardasil (Merck): 2 doses at months 0, 6 + placebo at month 2 4‐valent Gardasil (Merck): 3 doses at months 0, 2, 6
Outcomes	Adverse events: fatal, serious, leading to discontinuation, total, unsolicited, local, systemic Specific adverse events: Guillain Barre syndrome, POTS Pregnancy outcomes Follow‐up: 7, 12, 18, 24, 36 months
Setting	21 study centres in France, Hong Kong, Singapore, Sweden (HIC) National screening programme: yes; coverage: Singapore and France: 50% to 70%, Sweden: at least 70%, China: unknown
Notes	Funding: private/industry: GlaxoSmithKline Biologicals Conflicts of interest: yes, trial authors include employees, stock options holders, and shareholders in GSK

**Table:** 2v4v Nelson 2013‐HKG

***Study characteristics***	
Methods	Randomised, parallel group trial
Participants	11 females: 18 to 26 years old at first dose
Interventions	2‐valent Cervarix (GSK): 3 doses at months 0, 2, 6 4‐valent Gardasil (Merck): 3 doses at months 0, 2, 6
Outcomes	Local adverse events Serious adverse events (2 events reported but not by group; not included in analysis) Follow‐up: 14 days
Setting	1 study centre in China (Hong Kong Special Administrative Region) (UMIC) National screening programme: yes; coverage: unknown
Notes	Trial arms where vaccine was administered intradermally were not included. Funding: mixed: the Chinese University of Hong Kong; PharmaJet provided intradermal injection devices and associated consumables Conflicts of interest: yes, trial authors include funding and fee recipients from Merck and GSK

**Table:** 2v4v Sangar 2015‐IND

***Study characteristics***	
Methods	Randomised, parallel group trial
Participants	69 females: 18 to 25 years old at first dose
Interventions	2‐valent Cervarix (GSK): 3 doses at months 0, 1, 6 4‐valent Gardasil (Merck): 3 doses at months 0, 2, 6
Outcomes	Adverse events: serious, local, systemic Follow‐up: 7 months
Setting	1 study centre in India (LMIC) National screening programme: yes; coverage: less than 10%
Notes	Funding: public/non‐profit: Mr. Madhusudan Vakharia and Byramjee Jeejeebhoy Trust Conflicts of interest: no

**Table:** 2v9v DoRIS 2022‐TZN

***Study characteristics***	
Methods	Randomised, parallel group trial
Participants	930 females: 9 to 14 years old at first dose
Interventions	2‐valent Cervarix (GSK): 1 dose 2‐valent Cervarix (GSK): 2 doses at months 0, 6 2‐valent Cervarix (GSK): 3 doses at months 0, 1, 6 9‐valent Gardasil‐9 (Merck): 1 dose 9‐valent Gardasil‐9 (Merck): 2 doses at months 0, 6 9‐valent Gardasil‐9 (Merck): 3 doses at months 0, 2, 6
Outcomes	Adverse events: fatal, serious Pregnancy outcomes Follow‐up: 24 months
Setting	1 study centre in Tanzania (LMIC) National screening programme: no
Notes	Funding: public/non‐profit: UK Department for International Development (DFID)/MRC/Wellcome Trust Joint Global Health Trials Scheme; Bill and Melinda Gates Foundation; National Cancer Institute Conflicts of interest: no

**Table:** 2v9v Gilca 2018‐CAN

***Study characteristics***	
Methods	Randomised, parallel group trial
Participants	371 females and males: 9 to 10 years old at first dose
Interventions	2‐valent Cervarix (GSK): 1 dose at month 0 + 9‐valent Gardasil‐9 (Merck): 1 dose at month 6 9‐valent Gardasil‐9 (Merck): 2 doses at month 0, 6
Outcomes	Adverse events: local, systemic Follow‐up: 4 days after each dose
Setting	1 study centre in Canada (HIC) National screening programme: yes; coverage: at least 70%
Notes	Funding: public/non‐profit: Quebec Ministry of Health and Social Services; Bill & Melinda Gates Foundation Conflicts of interest: no

**Table:** 2v9v KENSHE 2021‐KEN

***Study characteristics***	
Methods	Randomised, parallel group trial
Participants	2275 females: 15 to 20 years old at first dose
Interventions	9‐valent Gardasil‐9 (Merck): 1 dose + delayed MenVeo 2‐valent Cervarix (GSK): 1 dose + delayed MENVEO MENVEO + delayed 9‐valent Gardasil‐9 (Merck)
Outcomes	Persistent HPV infection Serious adverse events Pregnancy outcomes Follow‐up: 18 months
Setting	3 study centres in Kenya (LMIC) National screening programme: yes; coverage: 10% to 50%
Notes	Funding: public/non‐profit: Bill and Melinda Gates Foundation, University of Washington Conflicts of interest: yes, trial authors include fee recipients from Merck

**Table:** 2v Bhatla 2010‐IND

***Study characteristics***	
Methods	Randomised, parallel group trial
Participants	354 females: 18 to 35 years old at first dose
Interventions	2‐valent Cervarix (GSK): 3 doses at months 0, 1, 6 Adjuvant placebo: 3 doses at months 0, 1, 6
Outcomes	Adverse events: fatal, serious, leading to discontinuation, total, unsolicited, local, systemic Pregnancy outcomes Follow‐up: month 7 (1 month after last dose)
Setting	4 study centres in India (LMIC) National screening programme: yes; coverage: less than 10%
Notes	Funding: private/industry: GlaxoSmithKline Biologicals Conflicts of interest: yes, trial authors include employees and stock options owners of GlaxoSmithKline

**Table:** 2v Carozzi 2016‐ITA

***Study characteristics***	
Methods	Randomised, parallel group trial
Participants	832 females: 25 years old at first dose
Interventions	2‐valent Cervarix (GSK): 3 doses at months 0, 1, 6 No intervention
Outcomes	Efficacy: participation in cervical screening, incident HPV infection Total adverse events (not included, no comparison group: only reported for vaccinated participants) Follow‐up: 30 months
Setting	1 study centre in Italy (HIC) National screening programme: yes; coverage: 10% to 50%
Notes	Funding: public/non‐profit: Istituto Toscano Tumori Conflicts of interest: no

**Table:** 2v CVT 2011‐CRI

***Study characteristics***	
Methods	Randomised, parallel group trial
Participants	7466 females: 18 to 25 years old at first dose
Interventions	2‐valent Cervarix (GSK): 3 doses at months 0, 1, 6 Hepatitis A virus (HAV) vaccine: 3 doses at months 0, 1, 6
Outcomes	Adverse events: fatal, serious, unsolicited, local, systemic Pregnancy outcomes Efficacy: intraepithelial neoplasia (CIN2+), incident HPV infection, persistent HPV infection, treatment for HPV disease Follow‐up: 12, 24, 36, 48 months
Setting	1 study centre in Costa Rica (UMIC) National screening programme: yes; coverage: unknown
Notes	Funding: mixed: US National Cancer Institute, GlaxoSmithKline Conflicts of interest: yes, trial authors include patent holders of the vaccine under investigation

**Table:** 2v Garcia‐Sicilia 2010‐EU

***Study characteristics***	
Methods	Randomised, parallel group trial
Participants	751 females: 10 to 18 years old at first dose
Interventions	2‐valent Cervarix (GSK): 3 doses at months 0, 1, 6 2‐valent Cervarix (GSK): 3 doses at months 0, 1, 6 + Boostrix Polio (dTpa‐IPV): 1 dose at month 0 2‐valent Cervarix (GSK): 3 doses at months 0, 1, 6 + Boostrix Polio (dTpa‐IPV): 1 dose at month ‐1
Outcomes	Adverse events: fatal, serious, leading to discontinuation, total, local, systemic Pregnancy outcomes Follow‐up: month 7/8 and month 12/13
Setting	35 study centres in France, Germany, Spain (HIC) National screening programme: yes; coverage: France: more than 50% to 70%, Germany: at least 70%, Spain: unknown
Notes	Funding: private/industry: GlaxoSmithKline Biologicals Conflicts of interest: yes, authors include employees of GlaxoSmithKline Biologicals

**Table:** 2v Harper 2004‐BRA/NA

***Study characteristics***	
Methods	Randomised, parallel group trial
Participants	1113 females: 15 to 25 years old at first dose
Interventions	2‐valent Cervarix (GSK): 3 doses at months 0, 1, 6 Adjuvant placebo: 3 doses at months 0, 1, 6
Outcomes	Adverse events: fatal, serious, leading to discontinuation, total, unsolicited, local, systemic Efficacy: cervical intraepithelial neoplasia (CIN2, CIN2+, CIN3, CIN3+), incident HPV infection, persistent HPV infection Follow‐up: 18, 27 months; Extension: 4.5, 6.4 years; Brazil extension: 7.3, 8.4, 9.4, 11.3 years
Setting	32 study centres in Brazil, Canada, USA (HIC, UMIC) National screening programme: yes; coverage: at least 70%
Notes	Funding: private/industry: GlaxoSmithKline Biologicals Conflicts of interest: yes, trial authors include employees of GlaxoSmithKline Biologicals

**Table:** 2v Khatun 2012‐BGD

***Study characteristics***	
Methods	Randomised, parallel group trial
Participants	67 females: 9 to 13 years old at first dose
Interventions	2‐valent Cervarix (GSK): 3 doses at months 0, 1, 6 No intervention
Outcomes	Adverse events: serious, total, local, systemic Follow‐up: month 7 (1 month after last dose) Data only reported for the intervention group
Setting	3 study centres in Bangladesh (LMIC) National screening programme: yes; coverage: unknown
Notes	Funding: private/industry: Grameenphone Ltd, Bangladesh Conflicts of interest: no

**Table:** 2v Kim 2010‐KOR

***Study characteristics***	
Methods	Randomised, parallel group trial
Participants	321 females: 10 to 14 years old at first dose
Interventions	2‐valent Cervarix (GSK): 3 doses at months 0, 1, 6 Hepatitis A vaccine (HAV): 3 doses at months 0, 1, 6
Outcomes	Adverse events: fatal, serious, leading to discontinuation, total, unsolicited, local, systemic Pregnancy outcomes Follow‐up: month 7 (1 month after last dose)
Setting	8 study centres in South Korea (HIC) National screening programme: yes; coverage: 10% to 50%
Notes	Funding: private/industry: GlaxoSmithKline Biologicals Conflicts of interest: no

**Table:** 2v Kim 2011‐KOR

***Study characteristics***	
Methods	Randomised, parallel group trial
Participants	225 females: 15 to 25 years old at first dose
Interventions	2‐valent Cervarix (GSK): 3 doses at months 0, 1, 6 Adjuvant placebo: 3 doses at months 0, 1, 6
Outcomes	Adverse events: fatal, serious, leading to discontinuation, total, unsolicited, local, systemic Pregnancy outcomes Follow‐up: month 7 (1 month after last dose)
Setting	6 study centres in South Korea (HIC) National screening programme: yes; coverage: 10% to 50%
Notes	Funding: private/industry: GlaxoSmithKline Biologicals Conflicts of interest: yes, authors include employees and share owners in GlaxoSmithKline Biologicals

**Table:** 2v Konno 2010‐JPN

***Study characteristics***	
Methods	Randomised, parallel group trial
Participants	1040 females: 20 to 25 years old at first dose
Interventions	2‐valent Cervarix (GSK): 3 doses at months 0, 1, 6 HAV vaccine: 3 doses at months 0, 1, 6
Outcomes	Adverse events: fatal, serious, leading to discontinuation, total, unsolicited, local, systemic Specific adverse events: paralysis Pregnancy outcomes Efficacy: CIN2+, CIN3+, incident HPV infection, persistent HPV infection Follow‐up: 7 months, 13.6 months (interim), 24 months (final main), 36 months (interim extension), 48 months (final extension)
Setting	13 study centres in Japan (HIC) National screening programme: yes; coverage: 10% to 50%
Notes	Funding: private/industry: GlaxoSmithKline Conflicts of interest: yes; several of the authors are employees of GlaxoSmithKline Biologicals

**Table:** 2v Lehtinen 2018‐FIN

***Study characteristics***	
Methods	Cluster‐randomised trial
Participants	34,412 females and males in 33 clusters (communities): 12 to 15 years old at first dose
Interventions	90% Cervarix (GSK), 10% HBV vaccine: 3 doses at months 0, 1, 6 (11 clusters) Girls: 90% Cervarix (GSK), 10% HBV vaccine: 3 doses at months 0, 1, 6; boys: HBV vaccine: 3 doses at months 0, 1, 6 (11 clusters) HBV vaccine: 3 doses at months 0, 1, 6 (11 clusters)
Outcomes	All‐cause mortality Effectiveness: HPV infection Adverse events: serious, total, unsolicited, local, systemic Specific adverse events: Guillain‐Barré syndrome, paralysis Pregnancy outcomes Follow‐up: 12 months, 6.5 years Results not reported by randomised group.
Setting	33 study centres in Finland (HIC) National screening programme: yes; coverage: at least 70%
Notes	Funding: mixed: Academy of Finland, ADVANCE, GlaxoSmithKline Biologicals Conflicts of interest: yes, authors include employees and stock shareholders in GlaxoSmithKline Biologicals

**Table:** 2v Leroux‐Roels 2011‐BEL

***Study characteristics***	
Methods	Randomised, parallel group trial
Participants	152 females: 20 to 25 years old at first dose
Interventions	2‐valent Cervarix (GSK): 3 doses at months 0, 1, 6 + hepatitis B virus (HBV) vaccine (Engerix B): 4 doses at months 0, 1, 2, 12 HBV vaccine (Engerix B): 4 doses at months 0, 1, 2, 12
Outcomes	Adverse events: serious, leading to discontinuation, total, local, systemic Pregnancy outcomes Specific adverse events: Guillain‐Barré syndrome Follow‐up: month 7, month 13
Setting	2 study centres in Belgium (HIC) National screening programme: yes; coverage: 50% to 70%
Notes	Funding: private/industry: GlaxoSmithKline Biologicals Conflicts of interest: yes, authors include employees of GlaxoSmithKline Biologicals

**Table:** 2v Lim 2014‐MYS

***Study characteristics***	
Methods	Randomised, parallel group trial
Participants	271 females: 18 to 35 years old at first dose
Interventions	2‐valent Cervarix (GSK): 3 doses at months 0, 1, 6 Adjuvant placebo: 3 doses at months 0, 1, 6
Outcomes	Adverse events: fatal, serious, leading to discontinuation, total, local, systemic Pregnancy outcomes Specific adverse events: Guillain‐Barré syndrome Follow‐up: month 7
Setting	2 study centres in Malaysia (UMIC) National screening programme: yes; coverage: 10% to 50%
Notes	Funding: private/industry: GlaxoSmithKline Biologicals Conflicts of interest: yes, authors include employees of and stock owners of GlaxoSmithKline

**Table:** 2v Lin 2018‐LA

***Study characteristics***	
Methods	Randomised, parallel group trial
Participants	148 females: 4 to 6 years old at first dose
Interventions	2‐valent Cervarix (GSK): 2 doses at months 0, 6 MMR vaccine: 1 dose day 0 + DTPa vaccine: 1 dose month 6
Outcomes	All‐cause mortality Adverse events: serious, leading to discontinuation, total, unsolicited, local, systemic Persistent HPV infection (oral) Follow‐up: months 7, 12, 18, 24 and 36
Setting	7 study centres in Mexico, Colombia, Panama (UMIC) National screening programme: yes; coverage: Colombia and Panama: 10% to 50%, Mexico: unknown
Notes	Funding: private/industry: GlaxoSmithKline Biologicals Conflicts of interest: yes, authors include employees and stock shareholders of the GSK group of companies

**Table:** 2v Medina 2010‐INT

***Study characteristics***	
Methods	Randomised, parallel group trial
Participants	2067 females: 10 to 14 years old at first dose
Interventions	2‐valent Cervarix (GSK): 3 doses at months 0, 1, 6 HAV vaccine: 3 doses at months 0, 1, 6
Outcomes	All‐cause mortality Adverse events: serious, leading to discontinuation, unsolicited, local, systemic Pregnancy outcomes Follow‐up: 7 months; 12 months
Setting	57 study centres in Australia, Colombia, the Czech Republic, France, Germany, Honduras, Korea, Norway, Panama, Spain, Sweden, Taiwan (HIC, UMIC, LMIC) National screening programme: mixed; coverage: mixed
Notes	Funding: private/industry: GlaxoSmithKline Conflicts of interest: yes, authors include employees of GlaxoSmithKline Biologicals

**Table:** 2v Ngan 2010‐HKG

***Study characteristics***	
Methods	Randomised, parallel group trial
Participants	300 females: 18 to 35 years old at first dose
Interventions	2‐valent Cervarix (GSK): 3 doses at months 0, 1, 6 Adjuvant placebo: 3 doses at months 0, 1, 6
Outcomes	All‐cause mortality Adverse events: serious, leading to discontinuation, total, unsolicited, local, systemic Pregnancy outcomes Follow‐up: 7 months
Setting	1 study centre in Hong Kong SAR, China (HIC) National screening programme: yes; coverage: unknown
Notes	Funding: private/industry: GlaxoSmithKline Conflicts of interest: yes, authors include employees and shareholders of GlaxoSmithKline Biologicals

**Table:** 2v PATRICIA 2012‐INT

***Study characteristics***	
Methods	Randomised, parallel group trial
Participants	18644 females: 15 to 25 years old at first dose
Interventions	2‐valent Cervarix (GSK): 3 doses at months 0, 1, 6 HAV vaccine: 3 doses at months 0, 1, 6
Outcomes	All‐cause mortality Efficacy: intraepithelial neoplasia (CIN2+, CIN3+, VIN2, VaIN2), treatment for HPV disease, incident HPV infection, persistent HPV infection Adverse events: serious, leading to discontinuation, total, unsolicited, local, systemic Specific adverse events: infertility Pregnancy outcomes Follow‐up: 7 months; clinical outcomes/SAE: mean follow‐up 14.8 months, 34.9 months and 43.7 months
Setting	135 study centres in Australia, Belgium, Brazil, Canada, Finland, Germany, Italy, Mexico, Philippines, Spain, Taiwan, Thailand, UK, USA (HIC, UMIC, LMIC) National screening programme: mixed; coverage: mixed
Notes	Funding: private/industry: GlaxoSmithKline Conflicts of interest: yes, authors include employees and stock owners of GlaxoSmithKline Biologicals and one author holds a relevant patent

**Table:** 2v Pedersen 2012‐NA/EU

***Study characteristics***	
Methods	Randomised, parallel group trial
Participants	814 females: 9 to 15 years old at first dose
Interventions	2‐valent Cervarix (GSK): 3 doses at months 0, 1, 6 2‐valent Cervarix (GSK) + hepatitis A and B (HAB) vaccine (Twinrix): both 3 doses at months 0, 1, 6 HAB vaccine (Twinrix): 3 doses at months 0, 1, 6
Outcomes	Adverse events: fatal, serious, leading to discontinuation, total, unsolicited, local, systemic Specific adverse events: Guillain‐Barré syndrome Follow‐up: months 7 and 12
Setting	21 study centres in Canada, Denmark, Hungary and Sweden (HIC) National screening programme: yes; coverage: Canada and Sweden: at least 70%, Denmark: 50% to 70%, Hungary: less than 10%
Notes	Funding: private/industry: GlaxoSmithKline Biologicals Conflicts of interest: yes, authors include employees and stock options owners of GlaxoSmithKline Biologicals

**Table:** 2v Petaja 2009‐FIN

***Study characteristics***	
Methods	Randomised, parallel group trial
Participants	270 males: 10 to 18 years old at first dose
Interventions	2‐valent Cervarix (GSK): 3 doses at months 0, 1, 6 Hepatitis B virus vaccine: 3 doses at months 0, 1, 6
Outcomes	Adverse events: fatal, serious, leading to discontinuation, total, unsolicited, local, systemic Specific adverse events: Guillain‐Barré syndrome Follow‐up: 7 months, 12 months
Setting	7 study centres in Finland (HIC) National screening programme: yes; coverage: at least 70%
Notes	Funding: private/industry: GlaxoSmithKline Conflicts of interest: not reported

**Table:** 2v Romanowski 2011‐CAN/GER

***Study characteristics***	
Methods	Randomised, parallel group trial
Participants	960 females: 9 to 25 years old at first dose
Interventions	2‐valent Cervarix (GSK): 3 doses at months 0, 1, 6, 20 μg 2‐valent Cervarix (GSK): 2 doses at months 0, 6, 20 μg 2‐valent Cervarix (GSK): 2 doses at months 0, 6, 40 μg 2‐valent Cervarix (GSK): 2 doses at months 0, 2, 40 μg
Outcomes	Adverse events: fatal, serious, leading to discontinuation, total, unsolicited, local, systemic Pregnancy outcomes Follow‐up: 7, 12, 18, 24, 36, 48, 60 months
Setting	21 study centres in Canada, Germany (HIC) National screening programme: yes; coverage: at least 70%
Notes	Funding: private/industry: GlaxoSmithKline Biologicals Conflicts of interest: yes, authors include employees and stock options owners of GlaxoSmithKline Biologicals, the vaccine developer

**Table:** 2v Schmeink 2011‐NLD/SWE

***Study characteristics***	
Methods	Randomised, parallel group trial
Participants	741 females: 9 to 15 years old at first dose
Interventions	2‐valent Cervarix (GSK): 3 doses at months 0, 1, 6 2‐valent Cervarix (GSK) + HBV vaccine (Engerix‐B): both 3 doses at months 0, 1, 6 HBV vaccine (Engerix‐B): 3 doses at months 0, 1, 6
Outcomes	Adverse events: serious total, unsolicited, local, systemic Follow‐up: 7 months, 12 months
Setting	7 study centres in the Netherlands, Sweden (HIC) National screening programme: yes; coverage: at least 70%
Notes	Funding: private/industry: GlaxoSmithKline Conflicts of interest: yes, authors include employees and stock owners of GlaxoSmithKline Biologicals

**Table:** 2v Sow 2013‐SEN/TZN

***Study characteristics***	
Methods	Randomised, parallel group trial
Participants	676 females (HIV seronegative): 10 to 25 years old at first dose
Interventions	2‐valent Cervarix (GSK): 3 doses at months 0, 1, 6 Adjuvant placebo: 3 doses at months 0, 1, 6
Outcomes	All‐cause mortality Adverse events: fatal, serious, leading to discontinuation, total, unsolicited, local, systemic Pregnancy outcomes Follow‐up: 7 months, 12 months
Setting	2 study centres in Senegal, Tanzania (LMIC) National screening programme: no
Notes	Funding: private/industry: GlaxoSmithKline Biologicals Conflicts of interest: yes, authors include employees and stock owners in the GlaxoSmithKline group of companies

**Table:** 2v VIVIANE 2014‐INT

***Study characteristics***	
Methods	Randomised, parallel group trial
Participants	5747 females: 24 to 72 years old at first dose
Interventions	2‐valent Cervarix (GSK): 3 doses at months 0, 1, 6 Adjuvant placebo: 3 doses at months 0, 1, 6
Outcomes	Efficacy: intraepithelial neoplasia (CIN2+), persistent HPV infection, treatment for HPV disease Adverse events: fatal, serious, leading to discontinuation, total, unsolicited, local, systemic Pregnancy outcomes Follow‐up: 7 months, 48 months, 84 months
Setting	76 study centres in Australia, Canada, Mexico, Peru, Philippines, Portugal, Russia, Singapore, Thailand, the Netherlands, UK, USA (HIC, UMIC, LMIC) National screening programme: mixed; coverage: mixed
Notes	Funding: private/industry: GlaxoSmithKline Biologicals Conflicts of interest: yes, trial authors include employees of the GlaxoSmithKline group of companies

**Table:** 2v Zhu 2014‐CHNa

***Study characteristics***	
Methods	Randomised, parallel group trial
Participants	6081 females: 18 to 25 years old at first dose
Interventions	2‐valent Cervarix (GSK): 3 doses at months 0, 1, 6 Adjuvant placebo: 3 doses at months 0, 1, 6
Outcomes	Efficacy: intraepithelial neoplasia (CIN2+, CIN3+), persistent HPV infection, incident HPV infection Adverse events: fatal, serious, leading to discontinuation, total, unsolicited, local, systemic Pregnancy outcomes Follow‐up: months 6, 12, 18, 24, 30, 36, 42, 48, 60, 66 and 72
Setting	4 study centres in China (UMIC) National screening programme: yes; coverage: unknown
Notes	Funding: private/industry: GlaxoSmithKline Biologicals Conflicts of interest: yes, authors include employees and shareholders in the GSK group of companies

**Table:** 2v Zhu 2014‐CHNb

***Study characteristics***	
Methods	Randomised, parallel group trial
Participants	750 females: 9 to 17 years old at first dose
Interventions	2‐valent Cervarix (GSK): 3 doses at months 0, 1, 6 Adjuvant placebo: 3 doses at months 0, 1, 6
Outcomes	Adverse events: fatal, serious, leading to discontinuation, total, unsolicited, local, systemic Follow‐up: month 7, month 12
Setting	1 study centre in China (UMIC) National screening programme: yes; coverage: unknown
Notes	Funding: private/industry: GlaxoSmithKline Biologicals Conflicts of interest: yes, several authors are affiliated with GSK

**Table:** 2v Zhu 2014‐CHNc

***Study characteristics***	
Methods	Randomised, parallel group trial
Participants	1212 females: 26 to 45 years old at first dose
Interventions	2‐valent Cervarix (GSK): 3 doses at months 0, 1, 6 HBV vaccine (Engerix B): 3 doses at months 0, 1, 6
Outcomes	Adverse events: fatal, serious, leading to discontinuation, total, unsolicited, local, systemic Pregnancy outcomes Follow‐up: month 7 and month 12
Setting	1 study centre in China (UMIC) National screening programme: yes; coverage: unknown
Notes	Funding: private/industry: GlaxoSmithKline Biologicals Conflicts of interest: yes, several authors are affiliated with GSK

**Table:** 4v9v Garland 2015‐INT

***Study characteristics***	
Methods	Randomised, parallel group trial
Participants	924 females: 12 to 26 years old at first dose
Interventions	4‐valent Gardasil (Merck): 3 doses + 9‐valent Gardasil‐9 (Merck): 3 doses at month 0, 1, 6 one year later 4‐valent Gardasil (Merck): 3 doses + saline placebo: 3 doses at month 0, 1, 6 one year later
Outcomes	Adverse events: fatal, serious, leading to discontinuation, total, local, systemic Pregnancy outcomes Follow‐up: 7 months
Setting	32 study centres in Australia, Canada, Colombia, Denmark, Hong Kong, Mexico, Sweden, USA (HIC, UMIC) National screening programme: yes; coverage: mixed
Notes	Funding: private/industry: Merck & Co., Inc. Conflicts of interest: yes, authors include employees and potential stock and/or stock options owners of Merck

**Table:** 4v9v Joura 2015‐INT

***Study characteristics***	
Methods	Randomised, parallel group trial
Participants	14,215 females: 16 to 26 years old at first dose
Interventions	9‐valent Gardasil‐9 (Merck): 3 doses at months 0, 2, 6 4‐valent Gardasil (Merck): 3 doses at months 0, 2, 6
Outcomes	All‐cause mortality Efficacy: cervical cancer, vulvar cancer, vaginal cancer, intraepithelial neoplasia (CIN2, CIN2+, CIN3+, VIN2+, VaIN2+), persistent HPV infection, anogenital warts, treatment for HPV disease Adverse events: serious, local, systemic Pregnancy outcomes Follow‐up: 6 years
Setting	105 study centres in Austria, Brazil, Canada, Chile, Colombia, Denmark, Germany, Hong Kong, Japan, Korea, Mexico, New Zealand, Norway, Peru, Sweden, Taiwan, Thailand, USA (HIC, UMIC) National screening programme: mixed
Notes	Funding: private/industry: Merck Conflicts of interest: yes, authors include employees and stockholders of Merck and Sanofi Pasteur

**Table:** 4v9v Van Damme 2016‐EU

***Study characteristics***	
Methods	Randomised, parallel group trial
Participants	500 males: 16 to 26 years old at first dose
Interventions	9‐valent Gardasil‐9 (Merck): 3 doses at months 0, 2, 6 4‐valent Gardasil (Merck): 3 doses at months 0, 2, 6
Outcomes	Adverse events: fatal, serious, leading to discontinuation, total, local, systemic Follow‐up: 7 months
Setting	7 study centres in Belgium, Germany and the Netherlands (HIC) National screening programme: yes; coverage: Belgium: 50% to 70%, Germany and the Netherlands: at least 70%
Notes	Funding: private/industry: Sanofi Pasteur MSD Conflicts of interest: yes, authors include employees of Merck and Sanofi‐Pasteur MSD

**Table:** 4v9v Vesikari 2015‐EU

***Study characteristics***	
Methods	Randomised, parallel group trial
Participants	600 females: 9 to 16 years old at first dose
Interventions	9‐valent Gardasil‐9 (Merck): 3 doses at months 0, 2, 6 4‐valent Gardasil (Merck): 3 doses at months 0, 2, 6
Outcomes	Adverse events: fatal, serious, leading to discontinuation, total, local, systemic Follow‐up: 7 months
Setting	24 study centres in Belgium, Denmark, Finland, Italy, Spain and Sweden (HIC) National screening programme: yes; coverage: Italy: 10% to 50%, Belgium and Denmark: 50% to 70%, Finland and Sweden: at least 70%, Spain: unknown
Notes	Funding: private/industry: Sanofi Pasteur MSD Conflicts of interest: yes, authors include employees of Merck and Sanofi Pasteur MSD

**Table:** 4v Chang 2020‐USA

***Study characteristics***	
Methods	Randomised, parallel group trial
Participants	1715 females and males: 10 to 17 years old at first dose
Interventions	4‐valent Gardasil (Merck): 3 doses at months 0, 2, 6 + MenACYW‐TT + Tdap: 1 dose 4‐valent Gardasil (Merck): 3 doses at months 0, 2, 6 + Tdap: 1 dose MenACYW‐TT: 1 dose MC4‐CRM: 1 dose
Outcomes	Adverse events: fatal, serious, leading to discontinuation, unsolicited, local, systemic Follow‐up: month 7 (1 month after last dose)
Setting	40 study centres in USA (HIC) National screening programme: yes; coverage: at least 70%
Notes	Funding: private/industry: Sanofi Pasteur Conflicts of interest: yes, authors include employees of Sanofi Pasteur

**Table:** 4v Dobson 2013‐CAN

***Study characteristics***	
Methods	Randomised, parallel group trial
Participants	520 females: 9 to 13 years old at first dose
Interventions	4‐valent Gardasil (Merck): 2 doses at months 0, 6 4‐valent Gardasil (Merck): 3 doses at months 0, 2, 6
Outcomes	Serious adverse events Follow‐up: 30 days after each dose
Setting	3 study centres in Canada (HIC) National screening programme: yes; coverage: at least 70%
Notes	Funding: mixed: Ministries of Health in the provinces of British Columbia, Nova Scotia and Quebec; Provincial Health Services Authority; Merck Laboratories Inc conducted the antibody assays at no cost to the study. Conflicts of interest: yes, authors include those serving on the advisory board, consulting, receiving grant support, travel expenses, or payment for lectures for GlaxoSmithKline or Merck

**Table:** 4v EVRI 2016‐ZAF

***Study characteristics***	
Methods	Randomised, parallel group trial
Participants	402 females (HIV seronegative): 16 to 24 years old at first dose
Interventions	4‐valent Gardasil (Merck): 3 doses at months 0, 2, 6 Saline placebo: 3 doses at months 0, 2, 6
Outcomes	Incident HPV infection Adverse events: serious, total Follow‐up: 7 months
Setting	2 study centres in South Africa (UMIC) National screening programme: yes; coverage: 50% to 70%
Notes	Funding: mixed: Merck; National Cancer Institute Conflicts of interest: yes, among authors are those on the Speaker's Bureau of Merck, research funding recipient and co‐investigator on Sanofi Pasteur vaccine trial, and former vaccine advisory board of GSK

**Table:** 4v Foresta 2015‐ITA

***Study characteristics***	
Methods	Randomised, parallel group trial
Participants	72 males (infertile): age at first dose was not reported
Interventions	4‐valent Gardasil (Merck): 3 doses at months 0, 2, 6 No intervention
Outcomes	Persistent HPV infection (in semen) Follow‐up: 24 months
Setting	1 study centre in Italy (HIC) National screening programme: yes; coverage: 10% to 50%
Notes	Funding: not reported Conflicts of interest: not reported

**Table:** 4v FUTURE 2007‐INT

***Study characteristics***	
Methods	Randomised, parallel group trial
Participants	5455 females: 16 to 24 years old at first dose
Interventions	4‐valent Gardasil (Merck): 3 doses at months 0, 2, 6 Adjuvant placebo: 3 doses at months 0, 2, 6
Outcomes	All‐cause mortality Efficacy: cervical cancer, vulval/vaginal cancer, intraepithelial neoplasia (CIN2, CIN3, VIN2+, VaIN2+), anogenital warts Adverse events: serious, local, systemic Pregnancy outcomes Follow‐up: months 7, 12, 18, 24, 30, 36 and 48
Setting	62 study centres in 16 countries in Asia‐Pacific, North and Latin America, and Europe (HIC, UMIC, LMIC) National screening programme: unknown, countries not reported
Notes	Funding: private/industry: Merck Sharp & Dohme Corp Conflicts of interest: yes, study authors include employees and stock owners of Merck and recipients of consulting fees, advisory board fees, lecture fees, or grant support from GlaxoSmithKline and/or Merck

**Table:** 4v FUTURE I/II 2010‐INT

***Study characteristics***	
Methods	Pooled analysis of included RCTs 4v FUTURE 2007‐INT and 4v FUTURE II 2007‐INT
Participants	17,622 females: 16 to 26 years old at first dose
Interventions	4‐valent Gardasil (Merck): 3 doses at months 0, 2, 6 Adjuvant placebo: 3 doses at months 0, 2, 6
Outcomes	Efficacy: intraepithelial neoplasia (CIN2, CIN2+, CIN3, CIN3+, VIN2+, VaIN2+), anogenital warts Follow‐up: up to 4.9 years
Setting	24 countries (HIC, UMIC, LMIC) National screening programme: unknown, countries not reported
Notes	Funding: private/industry: Merck Research Laboratories Conflicts of interest: yes: study authors include employees of Merck and recipients of consulting fees, advisory board fees, lecture fees, or grant support from GlaxoSmithKline and/or Merck

**Table:** 4v FUTURE II 2007‐INT

***Study characteristics***	
Methods	Randomised, parallel group trial
Participants	12,167 females: 16 to 23 years old at first dose
Interventions	4‐valent Gardasil (Merck): 3 doses at months 0, 2, 6 Adjuvant placebo: 3 doses at months 0, 2, 6
Outcomes	All‐cause mortality Efficacy: cervical cancer, intraepithelial neoplasia (CIN3) Adverse events: serious, local, systemic Pregnancy outcomes Follow‐up: 12 months, 24 months, 36 months, 48 months
Setting	90 study centres in 13 countries: Brazil, Colombia, Denmark, Finland, Iceland, Mexico, Norway, Peru, Poland, Singapore, Sweden, UK, USA (HIC, UMIC) National screening programme: yes; coverage: mixed
Notes	Funding: private/industry: Merck Sharp & Dohme Conflicts of interest: yes, study authors include employees and stock options owners of Merck and recipients of consulting fees, advisory board fees, lecture fees, or grant support from GlaxoSmithKline and/or Merck

**Table:** 4v FUTURE III 2009‐INT

***Study characteristics***	
Methods	Randomised, parallel group trial
Participants	3819 females: 24 to 45 years old at first dose
Interventions	4‐valent Gardasil (Merck): 3 doses at months 0, 2, 6 Adjuvant placebo: 3 doses at months 0, 2, 6
Outcomes	All‐cause mortality Efficacy: intraepithelial neoplasia (CIN2+, VIN2+, VaIN2+), anogenital warts, persistent HPV infection, treatment for HPV disease Adverse events: serious, leading to discontinuation, total, local, systemic Pregnancy outcomes Follow‐up: month 7, 12, 18, 24, 30, 36, 42 and 48
Setting	38 study centres in Colombia, France, Germany, Philippines, Spain, Thailand, USA (HIC, UMIC, LMIC) National screening programme: mixed
Notes	Funding: private/industry: Merck Sharp & Dohme Conflicts of interest: yes, study authors include employees, steering committee members, advisory board members, consultants, grant recipients, and potential stock owners of Merck, GlaxoSmithKline, and/or Sanofi‐Pasteur

**Table:** 4v Giuliano 2011‐INT

***Study characteristics***	
Methods	Randomised, parallel group trial
Participants	4065 males (including 602 MSM): 15 to 27 years old at first dose
Interventions	4‐valent Gardasil (Merck): 3 doses at months 0, 2, 6 Adjuvant placebo: 3 doses at months 0, 2, 6
Outcomes	All‐cause mortality Efficacy: penile cancer, anal cancer, intraepithelial neoplasia (anal, penile, perineal or perianal), anogenital warts, persistent HPV infection Adverse events: serious, local, systemic Follow‐up: months 7, 12, 18, 24, 30 and 36
Setting	71 study centres in Australia, Brazil, Canada, Costa Rica, Croatia, Finland, Germany, Mexico, Netherlands, Norway, Peru, Philippines, Portugal, South Africa, Spain, Sweden, Taiwan, USA, (HIC, UMIC, LMIC) National screening programme: yes; coverage: mixed
Notes	Funding: mixed: Merck; National Institutes of Health Conflicts of interest: yes, study authors include employees and stock owners of Merck. One author has an approved, filed, or pending patent related to subject matter discussed in the main publication.

**Table:** 4v Kang 2008‐KOR

***Study characteristics***	
Methods	Randomised, parallel group trial
Participants	176 females: 9 to 23 years old at first dose
Interventions	4‐valent Gardasil (Merck): 3 doses at months 0, 2, 6 Adjuvant placebo: 3 doses at months 0, 2, 6
Outcomes	Adverse events: serious, leading to discontinuation, total, local, systemic Follow‐up: month 7
Setting	10 study centres in South Korea (HIC) National screening programme: yes; coverage: 10% to 50%
Notes	Funding: private/industry: Merck & Company Inc. Conflicts of interest: not reported

**Table:** 4v Li 2012‐CHN

***Study characteristics***	
Methods	Randomised, parallel group trial
Participants	600 females and males: 9 to 45 years old at first dose
Interventions	4‐valent Gardasil (Merck): 3 doses at months 0, 2, 6 Adjuvant placebo: 3 doses at months 0, 2, 6
Outcomes	Adverse events: serious, leading to discontinuation, total, local, systemic Follow‐up: 7 months
Setting	1 study centre in China (UMIC) National screening programme: yes; coverage: unknown
Notes	Funding: private/industry: Merck Sharp & Dohme Corp Conflicts of interest: yes, several authors are reported to have been affiliated with Merck Sharp & Dohme Corporation or Hangzhou MSD Pharmaceutical Co. Ltd.

**Table:** 4v Mikamo 2019‐JPN

***Study characteristics***	
Methods	Randomised, parallel group trial
Participants	1124 males: 16 to 26 years old at first dose
Interventions	4‐valent Gardasil (Merck): 3 doses at months 0, 2, 6 Adjuvant placebo: 3 doses at months 0, 2, 6
Outcomes	All‐cause mortality Persistent HPV infection Adverse events: fatal, serious, leading to discontinuation, total, local, systemic Follow‐up: 7 months, 36 months, to data cut‐off (total 56.9 months duration)
Setting	24 study centres in Japan (HIC) National screening programme: yes; coverage: 10% to 50%
Notes	Funding: private/industry: Merck Sharp & Dohme Corp Conflicts of interest: yes, study authors include employees of MSD K.K., Tokyo, Japan

**Table:** 4v Mugo 2015‐AF

***Study characteristics***	
Methods	Randomised, parallel group trial
Participants	100 females: 9 to 12 years old at first dose
Interventions	4‐valent Gardasil (Merck): 3 doses at months 0, 2, 6 Adjuvant placebo: 3 doses at months 0, 2, 6
Outcomes	All‐cause mortality Adverse events: serious, leading to discontinuation, total, local, systemic Follow‐up: month 7
Setting	Study centres (number not reported) in Ghana, Kenya and Senegal (LMIC) National screening programme: mixed
Notes	Funding: private/industry: Merck & Co., Inc Conflicts of interest: yes, study authors include employees and potential stock or stock option holders of Merck

**Table:** 4v NCT00411749 2006‐JPN

***Study characteristics***	
Methods	Randomised, parallel group trial
Participants	107 females: 9 to 17 years old at first dose
Interventions	4‐valent Gardasil (Merck): 3 doses at months 0, 2, 6 Adjuvant placebo: 3 doses at months 0, 2, 6
Outcomes	Adverse events: serious, local, systemic Follow‐up: month 7
Setting	8 study centres in Japan (HIC) National screening programme: yes; coverage: 10% to 50%
Notes	Results analysed with 4v Yoshikawa 2013‐JPN reported under 4v Yoshikawa/NCT00411749 2013‐JPN Funding: private/industry: Merck Sharp & Dohme LLC Conflicts of interest: not reported

**Table:** 4v Reisinger 2007‐INT

***Study characteristics***	
Methods	Randomised, parallel group trial
Participants	1781 females and males: 9 to 15 years old at first dose
Interventions	4‐valent Gardasil (Merck): 3 doses at months 0, 2, 6 Saline placebo: 3 doses at months 0, 2, 6
Outcomes	Adverse events: serious, total, local, systemic Follow‐up: 18 months, 30 months
Setting	47 study centres in Colombia, Denmark, Mexico, Norway, Portugal, Spain, Taiwan, Thailand, UK, USA (HIC, UMIC) National screening programme: mixed
Notes	Funding: Private/Industry: Merck Sharp & Dohme LLC Conflicts of interest: yes, several authors were affiliated to Merck Research Laboratories

**Table:** 4v Senders 2016‐USA

***Study characteristics***	
Methods	Randomised, parallel group trial
Participants	2499 females and males: 11 to 18 years old at first dose
Interventions	4‐valent Gardasil (Merck): 3 doses at months 0, 2, 6 + saline placebo 4‐valent Gardasil (Merck): 3 doses at months 0, 2, 6 + Bivalent rLP2086 Meningococcal Serogroup B Vaccine Bivalent rLP2086 Meningococcal Serogroup B Vaccine: 3 doses at months 0, 2, 6
Outcomes	All‐cause mortality Adverse events: serious, leading to discontinuation, total, local, systemic Follow‐up: 7 months
Setting	63 study centres in USA (HIC) National screening programme: yes; coverage: at least 70%
Notes	Funding: private/industry: Pfizer Inc Conflicts of interest: yes, study authors include employees of Pfizer

**Table:** 4v Villa/FUTURE I/II 2009‐INT

***Study characteristics***	
Methods	Pooled analysis of included RCTs 4v FUTURE 2007‐INT, 4v FUTURE II 2007‐INT and 4v Villa 2005‐INT
Participants	18,174 females: 16 to 26 years old at first dose
Interventions	4‐valent Gardasil (Merck): 3 doses at months 0, 2, 6 Adjuvant placebo: 3 doses at months 0, 2, 6
Outcomes	Efficacy: adenocarcinoma in situ, intraepithelial neoplasia (CIN2, CIN3, VIN2+, VaIN2+) Follow‐up: up to 48 months
Setting	24 countries (HIC, UMIC, LMIC) National screening programme: unknown, not all countries were reported
Notes	Funding: private/industry: Merck Conflicts of interest: yes, study authors include employees of Merck and recipients of consulting fees, advisory board fees, lecture fees, or grant support from GlaxoSmithKline and/or Merck

**Table:** 4v Villa 2005‐INT

***Study characteristics***	
Methods	Randomised, parallel group trial
Participants	552 females: 16 to 23 years old at first dose
Interventions	4‐valent Gardasil (Merck), 3 doses at month 0, 2, 6 Adjuvant placebo, 3 doses at month 0, 2, 6
Outcomes	Efficacy: anogenital warts, persistent HPV infection Adverse events: serious, local, systemic Follow‐up: 7, 36, 60 months
Setting	Study centres in Brazil, Finland, Norway, Sweden, USA (number of centres not reported) (HIC, UMIC) National screening programme: yes; coverage: at least 70%
Notes	Funding: private/Industry: Merck Research Laboratories Conflicts of interest: yes, authors include employees and potential stock or stock options owners of Merck

**Table:** 4v Wei 2019‐CHN

***Study characteristics***	
Methods	Randomised, parallel group trial
Participants	3006 females: 20 to 45 years old at first dose
Interventions	4‐valent Gardasil (Merck): 3 doses at months 0, 2, 6 Adjuvant placebo: 3 doses at months 0, 2, 6
Outcomes	All‐cause mortality Efficacy: intraepithelial neoplasia (CIN2+), persistent HPV infection Adverse events: serious, leading to discontinuation, total, local, systemic Pregnancy outcomes Follow‐up: 30, 78, 90 months
Setting	6 study centres in China (UMIC) National screening programme: yes; coverage: unknown
Notes	Funding: private/industry: Merck Sharp & Dohme Corp Conflicts of interest: yes, study authors include employees of Merck

**Table:** 4v Yoshikawa/NCT00411749 2013‐JPN

***Study characteristics***	
Methods	Pooled analysis of included RCTs 4v NCT00411749 2006‐JPN and 4v Yoshikawa 2013‐JPN
Participants	107 females aged 9 to 17 years old at first dose and 1021 females aged 18 to 26 years old at first dose
Interventions	4‐valent Gardasil (Merck): 3 doses at months 0, 2, 6 Adjuvant placebo: 3 doses at months 0, 2, 6
Outcomes	Adverse events: serious, local, systemic Follow‐up: month 7
Setting	14 study centres in Japan (HIC) National screening programme: yes; coverage: 10% to 50%
Notes	Funding: private/industry: Merck Conflicts of interest: yes, study authors include employees of MSD (subsidiary of Merck & Co., Inc.)

**Table:** 4v Yoshikawa 2013‐JPN

***Study characteristics***	
Methods	Randomised, parallel group trial
Participants	1021 females: 18 to 26 years old at first dose
Interventions	4‐valent Gardasil (Merck): 3 doses at months 0, 2, 6 Adjuvant placebo: 3 doses at months 0, 2, 6
Outcomes	Persistent HPV infection Adverse events: fatal, serious, total, local, systemic Pregnancy outcomes Follow‐up: 30 months
Setting	14 study centres in Japan (HIC) National screening programme: yes; coverage: 10% to 50%
Notes	Results analysed with 4v NCT00411749 2006‐JPN reported under 4v Yoshikawa/NCT00411749 2013‐JPN Funding: private/industry: Merck Conflicts of interest: yes, study authors include employees of MSD (subsidiary of Merck & Co., Inc.)

**Table:** 9v Iversen 2016‐INT

***Study characteristics***	
Methods	Randomised, parallel group trial
Participants	602 females: 9 to 14 years old at first dose
Interventions	9‐valent Gardasil‐9 (Merck): 2 doses at months 0, 6 9‐valent Gardasil‐9 (Merck): 3 doses at months 0, 2, 6
Outcomes	Adverse events: fatal, serious, local, systemic Follow‐up: 12 months, 37 months
Setting	52 study centres in Canada, Chile, Colombia, Czech Republic, Denmark, Israel, Malaysia, Norway, South Korea, South Africa, Spain, Taiwan, Thailand, Turkey, United States (HIC, UMIC) National screening programme: mixed
Notes	Funding: private/industry: Merck & Co Conflicts of interest: yes, study authors include employees and stock and/or stock options owners of Merck

**Table:** Cecolin 2v Hu 2020‐CHN

***Study characteristics***	
Methods	Randomised, parallel group trial
Participants	605 females: 9 to 14 years old at first dose
Interventions	2‐valent Cecolin (Innovax): 2 doses at months 0, 6 2‐valent Cecolin (Innovax): 3 doses at months 0, 1, 6
Outcomes	Adverse events: serious, unsolicited, local, systemic Follow‐up: 7 months
Setting	1 study centre in China (UMIC) National screening programme: yes; coverage: unknown
Notes	Funding: mixed: the National Natural Science Foundation of China; the Chinese National Major Scientific and Technological Special Project for “Significant New Drugs Development”; the Fujian Provincial Major Scientific and Technological Project; Xiamen Innovax Conflicts of interest: yes, study authors include employees of Xiamen Innovax

**Table:** Cecolin 2v Qiao 2020‐CHN

***Study characteristics***	
Methods	Randomised, parallel group trial
Participants	7372 females: 18 to 45 years old at first dose
Interventions	2‐valent Cecolin (Innovax): 3 doses at months 0, 1, 6 Recombinant hepatitis E vaccine: 3 doses at months 0, 1, 6
Outcomes	All‐cause mortality Efficacy: intraepithelial neoplasia (CIN2+, VIN2+, or VaIN2+), persistent HPV infection, incident HPV infection Adverse events: fatal, serious, unsolicited, local, systemic Pregnancy outcomes Follow‐up: 42 months
Setting	5 study centres in China (UMIC) National screening programme: yes; coverage: unknown
Notes	Funding: mixed: the Chinese National High‐tech R&D Program; the Chinese National Major Scientific and Technological Special Project for “Significant New Drug Development”; the National Natural Science Foundation of China; the Fujian Provincial Major Scientific and Technological Project; the Chinese Academy of Medical Sciences Innovation Fund for Medical Sciences; Xiamen Innovax Conflicts of interest: yes, study authors include employees of Xiamen Innovax

**Table:** Cecolin 2v Wu 2015‐CHN

***Study characteristics***	
Methods	Randomised, parallel group trial
Participants	1600 females: 18 to 25 years old at first dose
Interventions	2‐valent Cecolin (Innovax): 3 doses at months 0, 1, 6, 30 μg 2‐valent Cecolin (Innovax): 3 doses at months 0, 1, 6, 60 μg 2‐valent Cecolin (Innovax): 3 doses at months 0, 1, 6, 90 μg HBV vaccine: 3 doses at months 0, 1, 6
Outcomes	Adverse events: fatal, serious, unsolicited, local, systemic Follow‐up: 7 months
Setting	1 study centres in China (UMIC) National screening programme: yes; coverage: unknown
Notes	Funding: public/non‐profit: the National Major Scientific and Technological Special Project, the Fujian Provincial Major Scientific and Technological Project, the Xiamen Scientific Project Conflicts of interest: yes, some study authors are affiliated with the vaccine developer (Xiamen Innovax Biotech Company)

**CIN:** cervical intraepithelial neoplasia; **CIN2:** cervical intraepithelial neoplasia grade 2; **CIN2+:** cervical intraepithelial neoplasia grade 2 or higher; **CIN3:** cervical intraepithelial neoplasia grade 3; **CIN3+:** cervical intraepithelial neoplasia grade 3 or higher; **dTpa‐IPV:** booster vaccine containing reduced diphtheria and pertussis antigen levels, tetanus and inactivated poliovirus; **DTPa:** diphtheria, tetanus, acellular pertussis vaccine; **GSK:** GlaxoSmithKline; **HAV:** hepatitis A virus; **HBV:** hepatitis B virus; **HIC:** high‐income country/ies; **HIV:** human immunodeficiency virus; **HPV:** human papillomavirus; **LMIC:** lower middle‐income country/ies; **MC4‐CRM:** quadrivalent conjugate meningococcal vaccine (MENVEO, GSK); **MenACYW‐TT:** meningococcal conjugate tetanus toxoid vaccine (Sanofi Pasteur); **MMR:** measles, mumps and rubella vaccine; **MSD:** Merck Sharp & Dohme; **MSM:** men who have sex with men; **POTS:** postural orthostatic tachycardia syndrome; **SAEs:** serious adverse events; **Tdap:** tetanus, diphtheria, acellular pertussis vaccine; **UMIC:** upper middle‐income country; **VaIN:** vaginal intraepithelial neoplasia; **VIN:** vulval intraepithelial neoplasia

### Characteristics of excluded studies [ordered by study ID]

**Table:**

Study	Reason for exclusion
Basu 2021	Ineligible study design: RCT which became an observational cohort study based on number of doses received by default after it was halted.
Brown 2012	Intervention/comparison not relevant to this review. Participants were randomised in a 1 to 1 ratio to receive HPV 4 vaccine in the standard (0, 2, 6 months) or a modified schedule (0, 3, 6 months). Only reports on immunogenicity.
Chen 2020	Reports on immunogenicity only (no safety or efficacy outcomes) for included studies Cecolin 2v Hu 2020‐CHN and Cecolin 2v Qiao 2020‐CHN.
Donken 2019	Reports on immunogenicity only (no safety or efficacy outcomes) for included study 4v Dobson 2013‐CAN.
Emeny 2002	Intervention/comparison not relevant to this review. Three versus four doses HPV 11 virus‐like particle vaccine (and reports on immunogenicity only; no safety or efficacy outcomes).
Esposito 2011	Intervention/comparison not relevant to this review. Cervarix at months 0, 1 and 6 versus at months 0, 1 and 12.
EUCTR2012‐004007‐13	Ineligible population: people with recurrent genital warts (study also known as NCT20101090 but not listed on the ClinicalTrials.gov website)
Feder 2019	Intervention/comparison not relevant to this review. Screening trial; HPV vaccination was not randomised.
Ferris 2014	Intervention/comparison not relevant to this review. Early vaccinated versus late vaccinated (previous placebo) in a follow‐up study: all received three doses 4‐valent HPV vaccine.
Ferris 2017	Intervention/comparison not relevant to this review. Early versus late (originally placebo) vaccination.
Folschweiller 2019	Pooled analysis on immunogenicity only (no safety or efficacy outcomes) for included study 2v Romanowski 2011‐CAN/GER and excluded study Puthanakit 2016.
Garland 2007	Intervention/comparison not relevant to this review. Consistency lots (batches) of Gardasil with immunobridging to monovalent vaccine (and reports on immunogenicity only; no safety or efficacy outcomes).
Giacomet 2014	Ineligible design: non‐randomised
Giuliano 2007	Pooled analysis reporting on immunogenicity only (no safety or efficacy outcomes) for included studies 2v Harper 2004‐BRA/NA; 4v FUTURE 2007‐INT; 4v FUTURE II 2007‐INT; 4v Reisinger 2007‐INT, and excluded study (NCT00092495 comparing Gardasil formulations without any control group).
Godi 2019	Reports on immunogenicity only (no safety or efficacy outcomes) for included study 2v4v Draper 2013‐UK.
Harro 2001	HPV vaccine not relevant to this review, monovalent HPV vaccine
Hillman 2012	Reports on immunogenicity only (no safety or efficacy outcomes) for included study 4v Giuliano 2011‐INT.
Hu 2022	Follow‐up analysis of included study 2v CVT 2011‐CRI, but the comparison is not randomised: new, non‐randomised, unvaccinated control group.
Kosalaraksa 2015	Intervention/comparison not relevant to this review. Gardasil‐9 concomitant versus staggered with DTP‐polio vaccine; HPV vaccine in both groups.
Koutsky 2002	HPV vaccine not relevant to this review: monovalent HPV vaccine
Krajden 2011	Reports on immunogenicity only (no safety or efficacy outcomes) for included study 4v Dobson 2013‐CAN
Krajden 2014	Reports on immunogenicity only (no safety or efficacy outcomes) for included study 4v Dobson 2013‐CAN
Kreimer 2015	Ineligible study design: not a randomised comparison: fewer than 3 doses among those randomised to 3 doses; pooled data from CVT and PATRICIA trials
Lamontagne 2013	Intervention/comparison not relevant to this review. All arms 4‐valent comparing alternative dosing schedules (0, 2, 6 months; 0, 3, 9 months; 0, 6, 12 months; or 0, 12, 24 months).
Lazcano‐Ponce 2018	Reports on immunogenicity only (no safety or efficacy outcomes). Traditional (0‐2‐6) versus alternative (0‐6‐50) schedule. Data reported for 2 versus 3 doses but only immunogenicity data. All safety data are for the booster (3rd) dose only.
Lehtinen 2017	Ineligible study design: long‐term registry‐based follow‐up of three trials
Lin 2014	Intervention/comparison not relevant to this review: compares different dosing schedules of 3 doses in both groups
Markowitz 2023	Narrative review article
Neuzil 2011	Intervention/comparison not relevant to this review: four different 3‐dose schedules of quadrivalent vaccine (comparing intervals)
Nygard 2015	Reports on immunogenicity only (no safety or efficacy outcomes). FUTURE II extension study
Ogilvie 2017	Reports on immunogenicity only (no safety or efficacy outcomes) for included study 4v Dobson 2013‐CAN
Olsson 2007	Intervention/comparison not relevant to this review. Extension study: those vaccinated received another 3 doses, those who received placebo received 3 doses of vaccine = 6 doses versus 3 doses.
Olsson 2020	Ineligible study design: extension study where only the vaccine group was followed up
Panagiotou 2015	Ineligible study design: bivalent versus unexposed to bivalent, non‐randomised comparison from RCT where placebo group was offered vaccination at end of blinded phase + unvaccinated cohort
Petaja 2011	Intervention/comparison not relevant to this review: compared one of three consecutive production lots of Cervarix, no placebo/control.
Petersen 2017	Reports on immunogenicity only (no safety or efficacy outcomes). Combined analysis of five phase III clinical trials: included 4v9v Joura 2015‐INT; included 4v9v Vesikari 2015‐EU; excluded NCT00943722 (comparing consistency lots of Gardasil‐9); excluded NCT00988884 and NCT01073293 (comparing Gardasil‐9 with or without other interventions).
Puthanakit 2016	Intervention/comparison not relevant to this review: compared intervals between doses
Rowhani‐Rahbar 2012	Ineligible study design: reports on one arm only, monovalent vaccine
Safaeian 2013a	Ineligible study design: nested case‐control study of participants in the HPV vaccinated arm of the CVT trial who received all three HPV vaccine doses
Safaeian 2013b	Reports on immunogenicity only (no safety or efficacy outcomes). Follow‐up of included study 2v CVT 2011‐CRI, only immunogenicity outcomes
Sankaranarayanan 2018	Ineligible study design: interrupted trial analysing those who received different number of doses by default due to interruption
Schilling 2015	Intervention/comparison not relevant to this review: concomitant versus non‐concomitant administration of 9‐valent HPV vaccine and meningococcal and Tdap vaccines; HPV vaccine in all groups.
Schwarz 2017	Ineligible study design: follow‐up of vaccinated arm only
Schwarz 2019	Ineligible study design: follow‐up of vaccinated arm only
Watson‐Jones 2012	Intervention/comparison not relevant to this review: class‐based versus age‐based delivery
Wheeler 2011	Intervention/comparison not relevant to this review: co‐administration trial with Cervarix in all arms: tetanus toxoid, reduced diphtheria toxoid, and acellular pertussis vaccine and/or 1 dose of meningococcal polysaccharide diphtheria toxoid conjugate vaccine.
Yu 2020	Reports on immunogenicity only (no safety or efficacy outcomes) for included study Cecolin 2v Qiao 2020‐CHN
Zimmerman 2010	Intervention/comparison not relevant to this review: HPV vaccine in both groups, comparing schedules 0, 2 and 6 months versus 0, 2 and 12 months.

**DTP:** diphtheria, tetanus and pertussis vaccine; **HPV:** human papillomavirus; **RCT:** randomised controlled trial; **Tdap:** tetanus, diphtheria, acellular pertussis vaccine

### Characteristics of studies awaiting classification [ordered by study ID]

**Table:** 4v‐Cecolin Zaman 2024‐BGD/GHA

Methods	Randomised, parallel group trial
Participants	9‐ to 14‐year‐old females
Interventions	2‐valent Cecolin (Innovax), 2 doses at 0 and 6 months 2‐valent Cecolin (Innovax), 2 doses at 0 and 12 months 2‐valent Cecolin (Innovax), 2 doses at 0 and 24 months 4‐valent Gardasil (Merck) + 2‐valent Cecolin (Innovax), 2 doses at 0 (Gardasil) and 24 months (Cecolin) Active control: 4‐valent Gardasil (Merck), 2 doses at 0 and 6 months
Outcomes	Immunogenicity; adverse events Follow‐up: 24 months Reports on critical outcome SAEs, but results are not notably different from high‐certainty results synthesised in the review.
Notes	Sponsor: PATH; Collaborators: International Centre for Diarrhoeal Disease Research, Bangladesh, Malaria Research Centre, Agogo Presbyterian Hospital, Ghana, Frederick National Laboratory for Cancer Research, Leidos Biomedical Research, Inc., USA, The Emmes Company, LLC Xiamen Innovax Biotech Co., Ltd. From update search September 2024. To be added at next review update.

**Table:** 9v Berenson 2024‐USA

Methods	Single‐blinded, randomised non‐inferiority trial
Participants	438 females 15 to 26 years old
Interventions	Gardasil‐9, 2 doses at 0 and 6 months Gardasil‐9, 3 doses at 0, 2, and 6 months
Outcomes	Safety and immunogenicity Follow‐up: 1 month Reports on critical outcome SAEs, but no events were observed, which will not have an impact on high‐certainty results synthesised in the review.
Notes	Interim analysis From update search September 2024. To be added at next review update.

**Table:** 9v MacCosham 2022‐CAN

Methods	Randomised, parallel group trial
Participants	18‐ to 45‐year‐old sexually active couples
Interventions	Gardasil‐9 (Merck), 2 doses Hepatitis A vaccine (Sanofi Pasteur) + placebo saline injection
Outcomes	HPV genital and oral infection Follow‐up: 12 months The study does not report on any critical outcomes for the review.
Notes	Sponsor: McGill University NCT01824537 From update search September 2024. To be added at next review update.

**Table:** Barnabas 2023

Methods	‐
Participants	‐
Interventions	‐
Outcomes	Reports on critical outcome SAEs, but results are not notably different from high‐certainty results synthesised in the review.
Notes	Companion paper identified for included study 2v9v KENSHE 2021‐KEN in the update search September 2024. To be included in next update of review.

**Table:** HPV‐003

Methods	Randomised, parallel group trial
Participants	61 females 18 to 30 years old who were DNA positive for HPV 16 and/or 18 and had a normal Pap smear or no greater than ASCUS with colposcopy negative for CIN or SIL.
Interventions	MEDI‐517 40 μg SBAS4, 3 doses at months 0, 1, 6 Adjuvant placebo, 3 doses at months 0, 1, 6
Outcomes	All‐cause mortality Adverse events: serious, leading to discontinuation, local, systemic Follow‐up: 30 days for safety
Notes	NCT00263744 Unclear if the intervention (MEDI‐517) is Cervarix. Emailed GSK on 19 April 2023 to enquire about the intervention.

**Table:** NCT00520598

Methods	Randomised, parallel group trial
Participants	511 females 16 to 26 years
Interventions	Gardasil, 3 doses at months 0, 2 and 6 V505 formulation 1, 3 doses at months 0, 2 and 6 V505 formulation 2, 3 doses at months 0, 2 and 6 V505 formulation 2, 2 doses at months 0 and 6 + placebo at month 2 V505 formulation 3, 2 doses at months 0 and 6 + placebo at month 2
Outcomes	GMTs (planned) Follow‐up: 4 weeks after last dose
Notes	V505‐001 Completed May 2011; no data available Unclear if V505 is Gardasil‐9 or a different vaccine E‐mailed Merck Sharp & Dohme on 19 April 2023 to enquire about the V505 intervention

**Table:** NCT05149248

Methods	Mixed‐methods study, including randomised, parallel group assignment
Participants	6000 MSM, transgender women, people living on the street (homeless), or people who have suffered rape, with or without HIV infection, from 14 to 45 years old
Interventions	Gardasil, 1 dose Gardasil, 2 doses at month 0, and 6 Gardasil (delayed), 1 dose at month 12
Outcomes	HPV infection (planned) STIs (planned) Follow‐up: 18 months
Notes	Completed November 2019, no efficacy or safety results available. Uptake and acceptability results (PMID: 31657665) and non‐randomised HPV‐infection results in transgender women (PMID: 34993304) have been published. Emailed investigator on 19 April 2023 to enquire about safety and efficacy data. Investigator replied that safety analysis has not been done and another publication is pending.

**Table:** Rivera 2018

Methods	Randomised, parallel group trial
Participants	1300 females 9 to 25 years old
Interventions	MenACWY‐TT 1d. Month 0 + Cervarix 3d. Month 1, 2, 7 MenACWY‐TT 1d. Month 0 + Cervarix 3d. Month 0, 1, 6 Cervarix 3d. Month 0, 1, 6 MenACWY‐TT 1d. Month 0 + Tdap 1d. Month 0 + Cervarix 3d. Month 0, 1, 6 Tdap 1d. Month 0 + Cervarix 3d. Month 0, 1, 6
Outcomes	Immunogenicity Adverse events: serious, unsolicited, local, systemic Follow‐up: 8 months
Notes	Cervarix was administered in all five arms: in one arm at month 1, whereas in the other arm at month 0, so it was possible to compare outcomes before dose administration at month 1. We have enquired whether such data exist.

**Table:** Shing 2022

Methods	‐
Participants	‐
Interventions	‐
Outcomes	Follow‐up analysis where the main focus of the paper is long‐term data comparing against a new, non‐randomised, unvaccinated control group. The paper reports on critical outcomes CIN2+ and CIN3+, but very similar outcome results from this study are already included in the review from main analyses.
Notes	Companion paper identified for included study 2v CVT 2011‐CRI in the update search September 2024. To be included in next update of review.

**Table:** V503‐018

Methods	Unclear, possibly RCT
Participants	Unclear, possibly 9‐ to 15‐year‐old boys and girls
Interventions	Unclear, possibly Gardasil or Gardasil‐9
Outcomes	Unclear, possibly GMTs
Notes	V503‐018 is cited in an FDA licence application supplement for Gardasil, table 21 (http://www.fda.gov/downloads/BiologicsBloodVaccines/Vaccines/ApprovedProducts/UCM190977.pdf); however, it is not listed among sources on page 10. V501‐018 is listed among the sources and is a Gardasil study (included in our review as 4v Reisinger 2007‐INT). V501 denotes Gardasil whereas V503 denotes Gardasil‐9. E‐mailed Merck Sharp & Dohme on 19 April 2023 to enquire about the trial.

**Table:** Watson‐Jones 2023

Methods	‐
Participants	‐
Interventions	‐
Outcomes	Reports on critical outcome SAEs, but results are not notably different from high‐certainty results synthesised in the review.
Notes	Companion paper (conference abstract) identified for included study 2v9v DoRIS 2022‐TZN in the update search September 2024. To be included in next update of review.

**Table:** Yao 2022

Methods	‐
Participants	‐
Interventions	‐
Outcomes	Reports on critical outcome SAEs, but results are not notably different from high‐certainty results synthesised in the review.
Notes	Companion paper identified for included study Cecolin 2v Hu 2020‐CHN in the update search September 2024. To be included in next update of review.

**Table:** Zhao 2022a

Methods	‐
Participants	‐
Interventions	‐
Outcomes	Longer‐term follow‐up of critical outcomes CIN and vaginal intraepithelial neoplasia (VaIN), but no new events since data collected at previous follow‐up, which are already in the review.
Notes	Companion paper identified for included study 4v Wei 2019‐CHN in the update search September 2024. To be included in next update of review.

**Table:** Zhao 2022b

Methods	‐
Participants	‐
Interventions	‐
Outcomes	Longer‐term follow‐up for combination outcome high‐grade CIN, vaginal intraepithelial neoplasia (VaIN) or vulvar intraepithelial neoplasia (VIN), but results are not notably different from results already presented in the review. Longer‐term follow‐up for critical outcome SAEs, but results are not notably different from high‐certainty results already synthesised in the review.
Notes	Companion paper identified for included study Cecolin 2v Qiao 2020‐CHN in the update search September 2024. To be included in next update of review.

**Table:** Zhao 2023

Methods	‐
Participants	‐
Interventions	‐
Outcomes	Reports on 10‐year follow‐up of critical outcome CIN2+ but results are not notably different from 72‐month data already in review.
Notes	Companion paper identified for included study 2v Zhu 2014‐CHNa in the update search September 2024. To be included in next update of review.

**Table:** Zhong 2023

Methods	‐
Participants	‐
Interventions	‐
Outcomes	Post hoc analysis. No critical outcomes are reported in this paper; it focuses on pregnancy‐related events.
Notes	Companion paper identified for included study Cecolin 2v Qiao 2020‐CHN in the update search September 2024. To be included in next update of review.

**ASCUS:** atypical squamous cells of undetermined significance; **CIN:** cervical intraepithelial neoplasia; **d:** dose; **FDA:** Food and Drug Administration; **GMT:** geometric mean titre; **GSK:** GlaxoSmithKline; **HPV:** human papillomavirus; **MSM:** men who have sex with men; **RCT:** randomised controlled trial; **SAEs:** serious adverse events; **SIL:** squamous intraepithelial lesion; **STI:** sexually‐transmitted infection; **Tdap:** tetanus, diphtheria, acellular pertussis vaccine

### Characteristics of ongoing studies [ordered by study ID]

**Table:** Add‐Vacc

Study name	Adding male single dose HPV vaccination to female HPV vaccination in Tanzania (Add‐Vacc)
Methods	Cluster‐randomised trial
Participants	14‐ to 18‐year‐old males
Interventions	1. Gardasil (Merck), single dose 2. No intervention Co‐intervention: girls in all clusters were offered 2 doses 4‐valent Gardasil (Merck) as part of the national immunisation programme
Outcomes	HPV DNA prevalence; immunogenicity; safety and tolerability; coverage; acceptability; cost‐effectiveness
Starting date	August 2022; estimated completion: October 2026
Contact information	Not reported
Setting	Tanzania
Notes	Sponsor: London School of Hygiene and Tropical Medicine

**Table:** ESCUDDO

Study name	Comparing one or two doses of the human papillomavirus vaccine for the prevention of human papillomavirus infection, ESCUDDO Study (ESCUDDO)
Methods	Randomised, parallel group trial
Participants	12‐ to 16‐year‐old girls
Interventions	1. Gardasil (Merck), 1 dose + DTaP, 1 dose 2. Cervarix (GSK), 1 dose + DTaP, 1 dose 3. Gardasil (Merck), 2 doses 4. Cervarix (GSK), 2 doses 5. No intervention (epidemiological survey)
Outcomes	HPV cervicovaginal infection; immunogenicity; adverse events; cost‐effectiveness Follow‐up: 60 months
Starting date	November 2017; estimated completion: August 2025
Contact information	Carolina Porras: cporras@acibcr.com
Setting	Costa Rica
Notes	Sponsor: National Cancer Institute (NCI); Collaborator: Bill and Melinda Gates Foundation

**Table:** Giuliano 2022

Study name	Efficacy against oral persistent infection, immunogenicity and safety of the 9‐valent human papillomavirus vaccine (9vHPV) in men aged 20–45 years (V503‐049)
Methods	Randomised, parallel group trial
Participants	20‐ to 45‐year‐old males
Interventions	1. Gardasil‐9 (Merck), 3 doses 2. Placebo saline injection
Outcomes	HPV oral infection; immunogenicity; adverse events Follow‐up: 42 months
Starting date	February 2020; estimated completion: August 2024
Contact information	Yingmei Tu: ying.mei.tu@merck.com
Setting	USA
Notes	Sponsors and Collaborators: Merck Sharp & Dohme LLC

**Table:** HANDS

Study name	HPV vaccination in Africa – new delivery schedules alias the HANDS HPV vaccine trial (HPV)
Methods	Randomised, parallel group trial
Participants	4‐ to 26‐year‐old females
Interventions	1. 9‐valent Gardasil‐9 (Merck), 3 doses, 15 to 26 years 2. 9‐valent Gardasil‐9 (Merck), 2 doses, 9 to 14 years 3. 9‐valent Gardasil‐9 (Merck), 2 doses, 4 to 8 years 4. 9‐valent Gardasil‐9 (Merck), 1 dose, 9 to 14 years 5. 9‐valent Gardasil‐9 (Merck), 1 dose, 4 to 8 years
Outcomes	Immunogenicity; adverse events Follow‐up: 36 months
Starting date	September 2019; estimated completion: June 2024
Contact information	Ed Clarke, London School of Hygiene and Tropical Medicine
Setting	The Gambia
Notes	Sponsor: London School of Hygiene and Tropical Medicine; Collaborators: Public Health England, University of Cambridge, Merck Sharp & Dohme LLC

**Table:** ICI‐VPH

Study name	ICI‐VPH: impact of HPV immunisation schedules against HPV (ICI‐VPH)
Methods	Randomised, parallel group trial
Participants	9‐ to 14‐year‐old females
Interventions	1. 4‐valent Gardasil (Merck), 3 doses 2. 4‐valent Gardasil (Merck), 2 doses
Outcomes	Persistent HPV infection; immunogenicity Follow‐up: 60 months
Starting date	November 2013; completed December 2021
Contact information	Chantal Sauvageau, CHU de Quebec‐Universite Laval
Setting	Canada
Notes	Sponsor: CHU de Quebec‐Universite Laval; Collaborators: Centre hospitalier de l'Université de Montréal (CHUM), Ministere de la Sante et des Services Sociaux, Institut National en Santé Publique du Québec

**Table:** NCT03943875

Study name	GARDASIL 9: 3 dose vs. 2 dose with delayed 3rd dose
Methods	Randomised, parallel group trial
Participants	15‐ to 26‐year‐old females and males
Interventions	1. Gardasil‐9 (Merck), 3 doses, females 2. Gardasil‐9 (Merck), 2nd dose with delayed 3rd dose, females 3. Gardasil‐9 (Merck), 3 doses, males 4. Gardasil‐9 (Merck), 2nd dose with delayed 3rd dose, males
Outcomes	Immunogenicity Follow‐up: 12 months
Starting date	June 2019; estimated completion: March 2025
Contact information	Abbey B Berenson: abberens@utmb.edu; Rosalina M Trujillo: rmtrujil@UTMB.EDU
Setting	USA
Notes	Sponsor: University of Texas Medical Branch, Galveston; Collaborator: Cancer Prevention Research Institute of Texas

**Table:** NCT03998254

Study name	Efficacy, immunogenicity and safety of V503 in Chinese women aged 20–45 years (V503‐023)
Methods	Randomised, parallel group trial
Participants	20‐ to 45‐year‐old females
Interventions	1. Gardasil‐9 (Merck), 3 doses 2. Gardasil (Merck), 3 doses
Outcomes	CIN2+; persistent HPV infection; immunogenicity; adverse events; HPV‐related definitive therapy Follow‐up: 30 months
Starting date	June 2019; estimated completion: March 2028
Contact information	Medical Director Merck Sharp & Dohme LLC
Setting	China
Notes	Sponsors and Collaborators: Merck Sharp & Dohme LLC

**Table:** NCT04635423

Study name	Efficacy, immunogenicity, and safety study of the 9vHPV vaccine in Japanese males (V503‐064)
Methods	Randomised, parallel group trial
Participants	16‐ to 26‐year‐old males
Interventions	1. Gardasil‐9, 3 doses 2. Placebo saline injection
Outcomes	Persistent HPV infection; immunogenicity; adverse events Follow‐up: 42 months
Starting date	November 2020; estimated completion January 2025
Contact information	Tanaka Yoshiyuki, msdjrct@merck.com
Setting	Japan
Notes	Sponsor: Merck Sharp & Dohme LLC

**Table:** NCT04772534

Study name	Immunogenicity and safety of the 9‐valent human papillomavirus (9vHPV) vaccine in Japanese boys and girls (V503‐066)
Methods	Randomised, open‐label clinical study
Participants	9‐ to 15‐year‐old boys and girls
Interventions	1. Gardasil‐9 (Merck), 3 doses boys 2. Gardasil‐9 (Merck), 2 doses girls 3. Gardasil‐9 (Merck), 2 doses boys
Outcomes	Immunogenicity; adverse events Follow‐up: 7 months
Starting date	May 2021; estimated completion April 2024
Contact information	Responsible party: Merck Sharp & Dohme LLC
Setting	Japan
Notes	Sponsor: Merck Sharp & Dohme LLC

**Table:** NCT05279248

Study name	The immunogenicity and safety of human papillomavirus vaccine and measles‐mumps‐rubella vaccine co‐administration
Methods	Randomised, parallel assignment, open‐label, phase 4 clinical trial
Participants	13‐ to 14‐year‐old females
Interventions	1. Cecolin 2d. (0d, 6m) + MMR vaccine 1d. (0d) 2. Cecolin 2d. (0d, 6m) + MMR vaccine 1d. (7m) 3. Cecolin 2d. (0d, 1m) + MMR vaccine 1d. (7m)
Outcomes	Immunogenicity; safety Follow‐up: 7 months
Starting date	August 2021; estimated completion date: June 2023
Contact information	Zhejiang Provincial Center for Disease Control and Prevention
Setting	China
Notes	Sponsor: Zhejiang Provincial Center for Disease Control and Prevention

**Table:** NCT05415345

Study name	Immunogenicity and safety of co‐immunization with Cecolin and Hecolin
Methods	Randomised, parallel assignment, open‐label, phase 4 clinical trial
Participants	18‐ to 25‐year‐old females
Interventions	1. Cecolin, 3 doses (0, 1, 6 month schedule) + Hecolin (hepatitis E vaccine), 3 doses (0, 1, 6 month schedule) 2. Cecolin, 3 doses (0, 1, 6 month schedule) 2. Hecolin, 3 doses (0, 1, 6 month schedule)
Outcomes	Immunogenicity; safety Follow‐up: 7 months
Starting date	October 2021; estimated completion date: June 2023
Contact information	Zhenggang Jiang, Zhejiang Provincial Center for Disease Control and Prevention
Setting	China
Notes	Sponsor: Xiamen Innovax Biotech Co., Ltd.

**Table:** NCT05672927

Study name	Comparing immune response of 2 vs 3 HPV doses (27‐45 years old)
Methods	Randomised, parallel assignment, single blind (outcome assessor) clinical trial
Participants	27‐ to 45‐year‐old females
Interventions	1. Gardasil‐9 (Merck), 3 doses 2. Gardasil‐9 (Merck), 2 doses
Outcomes	Immunogenicity Follow‐up: 12 months
Starting date	January 2023; estimated completion: August 2027
Contact information	Abbey B Berenson: abberens@utmb.edu; Rosalina M Trujillo: rmtrujil@utmb.edu
Setting	USA
Notes	Sponsor: University of Texas Medical Branch, Galveston

**Table:** NCT06345885

Study name	Immunogenicity and safety of one dose of HPV vaccine
Methods	Randomised, parallel assignment, open‐label, phase 4 clinical trial
Participants	9‐ to 14‐year‐old females
Interventions	1. Cecolin, 1 dose 2. Gardasil, 1 dose
Outcomes	Immunogenicity; adverse events Follow‐up: 2 months
Starting date	February 2023; Completion date: January 2024
Contact information	Fujian Provincial Center for Disease Control and Prevention: Xie Fangqin: okxfq@163.com Zhang Dongjuan: dongj8888@163.com
Setting	China
Notes	Sponsor: Xiamen Innovax Biotech Co., Ltd; Fujian Provincial Center for Disease Control and Prevention

**Table:** PRISMA ESCUDDO

Study name	Single‐dose HPV vaccination for the prevention of cervical cancer in young adult women in Costa Rica, the PRISMA ESCUDDO Trial (PRISMA)
Methods	Randomised, parallel assignment, double‐blinded, phase 4 clinical trial
Participants	18‐ to 30‐year‐old females
Interventions	1. Gardasil‐9, 1 dose 2. Cervarix, 1 dose 3. Injection control: DTP vaccine (Adacel), 1 dose
Outcomes	Efficacy; immunogenicity Follow‐up: 6 months
Starting date	March 2022; estimated completion date: May 2026
Contact information	Aimee R Kreimer, National Cancer Institute
Setting	Costa Rica
Notes	Sponsor: National Cancer Institute, USA

**Table:** Salmeron 2016

Study name	FASTER‐Tlalpan Study in Mexico: HPV vaccination impact on cervical cancer screening program (FASTER)
Methods	Randomised, parallel group trial
Participants	25‐ to 45‐year‐old females
Interventions	1. 2‐valent Cervarix (GSK), 2 doses 2. 4‐valent Gardasil (Merck), 2 doses 3. No intervention Co‐interventions: hrHPV‐based screening
Outcomes	HPV 16/18 incidence; CIN grade 2 incidence Follow‐up: 120 months
Starting date	January 2017; completed December 2018
Contact information	Jorge Salmeron, Principal Researcher, Instituto Nacional de Salud Publica, Mexico
Setting	Mexico
Notes	Sponsor: Instituto Nacional de Salud Publica, Mexico NCT03105856 Feasibility results have been published (PMID: 31184976)

**CIN(2+):** cervical intraepithelial neoplasia (grade 2 or higher); **DTaP:** diphtheria, tetanus, and acellular pertussis vaccine; **DTP:** diphtheria, tetanus, and pertussis vaccine; **HPV:** human papillomavirus; **hrHPV:** high‐risk human papillomavirus; **RCT:** randomised controlled trial; **Tdap:** tetanus, diphtheria, acellular pertussis vaccine

## Differences between protocol and review

- Additional interventions to supplement the analysis: we planned to include HPV vaccines not in use (old or experimental) in the NMA to increase the amount of indirect information on the HPV vaccines in use. After creating and inspecting network maps including these additional vaccines, we found that no useful additional information would be added by including them.
- Critical outcome for summary of findings table: we decided to include high‐grade CIN as a composite outcome in the summary of findings table, so that results for both CIN3+ and CIN2+ could be shown. These are critical outcomes for clinical practice.
- Critical outcome: penile (PeIN) or anal (AIN) intraepithelial neoplasia of any grade was changed to high‐grade (grade 2 or 3) PeIN or AIN.
- Critical outcome: in response to peer‐review comments, we clarified treatment rates for CIN, AIN and other HPV‐related pre‐invasive disease as the reduction in treatment rates.
- Sub‐outcome: a separate analysis for serious adverse events related to the vaccines (as assessed and reported in the studies) was planned but reported in tables instead of in a meta‐analysis.
- Adverse events: we planned to extract all reports of adverse events from the study documents to categorise them into the protocol outcome categories. We revised this strategy and extracted only those adverse event sub‐categories listed in our protocol. For example, specific adverse events due to traffic accidents were not extracted.
- Anogenital warts: in addition to the critical outcome 'Anogenital warts', we also reported on HPV 6 and HPV 11 anogenital warts as important outcomes, since HPV types 6 and 11 are responsible for 90% of anogenital warts (Hawkins 2013).
- Participation in cervical screening: was listed in the protocol under secondary outcomes, but it should also have been designated an important outcome and is treated as such in the review.
- 'Cervical cerclage and incompetence' and 'Premature rupture of membranes' have been added as adverse pregnancy outcomes.
- Time‐to‐event data: should there have been time‐to‐event data included in the review, we would have extracted hazard ratios (HRs) and standard errors (SEs) or confidence intervals (CIs) and they would have been combined using the generic inverse‐variance method.
- Rate ratios: should there have been incidence rate data included in the review, we would have calculated rate ratios with 95% CIs for dichotomous clinical outcomes reported as incidence rates.
- Effect modifier: HPV serostatus was changed to HPV DNA positive or negative at first dose.
- Assessment of heterogeneity: we had planned not to pool studies where I^2^ > 75%. In the review, however, we did pool since we thought it would be more helpful for readers. We reported on I^2^ > 50% in the results and downgraded for inconsistency if the effect estimates of included studies were in opposite directions.
- Objectives: in response to peer‐review comments, we simplified the wording of the objectives.
